# Supplementary material for: Research participants’ perception of ethical issues in stroke genomics and neurobiobanking research in Africa
Source: PLoS One. 2025 May 6;20(5):e0292906. doi: 10.1371/journal.pone.0292906 (PMC12054916; doi:10.1371/journal.pone.0292906)
Supplement: S1 File — (PDF) [file pone.0292906.s001.pdf]

African Neurobiobank for Precision Stroke Medicine - Ethical, Legal, and Social Implications (ELSI) Project

FOCUS GROUP DISCUSSION GUIDE

Note: The protocol below outlines the topics that will be covered during the focus groups and provides examples of the types of open-ended questions that we plan to ask. However, we will follow the lead of participants and will ask additional questions if necessary to fully discuss a particular topic, or pursue other topics if they are raised in the group as being important.

1. Welcome	Welcome. I want to thank you for coming today. My name is_________and I will be the facilitator for today's group discussion. I am a ________ and I work for ____________. We also have_______present to take notes for us.<br><br>We are conducting a study among people who have had a stroke, their care givers and other people in the community to identify and look critically at ethical, legal and social issues relating to stroke biobanking in the African context. Therefore we will be asking and discussing issues such as your knowledge, attitude, perceptions, barriers and facilitators influencing ethical, legal and social issues related to the use of blood and stored blood fractions, brain images (CT/MRI) and brain donation in the context of stroke genomic research.<br><br>We invited you to take part in this discussion today because you care for someone who has suffered a stroke within the last one year. We would be talking to you about the issues mentioned above and we would like your suggestions on how to improve on the tools so that they be clearer and more appropriate.<br><br>Whatever we learn from today's discussion will help us develop intervention program to address the ELSI issues related to stroke genomic and biobanking research in Sub Saharan Africa
2. Ground Rules	**Before we begin, I would like to review a few ground rules for the discussion.**<br><br>a. I will ask you several questions and I'd like to give everyone a chance to give their opinions. We do not have to go in any particular order but we do want everyone to take part in the discussion. We ask that only one person speak at a time.<br><br>b. I am interested in your opinions and whatever you have to say is fine with us. There is no right or wrong answers. I am just asking for your opinions and suggestions. I am

	here to learn from you.<br><br>c. Don’t worry about having different opinions from someone else. But please do respect each other’s answers or opinions.<br><br>d. You may choose not to answer any question you do not wish to.<br><br>e. Feel free to treat this as a discussion and to ask questions of each other and to respond to what others are saying, whether you agree or disagree.<br><br>f. I will treat your answers as confidential. I will not to ask for anything that could suggest your identity. I will only use first names during the discussion. I also ask that each of you respect the privacy of everyone in the room and not to say or repeat what is said here in any way that could identify anyone in this room.
Ground Rules<br><br>(continued)	I am recording the discussion today on an audio tape because we don’t want to miss any of your suggestions. However, once we start the audio recorder we will not use anyone’s full name and we ask that you do the same. Facilitators should please take note: [NOTE: ALL PARTICIPANTS WILL HAVE ALREADY AGREED TO THE TAPE RECORDING ON WRITTEN INFORMED CONSENT. IF A PARTICIPANT DECIDES THAT’S/HE DOES NOT WANT TO BE TAPED AND WANTS TO LEAVE, S/HE SHOULD STILL BE GIVEN THE INCENTIVE]<br><br>Finally, this discussion will last about 1 hour and we request that you stay for the entire meeting.<br><br>**Does anyone have any questions before we start?**

REQUEST TO TURN ON RECORDER AT THIS POINT IN THE INTERVIEW

S/N	**Questions**	**Probes**
1	Tell us what you know about genetic research	• Have you heard about it?<br>• What do you know about it?<br>• Tell us about any experiences you or others you know have had with participating in genetic research<br>• What do you know about genetic research in stroke?<br>• Role/benefit of genetic research in medicine
2	Explain what you know about biobanking?	• Probe for level of awareness, understanding/perception of concept, sources of information,<br>• How does biobanking operate?

		• How important is biobanking to medical breakthroughs?<br>• Belief/thought/opinion relating to biobanking<br>• Awareness, understanding/perception of brain banking<br>• Awareness of any policy or law guiding biobanking
3	Can you explain what you understand by precision medicine?	• Probe level of awareness, understanding/perception of concept, benefits, demerits, is it important in Africa?<br>• Can it be applied to stroke disease?<br>• Source of information<br>• Belief/thought/opinion relating to precision medicine<br>• Awareness of any policy or law guiding precision medicine
4	What do you understand by brain donation for research purpose?	• Probe for level of awareness, perception, sources of information, perception of complexity of the procedure, benefits of brain donation, misconceptions, personal willingness to donate<br>• cultural, social and religious belief on donating brain for research purpose<br>• What factors inhibit brain donation? Probe for cultural and religious reasons, peer values, parental influence, level of awareness, legal issues involved, knowledge of where it can be done, familiarity with medical and research settings<br>• What factors promote brain donation? Probe for cultural and religious reasons, peer values, parental influence, level of awareness, legal issues involved, knowledge of where it can be done, familiarity with medical and research settings
5	What do you understand by blood sample donation for genetic research?	• what are your thoughts on blood sample donation for research<br>• Source of information<br>• uses of blood sample donated for research<br>• cultural, social and religious belief on donating blood for genetic research<br>• awareness of any policy or law guiding blood sample

		donation for research and storage
6	Share with us your opinion and thoughts about blood sample donation for stroke genetic research.	• Willingness to be involved in such research<br>• What do you see as the barrier(s) that could hinder your donation of blood sample for stroke genetic research: family member, cultural and religious reasons, peer values, parental influence, level of awareness, legal issues involved, knowledge of where it can be done, familiarity with medical and research settings?<br>• What do you perceive as benefit(s) of giving blood sample for stroke genetic research that could promote your willingness to donate: cultural and religious reasons, peer values, parental influence, level of awareness, legal issues involved, knowledge of where it can be done, familiarity with medical and research settings?<br>• What can you say about your family member or other member of the community willingness to give blood sample for stroke genetic research?<br>• What could be done to make you and more people give blood sample for research: mass media, husband consent, family consent, donors group, peers?
7	Tell us what you know about informed consent?	• What do you know about the consent process for genetic research?<br>• Types of inform consent preferred (broad, restricted, tiered and dynamic). ***Facilitator to please explain each type to the participants<br>• Reason(s) for their choice<br>• Person to be involved before participation<br>• Data use in the incident of death and why<br>• Support for generic consent for community
8	What is your opinion on storage of blood sample and blood fractions for genetic research	• Opinion on storage of blood sample for future use in genetics research
9	Tell us what you know about sharing of data, blood/blood fractions, brain images (CT/MRI) as well as brain tissue samples	• Opinion on sharing data, blood samples, brain images (CT/MRI) or brain tissue samples with another researcher locally and internationally<br>• Commercial or non-commercial use of stored data, blood/ blood fractions, brain images and brain tissue.
10	Share with us your thoughts about return of individual research results and incidental	• What are the ways that you think one can receive the results of genetic research?

	findings	• What are your thoughts on returning individual research results and incidental findings<br>• Opinion on desire for feedback of research results and incidental findings<br>• What are the challenges of returning individual results<br>• Ways they would prefer to get feedback: phones, e mail, letters, feedback by a healthcare worker? a researcher? or a clinician?<br>• What are the ethical, legal and social issues relating to returning individual research results and incidental findings generated by genetic research?
11	Explain your understanding of Biorights	• How much control should/can individuals have regarding how their biological specimens will be used in research?<br>• What rights do/should individuals who provide their specimens for research have over their specimens, how they are used in research, and any profits from research discoveries made possible from them?<br>• How should autonomy rights be best balanced with societal benefits that derive from the use of human specimens in research?
12	What is your opinion about governance and regulation of biobanking?	• Need for ethical committee approval on future use of stored data, blood or brain tissue resource for research<br>• Need to set up a regulatory board
13	Explain possible intervention for implementation of biobanking	• What suggestions do you have that can help raise awareness and improve attitude towards blood sample or brain donation for research and encourage people to adopt the practice?
14	Any other major concern or recommendation on use of blood or brain tissue for research in Nigeria/Ghana.	
15	Brief written survey and<br>Wrap-up (5 minutes)	Thank you for coming today and for sharing your opinions with us. We hope you enjoyed the discussion today. I have a brief survey I'd like you to complete. Let me know if you need help. Please do not put your name on it.<br><br>I'm also going to be handing out the incentives.
