# Supplementary material for: Research participants’ perception of ethical issues in stroke genomics and neurobiobanking research in Africa
Source: PLoS One. 2025 May 6;20(5):e0292906. doi: 10.1371/journal.pone.0292906 (PMC12054916; doi:10.1371/journal.pone.0292906)
Supplement: S2 File — (DOCX) [file pone.0292906.s002.docx]

**SOCIO-DEMOGRAPHIC RESULTS FROM THE ELSI DATA**

|  | **CAB n (%)**  **n=85** | **Caregivers: n (%)**  **n=67** | **Stroke Free: n (%) n=63** | **Stroke Cases n (%) n=54** |
| --- | --- | --- | --- | --- |
| **Site** |  |  |  |  |
| Abeokuta | 7 (8.2) | 7 (10.5) | 6 (9.5) |  |
| Accra | 8 (9.4) | 7 (10.5) | 7 (11.1) |  |
| Ibadan | 22 (25.9) | 6 (9.0) | 7 (11.1) |  |
| Kano | 16 (18.8) | 16 (23.9) | 8 (12.7) |  |
| Kumasi | 9 (10.6) | 8 (11.9) | 14 (22.2) |  |
| Ilorin | 17 (20.0) | 17 (25.4) | 13 (20.6) |  |
| Zaria | 6 (7.1) | 6 (9.0) | 8 (12.7) |  |
| **Mean age (SD)** | 51.0 ($\pm$11.4) | 41.3 ($\pm$13.1) | 42.8 ($\pm$9.8) | 54.5 ($\pm$12.6) |
| **Age group (years)** |  |  |  |  |
| <=40 | 14 (16.5) | 34 (50.8) | 25 (39.7) | 7 (13.2) |
| 41-50 | 33 (38.8) | 19 (28.4) | 25 (39.7) | 13 (24.5) |
| 51-60 | 23 (27.1) | 11 (16.4) | 10 (15.9) | 15 (28.3) |
| 61-70 | 11 (12.9) | 0 | 3 (4.8 | 14 (26.4) |
| 71-80 | 3 (3.5) | 3 (4.5) | 0 | 4 (7.6) |
| 80+ | 1 (1.2) | 0 | 0 | 0 |
| **Gender** |  |  |  |  |
| Male | 66 (77.7) | 30 (44.8) | 40 (63.5) | 37 (68.5) |
| Female | 19 (22.4) | 37 (35.2) | 23 (36.5) | 17 (31.5) |
| **Marital Status** |  |  |  |  |
| Single | 3 (3.5) | 13 (19.4) | 14 (22.2) | 4 (7.6) |
| Married/living with someone | 82 (96.5) | 53 (79.1) | 46 (73.0) | 42 (79.3) |
| Widowed/Divorced | 0(0) | 1 (1.5) | 3 (4.8) | 7 (12 (13.1) |
| **Ethnicity** |  |  |  |  |
| Yoruba | 42 (50.0) | 29 (43.3) | 28 (45.2) | 24 (44.4) |
| Igbo | 2 (2.4) | 1 (1.5) | 2 (3.2) | 3 (5.6) |
| Hausa | 23 (27.4) | 16 (23.9) | 17 (27.4) | 10 (18.5) |
| Akan | 5 (6.0) | 12 (17.9) | 8 (12.9) | 11 (20.4) |
| Ga/Adangbe | 4 (4.8) | 2 (3.0) | 1 (1.6) | 0 |
| Others | 8 (9.5) | 7 (10.5) | 6 (9.7) | 6 (11.1) |
| **Level of education** |  |  |  |  |
| None | 1 (1.2) | 3 (4.6) | 1 (1.6) | 2 (3.7) |
| Arabic education | 3 (3.5) | 5 (7.6) | 2 (3.2) | 2 (3.7) |
| Primary school | 1 (1.2) | 6 (9.1) | 9 (14.5) | 5 (9.3) |
| Secondary school | 11 (12.9) | 25 (37.9) | 20 (32.3) | 26(48.2) |
| Degree/Equivalence | 31 (36.5) | 26 (39.4) | 21 (33.9) | 16 (29.6) |
| Postgraduate | 38 (44.7) | 1 (1.5) | 9 (14.5) | 3 (5.6) |
| **Household average income (USD)** |  |  |  |  |
| 0-100 | 11 (13.3) | 11 (17.5) | 28 (45.2) | 23 (44.2) |
| 101-250 | 20 (24.1) | 37 (58.7) | 23 (37.1) | 21 (40.4) |
| 251-500 | 31 (37.4) | 11 (17.5) | 7 (11.3) | 5 (9.6) |
| 501-1500 | 8 (9.6) | 4 (6.4) | 4 (6.5) | 1 (1.9) |
| 1501-3000 | 9 (10.8) | 0(0) | 0(0) | 2 (3.9) |
| >3000 | 4 (4.8) | 0(0) | 0(0) | 0(0) |
| **Location** |  |  |  |  |
| Urban | 84 (98.8) | 62 (95.4) | 59 (93.7) | 42 (77.8) |
| Rural | 1 (1.2) | 3 (4.6) | 4 (6.4) | 12 (22.2) |

**CAB FGD**

| **n=85** | **Frequency n(%)** |
| --- | --- |
| **Site** |  |
| Abeokuta | 7 (8.2) |
| Accra | 8 (9.4) |
| Ibadan | 22 (25.9) |
| Kano | 16 (18.8) |
| Kumasi | 9 (10.6) |
| Ilorin | 17 (20.0) |
| Zaria | 6 (7.1) |
| **Mean age(SD)** | 51.0 ($\pm$11.4) |
| **Age group (years)** |  |
| <=40 | 14 (16.5) |
| 41-50 | 33 (38.8) |
| 51-60 | 23 (27.1) |
| 61-70 | 11 (12.9) |
| 71-80 | 3 (3.5) |
| 80+ | 1 (1.2) |
| **Gender** |  |
| Male | 66 (77.7) |
| Female | 19 (22.4) |
| **Marital Status** |  |
| Single | 3 (3.5) |
| Married/living with someone | 82 (96.5) |
| Widowed |  |
| **Ethnicity** |  |
| Yoruba | 42 (50.0) |
| Igbo | 2 (2.4) |
| Hausa | 23 (27.4) |
| Akan | 5 (6.0) |
| Ga/Adangbe | 4 (4.8) |
| Others | 8 (9.5) |
| **Level of education** |  |
| None | 1 (1.2) |
| Arabic education | 3 (3.5) |
| Primary school | 1 (1.2) |
| Senior secondary school | 11 (12.9) |
| Degree/Equivalence | 31 (36.5) |
| Postgraduate | 38 (44.7) |
| **Household average income (USD)** |  |
| 0-100 | 11 (13.3) |
| 101-250 | 20 (24.1) |
| 251-500 | 31 (37.4) |
| 501-1500 | 8 (9.6) |
| 1501-3000 | 9 (10.8) |
| >3000 | 4 (4.8) |
| **Location** |  |
| Urban | 84 (98.8) |
| Rural | 1 (1.2) |

**Caregiver FGD**

| **n=67** | **Frequency n(%)** |
| --- | --- |
| **Site** |  |
| Abeokuta | 7 (10.5) |
| Accra | 7 (10.5) |
| Ibadan | 6 (9.0) |
| Kano | 16 (23.9) |
| Kumasi | 8 (11.9) |
| Ilorin | 17 (25.4) |
| Zaria | 6 (9.0) |
| **Mean age(SD)** | 41.3 ($\pm$13.1) |
| **Age group (years)** |  |
| <=40 | 34 (50.8) |
| 41-50 | 19 (28.4) |
| 51-60 | 11 (16.4) |
| 61-70 | 0 |
| 71-80 | 3 (4.5) |
| 80+ | 0 |
| **Gender** |  |
| Male | 30 (44.8) |
| Female | 37 (35.2) |
| **Marital Status** |  |
| Single | 13 (19.4) |
| Married/living with someone | 53 (79.1) |
| Widowed | 1 (1.5) |
| **Ethnicity** |  |
| Yoruba | 29 (43.3) |
| Igbo | 1 (1.5) |
| Hausa | 16 (23.9) |
| Akan | 12 (17.9) |
| Ga/Adangbe | 2 (3.0) |
| Others | 7 (10.5) |
| **Level of education** |  |
| None | 3 (4.6) |
| Arabic education | 5 (7.6) |
| Primary school | 6 (9.1) |
| Senior secondary school | 25 (37.9) |
| Degree/Equivalence | 26 (39.4) |
| Postgraduate | 1 (1.5) |
| **Household average income (USD)** |  |
| 0-100 | 11 (17.5) |
| 101-250 | 37 (58.7) |
| 251-500 | 11 (17.5) |
| 501-1500 | 4 (6.4) |
| **Location** |  |
| Urban | 62 (95.4) |
| Rural | 3 (4.6) |

**Stroke Free FGD**

| **n=63** | **Frequency n(%)** |
| --- | --- |
| **Site** |  |
| Abeokuta | 6 (9.5) |
| Accra | 7 (11.1) |
| Ibadan | 7 (11.1) |
| Kano | 8 (12.7) |
| Kumasi | 14 (22.2) |
| Ilorin | 13 (20.6) |
| Zaria | 8 (12.7) |
| **Mean age (SD)** | 42.8 ($\pm$9.8) |
| **Age group (years)** |  |
| <=40 | 25 (39.7) |
| 41-50 | 25 (39.7) |
| 51-60 | 10 (15.9) |
| 61-70 | 3 (4.8 |
| 71-80 | 0 |
| 80+ | 0 |
| **Gender** |  |
| Male | 40 (63.5) |
| Female | 23 (36.5) |
| **Marital Status** |  |
| Single | 14 (22.2) |
| Married/living with someone | 46 (73.0) |
| Widowed | 3 (4.8) |
| **Ethnicity** |  |
| Yoruba | 28 (45.2) |
| Igbo | 2 (3.2) |
| Hausa | 17 (27.4) |
| Akan | 8 (12.9) |
| Ga/Adangbe | 1 (1.6) |
| Others | 6 (9.7) |
| **Level of education** |  |
| None | 1 (1.6) |
| Arabic/Non-formal education | 2 (3.2) |
| Primary school | 9 (14.5) |
| Junior secondary school | 8 (12.9) |
| Senior secondary school | 12 (19.4) |
| Degree/Equivalence | 21 (33.9) |
| Postgraduate | 9 (14.5) |
| **Household average income (USD)** |  |
| 0-100 | 28 (45.2) |
| 101-250 | 23 (37.1) |
| 251-500 | 7 (11.3) |
| 501-1500 | 4 (6.5) |
| **Location** |  |
| Urban | 59 (93.7) |
| Rural | 4 (6.4) |

**KII**

| **n=99** | **Frequency n(%)** |
| --- | --- |
| **Average years (SD) working in current position** | 13.6 ($\pm$10.9) |
| **Number of years working in current position** |  |
| <=5 | 26 (26.8) |
| 6-10 | 22 (22.7) |
| 11-15 | 17 (17.5) |
| >15 | 32 (33.0) |
| **Mean age(SD)** | 48.6 ($\pm$10.3) |
| **Age group (years)** |  |
| <=40 | 20 (20.2) |
| 41-50 | 40 (40.4) |
| 51-60 | 29 (29.3) |
| 61-70 | 9 (9.1) |
| 71-80 | 0 |
| 80+ | 1 (1.0) |
| **Gender** |  |
| Male | 71 (71.7) |
| Female | 28 (28.3) |
| **Marital Status** |  |
| Single | 3 (3.0) |
| Married/living with someone | 95 (96.0) |
| Widowed | 1 (1.0) |
| **Ethnicity** |  |
| Yoruba | 45 (45.5) |
| Igbo | 6 (6.1) |
| Hausa | 22 (22.2) |
| Akan | 13 (13.1) |
| Ga/Adangbe | 4 (4.0) |
| Others | 9 (9.1) |
| **Level of education** |  |
| Arabic/Non-formal education | 8 (8.1) |
| Primary school | 2 (2.0) |
| Junior secondary school | 1 (1.0) |
| Senior secondary school | 2 (2.0) |
| Degree/Equivalence | 31 (31.3) |
| Postgraduate | 55 (55.6) |
| **Household average income (USD)** |  |
| 0-100 | 4 (4.2) |
| 101-250 | 15 (15.6) |
| 251-500 | 21 (21.9) |
| 501-1500 | 29 (30.2) |
| 1501-3000 | 19 (19.8) |
| >3000 | 8 (8.3) |
| **Location** |  |
| Urban | 96 (97.0) |
| Rural | 3 (3.0) |

**SIREN Stroke FGD**

| **n=54** | **Frequency n(%)** |
| --- | --- |
| **Mean age(SD)** | 54.5 ($\pm$12.6) |
| **Age group (years)** |  |
| <=40 | 7 (13.2) |
| 41-50 | 13 (24.5) |
| 51-60 | 15 (28.3) |
| 61-70 | 14 (26.4) |
| 71-80 | 4 (7.6) |
| 80+ | 0 |
| **Gender** |  |
| Male | 37 (68.5) |
| Female | 17 (31.5) |
| **Marital Status** |  |
| Single | 4 (7.6) |
| Married/living with someone | 42 (79.3) |
| Divorced/Widowed | 7 (12 (13.1) |
| **Ethnicity** |  |
| Yoruba | 24 (44.4) |
| Igbo | 3 (5.6) |
| Hausa | 10 (18.5) |
| Akan | 11 (20.4) |
| Ga/Adangbe | 0 |
| Others | 6 (11.1) |
| **Level of education** |  |
| None | 2 (3.7) |
| Arabic education | 2 (3.7) |
| Primary school | 5 (9.3) |
| Junior secondary school | 7 (13.0) |
| Senior secondary school | 19 (35.2) |
| Degree/Equivalence | 16 (29.6) |
| Postgraduate | 3 (5.6) |
| **Household average income (USD)** |  |
| 0-100 | 23 (44.2) |
| 101-250 | 21 (40.4) |
| 251-500 | 5 (9.6) |
| 501-1500 | 1 (1.9) |
| 1501-3000 | 2 (3.9) |
| **Location** |  |
| Urban | 42 (77.8) |
| Rural | 12 (22.2) |
