# Supplementary material for: Research participants’ perception of ethical issues in stroke genomics and neurobiobanking research in Africa
Source: PLoS One. 2025 May 6;20(5):e0292906. doi: 10.1371/journal.pone.0292906 (PMC12054916; doi:10.1371/journal.pone.0292906)
Supplement: S3 File — (ZIP) [file pone.0292906.s003.zip › Files for PLOS ONE - updated March 2025/Accra_SIREN Stroke Caregivers_ FGD.docx]

**African Neurobiobank for Precision Stroke Medicine - Ethical, Legal, and Social Implications (ELSI) Project:**

Completed Transcript- FGD

Site: Accra

Designation: SIREN caregivers FGD

Interviewer: Nathaniel Coleman

Note Taker: Gertrude

**Key note:**

I means interviewer

R means responses.

Findings:

Good morning all and thank you for making time for our discussion today on the ELSI project. As said earlier we are here to learn and share your views and there are no right and wrong answers and feel free to talk when called upon.

I What do you know about genetic research and have you heard anything at all about genetic research? Do you have any experiences?

R1 Briefly, your DNA and what comprise of your body cells and your blood cells and things that goes with your body. So the blood groups and other things.

R2 I have heard about it and what I know about genetic research is that what really happens concerning health issues in the family.

R3 genetic research helps us to know something about the family life and it helps us to know how to live our life.

R4 Its mostly what everyone has said and its about the generic composition of what is made up of someone and how the total composition affect the health of the individual and which runs in the family and its hereditary.

I Have you participated in any genetic research?

R1 No

R2 No

R3 No

R4 yes

R5 No

R6 Yes.

I Can you share any experience?

R1 Yes I participated in SIREN and we finding out the causes of stroke in blacks to find out if it is hereditary or not.

R2 I also participated in the SIREN myself and through that I realized that what has happened in my family I have to be very careful with myself.

I What are the benefits of genetic research and if so what are the benefits in medicine in general and stroke specifically?

R1 I think if we know more about this genetic research then you can educate your family so well so that nobody will fall into anything.

R2 If you are aware it helps to take care of yourself and how to go about your daily matters, your cleanliness, hygiene and intake of what you need to and what you don’t have to. Secondly, the research will help other health workers because it is more specialized and in case other people go there to another health center they will be up to date and be able to care for the people and others will also know how to care for someone if they should come to that place and suffering from any form of stroke.

R3 Is it in relation to the patient or what [Interviewer interjects….yes to the patient] ok. In my case it is helping me to live right and to know what to take, what to do and what not to do and I will stand in the position to give advice to someone if I find out that what the person is doing is not healthy.

R4 It gives you the information that changes your thinking on how you do things and how to prevent getting the stroke.

R5 When we do the genetic research it will help in clinical care because when you are sick and admitted at the hospital, the doctor is able to check your family background to know if something has happened and it will help with the treatment.

R6 To add to my sister, sometimes we practice self-medication without going to the hospital for the doctor to examine you well and we use self-medication that causes most of our problems a lot and even causing of stroke so the research will help a lot.

R7 I also think that some of the problems are not from what we eat only but also the stroke that happens occur from what we do at our work sites. We don’t have enough time for rest and you will be working all your years and these could cause stroke as well.

R8 Also when there is research and people know what to do, there is long life, good health and its for the future generations. Outside Ghana and Africa, people know about all these and they know how to take care of themselves and they live up to 80 and 90 years instead of people dying at 60 and below so if we know about these research it will help us to live long.

I Thank you and we are moving on with the discussion and we will be hearing some terms which you could try to explain whether you know about them or not. If you don’t know too that’s fine, I will explain for you all. So what do you know about bio banking?

R1 I have not heard about it.

R2 I have not hear about it also.

R3 I have not heard about it but I am trying to get that from the word, bio thus the study of plant or human nature and the bank is a place where we save or put things for storage and therefore collecting all these health tissues to the bio bank where we store them for later use or whatever. So then the research is done it could be written in a form of scientific paper. And that is what I know and anything concerning bio is health issues.

R4 Is like getting some information and store and it could be used when needed.

I So you are all right and bio means biological and banking is the place where we store money so biobanking is a place where we store human samples like blood, tissues samples for research. So now that we know about biobanking, how beneficial do you think it will be and how should it be operated?

R1 If we have something like that in place anytime we need the sample we can go there and take it. Than if there is an emergency and you now have to go looking for a donor and that might not be readily available but if you have such a system in place then you can go there and get it and use.

I So how will it benefit us in terms of medicine?

R1 With this bio banking, who goes there to get the information? is it the patient or the … [Interviewer explains that it’s the researchers and its samples to be used for research studies and findings from the research will be used to improve on care for the patients]

I So now that you have an idea of bio banking, how should it be operated? Should there be a law guiding bio banking?

R1 Yes it can be enforced because if you want anything to come on right there should be some policy that everybody should follow and I think that it helps a lot.

R2 Everything there is in order so you cannot go and take samples when you are not entitled to so even when you are collecting the data there is privacy and you will not mention the person’s name. You need to respect the clients and their privacy and although there is right to information, you should set limits. There are specific people samples and they are bound by rules and regulations so that you don’t misuse people’s data and you supply it at places where you shouldn’t have.

R3 I agree with them because when you say bio bank, there are a lot of customers who operate in the bank and you cannot take the customer’s information like his balance and let everyone know. There are things that needs to be kept secret. So if you say bio banking then there should be a law enforcing certain policies to it otherwise the information you collect will not be secret.

R4 There must be a law in place so that nobody can use someone’s data out and so there must be a law.

R5 I have a question since if you put your money at the bank you get interest, so when you put in all these information about clients health issues, what benefits do they get. [Interviewer explains that yes the banks gives interest on the money that is kept at the bank and so the findings from this study will benefit the whole community]

I So you all said there should be a law guiding bio banking in Ghana. But I want to know is there a law currently guarding biobanking?

R I have not heard of any.

R I don’t know of the law

R I haven’t also heard of any law but maybe if I should use the blood bank as an example, I think people go there and they donate their blood there. And the blood is tested and if there is any problem with it nobody hears of it and it is you alone they talk to.

R I have stayed outside for a while and anytime you go to the hospital there is this terms and agreement that we don’t have the time to read so I am sure maybe the laws will be there but the public may not be aware and the clients may not be aware and for we the black man we don’t like reading.

I Another term is precision medicine and what have you heard about it? Can you get the meaning from the two words?

R1 I don’t want to talk much but by the English word precision meaning something that you are doing and you want to be precise so being precise in the medicine and giving of remedies and which is direct and holistic and that is what I will say about precision medicine.

R2 No idea

R3 I have no idea

R4 No idea

R5 So giving the right treatment with the right medicine.

I So you are all right and precision medicine is targeted and right treatment and medicine given to individual and not the general medicine for all. So like you have malaria and we all take ACTs but this one is targeted and we look at your genetic system and give you the right medicine that is going to work for you and we do that for the next person by checking the genes and giving you the right medicines as well. So we are not going to give the general treatment but a targeted treatment per your genes.

Ok. So then how important will this precision medicine be to us in Africa?

R1 It will be great.

R2 It will help to reduce the guess work and rather focusing on giving the right and targeted treatment to avoid giving the general treatment and anyone who comes to the hospital with signs of malaria are given malaria treatment because they believe mosquitoes are around. So this will help to prevent the guess work.

R3 So then you have to focus on the medicine or the prescription given you and not to mix the medicine.

R4 I think most of us what we usually do it that when I have headache and I come to the hospital, and I am given a particular drug and my sister tells me that she has headache, I will say go and buy this drug because they gave it to me and I was ok so you too go and buy and you will be ok. But then if I understand what you are saying then what we have been doing is something wrong because maybe we are twins but then we have everything different all together. So we shouldn’t practice that.

R5 I also think that it will help us a lot because if Doctor prescribes something and you go taking another thing it will bring lots of problems. So if they could obey the rules of what the doctor has prescribed to you it can make things easier for us.

R6 Then it means that with the precision medicine when someone is sick and the persons goes to the hospital then the doctor or health personnel should try and know the problem with the person before prescribing the medicine if that is my understanding. Then I think with this one if we all agree it will help solve a lot of problems. Because at times we the patients when we have headache then it is malaria and we go and buy the drugs. At times too when you are sick and you go to the hospital, some of the health personnel things that the common sickness is malaria and everyone is complaining and they keep giving everybody the same drug. You go to a pharmacy and because they have the drugs that they are selling, when you tell them your problem they tell you that they have this so you should take it and you will be well. But if you implement this kind of precision medicine, before the doctor or nurse prescribes medicine to the patient, the person should be aware of the person’s genetic conditions before prescribing the drugs and it will solve a lot of problems for us.

R I have an experience on what we are discussing here. The time that I brought my wife here, the medicine given to us we sent it home but later the whole thing turned into something else and we came back here and we were told to go a different place. So we went to the different hospital and they saw the medicine and they said no they are not going to apply this medicine and they then changed the medicine and I don’t know if because of that it created a problem for me. So we have to look at that one too.

R To add to that I remember a day before my husband got sick, he said he wasn’t feeling well and he went to the pharmacy and told them that he wasn’t feeling well. He takes his BP medicines every day and that day the pharmacist gave him some drugs that he said it contains three different drugs together for BP. He took it and in the evening we had to rush him to Airport clinic and when we got there, the doctor asked the kind of drugs that he had taken and when we showed it to him and after doing a series of test the doctor wanted us to give him the name of the pharmacist and sue him and I asked why and he said the drug that the pharmacist gave my husband was so high that it then his sugar level was high too and it stimulated the effect. But my husband said he wasn’t feeling well and went for health care so he will not go ahead with any suing issue and that he needed care at that moment. So some of these things if everybody thus doctors, nurses know that if someone comes to you for health care and I am to sell and care for this person I have to do certain things before then it will solve a lot of problems.

R so the best way is for the person to go to the laboratory for testing and find out what is actually happening to the person.

I Alright thank you. So can we apply the precision medicine to stroke?

R yes [Echoed by all respondents]

I Like how?

R1 Like I said earlier when my husband took that medicine, and after a day or two he had the stroke so if the health personnel could have examined him well he could have prevented the stroke.

R2 One time a friend of mine was sick and she went to the hospital and she was given a drip and whiles taking the drip she collapse and later a senior doctor came and said the women had sugar in her blood already and the woman said that the drip that they gave her had sugar and so the doctor could have asked the woman whether she is diabetic before applying that particular drip. So in fact she went through a lot of problems before recovering.

R3 I want to ask a question but I know it should be at the end of the discussion. How well is this research with all this data that we are collating them and gathering together? Is it going to end up at the required end users point and I mean the patients and the clients and the required medical personnel. That is my question now and as for the data we collect them every day and we have good data and how well is it going to end up for the people who will use it. So that it will be applied well. [So the response will be given at the end of the discussion]

R We the patients sometimes when we are sick and we are in a hurry to get well like the market women and others on the street, instead of going to the hospital they rather buy the medicine at the pharmacy and take. But one thing that I have decided to do is that when I am sick or feeling hot, I will rather go to the pharmacy to get tested to be sure I have malaria before taking the medicine. But some people will not do that and in a hurry to do business and just go and buy paracetamol and take. So if they don’t meet someone who is educated and who will ask questions before giving the drugs out then they will go and buy malaria drugs and take. So my advice is that if you are not feeling well, you need to get tested to know the exact problem before you are given drugs to take.

I We move on to our next term that is brain donation. So what do you understand by brain donation for research purposes.
R My understanding is about how we think about something and bring it out.

I ok but what I mean here is we donating our brains to be used for research and learning purposes.

R So when someone dies and the person decides to donate the brain to be used for studies because stroke affects the brain of people and to make legal documents to enable the brain used for research and used to teach students at the medical school.

I Are you willing to donate your brain when you die for research purposes and why?

R1 To me I can donate my brain for the future generations.

R2 Yes I can donate my brain and when I am dead where am I taking that thing to when others can learn from it.

R3 No I will not donate my brain.

R4 personally I need to think about it but I think first the idea should be sold to the community so that we can start gradually and maybe I might not be terminal ill and someone might be so its broad and I cannot just say I will donate the brain or not. But for now I need to assimilate it.

R3 No, I will not be able to donate my brains for research.

I So now what are the reasons why people will not able to donate their brains for research purposes? What cultural, social, religious factors that will inhibit people from donating brain and body parts?

R1 Whether you are Christians or atheist, its our cultural belief that will prevent us and our culture gives us the know-how and understanding. First it has to be sold to the community and we have lots of superstitions and our way of thinking is different.

R2 I want to know if it is done elsewhere [other respondent responded yes] and is it beneficial [Interviewer responds Yes].

R3 Sometimes it’s the family and they will not understand why you want to donate your brains or your heart to somebody or for research and its very difficult for the family to understand unless you yourself and the one going to donate when I die that you have to explain to the family. So for example if I die and I said that within 2 to 3 days I should be buried and the family has to understand that because that is me and we have individual differences. So that is what I can contribute to that particular issue.

R I think it depends on the individual but if the individual does not agree, the family also do not agree because during postmortem some families do not agree to it and they will say that they don’t want to hear anything. This is individual decision but I am sure that after so much education people might change.

I So are there any religious beliefs or practices that can prevent people from donating their brains?

R1 yes due to religious beliefs some people will not donate their brains. For example Christians and I mean a few of them will understand and the moslem too a few of them they will understand. But for the traditional people I don’t think that they will understand so because they also have different beliefs and Christians too have different beliefs. For traditionalist I will say no.

R2 Its like a ghost without a brain [Laughter by all respondents]

R3 Also in the moslem community when someone dies then within few hours he is buried and even when the person dies at the hospital they will not even wait for the doctor to sign on him to be sent to the mortuary for Postmortem but they will quickly take the person home and bury the person. So in such cases it will be difficult to get the brain of the person for research.

I So we have outlined some of the reasons why people will not be able to donate their brains for research purposes. Now can we discuss things that could promote people donating their brains for research purposes?

R3 still it about education because if you do not understand you will not donate your brain.

R its all on education to the communities.

I The discussion is getting more interesting and I want to know what are your thoughts on blood donated for research purposes? I mentioned earlier on donating of brain for research purpose but now we are on donating of blood sample for research purposes. Are you willing to do that?

R1 I am ready to donate my blood for research.

R2 People could give their blood for research purposes but it needs a lot of education and in our culture as Ghanaians, the superstition is high and people are killing others for blood for rituals so people are scared about doing these things but real people will understand and do that. But the larger community where the sickness are and when the areas are not clean, and the hygienic condition is very bad then that is where we need to educate them the more about that when there is lots of blood donated then it should be studied and interventions developed to address the problem we face.

R Depending on who is taking the blood and where and if it is the health personnel who are taking it and its explained well to the people then everyone will be willing to participate because of this and that reasons. But depending on the area and the people involved then it will not be much of a problem.

R So its all about education and people don’t trust the health workers till date so you see we need a lot of education before they can understand exactly what they need that blood to do. So its all about education.

I But are we all willing to donate little blood for genetic research purposes?

R yes I will.

R You know some of us we understand what they are going to use our blood for but the larger community maybe we have different kinds of diseases in our blood and I will be suffering from different thing and others as well. But the problem is with those out there and they need the education.

R I think education is needed and if someone is sick from a community, and you realize that this person has this sickness, the health personal can do something about it. I remember when my husband was admitted here for treatment, we heard about this SIREN project and we were called and I was educated on what they were going to do and I was happy. So when you are aware it is good and I remember pressing on brother to do it for us. So if someone is sick and you realize that this sickness is something that we are having at the hospital, you have to go to the community, talk to them and let them know that this is what you have found and you want them to do sample test to know how they can be helped and I think that they will agree.

R Yes I am ready to donate my blood sample to be used for genetic research because you can’t educate the public without having an idea of what you are doing. So definitely you must have fair idea and take samples and to know what are the risk factors, what are the related diseases before going to the public and then educate them so for me I will say yes.

I So we are all willing to donate our blood sample for genetic research in stroke?

R All participants responded yes.

I Apart from the education that will inform people the need to donate their blood for research purposes, what will still be the barriers preventing them from donating their blood sample?

R Yes due to the issue of sakawa and cultural related issues with blood use.

R2 Some religions also do not support the donation of blood like the Jehovah witness.

R3 The barrier is not about outside but the one who took the blood to do the research. There are some people when they see something about you, instead of finding a way to tell you they will not and the way they will say it, it will even cause fear and panic to yourself. Sometimes the way some of the nurses gives out the information of the findings its not the best and when they give you the results you might even be frightened. They should rather release the results in a nice way. Because of these things, most people don’t want to go to the hospital.

I Do you think that your family members and community members be willing to donate their blood for genetic research in stroke?

R yes they will

R Yes by educating them so it is still on education

R2 Yes they will like to donate their blood sample but we need to educate them on that.

I ok so now lets move onto another term and that is Informed Consent. What so you understand by informed consent?

R1 Taking permission from someone before participating in a study.

R2 Hmmm, let me try something and see. To inform the person that this is what I am going to use your blood for so that he will know that my blood is going to be used for certain things like the research so that is what I can say.

R3 There should be an agreement to use your sample.

R4 You should be on the known for whatever you are going to do.

I So there are types of consents and I am going to explain the types and you can tell me the types that you will prefer if you are to give your samples out for research. So we have the broad consent or generic meaning I am giving you my sample and you can use it for any research you want to do. Then there is the restricted consent meaning I have given you my blood to be used for only stroke and you cannot use it for anything else. Then there is the tiered consent which says that you can use my sample for stroke and malaria and not for hypertension. So you can choose which ones your sample can be used for. Then there is the dynamic consent which is online based and the researcher could contact you to use your sample for any study they want to do and then you give your consent n that. So you are always in contact and its online based. So the four types of consent that I have explained thus Generic/Broad, Restricted, Tiered and Dynamic consent, which one will you prefer and why?

R The restrictive

R Dynamic so that I get to know what my sample is being used for.

R I will prefer dynamic consent.

R I will go for dynamic

R I will go for dynamic.

R I will also go in for the dynamic consent.

I Why is everyone going for the dynamic consent. Any reason please?

R I will go for dynamic because when you come to me and say I want to use your sample for stroke research so its between you and I. So its purposely for stroke and nothing else and that is why I want the dynamic. But the question now is how do I know if my sample is used for something else though I didn’t choose that.

I from your submission and from my explanation then I think its the restricted type of consent that you have selected because you said its only stroke that you want your sample to be used for but for the dynamic it is online and anytime I want to use your sample for any research I contact you for approval before using it. But in this I don’t take your blood any more but use what I have already collected.

R I also chose dynamic because since my blood will not be taken again and the researcher has my old blood then he needs to contact me when he wants to use the blood again for another thing else. If I chose the restricted then my sample could not be used for anything else apart from stroke but once I give the dynamic it could still be both generic and restrictive as the researcher will contact me for my approval before my blood sample is used again. So that I will not be pricked twice. So the same blood can be used for other tests and research.

R I chose dynamic because in case of anything concerning the research I will be in the known and it will also help me to enhance my way of living. Because if you come with hepatitis test I will say ok I haven’t done that so ok and I will always be in the known with inflows and outflows and that is why I prefer the dynamic. So if I choose dynamic is that how it will go with me.

I So yes that is how we will go about with the consent given. So if you give us your blood sample for stroke study and later on we want to look at HIV, we cannot use your sample without contacting you for your approval. So then we will contact you that we are having your sample and want to do other study on HIV so can we use your blood. So if you agree before we can go ahead. But for the restricted consent, we cannot contact you again and even in the case of your death that we cannot contact, we may have to contact your next of kin if we have to relay results to you.

R So if you take my blood today ad there is no infection after some years, will there be infection in my blood. [Interviewer said all questions will be answered at the end of the discussion.]

R I don’t think there is any issue storing blood for future use because it will help with researching on our health.

R I also support blood being used for future use.

R I want to know the life span of the blood that you are storing and if you take my sample like a year ago and after 2 years you come again for my sample saying you tested for stroke and hepatitis and then you contact me that you need to run another test again with my blood, I want to know whether it will be the same blood that you have at the bank. [All questions will be answered at the end of the discussion]

I So what is your view on data and sample sharing with other partners and collaborators in other country like I said the study is being done in Nigeria and we have partners in USA and UK as well. What is your view of sharing sample with the partners?

R1 It’s a good thing

R2 It’s a good idea.

R3 It is very good so that you also know the health status over there.

R4 When you share data it will be gathered and it will serve as a standard for other health officials to know how to go about it when someone has the disease and then you are able to care for people well.

R5 It will add up to the body of knowledge.

I So in the process whereby incidental findings are gathered during the research and telling you that results will help you, how do you want the findings communicated to you? Do you want it by face to face, by phone or by email?

R It should be done one-on-one and you should know how not to frighten the person with the results.

R I think it should be done one-on-one and you might have some drugs to give to the person.

R It should be by one-on-one or face to face.

R It can also be given by email.

R So how much control should an individual have on their sample given out for research. Should it be 100%

R I think it should be 100%

R I am counting on the trust and integrity of the medical personnel and the research fellows and there are guidelines so wherever there are the samples in the freezer, then it should be under secure storage. When I come to the hospital and I am taken care off and my privacy is kept and my folder kept well, I also think my sample should follow the same routine.

R I have 100% control because it is my sample.

R I have full control over my sample.

I How should biobanking be operated? Should there be a regulatory body to control affairs of it?

R All respondents said Yes there should be a regulatory body.

R1 So that they could have control over your samples and not to be misused.

R2 And if you go contrary to that then I can sue you.

R3 Yes there should be a regulatory body that will make it legally bound.

I What suggestions do you have that can create awareness and improve on sample donation thus brain and blood sample donation for genetic research in stroke?

R After taking the sample if it is a stroke then the researcher has to inform us about the findings so that we can go out and create awareness for the public and inform them that we did this research and this is what we found so that they can be educated on how to go about their daily activities.

R Set a table at the hospital like an information desk and when people come to the hospital then you sell the idea to them.

R There should be more education and when you come to the hospital and waiting to see the doctor, there should be someone who will come and talk to you about what is going on and once we are educated then we can also go out and tell others about it.

R We can use the media to create awareness on sample donation for research.

R There should be awareness creation at schools, churches so people know more about genetic research and get them involved.

I Alright thank you very much. Are there any recommendations or concerns on using these body tissues for research in Ghana which you will want to talk about but we haven’t mentioned it so far?

R It is good that this research is coming on and when everything is done the findings should be shared with the medical team. Because we came to the stroke unit and after everything we had an emergency and we were referred to the 37 hospital but then handling there wasn’t good at all and unsatisfactory and when you tell them something to do they ask whether you are a doctor. So when information is shared, it is good and knowledge is acquired over board so that is what I want to say.

R Like I said earlier most of the causes of diseases is as a result of the institutions that we work and the work place condition. I see that people get stressed and we don’t have rest and there are no off days and I have to call my boss that I am sick then I could rest. So if the information could go to the private sectors so that they can give the workers off days to rest and it will help us a lot.

R I think the way you have called us and collected our views, I think the same things should be done to the health personnel maybe not people at the stroke unit but others at other health centers and seek their views then when you are giving education on this blood and brain donation, I think you should be vice versa.

R I want to suggest that this awareness creation should start from schools and now they are coming so they have to know about all these and we can start with the education from JHS, SHS, Tertiary schools and I think that will also help.

I I will like to say a big thank you to you all and its been a great discussion and I am sure you have learnt something from it.
