# Supplementary material for: Research participants’ perception of ethical issues in stroke genomics and neurobiobanking research in Africa
Source: PLoS One. 2025 May 6;20(5):e0292906. doi: 10.1371/journal.pone.0292906 (PMC12054916; doi:10.1371/journal.pone.0292906)
Supplement: S3 File — (ZIP) [file pone.0292906.s003.zip › Files for PLOS ONE - updated March 2025/Accra_SIREN Stroke Cases_ FGD.docx]

**African Neurobiobank for Precision Stroke Medicine - Ethical, Legal, and Social Implications (ELSI) Project:**

Completed Transcript- FGD

Site: Accra

Designation: SIREN CASES FGD

Interviewer: Nathaniel Coleman

Note Taker: Gertrude

| **Items** | **Participant 1** | **Participant 2** | **Participant 3** | **Participant 4** | **Participant 5** | **Participant 6** |
| --- | --- | --- | --- | --- | --- | --- |
| Age | 60 | 43 | 60 | 41 | 61 | 46 |
| Sex | Male | Male | Male | Male | Female | Female |
| Marital status | Married | Single | Married | Married | Married | Married |
| Ethnic group | Akan | Ewe | Akan | Akan | Buem | Akan |
| Educational level | First Degree | Senior Secondary | Postgraduate | Senior secondary | Senior Secondary | Senior Secondary |
| How long had stroke | 3 years | 1 month | 3 years, 4 months | 6 years | 3 years | 2 years |
| Use of internet | Yes, for study | Yes, for reading news | Yes, for official correspondence and social media | Yes,  Education | No | No |
| Monthly income range | 501-1500 | 0-100 | 501-1500 | 101-250 | 0-100 | 101-250 |
| Location | Urban | Urban | Rural | Urban | Urban | Urban |

**Key note:**

I means interviewer

R means responses.

Findings:

Good morning all and thank you for making time for our discussion today on the ELSI project. As said earlier we are here to learn and share your views and there are no right and wrong answers and feel free to talk when called upon.

I What do you know about genetic research and have you heard anything at all about genetic research? Do you have any experiences?

R1 I have heard about it but I don’t know what it is.

R2 I have also heard about it but I don’t know how genetic research is done.

R3 Genetically we are all different people so they take the blood sample of me, you and they do a research of it. And that is to show whether he is a Chinese, he is Gambian and the likes.

R4 I know it’s a human research about our different genes.

R5 I have heard it before and it helps us to know our systems and they check to know whether I have BP or those diseases.

I Have you participated in any genetic research?

R1 I don’t

R2 I have just read about it but for somebody participating in it, no.

R3 I have heard about it on social media and on the news but not participated in any.

R4 No

R5 No

R6 No.

I Can we apply genetic research in stroke?

R Yes and when you apply it in Stroke, you ask yourself how old was this man before he had the stroke or how young was this man before he has the stroke. If he is getting nearer you can tell and that’s my view.

R2 For instance my dad had a stroke and my big brother has a mild stroke and my dad died of stroke and he had the attack on Sunday and died a month after. And my big brother had a mild stroke and he survived it and he had it around 40 years but passed on at 60 years no sorry at 58 years. When he had the stroke he had his mouth turned and he didn’t recover from that and when he speaks it was not clear. So I was very careful of myself but the day before I had the stroke, I felt some sort of heaviness within me, and my cousin was going to have an engagement the following day so I was helping with the arrangement. As we were going, I felt some heaviness within in and I was afraid and I was wondering what was going to happen to me. So as we were moving it came to a time I said I wanted to relax and I relaxed that for a while and when we went home I still felt the heaviness and the next day I had the stroke. So I have been advising my sisters that anytime they feel the heaviness they should be careful.

I So from your submission it is good to have genetic research to know what runs in the family to see how to improve upon it. Then there is so much benefit in doing genetic research even in medicine?

R1 A lot. There are lot and in my case, I didn’t see any sign but about 2 weeks before the time, there was a blood clot on my eye and not knowing it was a sign to that so I came here to the eye center and I was asked to do some test. I did it and by the time I went for it, it had happened. Apart from that there was nothing and I was normal. So I think that the research has to be done and to let everyone know so that if by chance anything is happening to your body, you will know.

R2 In my case I have four siblings and they all had stroke, my mother had one and myself. I had the attack on 2013 and it came back in 2017 although I wasn’t 100% fit thus 70% recovered and it came back in 2017 till now. You see so the research will really help.

R3 I got to know that the experiences you have or the suffering you had when you were growing up could also contribute to this. When we were growing up, we were maltreated so there was this fears that developed in us and also through the challenges of labor. I am the type that I fear things a lot due to the maltreatment I was subjected to when I was growing up. And I also didn’t get any support during labour and childbirth and I had to do everything all by myself. When I gave birth to my last born, I had no one to help me and my BP was high and I was operated on. In all these challenges I didn’t have anyone to assist me and I went through it alone and when I gave birth to my last child, he had something on his face and had to be sent to NICU (Neonatal Intensive Care Unit) so I had to care for the child at the NICU. Because of that I couldn’t go for checkup because my BP was high and I realized that most people with high BP were detained at the hospital and when I heard that my child was admitted at NICU and my BP was high and I had to see the doctor, I run away from the hospital. So then I realized that all those activities that I went through contributed to the stroke. But now I have the confidence and I am not afraid of things again.

In addition I learnt that if there is history of stroke in your family, then you are at risk of also getting infected with stroke. I have seen people in my family that suffered from stroke and even my father had stroke but he got healed early and he lived till over 80 years before he died. In my case, depression set in and I ended up at the psychiatric hospital and I felt so much pains and it was like pepper poured on my body and I couldn’t sleep too. So my sister took me to the mental hospital and I was given drugs till I got well.

I Thank you and we are moving on with the discussion and we will be hearing some terms which you could try to explain whether you know about them or not. If you don’t know too that’s fine and I will explain for you. So what do you know about bio banking?

R1 I have not heard about it.

R2 I have not hear about it

R3 That is dealing with human beings ad take blood and bank it.

I So you are all right and bio means biological and banking is the place where we store money so bio banking is a place where we store human biological samples like blood, tissues samples for research. So now that we know about bio banking, so if how important will bio banking be in the area of medicine?

R1 This will give the researchers the opportunity to learn about how to treat cases since they will be able to research and learn from the samples that has been stored.

R2 For instance if my blood is taken and stored and I am O’ positive and someone has a stroke and they take the person’s blood too then they can research to know the medicine to give the person.

R3 It is key in the sense that if my blood is taken and researched into and certain things are found out of it, it will help the researchers to draw conclusions as to those with the same blood.

R4 In the outside world like India, if you have kidney problem or your kidney is spoilt, when you go to India, they can do transplant for you but Ghana, I don’t know if its possible. So the banking aspect if someone is in the state of dying, then we could remove the kidney and store it.

I So are there laws guiding bio banking operation in Ghana?

R1 Definitely there should be a law.

R2 I don’t know of any law.

R3 I am not aware of any law.

R4 I am not aware also and I am even hearing of the word bio banking today and even if there are laws, I wouldn’t know.

R5 I don’t know of any law.

R6 I also don’t know of any law

I We are moving on with the discussion and we have another term that is precision medicine and what have you heard about it?

R1 It should be precise medicine for the treatment of particular disease. So maybe malaria the precision medicine is coartem so it should be precise.

R2 I think it’s a medicine that is prescribed by a medical doctor and not just taking any medicine.

R3 I am hearing it for the first time but I think it’s a medicine that is there like Malaria which is prevalent in our environment and there are a lot of medicine for the treatment of malaria in our environment.

R4 I think it should be precise medicine for the same sickness and that’s how I understand it.

R5 People are resorting to herbal treatment of stroke which is not good and I have even used the herbal medicine before but my sister advised me to use the medicine from the hospital and that is good.

I So you are all right and precision medicine is targeted and right treatment and medicine given to individual based on their genetic assessment. So like you have malaria and we all take ACTs so we give the ACTs generally and you are done. But with Precision medicine, you have to look at the person and check the genetic system and then you give the drug that will work for them individually but not giving them all the same drug but what will work for your system and you are given the medicine.

Ok. So then can we apply precision medicine in stroke and how important will it be?

R1 I think that it cannot be because my little understanding is that there is different level for different people. I suffered stroke and I came here for 21 weeks and my brother suffered stroke and he came here for 21 weeks and walked back home. So if you give the two of us the same medicine I don’t think it will work.

R2 yes and in stroke we have different levels and I had mine in the hand and someone’s could have it maybe in the brain or somewhere else. So the medicine that will be given to A might not work for B so it depends on the type of stroke that you have.

R3 I think yes because when I was discharged and I came here for my medical report, I went through and its like they were testing me with all kinds of medicines, so it is like they were practicing the medicine through my body to see which one works so I think they should use the precision medicine to give me the right drug in order to reduce the cost.

R4 I will want to say that it should be long and from what all have said it depends on what caused the stroke and it may be due to a particular system so rather they should take time to investigate.

I So with the precision medicine like I explained we are not going to give general medicine to all like we are all here and we are sick and then they come and say this is the medicine so you all should take. No, rather with precision medicine, we will examine each one by looking at the genes and then we will see that when we give you this medicine based on your genetic analysis, it will work.

R Ok. yes

I Alright thank you. Are you aware of any laws guiding precision medicine in Ghana?

R1 No

R2 No

R3 No

R4 No

I How important will precision medicine be to the African broader picture?

R So far we have no results and so how do you treat the patient with that results so unless you have come out with results which shows that you have A, B,C, D and this person has used this and that is the results and it can be the best but apart from that it can be done but not now.

I We move on to our next term that is brain donation. So what do you understand by brain donation for research purposes.
R1 It is happening all over the world, either, heart, brain, or backbone or anything but I will prefer that when I am about to die, I write a note that donate my brain to the medical school to use it for a particular research and by then I am dead and gone ooo.

I Ok so you are willing to donate your brain for research and learning purposes.

R1 Yes

I So I am ask the rest what you understand by brain donation and whether you are willing to donate your brain for research purposes?

R2 To donate my brain for research purpose, no problem if only it will be useful. Because when you are dead you are dead and you taking it to the grave so if someone is ready to use it for research and it will yield something profitable for the future then I think it is something good and better.

I Are you willing to donate your brain when you die for research purposes and why?

R3 Yes I will donate my brain.

R4 Stroke is a dangerous disease so if you can research on it and it will help us why not. .

R5 The reason why I said I will donate my brain for research purpose is that most of the people that I talk to they ask me that they heard that I have a stroke and I tell them that its true and they end up saying that but I talk like someone that doesn’t have a stroke and I tell them that yes everything is intact. As times when we have a chat on our year group whatsApp page and they try to recall things that happened many years ago, like 46 years ago, then I will cite an example and bring them all back on it and they will say that upon all my challenges I still have everything intact and I will say that yes. So I think that the brain is useful and somebody can use it for research.

R6 For the brain donation when I am dead and gone and my family will allow you to take my brain then that is fine because I know they will not allow parts of the body taken but then for me when I am dead and gone I am gone. So why not I will donate my brain.

I Are you also willing to donate your brain for research purposes?

R1 The brain is the main thing that keeps the body going so to me if you die then the bread is also dead and not useful again so how will it work when given to someone else to use. [Interviewer intersects…the brain is not to be given to someone to use but for learning and research purposes] Alright then that is fine and I am willing to donate.

R Can I ask a question? Please does the brain function after you are dead? [All questions was agreed to be answered at the end of the discussion]

I What cultural, social, religious factors that will inhibit people from donating brain and body parts?

R1 This Jehovah’s Witness they are not willing to take blood so maybe they will not be willing to donate blood.

R2 If only the social environment allows that.

I You mentioned that if only family members will allow that your sample is taken for the research so I want to know can family be an hindrance to you donating your sample for research purposes?

R1 Yes they can be and in some families when you are dead the body belongs to them.

R2 But how because if the sample is taken at the mortuary how will the family know about it. [A participant responded that before a dead body is touched, the family has to be informed first].

I So what will make people refuse not to donate their body parts and you said family and religion can be a barrier? So what else could be done about it?

R1 So you need to sit down with the family and show them the document that their family member signed before dying so that they will understand.

I So what else should we done to promote sample donation for research purpose?

R1 It will be difficult

R2 There should be more education.

I What about those in our communities, what can be done to promote them donating their samples for research purposes?

R1 I believe that once you suffer from stroke you are able to donate and contribute to the research and that is what is common to us all.

I Do you think the people in your communities be willing to donate their samples for research purposes?

R That will be difficult because I am experiencing a stroke and I know the implications but when I am telling somebody, they will say different thing so there should be more education.

I The discussion is getting more interesting and I want to know what are your thoughts on blood donated for research purposes? I mentioned earlier on donating of brain for research purpose but now we are on donating of blood sample for research purposes. Are you willing to do that?

R1 That one is good and will be willing to do that.

R2 small blood donation is good and I will do that and its not a problem.

R3 Even this morning they took small of my blood for sugar test.

R Yes I will be willing to donate that.

I What will also be the barrier for people not able to donate their blood for genetic research? Are there cultural issues, religious that could prevent people from donating their blood? Will your family be willing to donate their blood?

R1 I think they will be willing to donate because finally it will help you to know that this sickness is in the family. And they will take care of that.

R2 You see the blood donation that DESPITE GROUP does, people will be saying that he is going to use it for blood money and so primitive ideas is hindering our progress.

R3 In my case there will be no problem donating blood because my junior brother is there and every year they go to donate and he donates a lot during those blood donation sessions and he joins the queue and brings the provision till he died.

R4 I know of a certain man in my area and he always has to donate blood and I don’t know if he has excess blood in his body [laughter by respondents] and I think every month he goes to donate blood and I don’t know what his problem. And what if its hypertension, I just don’t know.

R5 I think that if you go to the hospital and they take your blood they will screen it so if you have diseases or even hypertension you will be told. Then right from there you will be guiding yourself against those things and it will even benefit you.

I So you said earlier that education is one of the factors to use to promote sample donation for research purposes. Any other ways to promote people to donate their biological sample for research and learning purposes?

R All participants responded through education.

I let move onto another term and that is Informed Consent. What so you understand by informed consent?

R1 They will make you to read a document and if you like the content of the document then you sign.

I Like we did this morning and you all agreed to it.

R3 Yes like we did this norning.

R4 Yes like we did this morning.

I So there are types of consents and I am going to explain the types and you can tell me the types that you will prefer if you are to give your samples out for research. So we have the broad consent or generic meaning I am giving you my sample and you can use it for any research you want to do and I am ok with that. Then there is the restricted consent meaning I have given you my blood to be used for only stroke and you cannot use it for anything else. Then there is the tiered consent which says that you can use my sample for stroke, not malaria and for HIV and not the other. So you can choose which tests your sample can be used for. Then there is the dynamic consent which is online computerized based and the researcher could contact you to use your sample for any study they want to do and then you give your consent n that. So you are always in contact and its online based. So the four types of consent that I have explained thus Generic/Broad, Restricted, Tiered and Dynamic consent, which one will you prefer if I have taken your sample for research and why?

R1 The broad one because you take my sample and there are lot of diseases around and you can check for stroke, malaria and every disease.

R2 I will go in for broad because my family is not suffering from only stroke but so many things and anything can happen so.

R3 I will also go in for the broad consent because once the sample is taken, it could be used to run all the tests involved for you to be aware and be kept informed.

R I will prefer the broad consent as well.

R I will go for the restricted because for example with HIV, when they test you and you have HIV the thinking alone can make you die early so if you don’t know you can live.

I The example that I gave in defining the various types of consent doesn’t mean that we are going to do HIV test with your blood. It was just for you to understand what those types of consent meant. So meaning that if you give your blood to be used for stroke then only stroke and nothing else?

R Yes

R I will also go in for the broad consent.

R I will also go in for the last one that you said, the dynamic consent.

R So will you be pricked again anytime when collecting blood because the process is painful. [Interviewer explains that the sample collection will be taken once]

I I am happy that you have all agreed to give me your sample and expressed the type of consent that you will give as well. Now I want to know what is your opinion on blood sample storage for future use. So you have given me your sample and I am going to use and store some for future use. What is your views on that?

R I learnt blood expires after 35 days or if not why not [Interviewer said that blood well stored can be used in future]

R For medical care always there is a new issue so why store the blood for long time. Use it and if an issue comes up and it needs some research use it than the man says this and that.

I So the reason why we asked this is that if we have taken the sample for stroke research and we have done our first analysis and we still have the samples and can run further analysis on the samples again and run compare the findings after years of he studies. And if we need to take new blood sample from the patient then we need to come back to the patients again. So we can take the blood and store for long for research purposes.

R So for how long can samples be kept?

I Blood samples can be kept for a long time provided it is well processed and stored under the right conditions. Because if you don’t get the right power source and the lights goes off, then the samples will get destroyed and you lose everything. Also on the consent form, it will be indicated the number of years the samples will be stored before they are destroyed so you will be in the known and all is part of the consenting process.

R Ok.

I So initially I mentioned that this study is being done in Ghana, Nigeria and other partners in US and UK and so now we have taken your samples and you given your consent, now I want to share the data that we have with our partners. What is your view on data and sample sharing with other partners and collaborators in other countries like I mentioned. What is your view of sharing sample with them? At times we don’t have the equipment for analysis and we share data with the partners to collectively work on those samples so that we could get the results out. What is your view on that?

R1 The world is a dynamic world so we cannot keep the information to ourselves alone that you are the best. Share it the information will help others as well.

R2 It’s a good idea.

I What about if the sample is being used for commercial purposes and to gain some extra cash?

R You mean for some rituals?

I No, so the sample has been used for the research and there are some money benefits from the project. So the researcher has published the results and other donors have come in and there are some benefits. What is your view on that?

R1 Then they should give the participants some of the benefits.

R2 There is nothing wrong with it.

R3 It should be shared so that the next time you call the person he will come.

R4 There is nothing wrong with it.

I So in the process whereby incidental findings are gathered during the research and telling you that results will help you, so I took your sample and going to use it for stroke but during the analysis the microscope identified something else in your blood and I want to share that with you. How do you want the findings communicated to you? Do you want it by face to face like we meeting with you now, or call you by phone and tell you that Auntie we found this and that in your blood, or by email?

R1 In today’s world, there are so many ways of sending information. You can send me email or any mean but use what you can reach me fast. There are so many ways but I want the fastest means of sending the information to me.

I So you said you want it by email?

R I want it by the fastest means of reaching me with the information.

R2 I want you to call me and tell me that you have information for me then we come and meet one-on-one.

R3 As you invited us here and we are here so you can call me and I will come and meet you and then you give me the information. My doctor is my best friend in the world so when you tell me that then he could counsel me. If he sees me going down, he knows how to counsel me and so I think that I should be called and then we meet on that.

R4 the reason why I said that I want the fastest means of getting the information to me is that, Doctor might have travelled to the US during the time my sample is taken and analyzed and I need to get the information to me in the fastest means than having to wait till doctor comes. Even if the patient is in Accra, then there is no problem but if he has gone to the US then that is the problem.

R5 I prefer one-on-one.

R6 I want it like a book form so that I can keep for my children to keep so that they know that their father had this and that and they will know how to take good care of themselves and will know as a family history.

I For those who said they want the results released to them one-on-one, do you want your doctor around? Or how do you want it?

R1 I need the clinical psychologist to be around.

R2 You don’t know the disease that is coming.

I Yes you don’t know the disease that I found but someone will like the doctor or physician around so that when the results are released he could advice and counsel her on that. Do you want to meet the researcher alone for the results or you also want your doctor in attendance?

R1 Who are you giving the information? [Interviewer explains…..I am the researcher who took your sample and giving you back the findings] Then I prefer you teaming up with the doctor and giving me the information.

R2 If I know how to read then I am fine but if I don’t understand then I will like the doctor to explain it to me.

R3 I want my doctor to be there as well.

R So how much control should an individual have on their sample given out for research? So you have given us your brain, blood and tissues to be used for research. So how much control do you have on your sample given for research?

R1 I gave it out wholeheartedly so to me I don’t need any control again. [Interviewer intersects… so once you are done giving me your broad consent you are done with your samples? Yes.

R2 I think you need to be talking to the doctor and how to get in touch with him and know how the blood sample is fairing. So you should have some check on it.

I So from 0 to 100%, how much control should you have on your sample given?

R1 I have at least 40% control of my sample.

R2 I have given it out for research and I do not have any control over it again.

R3 Once I have given it out, it is the discretion of the researcher on how it should be used.

R4 I gave the restricted consent so they should contact me first before using the samples. [Interviewer asked….So you have 100% control of your samples] Yes

R5 I have the sample out so I don’t have the control.

R6 Human beings are sometimes something and they can so anything they want without checks. They take your samples and before you hear, he is conducting another thing with your blood and it is not good. I gave you my sample to do this study so why do you change it and there should be a collaboration between the blood donor.

I In that case how should autonomy rights to your samples be balanced with societal benefits for the use of your sample in research. So I took your sample and you gave me restricted use and only for stroke research but whiles doing the research I realized that if I run another test using your samples and that will improve another condition in the society which will also help them. But because you only gave me only restricted consent I cannot do that and if I do that too, the community will benefit. So how do you want me to handle that. Though the sample is for stroke study but once I study something on malaria, then it will also help to improve the malaria condition of the community. So how do we balance that autonomy and societal benefit.

R1 Once I have given my blood sample to the researcher it is up to the researcher to do whatever he wants to do. If he does stoke and malaria I will not know.

R2 That is why the participant chose the broad consent so you can go ahead.

I What suggestions do you have that can create awareness and improve on sample donation thus brain and blood sample donation for genetic research in stroke?

R There should be motivation with money and food stuff and we will be ready to get involved.

R You see blood, our brothers in that sect and assuming you see that this thing is disturbing and you want to take the sample and do something to see the outcome, what will they do. Will they still say No, and I have been interacting with the church people a lot and as for blood they do not want to go there meanwhile the disease is killing a lot of people. So what do you do for such a person and he is not taking the blood. I know some of them and I worked in a circle that when the thing happened he run away and went to the hospital and got the blood donated meanwhile the members don’t know and its not good. So there should e education and motivation.

R The education should me more.

I Alright thank you very much. Are there any recommendations or concerns on using these body tissues for research in Ghana which you will want to talk about but we haven’t mentioned it so far?

R1 Finally its all about education.

R2 Whether you like it or not this things should have been in place long time ago and I mean this research should have been done long time ago because the stroke disease is very terrible. I meet a friend today and I ask of friend A and I am told he had a stroke and he is dead. That is the language now and so as early as possible the research must be carried on and the findings will be published so that the whole Ghana and the whole world knows that this and this is what is killing us so we should be careful.

R3 In my situation, many women didn’t know that BP can cause damage so there should be more education.

R4 The education is very important because until I suffered it, I thought it was too far away from me. I never dreamt that one day I will suffer it to that degree but I will say that on hindsight if I knew maybe I wouldn’t have gone through that.

R I will say that people should go for frequent check-ups.

R Sometimes too the hospital doesn’t help because I was attending one hospital for 10 years and from when I was diagnosed to have a very low blood pressure to when I was told my BP is high, the hospital was not telling me but I was going there every day and a renowned hospital and I was going there and I even created and family file so every one of my family was going there so that they can have the history. But one day, I went there and the doctor looked at me and said do you know that you have BP and I said that I am not aware meanwhile the first time I heard of BP was that when the BP is very low then it is dangerous so to take come coffee or alcohol to boost it. But I said for alcohol no and rather I will take more coffee. So I started taking my coffee but as it was going up I didn’t know till one day I was told that my BP was high then I started taking drugs and I started checking on that from then. But even then the doctor can tell me that even this one it is within range so its not too serious so don’t worry but then on and on. But the day I was brought here is didn’t see anything and according to my wife, I was here for 21 days without talking.

R What happens is that the relationship within the doctor and the patient and before a particular educational program goes on the education and the working relationship between the doctor and the patient must unite.

R if I come to a hospital and for one reason or the other and you do a test, tell me the results of what I should do to minimize the effect of what you have seen but every day they take your BP and no one tells you of what they have seen and what you should do. They should even do that through the education.

I So with the idea of having a bio bank and we have all discussed that it will help to find the best way of handling stroke, from all that you have said, I want to know how should the bio bank be operated and governed. Should there be the need for an Ethical committee in place to guide the process?

R Yes there should be.

R Please explain the terms Ethical committee.

I Ok so Ethical committee is a board that checks that the right things are done by the laid down procedures. So if you say that you want to take blood or brain for this research on A, B, C then there is a board sitting down to check that you said you will take blood 5ml, are you taking that and are you running the tests that you said you will do. So that’s what the Ethical committee will check. So should there be such a committee or a law to guide bio banking.

R I think the law is already there and most of the things that we are talking about they are not new but just that somebody is not doing the work. The committees are there but the person to sit and do the right things is not there.

I So the committee should be enforced.

R Yes.

I I will like to say a big thank you to you all and its been a great discussion and I am sure you have learnt something from it.

End of discussion.
