# Supplementary material for: Research participants’ perception of ethical issues in stroke genomics and neurobiobanking research in Africa
Source: PLoS One. 2025 May 6;20(5):e0292906. doi: 10.1371/journal.pone.0292906 (PMC12054916; doi:10.1371/journal.pone.0292906)
Supplement: S3 File — (ZIP) [file pone.0292906.s003.zip › Files for PLOS ONE - updated March 2025/Accra_SIREN Stroke free control_FGD.docx]

**African Neurobiobank for Precision Stroke Medicine - Ethical, Legal, and Social Implications (ELSI) Project:**

Completed Transcript- FGD

Site: Accra

Designation: STROKE FREE CONTROLS FGD

Interviewer: Dr. Benedict Calys-Tagoe

Note Taker: Nathaniel Coleman

| **Items** | **Participant 1** | **Participant 2** | **Participant 3** | **Participant 4** | **Participant 5** | **Participant 6** | **Participant 7** |
| --- | --- | --- | --- | --- | --- | --- | --- |
| Age | 26 | 37 | 43 | 33 | 47 | 47 | 28 |
| Sex | Male | Male | Male | female | Male | male | Male |
| Marital status | Single | Single | Married | Single | Married | Married | Single |
| Ethnic group | Akan | Tulensi | Ga | Akan | Bulsa | Moseis | Dagbani |
| Educational level | First Degree | Postgraduate | Postgraduate | First degree | First degree | Senior Secondary | First degree |
| Use of internet | Yes, for research and socialize | Yes, for research | Yes, for communication | Yes,  Research | No | Yes, for work | Yes, for seeking information |
| Monthly income range | 101-250 | 101-250 | 501-1500 | 251-500 | 251-500 | 251-500 | 501-1500 |
| Location | Urban | Urban | urban | Urban | Urban | Urban | rural |

**Key note:**

I means interviewer

R means responses.

Findings:

Good morning all and thank you for making time for our discussion today on the ELSI project. As said earlier we are here to learn and share your views and there are no right and wrong answers and feel free to talk when called upon.

I What do you know about genetic research and have you heard anything at all about genetic research? When we say genetic research what do you know about it?

R1 Genetic research is a research that got to do with a particular issue that runs through a particular family. So a researcher will want to know the impact of that particular genetic issue that affects the family and for being positive or negative and how it begins or how it starts and that’s the smallest knowledge that I have about it.

R2 Like my brother rightly said but let me put it this way like there is a problem with a particular family and the researcher will like to know how that problem started and to know much about it and to drive out a solution as to how to solve that particular problem with the related or particular family.

R3 I am agreeing with what my colleagues have said and it’s a kind of research that refers to a problem in the family that runs in the family and there is a research to find out what causes that.

I Thank you very much but has anyone here has a personal experience by way of participating in any genetic research or do you know somebody who has participated in genetic research?

R1 I know of a doctor who is my friend who has participated in genetic research.

I So how important do you think that genetic research will be in managing people who have had a stroke? Do you think genetic research is useful in managing people who have had a stroke?

R1 it is useful in the sense is that when you get to know the cause or the origin of the stroke, and where stroke emanates from those who are suffering from it, then it will be very important. Because if you don’t know the original cause of it, then it will be very difficult in managing the case. So once you know the original cause then you will know how to tackle or manage it. If not to do away with it at least how to manage it.

R2 Like he said its managing a stroke or something with symptoms of a disease and I know initially the doctors will look at the background, area of living and all these questions comes up.

R3 To add to what my colleagues have said, I will add that if a proper research has been done about a family and the solution to that is being captured and probably wholly treated and managed and a different person comes in with a disease similar to that and prior to the knowledge that the person has in solving that problem, he would observe the symptoms here and compare them together and probably come out with a better solution to the situation at hand so it is very much important to do that.

R4 I think it is very important and you cannot use the same treatment for different people with different types of stroke but then if you do the research then you will be able to know the type of treatment to give to a particular stroke case.

I So there are a number of terminologies that we will be encountering, and I am going to ask your opinion. If you know them fine and if you don’t know them there is nothing wrong about it. I will start by asking whether you have heard of the term bio banking. Have you heard of that term?

R1 I have no knowledge.

I And can you guess from the name?

R2 To me bio means biology.

R3 Is it the storage of blood samples or something. [Interviewer interjects….well, its part of it]

I Any other?

R {laughter by respondents and interviewer}

I You remember I said there are no right or wrong answers and it’s a learning curve so if you have not heard of it, that is fine.

R I have heard of it so many times.

I And what did you hear of it?

R1 I heard it is about blood and blood issues.

I Ok. Let me explain it. So bio banking is actually the taking of human specimen for storage. You know we store money at the bank so this one is a bank of living tissues, and it could be blood, other body tissues, and could even be brain, parts of the brain or any other part of the body. So taking body parts and storing them for research purposes and that is bio banking. So now that I have explained it do you think that it has any role to play in the management of stroke patients?

R1 I think it is very important because sometimes when somebody passes on, you know after this postmortem and other things, they normally take of the samples to the lab and do some test. So what I am trying to say is that when someone dies of stroke and you are able to take some of the samples to the lab, we will be able to know exactly what causes the death or something of that sort. So I think that it is very necessary to do that.

R2 The question again?

I Do you think that bio banking has any role in the management of stroke patients? And whiles you are thinking of it, lets also think of this, what do you think that people in our communities accepting come of these body samples like blood, like brain tissues and we will come to a more details. How will people in our communities be willing to accept that? Are there any cultural beliefs, are there any religious beliefs, are there any social norms that you think can affect the donation of these body parts for research?

R1 Let me start and from my experience, my mum is very superstitious when it comes to these things. So in terms of blood issues she will say no to allow anything of that because of her superstitions and she might think that it is for spiritual purposes. And the way she believes I think people of her caliber has the same belief as well.

I Let me ask a follow up question. Personally will you be willing to donate blood for research purposes?

R Well I have been donating blood.

I I am not talking about donating blood for transfusion but for research purposes.

R Yes I will.

I ok. Any other view?

R1 My Uncle once called me that the daughter who is a nurse said she is bringing someone who is coming to take blood sample so that they run some lab test. But I was like I will not allow that because I don’t know what they will be using my blood for. So they came and took me to the lab and they took it and run the test. So the man is having something behind that those blood could be used for rituals or something.

R2 Some of them will not agree because they may know the kind of disease that they may have which they do not want to disclose to the family so by doing that the doctor will come to the realization that this is the kind of disease that the person has and do not want to disclose it. So they are hiding from taking the blood sample to store and study it.

R3 To add to it I think the primary reason for not allowing the drawing of the blood sample and body tissue to be banked for research purpose is that, it is true that when you study our culture and our beliefs these tissues and blood are being used for different rituals. And most of the communities are aware of this sort and for somebody to allow you to take sample for no other purpose but for research purpose, the person will think otherwise that you are going to use it for this and that and at the end it is going against me. And the secondary purpose is that there may be some diseases or sickness in the person and the person is aware of it and will not want a second or third person to know about that sickness and that one will also prevent the person from being used in the research.

R4 I think that stigmatization is also another factor and we are in a society where people would always be stigmatized and given names based on their sickness and because of this people find it very difficult opening up, to give themselves up for a certain research work. And the bio banking that you talked of as to whether it will be purposeful in terms of stroke research I think it is very purposeful and if it is banked, the purpose of the banking is to ensure that you get the information so lets say you have stroke or the sickness, when its bank, it helps to do more and get to see the extent to which it has gotten to. And if it is at this level, how does the person or patient behave. And because it is at this level, that is why the person is behaving this way. So then you are able to know the characteristics of a certain level of the sickness or disease if it is banked. And the research will take that when done.

I Alright. So he has shared his personal experience and he will be willing to donate. What about us? how willing will you be for donating your blood sample for research purpose.

R1 For future purposes I will be willing to donate.

R2 If I am able to be convinced that it will not be used for a purpose then I will.

R3 I will be willing to do that because it will go a long way to benefit us as my brother rightly said and if not to me but you have a family and you cannot predict what diseases the family will face in future. So when the research is done in it and they get to know more it will go a long way to help if and only if like my brother said the personnel there is trustworthy so that these stigmatization and name calling at the community level will be eliminated.

R4 So legally and with a reliable department I will do it.

R5 I think it will be purposeful and I will be willing to donate.

R6 I will be willing to donate but not because of money but if the details are well explained and assurance given then that is fine.

I That is blood donation but let’s go higher. Do you know anything about brain donation for research purposes?

R1 I have heard something about brain donation but I have a problem with taking someone’s brain and is it appropriate. I don’t know and taking my brain and fixing it back, I don’t get it.

I ok let me explain. So brain donation usually occurs at the point of death so there are people who will make a will that should I get to a point where it is obvious that I am dying, I want my brain donated for research purposes. So it is not a point where it is replaceable. And it is not when you are alive but when you are no more in need of the brain.

R1 So it is after death.

R2 So when you die will the brain function well.

R3 I want to ask about brain donation is it taking the whole brain or some part.

I Well what are your thoughts, will you be willing to donate whole or part and bearing I mind that this will be out to use when you are dead so we are not taking the brain whiles you are alive. So will you be willing to donate all or part of it.

R1 Some people will want that when they die they should be buried full so it will be very difficult to donate some parts after death. Then you are going incomplete but for me I can’t donate my brain.

R2 Is it something that is being done.

I yes but it is not something that is common in our part of the world and that is why we are doing this research to get what the general feeling is.

R1 I learnt someone donated his whole body to be used for learning at the Ghana medical school and I think it will be good for those who know book and those who learn a lot [laughter by all respondents] so that when we are using his body for research his name will not go off the books. Then we say that this person has done this and its being used for teaching. So I think it is very important.

R2 My question is with the brain harvest will it be used for the replacement of others?

I No its just for research purposes. So we are not talking about brain transplant but for research purposes.

R2: ok

I What are the potential barriers to brain donations?

R1 Some people want to be buried whole.

R2 There are also socio-cultural factors that restrict that. Some people are in the known community leader and it will be very difficult because of the cultural practices they will not allow and they expect that we have our culture, norms and values, traditions and cultural practices that when someone dies he has to go through these things and they perform these with the mindset that the person is full and that is why they are doing all these things. And per our beliefs it will be very difficult to you to get to that stage and be called and they believe that when you go through these rituals and performances after death, you turn an ancestor and your benefit to them as spiritual soldier is something that they cannot do away with. So it will be very difficult in the part of our society that such a person will agree for the brain to be taken out.

R3 I will not do that and I won’t just do it and if I have the power I will not also allow my relatives to do it but when I am dead and they want to give out my brain then that one I am already gone.

I What are your reasons why you won’t allow it?

R3 I want to go in full and I don’t know how the corpse will look like when laid in state.

R4 When you go without the brain the ancestors will say you are coming without your brains. [laughter by respondents]

R5 To add to what my brother just said, in our various communities, societies we have leaders and specifically to the traditional leaders in community, family and society, they are regarded as important persons and to the ancestors though they believe we don’t see them, they are there and they are real. So you being a leader to them you come and when you are going to them, they are saying that you care coming to report to them what you left over there. And it is your brain that you will use to report and you have left it there [laughter by respondents].

R6 No one knows how you are going to die, and you may die through accidents, you may be sick, and part of your body is cut off and you die so what about that. So I don’t agree with the basis people are giving and I know people go for brain surgery and after two or three months they die. So what about that.

I So what is your personal view on brain donation?

R1 They should be willing to do that.

I Will you be willing to do it?

R1 Yes

R2 You know these cultural beliefs that we are talking about here, so the ancestors are believed to see whatever happens here but we don’t see them to be touched and seen. So incidentally if something happens and you are dead, it is believed that they also see it. So if something happens and you die they know its through this accident that you are dead and your head or leg is chopped off and that one when you come there are no excuses [laughter by all respondent]

R3 I know a chief who was amputated because of diabetes but they never allowed the amputated leg to be thrown away. They went and paid at the mortuary and the amputated leg was stored until when the chief died then they added it and went to bury him. So these our beliefs we should not ignore it because if we do then we might not get. But I am not saying that personally I might not donate my brain but I just want us to look at what our society is made off.

R4 I think education actually plays a key.

I We are looking at the barriers then we will come to how to overcome the barriers.

R4 In Ghana we are much attached to the traditional customs and I think looking at certain perspective in the spiritual world there is nothing like physical body in the spiritual world. So if much education is given then people will not give certain excuses as to not to donate because in the western world people do cremation so I don’t get the reason why people refuse to donate part of the body and thinking that when they go to the spiritual world they will be questioned or something.

I Now I want us to look at how to overcome these barriers? What can we do to make those who are not eager to donate to become willing to donate?

R1 Effective, efficient education.

R2 Education.

R3 People are not willing to donate due to lack of education and like my brother said in the spiritual world, we don’t have a physical body and if people are made aware of what happened after death then I think they will be willing and personally I will be willing to donate my brain.

R4 People will want to do something because of the motivation or the benefits of doing that.

I What do you want to be the content of the education? What should we be telling them?

R1 People must be made to understand that in the spiritual realm and like where our ancestor who are our spiritual soldiers are working hard to protect us in society must be made to understand that in that part of the world there is no physical appearance and people don’t come to appear just like they are as standing. So in as much as we cannot see the physical standing of the person, the same way we cannot see the brain which is inner. So we need to be made to understand and also people must be educated to understand the benefits of it to society. Because if they get to know how beneficial it is to society and the individual who is being accepted to doing that then, I think it will go a long way to help.

R2 I think that you have to also educate us of the importance of the brain to the dead and is the brain so important to the dead and also the importance of the brain to the research so if you are able to explain further then they can also get involved.

R3 So we have looked at the barriers that prevents us to donate part of our bodies and I think we should be made aware that after death, your spirit is not in the body again, and it is the spirit alone that is going. So if the spirit leaves the body after death then even if a part of the body is chopped off, its not the spirit that is chopped off. So with that people will be willing and in our part of culture here, we the blacks we believe our traditional more than the western part does and if we preach to them to know the importance of what my sister said over here, our people will be willing to do that and to cut it all, when people know Christ then that will go a long way to help because that will get them educated more.

R4 I think our community and religious leaders play a key role when it comes to the mindset of brain donations. Because looking at the 21^st^ century we call something like civilization and before a king will be enstooled, there is something like educational background and they send kings and queens to go to school to get more knowledge. So I think if our tradition sectors are even thinking of education then I think it will help with lots of education to the people.

I Ok. Are you aware of any laws guiding Bio banking in Ghana?

R1 No

R2 I have read through Act 525 of Ghana Health Service but have not come across that.

I Lets move onto another term called precision medicine?

R1 Is it about the exact medicine and you have investigated and you know what you are giving the person.

I Another name for precision medicine is tailor made medicine. So two people may have come with a condition that is stroke. But we will end up treating the two people differently because of their different genetic make-up. So that is precision medicine and that is what this whole project seeks to achieve. So at the end of the day we will be able to come out with tailor made medicine so if Mr X has a stroke and madam Y too has a stroke, we may not necessarily give them the same treatment because their body responds to the medication will be different based on their genetic make-up. So that is precision medicine. So lets talk a bit about it because I have explained it and lets discuss it. So can this again be applied in the management of stroke?

R1 yes it can be.

I So I may go back to the questions asked earlier about the beliefs people may have about it and the cultural settings that we find ourselves in and are these going to affect our practice of precision medicine? Because you can tell the genetic make-up of an individual, you will need to do genetic studies and doing genetic studies involve taking bio samples. So let’s have that at the back of our mind and how easily can we practice precision medicine in our part of the world.

R1 It will be quite difficult because as mentioned earlier, bio banking it is where the issue is coming from and patients will find it very difficult or an individual will find it difficult to accept that you take a sample of him or her. So in that case you cannot determine the source of treatment that will be prescribed to the person because you don’t have the genetics background so it will be very difficult in that direction.

R2 I think it is very difficult to come by this precision medicine because of our different beliefs of not allowing these kind of researches to take place. If the research indeed have its own way to take place, and then people are aware of these results and other things, then when people come of such nature then we will be able to address them in that manner. But since it will be very difficult for us to even get the samples to be researched on, I think it will be very difficult for us to be able to implement this. But if we are educated more and then we are willing to donate part of our bodies for it to be researched then I think it will be done easily other than that it will be very difficult for us.

I Is it important for us to practice precision medicine in African?

R yes it is very important.

I So how can we move the frontiers to get there. We are not there yet. How can we get there?
R1 Again we come back to effective education, create awareness and get the society leaders in involved and I think most of the leaders people believe them so whatever they say gets to the person more than the outsider comes in. So when we get our society leaders involved, whatever they say to the community, they pick it up and they are willing to do whatever they are asked to do.

R2 And also by doing more of such researches.

R3 We are also in the part of the world where poverty is also eating us up so I think that a research of that sort should also involve packages for motivation to be able to convince people and if a package is given to someone and the research is conducted I will make him to understand that in this research I am not buying you. But you take a token in support of yourself and I will keep the information.

I Wouldn’t the giving of packages influence people’s participation? So like you rightly said there is poverty so if I realize that there will be a package for my participation, I will go and participate not that I am willing but because I want the money. Then it forfeits the purpose of the study.

R1 Yes

R2 The point that I am coming from is that I think that the research will come out with the details of what is involved in the research and even mentioning of the name and the agenda might be disclosed here with the age but the person will not come out with the name of the person. So as much as there are things that will behold the ones being used for the research, there are also other things that the researcher is holding on that after the research they can be able to give out.

R3 To add to that not that those things will influence them but the person should be made aware and be educated on what the person is coming to do, the benefits to the person and to the community and the world at large before the person gets involved. So after everything is done then that will serve as a token but not specifically make the person aware that we are going to give you this if you come to do this we are giving you that but the education should get to the people very well so that after which that one some small token is given.

R4 I think the package somehow is necessary because some time ago, the University of Oxford came to do some research here and it was on corruption and we didn’t know there were money involved and they never told us too but in the course of what we are doing, they played a game and that is where the money started coming in.

I So your decision to take part was not based on the money?

R4 yes [interviewer intersects…..so later people wanted to take part but then it was too late] yea

I Alright thank you for those submissions. Lets move ahead and talk about something else. Informed consent. When you hear of informed consent, what does it mean to us? what is informed consent.

R1 Informed is to be aware of something and the consent is………

R2 Informed consent is like you want to do a research and you want the person to participate and the informed consent form will tell the person what you intend doing, the details of the study, the benefits and if there are compensations and then signature like we all did before starting this meeting. So informed consent is giving out details of the study and our acceptance to participate.

R3 The willingness of you accepting the outcome of whatever you are to do.

R4 On a particular issue, one is informed of his consent and when he consents then it is considered informed consent.

R5 Like you are already aware of what you are to do and you agree on to do that.

I There are various types of consent that we can give. Do we know of the various types of consent that we can give?

R3 Is it an in consent form, assent consent for children.

I Any types? Ok so beyond that there are various degrees of consent and I am going to run through them because I am going to ask your opinion of them. So there is what we can the Broad consent, where you come to me and you want to take my sample for research and I say ok take it and go and do whatever research that you want. So with Broad any research at all you can go and do with it. So that is the broad or generic consent. Then we have the restricted consent so you come to me and take the sample and then says I am going to use the sample for stroke research. So I say you can only use it for stroke research and hypertension and not diabetes, so restricted. And then there is the tiered consent and it comes in layers so I choose and pick so you can use it for stroke but you cannot use it for diabetes. You can use it for hypertension and you cannot use it for this. And then finally we have what we call the dynamic consent and in dynamic consent there is a constant interaction between the researcher and the one who gave you the sample. So anytime I want to use the sample, I come back to you and say do I have your permission. So that is dynamic and in our modern day that takes place via the internet because the researcher may be sitting far away but through the internet you can interact. So these are the degrees of consent and if any of you were to give your sample for research purposes, which of these consents would you be willing to give and why.

R1 I have a question and I have seen that in some research they will tell you that I am going to use the sample in future research. So where will that be.

I That is in the issue of storage and for future research is it in the same area or another area and that is where the tiered system comes in so I can choose and pick which ones I want. Ok so now lets continue with the answers for the previous question.

R1 I will go in for the broad one because if I go in for the restricted one, how will I know that they are going to use it to do what they told me. So its better I go in for the broad one so whatever they will use the sample for they can go ahead and use it.

R2 Also how will I know that they are using the sample for what they said they will do so like I selected where they will tell contact me whatever they are going to use the sample for me to permit them but how trust do I have that maybe my sample will be used for whatever purpose I choose to be used. So with that there could be only trust here that my sample could be used for this and that alone and if they want to use it again, they will contact me again then I will choose the dynamic.

I So for the purposes of the discussion let’s assume that the trust is there.

R2 Then I will go for the dynamic.

R3 So just as he said if I am going to monitor how they use it, then I will choose the broad one but if I am not able then I will choose the dynamic one.

R4 I will go for the restricted because I am not a researcher but a lay man and you have taken my sample for research so tell me this is what you want to do with my sample and I don’t have any knowledge but the information is that it is for stroke research. So if you should take my sample for stroke and you want to use it for other purpose, I will not be there to know. So I should also understand it well. [interviewer interjects…so you want full disclosure] yes.

R5 I will go for the dynamic with two reasons. First because I want to experience new things so it won’t be a problem if they take my samples to do three, four, five, six researchers or even for future use. And secondly I think it also has its advantages and it can act as the tiered because you can decide if maybe this research or you can say no to a particular research.

R6 I prefer that you come to me anytime you need to use the sample [Interviewer interjects…that’s dynamic] because I am not sure of the monitoring so if you want to use the sample, contact me and give me the details and assurances then we move on.

R7 I trust the researcher and the researcher doesn’t need my consent alone, I need the researcher consent too and in the first place I have agreed that you can do it but which one does the researcher chooses now, and which one is the researcher interested in? Because there are many people around and you have seen and chosen me and when you decided to settle on me, fine I also agreed and which one is also in your interest?

I So the researcher has an interest to do research so he wants a consent that will allow him to do research.

R7 So personally I will go in for broad and the reason is because I won’t go in for dynamic because I don’t know when I will die probably they will run a test and the next test would have saved my life. And I will then be saying no, when you need to do another test before you contact me and so when I pick that one and by the time you come, the results might have helped me and I would have restricted that. So I will go in for broad and it will give me a holistic view of myself, my family and that will help me.

I So there are two things that I picked from the last submission that will lead me to my next sets of questions? One is how much control should an individual who gives his samples have over how that sample is used.

R1 So that is what I asked and you said assuming the trust is there. So lets assume there is trust so the person giving the sample to the researcher has some amount of control over the samples.

I We agree but how much control do you have over the sample. But how much control should you have?

R1 It balls down to an LI (Legislative Instrument) [interviewer interjects….can you break it down to everyday language]. If there is a law and as you mentioned about the law on bio storage or banking, if there is a law that will tell me to have a knowledge on the limit of the researcher and this is my control, it is appropriate and how one will have the control, it will be very difficult because if I have agreed that my sample is taken and its taken away I don’t have the control because I am not a professional in that area. And I won’t have the control. I don’t know if you take my sample in a particular chemical like this, is it restricted in the test of the particular sample in the chemical inside of it can be used for other test.

I They can be stored and used in other researches.

R2 Everyone is saying trusts but trusts are meant to be broken. So before a researcher comes to you to say he needs sample it will not be done verbally because if that is done, then it will not be backed by law but then I think that he will give you something to sign and that moment that you sign, it actually limits you so I don’t think that the donor has much control.

R3 So my control is before I sign the consent but after that I don’t think that I have the control again.

I So the import of that question is that most of the sample in fact all the samples for research are meant for public good. So now you are trying to balance your autonomy or your bio rights with the public good so how do we balance the two. I have my right, yes but then there is also the public good. So how do we balance the two and that is what we have been talking about. The second part of what he said which I want to bring back is the return of incidental findings. He made mention of something that they find that may even save his life. And you know the samples are taken primarily for research but its not diagnostic like we are running test to find out if anything is wrong with you. But there are incidental findings where I have taken the sample for research but then I have come out with some findings and I think this will have untoward effect if I don’t communicate this to you who gave me the sample. In such an event, how would you want this incidental findings communicated to you? Would you want it sent to you by email, or would you want someone call you by phone, or how would you want such incidental findings communicated to you?

R1 I think this incidental findings, I will prefer it by face to face because some of the issues are not meant for you to just hear it this way. But if the person is before you and he is a professional he will know the way to even approach you and even if it is a deadly something, the way he will talk to you ahead before breaking the news to you will make you a little relieved than just the phone call or sent through email. And now there is kind of advance technology and you send the thing to me through email and I want to know what it is so I google it so find that this is the problem.

I So you will want a face to face. Does anyone think differently?

R2 The same thing but you have to add a psychologist to psyche you and then educate you as well.

R3 I think what he is saying is right so through a counselor or psychologist but its based on what the news is, is it good or bad and if it is good then you can even tell me on phone but if you think its bad [Laughter by respondents] Then through a counselor or psychologist you can deliver the message.

R4 Well it depends on the incidental findings and if its relevant, then face to face but if not that relevant then through other means, phone or others.

I Now he again ask that if the sample is taken, will it all be used in the same tube that it is taken? So the question that I am posing is this. What are your views on the storage of blood samples for research purposes for later. So the samples are taken but they are being stored for use later for the research as opposed samples that are taken and used for research immediately.

R1 Will it be for the same research or another. [Interviewer interjects….It may be for the same or another research]

R2 I have a view on that and my view is that it is not appreciative why it should be stored for future use. Why because the labelling might not be right and if the one that has taken the sample has not been able to label it well, it could be that Ama’s sample could be used for mine, Kwabena’s sample used for Kojo because it is not well labeled and the person might not see it well. So the results or outcome might not give a true reflection of who I am or what might results would have been so mine could be used for the other.

I So if that issue can be overcome with proper labeling, would you have a problem with storage for future use?

R1 I will not have a problem if proper labeling is done on the samples for future use.

R2 If proper labeling is done then there is no problem for future use.

R3 So you have to informed the person that sample will be stored for future use because you cannot just take the sample and use it without informing the person. [Interviewer interjects…..so it should be part of the consenting] Yes

I So generally we do a lot of collaborations during research and so what are your thoughts on sharing of research data and this data could be blood samples that are taken, it could be brain images in the form of MRI, CT scans or it could be in the form of other samples that are taken. And the sharing could be with local or international collaborators. What are your thoughts and do you have any reservations with that?

R1 I am much concerned with the sharing of the data. Because sharing of my data can be used for a different research that I am aware of. [Interviewer interjects…..but you were told when the sample was being taken] yes but I was not told that my sample will be shared with other collaborators.

R2 I know of data protection law but I don’t have the details so if you are going to go according to the law then I don’t have a problem with that.

R3 As you mentioned earlier that every research is for the general public sake, so the purpose also is not to restrict yourself to a particular environment but to also gain knowledge in other areas and understand the dynamics of those areas pertaining to the things that you are researching on. So I think that probably what might have been the cause of a particular research you are looking for in Ghana might not be the same cause of what you are doing in Nigeria. Or any other country outside Africa. So you want to get the dynamics and I think that it is not from our parochial interest and the research taken from me is to ensure that it gets the benefits and society benefits from it. This research thing that we are talking about here before we started and as a country we even had the education and we can speak English, it was the same way difficult to even get somebody’s land to put up a school and people were made to understand that it was for the societal benefit and then people gave their lands and that it is good and it will help the society. So I think that because it will be of benefit to the whole society, I am even going to be the center of it that because of me, society is benefitting from it and I think it is appropriate.

R4 I think the third party here that is going to share my data with is a professional body and there is trust and the assurance given me by the first party, the second party is the same with the third party coming in here. And maybe like my brother said, maybe your findings might not necessarily getting a certain diseases that might have taken the life of me early but the next one coming in might rather find it and communicate to you. And that one will also be channeled to me and that will save me and that one will not be to me alone but society at large so I don’t have problem with data sharing and I think that the question here again is how much control do I have on the sample that I have given to you. So I think I don’t have a problem with that.

R5 Normally I know that when you give the data it is coded so sharing will not expose anything so that is ok.

R6 Since he was saying that he doesn’t have much control I want to know that in signing the informed consent, is it stated? [Interviewer interjects…..It should be stated]. Well if is it stated then I should have a second thoughts but if not and later and later I get to know that it has been shared then I will have issues with that.

I We are gradually getting to the end and now that we have all shared our consents and samples and how they will be stored and all that, so the question is how should bio banks be regulated? We are all concerned about the samples that we give so how should bio banks be regulated.

R1 There must be an LI, again there must be a legislative instrument that will pass by our law makers through the professional bodies to regulate and give a full consent depending on the area that the research might be going the individual must have the right to know the detailing of all these things.

R2 So there should be regulations or regulators.

I Should there be the need for a regulatory body?

R1 yes

R2 That is what I was saying there should be regulators for such activities.

R3 So there are laws in the country but as you ask earlier on we don’t know if there are particular laws governing that bio banking and if it should be there then the supervision should be enforced.

R4 I think that the Ghana Health Service has a role to play when it comes to the bio banking.

I ok. So what suggestions do you have that can help raise awareness and then to improve people’s attitude towards blood sample or tissue donation for research purposes and to encourage people to adopt the practice.

R1 We said earlier that there should be education.

R2 There should be exposure to different cultures.

I Exposure to different cultures can you explain that please?

R2 So when there is exposure to different cultures people actually experience the importance of these donations and also some of the mindset we have towards donation and donating part of the body so if people are exposed to different cultures to see the relevancy in donation for research purposes.

R3 I also think that our religious groups must also be given the understanding for all those things. And these should be the most target because the religious groups makes people to believe and understand that nothing of that sought can be except to say what they believe in. And so they don’t think that you the researcher what you will do will bring the change. If the religious groups and men and women in our society are given the opportunity to attend workshops when they are organized for them to have the understanding of these things, then I think it will help them.

R4 I think it all balls down to the same way and for example if we are going to attack a group of people you don’t just go straight to the people and say that I am attacking you guys. But you figure out their strong men and when you capture their strong men then you will definitely get those behind them. So when we target the various groups and the leaders in the society and get them trained and get them onboard and I think that one will solve the problem for us.

R5 I think the researchers also play a role in this because they come to us the lay people that they are doing a research for a specific purpose but later on we will find out in a publication that it’s a different thing. And since we the lay person don’t know any laws that gives us the right to either sue or come forward and I think the research body also plays a part.

R6 To add to what is said the regulation should start from the national level and it will get to the patient as much as possible.

I In conclusion is there any concern or recommendation that anybody has regarding all that we have been discussing, bio samples, bio banking, precision medicine and things that has to do with the legal, ethical and social implication of genetic research which we have not discussed here. If you have any such concerns please lets have it and if you have anything you had wanted to talk about hoping that a question would have come out but the question hadn’t come up. If you have any concerns we want to hear it before we wrap up.

R1 My concern is that I think we need to do more and give education to the general public.

R2 I am aware of a friend of mine and we were discussing issues and it relates to diabetes and others and we were discussing and I just mentioned to him that we have issues of recent times than those ones in the other part of the world because of our way and style of eating food and he told me that in the olden days and our mothers and fathers in the village why is it that they are not having problem with us but those in the cities. And I said yes its because of the city life and the life that we live in the city and the food is the cause but those in the villages that you know they eat fresh vegetables and all those things and there are no chemicals in all those things. I think that it is a good initiative and a good thing and I would even like to be an ambassador in this particular program but lets enhance it more and lets get more education to the general public and that will make up understand the real purpose of this things. Because people think that it is for certain purpose because the errm, errm, ermm, there is a term I want to use, the socio-cultural beliefs, the perception and it is eating up people in our society. So let’s open up but in all, its very appreciative.

R3 I think in all it’s a very good move and initiative as my brother rightly said over here and the only thing that I will bring on board particularly to the specimen that you will take for the various tests. If you come to me that you want to take my brain or my blood for a test on stroke or a research on stroke, probably as a layman I will not know the causes and whatever is the cause to that stroke over here but if I should be educated on, and been made aware of the causes of stroke and what the stroke is, I think that one is also good. Also get persons on board like my brother says over here and if people are trained and sent particularly to the rural areas I think that one will also help and enhance the program and that is a good thing.

R4 I also will recommend that the ethical issues and the confidentiality of the data must be well explained to the person so that the person will be willing to participate in it.

R5 I am thinking of the next generation and how will they accept to do these body part donations. So my suggestion is that I think we should start from the school at the infant stage so that we can impact in them and they will know the importance and relevance when it comes to body part donations in terms of research. So that the next generation will not be discussing are you willing to and are you willing to and so public education is good.

R6 I also support that he said.

I I will like to thank you all for being part of this discussion and I believe that you have all enjoyed our discussion. Thank you very much for freely sharing your opinions and we really appreciate them.
