# Supplementary material for: Research participants’ perception of ethical issues in stroke genomics and neurobiobanking research in Africa
Source: PLoS One. 2025 May 6;20(5):e0292906. doi: 10.1371/journal.pone.0292906 (PMC12054916; doi:10.1371/journal.pone.0292906)
Supplement: S3 File — (ZIP) [file pone.0292906.s003.zip › Files for PLOS ONE - updated March 2025/Accra__CAB Members_FGD.docx]

**African Neurobiobank for Precision Stroke Medicine - Ethical, Legal, and Social Implications (ELSI) Project:**

Completed Transcript- FGD

Site: Accra

Designation: CAB FGD

| **Items** | **Participant 1** | **Participant 2** | **Participant 3** | **Participant 4** | **Participant 5** | **Participant 6** | **Participant 7** | **Participant 8** |
| --- | --- | --- | --- | --- | --- | --- | --- | --- |
| Age | 44 | 50 | 66 | 36 | 27 | 47 | 66 | 48 |
| Sex | Male | Female | Male | Male | Female | Male | Male | Female |
| Marital status | Married | Married | Single | Married | Single | Married | Married | Married |
| Ethnic group | Kokomba | Ga | Krobo | Akan | Ewe | Ga | Ga | Akan |
| Educational level | Postgraduate | Postgraduate | Postgraduate | Degree/  Graduate | Postgraduate | Degree | Senior  Secondary | Postgraduate |
| Use of internet | Yes, for work and social | Yes, for education, information, entertainment, social interaction for work | No | Yes,  Research | Yes for information | Yes for Facebook  And  whatsApp | Yes,  Personal | Yes, work |
| Monthly income range | 1501-3000 | 1501-3000 | 501-1500 | 251-500 | 501-1500 | 101-250 | 101-250 | >3000 |
| Location | Urban | Urban | Rural | Urban | Urban | Urban | Urban | Urban |

Key note:

I means interviewer

R means responses.

Findings:

I What do you know about genetic research and have you heard anything at all about genetic research? Do you have any experiences?

R No response from respondents and a long pause.

I When we talk about research in stroke in general what comes to our minds?

R1 we will want to know the origin or the cause of the stroke. And why is it that this person has gotten the stroke and also we want to define what causes the stroke because some conditions look so similar and when somebody begins to experience some type of none wellness, then one could think it is stroke whiles it might not be so to research into it is very helpful not to get the wrong medication into the cause of the none wellness.

R2 I also think that it will also involve the management of stroke both at the hospital and at home.

R3 the research helps to know the condition that leads to people getting stroke.

I What is your idea on genetic research study into stroke?

R1 I think it is a branch of biology that is concerned about genes and it is something that is hereditary and runs within families.

R2 I am wondering whether it has some medical background as to how people get involved with diseases.

R3 I think that to know whether certain families are more vulnerable than others and disease as it relates to high blood pressure, diabetes, cholesterol or high cholesterol or a combination of all in the family that makes the person predisposed to stroke.

R4 I think that I agree with what everybody has said and the genes runs in the family and you get your genes from your parents or grandparent but for me being a stroke survival, I remember I learnt something that your genes can change and there are many factors that can contribute to your genes changing.

R5 The little that I can add is that apart from your genes, our way of life leads to all these stroke things because during our first CAB meeting on SIREN, we learnt a lot of things on the way the community members go about their life in James Town. The way they eat, the kind of food they eat and their life styles leads to getting stroke and that is what I learnt.

I Are there benefits of genetic research and if so what are the benefits in medicine in general and stroke specifically?

R1 Yes there are and what we learn could help prevent others from having it by making recommendation as to the kind of management, rehabilitation that stroke patients should have and it could be beneficial.

R2 It also makes us aware and raises our level of awareness so that we don’t get privy to stroke and we get to know more on the disease prevention.

R3 It gives us information on how a certain group of people are more proned to stroke in a specific area and why others are not. So it helps us to know the risk factors and why these group of people are proned to it and why others are not getting it and it gives us a general idea on stroke.

R4 It helps with investigations by doctors in finding the diagnosis as well. But sometimes there might be something that might look like stroke and you might not really know what caused the stroke so if you do more research it will help us a lot to know the causes, diagnosis and how to manage it.

R5 It gives you the opportunity to know what causes the stroke and what can be done to be able to cure those causes.

R6 I have a question and it is that are there laws backing the works that the researchers or doctors do because you know some things are not clear but they are being done so is there anything to back those actions. Secondly, those selling these bad drug products in buses and at the open to treat stroke. We are told that those drugs are not good but what is being done to control these things. Is there a law backing these activities? [Interviewer explains that we will come to that soon ok and the questions will be addressed]

R7 I think that genetic research will provide the needed information to educate people and expel certain myths. I know that some people think that stroke comes about as a result of some spiritual causes and research helps to educate people and by so doing it will change those perceptions. And also it will reduce the prevalence of the disease.

R8 As we do genetic studies and come out with findings on the genetic causes of stroke it will help a lot because two people might be suffering from stroke but the type of genes causing it will be different from each other. Then that will also focus on when it comes to treatment so then we have what we call precision medicine that is giving what is specific to a particular individual that we found. So then we look at the genes of this person and we find out that this is what will be good for the patient based on the genes. Also those that are yet to get the stroke the advice that I will have for you will not be the same than those with stroke but it will be more on prevention and it will help us to individualize our treatment for the patients.

I so any other more on genetic research?

R Yes, so genetic research looks at genes, and we understood from submission from one of our colleagues here that they may run in families, communities or races, and the benefits of that are immeasurable. One of it is that the genes are affected by what happens in your environment. And therefore it provides clearer understanding why these people in the community get this kind of disease that are not that common than that community. Because the genes are shared by family, race and community it is just a window that you predict what is likely to happen if you share the same pool of genes coming from this particular family, race or community. The problem is that it avoids using a broad brush approach to medical service delivery where you are now in the position to be much more precise and targeted and its important that once you pull genes and you create a bank of genes, it helps you to be more precise in how you predict on what will happen to one or group of persons. Also, once you recognize that these group of genes are responsible for the kind of unwellness that we are seeing in this class of people then you can do genematic engineering and twist the genes a bit and it may avoid the consequences that it we have now predicted. So we will see that when we get to understand this as a community, most people will be happy to support the process. Whereas in the bank you share your risk and once we have several banks of the genes, then we can see that we can begin to have a much more targeted approach and be more precise in how we deliver quality health care to specific group.

I Thanks for the submission but two things came up, being precise and having a bank? When we talk about precision medicine, what are your thoughts and have you heard about that before and what does it mean to you?

R1 Before we go on can I ask a question? Is stroke transmittable? Can it transmit from one person to the other? [Interviewer intercepts…to answer your question, stroke is not a communicable disease.]

R2 for me precision medicine is the opposite of try and error and you know what you are looking for your know your targets and you go in just for that. No sideways and no forward backwards but you just pinpoint your practice because my then you know what you are looking for but then the try and error seems to have eclipse the regular medical practice.

I Any other point on precision medicine and are we aware of any law on precision medicine that guides precision medicine in Ghana? Is precision medicine beneficial at all?

R1 I don’t know if that relates to protocols from particular types of strokes as relates to the causes of stroke that the person has. That’s what I think.

R2 maybe I can set the ball rolling. Because we are saying that genes or genetic research is to identify specific genes that may be causing some conditions which will lead to unwellness and it is possible that through careful targeting and instead of giving medicines that will be killing other things and including that one. You target and direct your treatment to a specific cause of the offending genes and remember anything that we take to treat is toxic and to avoid the toxic going through the healthy part of the body that will not necessarily require that. There are much more efficient in how then though these treatment are quite expensive and you are much more efficient in delivering the right dose to the specific target. For the benefits, the social implications are obvious and if you take something that will make you stay in the hospital for the whole week, and you are able to target then you could reduce your one week to maybe a day. But the requirement of having relatives or caregivers and the discomfort it brings, the monetary and financial implications that it brings you will find that when we are able to perfect the act of precision medicine we may be doing a great service to ourselves as social being first and to the health economy because the cost of taking care of sick people will have come down.

The issues of laws and policies is that once you are looking at genes, and it will come from Uncles and Aunties and all that you need to have enough of them together for your prediction to be precise. So talked about banking and once you pull all these genetic information together then the concept of the bank allows us to get more data. So what it means is that as a country, we need to have laws that will deal with how to take tissues of human beings and keep them in a bank and the bank will have my information and that of others. And that information if carefully managed once they see what is in my genes and we are twins then it’s a window into you what is in you as well if we see that. So we don’t just need general laws but specific guidance that relates to how materials from human beings called biomaterials are procured, used, are banked for future use and how people are allowed to go into the banks. As we speak the laws that we have in this country there is none that I am aware of that is on gene research, genetics, gene ethics and genomics and the simple name is bio banking or neurobanking and we don’t have one here as we talk.

I There is a term that we use in genetic research called Biobanking. What do you understand by biobanking?

R1 When you do precision medicine you also save the body from general medicine which can harm the body in some way. Because the person may have healthy tissues and because you are not doing precision medicine, it will harm the other tissues or body chemistry which would have had no problems. So that precision medicine because you have a target it is easier. And Bio banking is getting tissues from a living person and preserved in a bank for research.

R2 From the little engagement that we have had with communities across the country, there is no guidelines to the treatment of stroke in the country and so when it comes to the precision medicine that is where we have only three centers now that is Korle bu Hospital, 37 Hospital and Komfo Anokye Hospital and these are the hospitals that I can say maybe use the right procedures for stroke. So if you go to community you realize that people refuse to use the right approach regarding treatment of stroke. Some claim it is a curse and it will never be cure and it is there for life, so that is why the stroke organization is in to help to reduce the burden of stroke.

I So how do you think it operates?

R It operates more like a blood bank and someone can say that when I get an accident and you are able to get there before I do, take my organs and body parts and preserve them for research. But if care is not taken and somebody gets the code of somebody else’s bio system then the person will get all the information about the dead patient. So they are making sure that when they get the sample it is well kept under tight security.

I So we are going to talk about donation of body parts? So what do you know about brain donation?

R Is it only brain donation because we hear of kidney donation and we marvel as it is done overseas. We keep talking about that and we think we can’t have it here because we don’t have those practices here.

I Why don’t we have those practices here? Is it that we lack the expertise?

R1 maybe it is not well publicized and the public is not well educated.

R2 For the education its everywhere and I remember a case in the UK where I had to give therapy to someone before kidney transplant and he wanted to know the character of the donor because if the person has a character, he feels it will be transferred to him. [Laughter by respondent]. So the education is very important.

R3 Also religion sets in here and the Jehovah Witnesess doesn’t accept blood donation and they have their reasons and all these things draws us back.

I What cultural, social, religious factors that will inhibit people from donating brain and body parts? Can we have specific cultural and religious practices that could inhibit these activities?

R1 It is well known that the Jehovah’s witness do not accept blood donation of any kind.

R2 There is a church also in my community and they don’t even allow syringe to be put in the anus of the children and when even it is time for Ghana Health Service to have polio and other immunization then they will have to go there with police for the children to be forced and immunized. I think I had an uncle who signed MOU with the hospital that when he dies the body should be used for training by the medical school. So there are still many people in Ghana who also believes that instead of throwing my body away why don’t I allow that the body could be used so that others can learn out of it. So it varies.

I I am curious about your uncle and do you know why he made those decisions?

R1 That my uncle didn’t have any children and he might think that he may die and people will be worried about how to go about my burial activities and all that so he decided that this is what I wanted to do so we even got to know when he passed on that this is the MOU that he has signed with the hospital. That this is what he wants the body to be used for. So when we went to make arrangement for the burial and they told us that they cannot release the body to us.

R2 I don’t know about brain donation but I know of those that has to do with fertility. In spite of these religious beliefs that you are talking about, I think it has to do with what the individual considers important as against what religion prescribes. I am saying this because a person who goes to this church that doesn’t allow children to be immunized or a Jehovah Witness will do anything that the doctor asks for get on fertility to get a child but may not take blood or any other medicine. So I know about fertility donation thus taking people’s eggs and have children but they will not donate brain or other things like that.

R3 What I will add is that I don’t know if some of the cases are recorded but we hear of some cases even here at Korle bu. There was a boy who needed blood and the parents who were Jehovah’s Witness refused to allow the boy to be given the blood and the doctor said it should be done and the parents threatened them legally. So what the doctor did was to let the parents go when it was visiting over time and later gave the boy the blood and when the parents came the next day the boy was fine. So this is what happened years back in Korle bu and one doctor told me this and I will not mention his name. and the fear of the doctor was that if he didn’t give the blood transfusion and the boy died and the postmortem was done on him it will show that due to blood shortage the boy died, the blame will be on the doctors so they have to do that and the boy survived but they didn’t tell the parents. So its not about your personal belief and wants but its about the society that you are in. And so medically if the blood is not administered the person will die. And can you doctors be persecuted because you allow the person to die [doctors replied…yes]. So these are the legal things that we have to look at and I don’t know about stroke.

I Whiles we are talking about blood donation for transfusion, what are your thoughts on blood donated for research purposes?

R1 It is laudable.

R2 It is beneficial and fine so I don’t see why it should not be done. Once its done safely.

I Are there any cultural, are there any legal issues and are there any social issues to blood donation?

R It is important to understand that the highest law in the country is the constitution and it guarantees certain things we call them rights for people and usually we don’t allow parents to make matters out of their children and the child may grow and become a chief Imam or a Catholic priest or a Presbyterian Moderator. So under the constitution, anyone under the age of 18 years, the constitution will not say that if there is a life threatening situation, and there is a life to save, you cannot say that on religious grounds alone, they should deny that. No, they cannot do that and the parents in this country do not have the right to do that. But the point that I am making is that some of us are very passionate about research in general and especially research in relation to the topic that we are discussing right now. But we need to understand the context in which we are. For example my village people when you are in the community taking blood, they will say that no and that you are going to use the money for juju (rituals) to go and make money. The time that I was doing my research, a lot of things surprised me and stories are common and one of it is that body parts being removed at mortuary for black money or juju. So we need to be very careful whiles we are happy with the advantages, and the prospects of the common good of tissues and organ donation in general including blood donation can do to us as a people. We must be aware of our cultural myths and beliefs, that may act as stumbling blocks and one of them is the whole concept of syphoning blood from people and use them for juju. We will require targeted approach and like medicine is specialized in different areas, Journalism must be specialized into different areas so then we will have those who are specialized in health research and then they will report consistently and accurately so that they tell the message the way it should be to avoid hearsay. Because one thing in this country is to allow false information to go ahead of you then you are finished. And that is why I used my village, we have two radio stations there. And once the message goes to them that we are siphoning blood then no amount of excellent scientific information will cut their eyes. And so whiles we are actually happy with that, we have to recognize who we are and the community and ensure we get a strong advocacy and media engagement and good communication strategy to go with the good message so that when the falsehood comes then they will say we have been told of the good news already so that is a challenges we may face and we may have to put our heads to it whiles we are looking at the good.

R2 Then Doctor, add to it that the media should advance knowledge with the community before the practice is done. Because if you don’t tell the community about the benefits, then they will not accept it.

R3 yes the honors is on us as the custodian of the technical knowledge to break it with them and that is why I asked for this continuous engagement with select media who will understand and non that today we have one reporter and tomorrow another reporter so then my colleague has to repeat then it becomes difficult. So once we have an effective communication strategy which includes this kind of partnership then I think that it is something that a lot of Ghanaians look at. To be honest with you, if you take out these myths things that I have raised, the foundational ethos of the Ghanaian community be it Ewe, Dagomba, Fante, it is collected solidarity.

I Thank you very much and I am enjoying the discussion. Now lets move out of the community and get a bot personal. Will you as an individual here be willing to get involved in such research? Whiles thinking of the answers you could also think of the possible barriers that you could encounter if you should decide that I want to get involved in either blood donation for research or body part donation for research. What are the barriers that you are likely to face and who are the significant others that you may have to consult?

R Do you want the answers today [Laughter by all participants]

R2 I will be very happy to take part in such research. But I will also want to be assured that after such research I will not suffer any side effects and that is going to affect me for the rest of my life. If for instance I am to give blood and I am going to suffer some discomfort. For instance the prick, little bit of dizziness, nausea, for which I am assured that it will last for maybe 3 days, I will do that so that is the extend that I will participate in the research.

R3 And other than that?

R4 So why will I donate blood and not be able to walk back home [Laugher by respondent]

R5 The second part of the question that which significant others will I consult and that is why I ask do we have to do that now or later because if I have to consult my wife she is not here now.

I We are not taking the action now and I asked whom are you likely to consult and what are the perceived barriers that you are likely to face?

R1 for procedure like donating blood I am not going to consult anybody. I am going to do it myself but if I had to give my kidney to a member of the family I will consult my husband because he is likely to suffer some of the consequences of my action.

R2 From what she said, it shows that in the consent form you need to ensure that every details that the participant needs to know and to understand in terms of the risk and all that needs to be clearly spelt out there and the person understands it before going ahead. If you hide some information then that is where some problems arises and if one person encounters one problem, that is where the others will not want to partake in the research. So the consent form that you shared with us is it for us? [Interviewer said yes]. So is it that because there are no risks associated with the study that is why you didn’t mention that on the consent form. [Interviewer said for this particular FGD there is no risk associated] Then do you think that it will be necessary to just put it there that you don’t foresee any risk associated with this research.

I It is interesting how the discussion dovetails into other issues. She has just mentioned the issue of consent and that is where I am coming to. You people are just wonderful. What is your understanding of informed consent and I am sure we all in one way or the other we have obtained or given an informed consent. What is your understanding when they say a consent is informed and what are the processes?

R Meaning that it has been explained to me what I am about to do, the likely side effects of what I am coming to do and that I will not blame anybody for anything that comes out of what I am going to do, I take responsibility and I agree to it totally.

I Thank you and any other opinion on informed consent and we are looking at the processes, and we are looking at the types if there are any and if you know of the types of consent.

R1 Its all about taking responsibility for the action and the participants needs to know that it’s a voluntary activity and the information given should be confidential and I am assured that once i turn my back nobody has access to my information so for the informed consent the individual must understand what they are going to do, the process they are going to go through, their role in that process and what is required of them and that tis voluntary, confidential and whatever you will say will not be of public hearing. Also the benefits are outlined and if there are any risk too you are told. Sometimes you can start and along the line you can say that you are not interested in the research and you are allowed to move out. And usually in the clinical settings, we go to the ward for the usual treatment and they ask for your consent for a study and if I don’t feel like participating in the study, it does affect the treatment that you are going to receive and participating in research is voluntary.

R2 Just imagine what happened about our data with the Electoral commission and other institutions about three weeks ago. We were asked for our consent and all consented and got registered and got our Ghana card but a month ago we heard that our data was being sold out to a banking institution so do we need further consultation and I am asking this because we can personally consent to this and later redraw but you should be convinced that if you give your personal consent there shouldn’t be an risk in future or our data should not be used for any other thing.

R The informed side the information must be very clear and the person receiving the information must also be sober at the time of receiving the information in order to make a sober decision.

R Before you tempt me into the types of consent I think it is better to cast on what has been discussing by others here. To be responsible for a decision means that you must be capacitors and competent. So the first step to get to understand whether this information is informed or not is the first whether the person is sober or not. So if you are not an adult then the processes are different and if you are an adult then whether you sober at that time or you are not. So if you were 18 years and above and your mindset was not destructed by diseases or drugs and you had clarity then you are in the position to give your consent. The second point is that the information that you are going to give has to be adequate, relevant to the specific intervention you are going to make and must be given in the language that the person understands. And all the options including the options of doing nothing must be provided and unfortunately for us there is a law that was passed in 1932 and its still valid for us in Ghana and we don’t look at it. So like if you went to consent my Auntie who is in the village who doesn’t speak Twi and English and the interviewer procures informed consent from her, it will surely be impossible unless he can show that there was interpretation by someone who speaks the language. And the last point is that it has to be on the persons own voluntary and these three has to come together before we can say that technically there was an informed consent given.

And for the types of consent I know you have all been to the hospital and you might have a sore throat and its painful and all that and after the story you have told the doctor, he will say he needs to look at your mouth and he will say its going to be a bit uncomfortable and I will be using this wooden spatula and other funny things and that is why people don’t like coming to the hospital. So in that case you don’t ask them to bring a form like you have done here because it is not pragmatic and not the order of the day. But you saying “oh yes”, you then have expressed your consent and it might be by speaking, or by writing. So then we have two. But if you are not sober then you will not be in the position to express and you have to get a third party to presume that if you were sober, this is what you would have wished so in that case you will have presumed so we have the expressed or presumed. And this covers with the Public Health Physician and they are here and in those days in Ghana Health Service when we were growing up they were doing vaccination and you will go and get injected in your buttocks. There was one long queue and everyone goes and gets injected and after one hour its my turn and I remember that the interviewer said I need to ask for this consent before participating and so I can sue them but then by your conduct you have consented. Because how can someone who is sober see a long queue and just join for injection. So there are others and this is a complex situation. So there are more than that.

R But I have a case with this last submission and lets look at the case of Dr. Jones in Jones Town at America in several years ago. The people consented to his drink and others refused but they were forced to participate and if they were all to be alive, they will sue him.

R Another point is that because we said there are three things that has to happen thus capacity, the information has to be adequate and relevant, and it has to be free. In Philosophy, in bioethics it cannot be free when it is forced and so when you deceive me it is no longer free, when you misrepresent to me it’s no more free and if she is giving consent for only the blood to the used and check on her HP, then he cannot use part of her blood for genetic research.

I You people are phenomenal and he has just dealt into what I am going to talk about. Then we are looking at, I come to you I take blood and I said with this blood I want to do A, B, C then 10 years later I realize I could do more with your blood, where do we stand? Do I need to come back to you and ask your permission and say ok I took your blood 10 years ago and I said I was going to check HP and now I want to do HIV. So we are going to the types of consent where you could have the broad or generic, restricted, or a tiered consent where its in graduations. And with the internet these days we have what we call the dynamic consent where you are in touch with those who have your samples and at any time you can interact with them and update them as to whether you want to be part of any study that they have or not. Now looking at these types which one will you want to have thus giving a restricted consent, or a dynamic consent or a broad generic consent and whatever you want to go and use my blood for go and use. So what are your personal views on that?

R I think this depends on the nature of the research and you cannot give the blood for HP and later say you want to check for HIV. And when you do any small thing and it spreads in the community then people will start that this team that is how they are. So we need to be specific and the society they have a different mindset for medical issues. And now there is Sakawa in the system and everyone is afraid of those things so you need to be restricted. So I will go for restricted consent.

I If you are told that this blood will be used for research purposes, will you be comfortable with that and if it’s a genetic research in stroke will that be enough to give a general consent?

R1 Yes

R2 I will give a restricted consent and want to know what the sample will we used for.

R3 I will like the dynamic one so that anytime you need to use my samples then you contact me and ask so if its ok then I give the consent.

R4 So I need to understand what it is going to be used for and I think recently peace FM did blood donation and we heard that the owner of Peace FM was using people’s blood for wealth and other things. But the information that I got was that he made arrangement with the blood bank and they came and did the donation themselves and after the donation they took the blood to the blood bank but that was the perception of the general public. So I think it will be prudent that if I donate my blood whether for research or learning it should be consented that this blood will not be used for anything apart from what we have agreed for it to be used for the right purpose.

I What is your opinion on storage of blood for future use?

R I think they should inform me on that before they do it.

I Anyone with a different opinion than hers or you consent to that?

R Yes I consent to that but like I said if I go in for dynamic consent then its for future use and it should be used as such.

R And that is where the education comes in and for what use? To sell or for other things so information is very important for the person to be aware that in future it will be used for such and such research.

R How about the safety of the sample because if after three years there is domsor (power outages) and the samples goes useless and I get to know [laughter by respondent] then next time I will think otherwise so the safety of the sample is paramount. Because that can boost the confidence of the donor or deem his confidence.

R On the lighter side, the point raised is like bank of Ghana is on fire because if your money is there you have banked, so it tells you that if all agree that the future care and the future wellbeing is here in this bank, then it behooves us as a committee of nations as a people to put a lot of resources to ensure that preservation we don’t lose that results which is something that whether we like it or not as we are looking at creating a bank of valuable resources, the value they have for today and tomorrow’s generations, is such that we must put in a lot of resources to protect them. But I noticed that you said what if they use it for a different thing, I know we haven’t touched on them directly. But whose money is it? Is a question operating at the back of our mind where we have the bank.

And that is one of the things and we started by saying genes research is in families and race so whiles you are actually doing genes research you find out that is it my body or our bodies, your genes or our genes, the communities genes or the race genes and so this is where you have to be careful of ethical, social, legal issues overlap and generate very uncomfortable questions. So once they are operating or using it and if it’s a family good. Do you have more of a right to it than I do? Who can consent or authorize its utilization or subsequent utilization and if it generates benefits, who should the benefits be lured to. Is it any member of my family who can get that and if we should keep these human samples, should we get to the extent of allowing people to sell it? Should we commodify it or should we commercialize it? Or should we even incentivize it for scientific research? So these are some of the questions that are operating at the back of our minds if we think of what if someone is commercializing it. If in your subconscious you are not thinking that it is yours, then you will not be worried about they selling and not telling you. If it is everybody’s money at the bank of Ghana, then you shouldn’t worry. But if it is my money at the commercial bank, then I will ask for the accounts.

I As you know science research involves a lot of collaboration so what if there is collaboration and the need of sharing data and I am sitting at Ghana and working and I have collaborators in US, UK, Nigeria. What is your thought on sharing of these bio data, blood and blood fractions, brain images like CT scans, MRI as well as brain tissue samples in respect to research. What are your thoughts on sharing these bio data from the bio bank?

R Well I don’t have any reservation about it and such information can be shared but in relation to that as well will it be shared in a manner that will involve you saying this is my blood which contains something that I don’t want anyone to know and is it going to be shared in that manner? If it is going to be shared without my identity that that shouldn’t be a problem.

R let me complicate the issue and complete the question. Sharing data is one leg. To be able to share the data you need to share the tissue as well which has a different character. So sharing data is a different and sharing tissue is also different. So if it was the tissue that someone is sitting in China, UK and is sharing the data with.

R For me I don’t have a problem with the data and tissue or sample being shared.

R Besides that I know that sometimes people have power play and you can take the data from Ghana and share it somewhere and they take the data and make it his own and eclipse your contributions and if that happens its that person who has given your power out and all the sweat has achieved nothing. So I don’t know if we can check that transmission of information and it all comes to money. You have given this information and I have done this research and this is coming from Ghana but I have the money and I have produced the medicine that can counteract a particular presentation of these unwellness. So with this medicine my sample used will not be identified to it.

R So I want to ask a question, when you share these information and other get it is it for fame or you just share these information for advancement of medicine? Because you know of Nana Drobo of blessed memory when he said he had discovered medicine for HIV and he was invited outside the country and you all know of what happened. So was it for money purposes that he was invited or for fame. And if it was for advancement of medicine to cure HIV then why must we bother.

R so for the purpose of having a bank, remember that you donate for a number of things. First is to advance knowledge, and you may donate not to advance knowledge but to teach, and bring others on board. You may also donate so that it can cure somebody and also you may donate to find the sure and get money. So at least for us the general rule is that you donate for advancement of medicine, science in general and therapy and for teaching. But also for policy and decision making, once it’s a bank for communities, nations then its population information and it allows you the amount of information to gather about the community, nations and races and to find the common things that leads to ill health or un wellness and that amount to a lot of it. Then part of it may appear in academic writing because we feel we have found something and we should share that knowledge within academic that’s why we publish in papers. And when that happens you have to acknowledge the people involved and there is a strict rule now that if you are coming to publish papers and it involved collaboration, they need to see all the contributors of the others.

I The discussion is getting interesting and we need to be mindful of the time. So you have taken these samples and what are your thoughts on how individual samples should be returned? So we have collected various samples and we have issues with individual samples which we think you need to know. How do you want these information returned to you and in what form? Do you want a face-to-face interaction with your doctor, do you want results sent by email, or news given to you on the phone, how do you want the information given to you?

R It will depend on the form of information and for instance if you are going to tell me that when you took my blood you found out that I have malaria so I should go and buy drugs and treat it then that is fine on phone. But if you are going to tell me that you found HIV in my blood then you need to tell me face-to-face.

R Because I have been in a similar situation and I lived in the UK for some time and as soon as I got to immigration, then they will send your address to NHIS and they will call you to come and do a test. I have forgotten the name. [Another respondent interjects….Its RDT. If you are coming from African they will tell you to come and do RDT to check if you have malaria.] so when they called me and I went they took my blood sample and then the nurse said that she has drawn too much blood and can she test for HIV and I said ok and she said if anything she will call me to come for the results. So she called me and said the test didn’t show anything and that was by phone but they could write to me with my results.

R This day that technology is advanced we can use face-to-face though but we can also use video conferencing like Skype call or whatsApp video and we can look at all these things.

R These things have bottle necks and I will prefer face-to-face.

R I think it should be on the person’s preference and whether the person wants to see you face-to-face, or through text message or whatsApp message and that is what should be done.

I Somebody mentioned bio rights. What do we understand by bio rights?

R I don’t have an idea and its too technical.

R I think the issues of bio right started around 1980 or so and these human right thing is becoming a problem and so who is a human being. And so I may be a human being and I may want to disassociate myself from certain facts of me and this is what the bio rights things comes in and so any living samples collected from me I have the right to say this should be done with it and this should not be done with it. So if I have the bio right that my eye can be plug out to do something when I have a problem but only one eye. Or even if you want this precision one.

I Alright thank you very much. Are there any recommendations or concerns on using these body tissues for research in Ghana which you will want to talk about but we haven’t mentioned it so far?

R No please

R No please

R No please

R No please

R Its the safety of preservation because if we want to preserve things then we need to get all our power sources and even solar before we start.

R No please

R Maybe to recap the things that we have talked about and currently there is a big challenge in terms of law, policy and oversight and these are the three things of research involving human subjects in the country and these are the things that we hope that as part of the engagement, we hope to become advocates to ensure that at the end of the day we have touched the system in a more sustainable way.

I What suggestions do you have that can help raise awareness and improve attitudes of people in our communities towards blood sample and tissue donation for research?

R We have to start with education and the stroke association has been running lots of education programs in the country and we have been educating people on the risk factors on stroke and it’s a challenge and we tell them of the risk factors in family lines and alcoholism can lead to stroke. So there should be research in stroke and we need to intensify the education. So education is good to the communities to raise awareness.

R use of mobile vans to raise awareness on stroke.

R The use of radio and a lot of people listen to the radio a lot and what they hear to them is the truth.

R I will say targeting specific messages for specific audience so that if the message is meant for the youth then you are looking more online and social media and if it is for people who are not more educated them you are looking at using drama so that you can get the message down to the people that you want.

I I will like to say a big thank you to you all and its been a great discussion and I am sure you have learnt something from it.

End of discussion.
