# Supplementary material for: Research participants’ perception of ethical issues in stroke genomics and neurobiobanking research in Africa
Source: PLoS One. 2025 May 6;20(5):e0292906. doi: 10.1371/journal.pone.0292906 (PMC12054916; doi:10.1371/journal.pone.0292906)
Supplement: S3 File — (ZIP) [file pone.0292906.s003.zip › Files for PLOS ONE - updated March 2025/FGD _Stroke Caregivers_ Abeokuta.docx]

**FGD- CAREGIVER ABEOKUTA**

**1. Tell us what you know about genetic research**

06- I have heard about it before, it is all about checking the relationship of some people.

04- In the hospitals, we do hear about gene, that is relationship. Maybe in a particular family they detected that whenever they give birth, the child gives up immediately. So, when it becomes rampart they may find the cause.

01-What I know is that during the time of our fathers they used to have a lot stillbirth and they always give concoction. But now things have changed, we have been enlightened. If such a thing happened they go for blood test to check the compatibility of the father and mother.

03- Genetic research is all about some issues that are re-occurring, just like something that happen to somebody and re-occur in another generation. For example, in some families they die before a certain age, they don’t exceed that age and that is peculiar to genes in family.

07- Most families when things are happening to them they will begin to think about it and link it with other occurrences in the family. In the olden days when a woman has miscarriage they attribute it to witches without knowing it is caused by disease condition in the woman. But now due to research, such woman’s medical history will be traced. She may be put on bed rest for some time to save the pregnancy till she gives birth. She may likely overcome the problem and have safe delivery.

Also, on the issue of sickness of the body, some always die anytime they attain a particular age, for example 21 years. Later it was revealed through research that if husband and wife’s genotype are AS, it is most likely that they give birth to a child with sickle cell. Such child/children don’t last no matter how much they spend on them, they die eventually. Now, people take caution and make necessary findings before they fall in love. They both go for blood test to know their blood group and genotype

05- I have heard about it, my father made it clear to me that arthritis is mostly inherited. Anybody that has arthritis in those days can’t come out during the rainy season. Nowadays there have been a lot of researches about it and there are drugs for people with arthritis.

02- When someone has a particular sickness, the family history will be checked to know if the sickness has happened in the family before or it is just happening. If it has been happening, then the sickness will be established to be peculiar to that family. If not, it will be seen as sickness that just occur.

**2. Is there anyone in this gathering that can share his/her experience or that of another person he/she knows who has participated in genetic research?**

04- During the time my husband was hospitalised, when we got to Sacred Heart Hospital, Lantoro, all the doctors examined him. Then, they asked me if I have the idea of what happened to my husband. I told them that I believed it is stress which can cause stroke. They said apart from stress what else do I think can cause his stroke. I told them that my husband told me a story that his father who was a transporter was set to travel to Lagos one day when he started feeling a terrible headache. They told him not to travel again that he should relax. He sat back on a chair and relaxed. Later, he complained of having chest pain and leaned on the chair he sat on, that was how he died. So, when my husband fall sick and they said we should go for CT scan, we did the scan and the doctor was asking my husband about his father. When my son came to the hospital the doctor told him to always check his BP and that he must exercise all the time, avoid alcohol and cigarette. This is how we got to know that stroke is genetic.

02- Someone very closed to me, when she married; neither she nor her husband did blood test. She had two children; one of them has sickle cell. They took a very good care of the chid but lost the child. So, she divorced her husband. When she wanted to remarry, she went for blood test with the man, and they realised they are both AS. She said what happened to her the first-time should not repeat itself. She didn’t marry the man. That is all I can say about it.

**3. What do you know about stroke genetic research?**

03- That is tracing the root causes of stroke with the person’s relation, father, mother, brother, uncle whether they have similar problem, to trace the root cause where it started.

06- When we look at stroke, the major cause is high blood pressure. Considering high blood pressure, it is hereditary. That is why in a family those who have high blood pressure may be many, and high blood pressure is the major cause of stroke. So, there is every possibility that majority of people in the family may have this disease.

01-It is not all stroke disease that is hereditary. Some may be due to our unrest and strenuous lifestyle. Something happened to my husband, it is exactly 3 years, he and his friend did a business, and the friend cheated him. Due to this, he could not sleep most night and it led to stroke. We brought him here from FMC. From here he was referred to UCH. UCH was the fourth hospital we took him to. No one ever had stroke in his family. It was lack of sleep and excessive thinking of how his friend cheated him that caused it. It is not every stroke case that is hereditary. Excessive thinking caused by action or behaviour of someone close to us can cause it. It can be between husband and wife if one cheated on the other, and this can cause sickness for the other.

**4. What are the roles or benefits of genetic research in medicine?**

03- It will make people to avoid something that could cause stroke. Researchers will be able to trace the root cause of stroke.

06- When talking about genetic research, it has given us a lot of benefits. For example, a family that has rheumatism, it is only this test that can help to know what they can use to prevent the re- occurrence of this disease. I think that is what genetic research is meant for, and that means when we do this type of test it will stop the re-occurring of some ailments that may cause premature death or any other thing in the family.

05- The benefit that is there that I noticed as a caregiver is that I normally tell people is that we should always take things easy. It happened to my brother, his wife divorced him. Later, she came back to the area and rented an apartment. Three (3) months after that my brother was down with stroke. The thought of what his wife did caused the disease. What I am trying to say is that if we have anyone going through such thing, we should always be there for them so that they will not develop high blood pressure and have stroke.

02- There are many benefits, if for instance someone had stroke and got treated, it will prevent it from happening to other people because what caused the sickness for that person would have been known to others. Most times, stroke is caused by carelessness. If you are warned to take things easy and you decided to stress yourself the more it can lead to stroke. When people have this knowledge they will learn from other person’s carelessness.

07- The benefits are numerous, by the time people around the stroke patient see the person and the outcome of what happened to the person they will try not to have the same problem. To be sincere, it is not easy to care for a stroke patient. The care giver of a stroke patient is doing a great job. Stroke patient can’t but have a deep thought because he/she never wanted the disease and he/she does not like the condition he/she is. The caregiver has to be patience with him/her. Most of the stroke cases are caused by carelessness and stubbornness of the person on drug medication of other diseases. You have to accept him/her the way he/she is and have a lot of patience.

5. **Can you explain what you understand by bio-banking?**

06- I have heard about it but I don’t know much it. I first heard it from Dr. Tai Solarin, he said that time that when he died some of his body parts should be removed and taken to UCH for keep so that people that need it in the nearest future can have it and his carcass be buried somewhere else. That was the first time I heard about it but I didn’t take it upon myself to go for research. I know parts of the body like the kidney, lungs and other vital parts of the body are useful and people do say that upon their death they should remove it and keep for people that may be in need in the nearest future.

04- Even though I have heard it and watched it on the television, there was a program on CNN where a lady was about giving birth, she was operated on and she had twins. In the programme, they removed something from the woman’s body to the babies. I could not watch the programme to the end because it was scaring. When my child came home I told her about it and she said it is possible to transplant organs. She said some people voluntarily donate their organ. She said we should always pray that we will not have cause to need transplant of organ. She said we should be careful and watchful of our intakes, that fruit is very important in the body unlike carbonated drinks like coke and the rest. That is how I got to know that it is possible to transplant.

**6. How does bio-banking operate?**

04- I have never heard of banking parts of the body but I know about blood. There was a group or maybe association in Abeokuta that always talk about blood, that people should come and donate blood. My friend’s husband always donate blood freely, here in Abeokuta but I can’t remember the location. That is all I heard about it.

**7. How important is bio-banking to medical breakthroughs?**

07- Bio banking helps the treatment of patients. For instance, when I was about giving birth to my last child, I went to hospital that day they gave me two (2) weeks as next appointment date. When I got back to the house I went into labour. I just came back from the hospital what will I do. I packed my bag and gave it to my older child to go ahead of me that we will meet at the junction and board a taxi. I didn’t want people to know but I could not leave the house. It was hard for me to move. I helped myself and delivered the baby. Our tenant came and asked me if the cry she heard was of a baby I said yes, but the placenta is yet to come out. I told her to go to the church and called my mother. The placenta eventually came out but I was bleeding profusely. Then, there was no phone to make call and my husband was not at home. As God would have it he came home and I was taken to the hospital, the same hospital I went in the morning that they said I should come back in two weeks’ time. I lost a lot of blood. I could not walk again. What I want to point out is that when I got to the hospital, they said I would need blood that they should bring people that will donate for me. Before those people came they have gone to blood bank to bring blood and transfused me. If they didn’t have blood bank and have blood kept I may not be able to stay long until people that wanted to donate for me came, another thing might have happen. I am still alive. Blood transfusion saves life.

06- Keeping organs help us, but unfortunately I don’t think it is rampart in Nigeria. For example, four years ago I lost somebody. He had kidney problem. There was no match for him, no donor matches his own. Unfortunately, he died the very week they finally got a match for him. Had it been that there are organ banks in Nigeria, maybe he wouldn’t have died. Maybe they would have got a match for him and did the transplant and he would be alive today but no match for him and he died.

02- Just like our mummy said earlier, especially for women when we are in labour, some may need drip or blood. Before they can get it for use, they would have use from the ones they keep and when the family of the pregnant woman come with the one they went to buy they keep it again for another person.

**8. What is/are people’s belief, thought and opinion relating to bio-banking?**

05- The belief of people about bio-banking is that when they have people having related problem, they easily help such person and the person will not die

06- It is like in our environment we don’t have the knowledge yet. If we have the knowledge we won’t perceive it as something serious. As I said about Dr. Tai Solarin, it was because he was educated, enlightened and informed. He voluntarily said they should remove vital organs from him for keep so that they can use it for other people in need of it. Our people believe in returning back to God the same way they came. Nothing should be removed from their body. It is because we don’t have the knowledge yet, if we have the knowledge we will know that whatever is being removed from our body is beneficial to some other people especially those that are not old enough to die but are in need an organ for survival. If we continue to work on it, science will explain it better to us. Atimes when someone is at the point of death if vital organs are taken from the body, it will help to prevent premature death.

04- May the Lord have mercy on us, these things we are talking about is not impossible. There are people that voluntarily donate organ especially kidney, even when they are still alive, our people condemn it because we don’t have the knowledge. I think that to make our people have the knowledge, Federal Government should make public enlightenment about things that will be beneficial. Let people know that at death they can still be useful to people. I have a grandfather that has died; he voluntarily donated some of his organs before he passed away. The person that benefitted from it is in Lagos. I believe that if our people are enlightened it will help. At least some people willingly donate blood. Public enlightenment will help us in this country.

03- Bio-banking is good in the sense that it prevents premature death. I don’t know why people drag their feet when it comes to helping people. The dead is already gone. What is the benefit of that part of the body if it does not save life? But some do not believe in it. I believe in it.

**9. What is your understanding about brain banking?**

06- I have never heard about brain banking

07- I have never heard about it

05- I have never heard about it

04- I watched it in a movie, how they do brain surgery. They removed the skull. I saw it in the movie how they removed a part of the skull. They said it cracked and reset it. That is all I saw, after the surgery, they pass oxygen for the person.

03- No, I have never heard about it.

**10. Awareness of any policy or law guiding bio-banking?**

04- There are laws guiding bio-banking. If anyone wants to donate, let say blood, the blood will go through thorough screening to be sure that it matches that of the person that needs it. For example my son is (0-) SO negative he is a donor and as a donor he can donate to anybody but he can’t take from everybody except (O-) O negative like himself. I got to know this when he was 3 years old. He was sick and we took him to hospital where it was pronounced that he had typhoid fever. I was so afraid. At the end of the day, we were told to donate blood for him. That was when we realised he is O negative. His daddy is B negative while I am O positive. We could not donate for him. They brought blood from blood bank for him. Later, one of my younger ones came and he donated blood. They replaced it with the one they brought from the blood bank. Before they took the blood from him, they did series of test for him to be sure there is nothing wrong with the blood they want to take.

06- In this country, everything is law. If there is/are no law(s) guiding us in keeping things or law guiding the use of stored materials, if anythingnhappens, it is the same law that will be used to support the action. Definitely, it is important to have a law.

02- There is law, in the sense that there are different diseases in our body. Some people are HIV positive and whatever blood group these set of people may belong to, their blood cannot be transfused. It cannot be stored in the blood bank because it has been contaminated. Blood can only be taken from people with clean and pure blood. That is part of the law they follow.

**11. Can you explain what you understand by precision medicine?**

06- If I get you right, it is like when someone has headache and goes to the hospital to see a doctor, it is that person the doctor will attend to and prescribe a drug for, not somebody else. Is not that when he/she gets home and a neighbour complains to her of headache and he/she gives the drug in the hospital to the neighbour. That is no more precision medicine. Headache can mean so many things to many people. For example, in my case, when I have headache, I may be hungry, I may be pressed to use the rest room or it may be lack of sleep. If it is not of these three, then it is serious. Receiving or using someone else’s drug is wrong.

07- I don’t have another thing to say from what she said. I have been tested, they know what is wrong with me and they know the drug to give me for use. For example, as at now, I have arthritis and it is very painful. I have a drug I use, let say a sister comes to visit me and complain of knee pain, and I give her the drug I use for arthritis, it is possible the drug will not take care of her pain because her pain may not be because of arthritis. The drug has become a waste.

02- To add to what they told us, the person that went to see the doctor may be told to take a tablet per day, the other one that is been given the drug to use may take more than the prescription. That is what I want to add to it.

04- When someone goes to the hospital, the doctor will prescribe drugs. I have arthritis I went to the hospital and the doctor prescribed drugs for me and that is what I have been using. Sometimes ago a doctor came to our church to educate us about arthritis, people that have arthritis expected the doctor to prescribe drugs for them. The doctor only told us to add ginger and garlic to our food that he does not have the right to prescribe; only our doctor that we have been seeing can do that. Later a grandma came to ask me the drugs I use, I told her I did test before my doctor prescribe the drug that her children should take her to see a doctor, they later took her to Lantoro. She did just five thousand naira test and the doctor prescribed drugs for her. Today the grandma has improved better than me. The doctor said it is because I always put hands inside water. Since my husband’s sickness has started, I always put hands inside water because I wash clothes all the time. The doctor said I should massage it every night but now my son have bought gloves for me that I use.

01- As we gave individual consultation in the hospital so also we have it at home. We have a tree called Iyeye tree, if one has hypertension and the person takes Iyeye and licks it, the hypertension will be suppressed immediately. So, the way I drank from the leave and it heals me is not the same way it will work for another person. Some people if they have headache, it might be only water that the person will take and will be alright, to some it might be paracetamol, some panadol extra, so it is not what I use that works for me can work for another person at the same time.

**12. Benefits and demerits of precision medicine**

05- The benefit is that if you see a doctor and you are examined, the test will tell you which medications will work for you. If another person uses the same medicine that is given to that person even if they are nursing the same illness, the medicine may not work for that other person

06- The benefits cannot be quantified because I visited my doctor I have spoken with him and he has given me drugs, so if I now give it to another person, I have abuse the drug and it could cause another ailment in another person’s body.

04- The advantage as our mummy have said is that, doctor knows individual ailment and if it happens that there are lot of people to see the doctor and it is not possible for you to see your doctor you can as well see another doctor because you have a record already. Since the doctor who is seeing that person can examine and give you feedback on your health condition. The worst thing about seeing a doctor is to buy your medicine again, and it may be only two weeks before the doctor wants you to use the medicine, which is prescribed but if the person has a medical condition, it is the doctor who will prescribe the drug.

**13. Is precision medicine** **important in Africa?**

06- It is important because it is the illness that pertains to that person is what they will treat the person for. Sometimes, they will ask us to check our weight and height; these parameters are used to prescribe drugs. Now if my weight is much they are going to prescribe the drugs base on my weight. If someone with low weight takes the drugs meant for bigger weight, there will be problem in the body system. Precision medicine is very important because those are the things the doctors will look into before prescribing drugs to a patient.

04- Precision medicine treatment can save but what we are pleading for is that there should be many doctors. If we have about thirty patients to be attended to by the doctors, and it is compulsory to ask questions relating to the person ailment so as to know the type of drugs to be prescribed. They must not be in a hurry to consult patient, since we have admitted that precision medicine is important and if we have limited doctors to attend to individual patient that can be risky.

01 - Very important to all of us

**14. Can precision medicine** **be applied to stroke?**

07- Precision medicine treatment can be used for stroke if the person is on medication and is using it on a regular basis.

06- Precision medicine treatment can be used to treat stroke. It is important for the treatment of stroke because there are different causes for the disease. For someone it is high blood pressure, another person could be depression, some hereditary and the medication is different for one person to the other. Let me use my mother as an example, she has hypertension and as it increases they increase the dosage of the drug and at some point the drugs was reduced. If not that we usually come on a regular basis to see our doctors and that they are taking care of us and if we had refused to see the doctor and she was not well taken care of or monitored mama might have gone.

05- We can use precision medicine for stroke disease it is very good, like my uncle that has stroke that we brought here, we came to the clinic and we attended physio we can see now that he is improving. We usually come on a weekly basis, later they gave us appointment for two weeks, now we are coming on a monthly basis. We usually bring him for physio and do it for him at home as well.

04- Can be used for stroke disease. Atimes, they may not want to listen to us (caregiver) but by the time we make them know that they will soon see their doctor and by the time doctor sees that they are not improving that the doctor will complain, they tend to listen. By the time they get to the doctor, they always cooperate which is a plus to precision medicine. The area of complaint is that there should be a place where physio can be done apart from coming to the hospital.

03-It can be applied to stroke because stroke doesn't just happen, something will lead to it. The doctors that prescribing the drugs are experts that know what must have led to stroke. They are the only one that can tell you what to use to eradicate the disease.

02- It can be used for the treatment of stroke because if we have 10 people with stroke, the ways in which they will be treated will vary, and the drug will also be different.

01- Can be used for stroke treatment, and the doctor treating them should follow that procedure

**15. Are you aware of any policy or law guiding precision medicine?**

06 - Available but not common in Nigeria.

**16. What do you understand by brain donation for research?**

06- I've heard that people donate their brain for research.

04- I have heard of body part. I have heard heart transplants taken from the hospital for medical doctors. I have heard of heart but I have never heard of brain donation.

**17. Benefit of brain donation**

06- It has benefit. The research is that, let say someone has mental disorder or stroke because stroke also has to do with the brain. A research will be done to know what really happen to the brain. There are benefits. What I also found out was that we should legislate it, we came to the conclusion that legislation does not exist because of the understanding we have, and that in this part of the world we don't read. If we read properly we will know that something like that do exist. The way our country is does not give room for people to search for knowledge.

04- I want to make a small contribution about the benefit of getting a brain donation. I went to a psychiatry hospital and I met a woman that I know before and she informed me that she came for treatment. What I know about the woman is that she has epilepsy, which make her fall, and I think that's how she was diagnosed with the brain problem. When my husband was sick and we went for a CT scan, I was with him, the doctor told me that one vein has cut in the brain, and that my husband was lucky that it wasn’t in the front that that is the reason why he can still remember something but if it was at the front he will not remember anything

**18. Do you think people are willing to donate towards research?**

06- As I said earlier, we need to be enlightened. Our government is not helping us either. If there is proper awareness that if we can release parts of the body for research after death, it will be beneficial for some people than for body (corpse) to be rotten away in the ground, it will be better.

03- I can willingly donate my brain, I don’t know of others

02- People will not be willing to donate for research because of civilization. As our mothers said our civilization is not as good as they do in oversea and we cannot do it here.

**19. Is it our people, relative, family or religion that inhibit people’s willingness to brain donation?**

05- Something I've found out that can inhibit us is fear. Fear can grip people from donating.

**20. Don’t you think our religion has a role to play?**

02- As they said people do fear. If it is about religion, there is a belief that when someone dies, they will need to remove some part of the person’s body though it may not be used for healing. We fear often and people do say that whatever we do today will become history tomorrow. Though they may be abusing their family and refer to the situation which had occurred in the past and there may be something in the future which may cause them to refer to the situation. They can alleged them that the part which was removed was used for rituals

06- Apart from all these things I believe our culture has to do with it. You know that our belief here on earth is to return to God the same way we came, nothing must be removed from me. So when we get oriented we will know that all the things we hold tightly on are nothing.

**21. Share with us your belief and thoughts about blood sample donation for stroke genetic research**

04- I have said it before that blood donation is very important. In some hospitals they need blood before they can revive people. It is very important to donate. Sometimes our people need to be enlightened to endeavour to donate blood whenever the blood is tested and found to be okay. But people have now turn it to business. When they are asked to bring in people who will donate blood they will instruct those people to demand for huge sum of money and if those that need the blood could not pay for the money demanded, they will tell them not to donate. Those middlemen will still demand money from the donor which is very bad.

**22. The blood in question is just a sample to run a test. Can you willingly donate it for stroke genetic research?**

06- I understand well, if they approach me I will willingly donate. I used to have a cousin she is AS and she is married to AS. Her first three children are AA and the next three are AS while the last child is SS. They brought the boy for test, the boy is okay now through blood genetic examination. Then, they told us that it was his bone marrow and everything was tested and replaced at one hospital in Lagos, the boy is okay now.

04- I had voluntarily donated blood when my baby was sick. The baby had high blood pressure. I was tested and daddy was also tested. I do not know that daddy will eventually have stroke

07- I can voluntarily donate blood for research work to help the family and for me also to determine if I have that disease in my body so as to be able to know the solution or treatment. To die is not a problem but for the struggles/pains one will experience before death. Coming to this world is struggle, returning is also a struggle. It will be better for someone to know the way out to any health challenges and allow the person to die in peace. There is nobody who will come to this world and will not die.

05- I can voluntarily donate blood, if I see someone who is ready to collect. I am ready for the benefit of my family in future.

**23. What do you see as the barrier that could hinder your donation of blood sample for stroke genetic research?**

02- There are some who do not want blood transfusion, definitely those category of people we cannot donate for

**24. What are the benefits of willing donation of blood sample for genetic research?**

04- If we voluntarily donate blood we can even know what is in our blood. Besides, we will keep a record of it because there was a time at Akomoje that names were been recorded for those who donate blood just in case of emergency, it is possible for that person to need help in terms of blood in the future.

06- There is a benefit, as I said earlier, I went for check-up because I was feeling pains in my leg, my mother has arthritis. I don’t really know what happen, I just noticed that I have swollen legs. I went for check up to know the cause of the pain because if I know I would be able to take care of my children and my younger ones too, by that we would be reducing that type of sickness within the family and the society at large.

**25. What can you say about your family member or other members of the community's willingness to give blood sample for stroke genetic research?**

06- As I said earlier education, if they educate us, we are only seven here, this thing has to do with millions of people. We should educate them, on television and radio broadcast it, announce it, enlighten the whole community. We will then see that the benefits more than the risk involved, we might respond positively.

07 - in addition to the things that have been said is to invite people to meetings in the church, in the school, in the community, to meet in the community, to have the government enlighten us, and to explain to tell them the benefits that are available there, and the things that people might miss and explain them to us all. We will all have more knowledge about it.

.

01-As pointed out before, we urged our governments to put forth such a plan. Many of our people could not come to the hospital. They should organise a campaign in the market. In past, they bring their motor to the market and doctors do come around at the market and they will tell us what we need to do. We were given leaflet. Not all of us can come to the hospital because some of us cannot do without our market. They should let it circulate.

**26. Tell us what you know about informed consent?**

**--- type of informal consent preferred**

I would like a two-tiled number two

01 - First of all, dynamic

05- tiered

04- dynamic

07- broad

06-broad

**27. Person to be involved before participation**

05- No.

02- Yes

03- Nobody

01- I'll tell my husband

07- My first child

06- My husband and children

04- My children

**28. Support for generic consent for community**

04- We use to have community meetings where all community members sit together on their meeting days. On these days you can send representative to the community to sensitise them.

06- We have ministry of community and social development, you can pass through them, they know where the communities are, they have staff in charge of each community.

**29. What is your opinion on storage of blood sample and blood fraction for genetic research?**

06- We have talked about it, it will make diseases reduced in our society and anything that will reduce illnesses and diseases is good for our society.

02- There are lot of benefits in it because you will have access to it, when such a person is at the verge of death they can save such person

07- We have talked about it before; we have discussed it and said it will help in taking care of sick people so as to receive immediate medical care.

**30. Tell us what you know about sharing of data, blood/blood fractions, brain images (CT scan/MRI) as well as brain tissue samples**

02- There is nothing wrong in it, it will be a reference point in the nearest future.

05- I think it's better to share it.

04- It is good to share.

**31. Share with us your thoughts about return of individual research results and incidental findings**

02- Invite them to the hospital and talk to them, and if there is anyone who needs counselling you can counsel them as well

06- If someone writes a test that person needs to receive result, whether it is good or bad. It is good to get result and take step to correct the anomaly, if there is any. Besides, if the result is good there are some things he will need to keep to.

05- I think I need to be called for the result.

0?- Anyhow, I need to know my result.

04- You know when we are in the class teaching, our students do expect result either bad or good, but we do not expect bad result we expect good result. You can send my result anyhow through anybody.

06- When we are talking, we must not consider ourselves alone we must think about you that want to send the result, those you will inform are many, the way and manner you think will be easy for your organisation in disseminating the information

07- I am not the only one that should receive the result so that it can be beneficial to others

**32. What are the ethical, legal and social issues relating to returning individual research results and incidental findings generated by genetic research?**

02- it depends on how the organisation want it

**33. Explain your understanding of Bio-rights? Do you think there is any law?.**

02- There is

**34. How much control should/can individuals have regarding how their biological specimens will be used in research?**

02- It depend on your agreement before donating. There must be an agreement between the parties involved and that is what the are going to follow.

06- We understand, but it is no something that is common. I think there should be laws which should guide the people because where there is no law there will be no sin. We have said it before that if want things done properly there must be law guiding it.

**35. What right does the donor has over the organ he/she donated?**

07- It is what the owner of the part wants that is should be adhered to, whatever the person says should be followed.

02- Like I have said earlier agreement that is done should be followed.

**36. What is your opinion about governance and regulation of bio-banking?**

02- We were informed before the program started that what we are discussing is not something that is common, we only know about blood donation, for that we cannot say much about it.

**37. Should there be a regulatory board in bio-banking?**

05- We should have it.

01-We should have it.

**38. Explain possible intervention for implementation of bio-banking**

02- They need to constitute a committee that will see to the affairs of what is about to happen.

04- There must be thorough preservation of materials collected from people and we all know power supply is a problem here. To preserve these things we need constant power supply or generator. Body parts collected must be kept in cool area so that it will not spoil. If they collect it and they fail to take care of it it will get spoilt. Blood collection from people is not something difficult at all, but how to take care of it and preserving it is the most important.

06- There should be government policy and regulation.

**39. What suggestions do you have that can help raise awareness and improve attitude towards blood sample or brain donation for research and encourage people to adopt the practice?**

06- My last contribution was government policy and regulation, when there is government policy and regulation and we educate our people, we orientate them through radio, television, sensitisation, within a short time the whole world will know about it.

01- Government has a lot to do about it, it should be publicised on the radio, organise a workshop/training about it, you will see that people will be more enlightened.

**40. Any other major concern or recommendation on use of blood or brain tissue for research in Nigeria and Ghana?**

06- We have said it if we want this study to advance I said we need to legislate it. We need rules regulation, and we need trustworthy people to work with without favouritism. If the Europeans that brought this are not trustworthy they will not be able to improve on it, let us follow their steps in details.

07- We must be proactive because we do not always take things serious when it comes to health issues in Nigeria. Unlike the Europeans they take things serious when it comes to health issues. When we fail in one aspect we do not want to continue because of the failure we encountered. For examples there was a time when they use to arrest those with indecent dress but before we know it, it became the things of the past. Now, children can dress half naked and no one to curb them. If we have laws that we follow, we should not allow ethnicity, religion to dictate how we are to some things so it is better to show interest in this.

04- As we said health is wealth. The first person to be involved in this is our government. Government is the number one on board. The government can help us better, they should set committee for this program, once our people are enlightened through newspaper, television, public awareness in the market they will response. We also beg that all the material donated should be kept properly, we don’t want sentiment.

02- Those who talked before have said it all, those who will work with this project must be people who are knowledgeable about it. I remembered there was a time I went for an interview, questions were set for us and it was those who never passed that were contacted, they just abandoned others and that is what happened everywhere. This should be avoided, those who know about this must be employed to work on this study, if they collect any part of human body it must be handled with care, there is no where that is secure, the security must be tight and there must be preservation of those part of the body collected.
