# Supplementary material for: Research participants’ perception of ethical issues in stroke genomics and neurobiobanking research in Africa
Source: PLoS One. 2025 May 6;20(5):e0292906. doi: 10.1371/journal.pone.0292906 (PMC12054916; doi:10.1371/journal.pone.0292906)
Supplement: S3 File — (ZIP) [file pone.0292906.s003.zip › Files for PLOS ONE - updated March 2025/FGD_ Stroke patients_Abeokuta.docx]

A**BEOKUTA FGD CASE (ENGLISH)**

**Moderator: B**

**NOTE-TAKER: Y**

1. **Tell us what you know about genetic research**

01 – What I can say about genetic research is that it is used to know those things that are genetic in the family, that occur in the family, in some families they are dark in complexion in some het are fair in complexion, at times the father may be short and the mother tall it may be a male child that will take the gene of the mother aand he will be tall, some of the daughters make tall after the father and be short. Also we have gene for fatness and slim frame, likewise eyes ball, nose use to define the family in which someone comes from.

03 – What I know about gene, the way in which I understand it is that for instance a man and a woman want to marry especially in the olden days they use to make findings about the family in which the man or woman want to marry from, even now they still make findings to know the genotype either the man or woman is AA or SS, if their blood is not compatible there is no amount of love they have for each other they have to break the relationship, that is how I understand it.

05 – Gene you are talking about brings about resemblance when they see someone they will say this boy or girl is a child of this person there is a sign they have seen in the father or mother that makes them to know this is their child. Name can be the same, like me my name is Solanke, there are a lot of Solanke in Abeokuta, also in Ijebu. So if you mention Solanke they will ask you if it is Solanke from Ijebu or from Abeokuta, what I am saying in essence is that resemblance, physical appearance will let people know where the person is from, the family the person comes from.

- **Tell us about your experience or other you know have had with participating in genetic research.**

1. I have never participated and I don’t know anyone that have participated before in genetic research
2. I have never participated

All- Never participated

**- What do you know about genetic research in stroke?**

04 – if I understand your question stroke is a disease that happened when you least expected. I as a person the day the stroke occurred, I and a brother were eating pap, I made the pap myself at a point I couldn’t lift the spoon into my mouth again, that was all I know until I was brought to FMC here and when they brought me here they received me and the treatment they gave me helped me a lot and I see a lot of changes till now. That is all I can explain.

02 – My sickness started in the middle of the night and no one ever had it in my family, it happened to me at night and I could not lift my hand and leg, I went to the market that very day, I cooked and ate before going to bed. That is all I can say it has never happened to anyone I know before, my father or mother.

03 – What I can say is that, our belief as Yoruba is that if anyone has a disease in a family it is possible that another person will have it. For instance when I had stroke I was asked if anyone ever had stroke in my family, this means that if anyone has stroke someone must have had it in that family before that is why the person has it. we are made to understand that stroke is genetic and it is hereditary someone can have if somebody have had it before in the family.

06 – From my experience I realized that before stroke would occurred something must first happen, if you realize that you are not having enough sleep let say 2 hours of sleep, know that that thing is coming. Let me use myself as an example, I had a little misunderstanding with my friend, that my friend cheated me, people intervened and advised me to let go but it was hard for me from there I started having sleepless night I used sleeping pills still I could not sleep. I started having serious headache I used paracetamol the headache did not go. I later went to a hospital close to my house from there I was from to FMC, that was all I know, I didn’t know when I was brought to FMC, when I opened my eyes I was in UCH. In short, so that I will not waste our time when you have something bothering you and you can’t sleep it can lead to stroke and it can affect the mouth leg or hand. When I opened my eyes I told them I remembered going to the hospital where I was first admitted by myself, I drove myself there, it happened that I could not lift my hand and leg I wanted to talk I could not open my mouth to talk, but as our God is so powerful by the fourth day I told them myself that I have done in the hospital they should let go home. So from my experience I know that when you put something in mind and the thing is troubling you, you can’t sleep even with sleeping pill with have headache then stroke is at the corner. What surprised me that I want to add to it has to do with what my friend said is that last month my daughter that happen to be my first child had a similar case, I was called around eleven o’clock to come with my car when I got there I realized she could not talk. I said we should take her to the hospital, her case was exactly like mine. By the time we tried to carry her six of us could not lift her hand, when we got here we did series of test, we went to Babcock for another test. She spent a week in FMC here before she was discharged, to the glory of God she is back to work now.

01 –Just like those that spoke before me said, what I first noticed was that I hardly sleep, I didn’t have headache but if I don’t take sleeping pills I don’t sleep. It happened that some people came to our school one day and they checked our BP and they told me that my BP is not too good that I should go and take care of it, he didn’t tell me more than that. Sometimes later my uncle died and he was into thrift collection business, his children then brought his record books to me, I didn’t know anything about thrift but I took my time to study the record for a whole day and a night to write out the names of the people the amount contributed and what they are getting back. I didn’t sleep throughout the night, I still went for early Morning Prayer. As I was going back to my station in the morning on my okada I remembered I was supposed to tell my wife where I kept the keys to my uncle’s house because some people were coming that morning to inspect the house, then I turned back. I got to my wife, I told her what I came to tell her, to now turn my okada and start going back I could not again, my hand became stiff, I could not lift my leg. My wife was busy she turned back because she didn’t hear the sound of the okada, on seeing my condition she called on our neighbors, they came and carry me from the okada because all along I was there on the okada I didn’t fall. Like our daddy said I was conscious but most of the time I couldn’t open my mouth to express even though I had a lot on my mind to say. By the time we started coming to clinic here from the talk they give us here they made us to know that stroke is not infectious, but the patient needs care so that he can recover fast.

- **What do you think are the roles/benefits of genetic research in medicine?**

02 – Since the I had stroke I didn’t go to any traditional healer for care, my children wanted to take me but I said know, Jesus is the ultimate He lives in me and I live in Him. In Him I sleep, wake up and eat, He want to take away with Him I am ready.

**--- Interviewer- Thank you mama, the medicine we are talking about here is the care we receive from the hospital. My question is that what are the benefits of genetic research to medicine.**

04 – If I would answer your question very well, the time I used my drugs religiously my BP was well controlled. Before I used to wait till I eat before I take my nifedipine but a matron educated us sometimes ago the it is not a must to eat before taking nifedipine it is only vasoprin we can’t take without food, we must be consistent in using our drugs. Before my BP was fluctuating but now it is very okay. I hope I got the question right.

03 – If I heard you right you said benefits of genetic research. There are lots of benefits in genetic research, if someone has a disease and gets to the hospital before they prescribe any drug at all they will do some investigations what is good for you, good for your, what is good for your system because what is good for one person may not be good for the other. For instance when I was still going for physiotherapy, my recovery was very fast, I could not believe it myself. I spent 5 days on admission, by the time I was discharged I could not walk without walking stick, but to the glory of God and to my surprise after a month and few days I resumed back to work. This is where I am going people used to ask me the type of drugs I used, what I used to tell them is that it is not the drugs I used that matter because the drugs I used may not be good for you. Some people are using close to 8 thousand naira drugs a month, mine is not up to 2 thousand naira, that is what is good for my body. That is exactly about genetic research it will to know what is good for you and that is what they will give you.

01 – In short, the benefit of genetic research is that some people prefer herbs to orthodox medicine but orthodox medicine works better and faster than herbs or traditional medicine because herbs does not have measurement, but orthodox medicine has standards, you will see noticeable changes. Like uncle said the other time when I started physiotherapy, there were rapid changes in my body.

07 – I greet all my father here present before me. What I want to say about the research you are talking about is that for me by September 2^nd^ it will be two years that this challenge started. When this challenge started I was totally on supplements even when I was discharged from the hospital because my condition was a bit bad half of my body was paralyzed till I was discharged from Lantoro hospital I was totally helpless they used to carry me but when I got and started with the supplements I started seeing changes I could lift my hand and legs. This makes me to believe so much in this supplement to the extent that I was not taking the drugs I was given in the hospital for more than a year. There was something I added to the supplement then that was exercise, I used to exercise so much that it got to a time I almost run mad, I was later admitted to this hospital because then I was not thinking about anything but exercise. There was a day I went for exercise with my wife, when we were come home I started misbehaving, ran amok. They admitted me to psychiatry ward here in FMC and I spent another 10 days. Where my problem from was my father, he didn’t have stroke but he had high BP and diabetes. It never occurred to me to always check my BP, I believed I was not old to have such conditions, but I was careful of what I drink and eat. My mother too is diabetic and hypertensive, I pray to God all the time so that diabetes will not be part of my challenges. When my sickness started and I was on admission, I said only if I had been more sensitive and careful I would not have had this disease. At a time my healing stopped no more improvement, I am a right handed person and the sickness affected my right hand, I had to train myself to use my left hand and this made me to abandon my right hand. This is a big regret because had it mean I trird to use my right hand then even though I could not use it maybe I would not have abandoned the hand totally to the extent that I now use my left hand instead right, the left hand is now like right hand for me. Even in bank, I was using thumb print initially but now I told them I don’t want to thumb print again I have changed back to signature, I now use my left hand to sign. My left hand is now very strong, all the thing I do before with my right hand I now do with my left hand. So, my experience is a bit different but even at that I believe there is nothing too hard for God to do, He has been in charge from the beginning I believe He will continue to take the glory.

2. **Can you explain what you understand by biobanking**

05 – Try to xplain to us what you me in a way we will understand, we know about blood banking but biobanking that you said try to explain.

**- Interviewer explained further**

05 – I know about blood donation which is very common, everybody knows about it. If you want to donate blood, you will go to blood bank, they will test the blood volume to be sure the person has enough blood. So, talking about biobanking am I in order if I mention blood banking?

**Interviewer- Yes sir, you are in order.**

01 – Talking about biobanking, I have the experience, when my father was sick I donated blood for him. Before I could donate they tested my blood and they confirmed that it is compatible with my father’s blood, they took it and used for him. After two weeks my father needed blood again and I didn’t know that I would have to wait for six month before I would have to donate again and because I am the only person that could donate I have checked on family members no one was compatible. So I said it is okay after two weeks, and the nurse that took my blood the first time was not the one to take it the second time maybe by now I would have died but as God would have it the nurse that first took my blood was on night shift, I thought she forgot something she then came back and she saw me where I laid down ready for the other nurse to take my blood and she said uncle you donated blood two weeks ago you can donate blood now, the other nurse said I should have told her that I donated blood two weeks ago. So, I had to go and buy blood that they used for my father. Blood donation is beneficial to the person that is donating and the person taking it, though it is more beneficial to the one taking it than the one donating but blood is too much in someone’s body it can cause problem to the health of the person.

- **How does biobanking operate?**

01 – I don’t know how biobanking operate, I don’t know what happen to the blood they take except that he give it to people that need it.

07 – I have experience about blood banking if we are talking about blood but I know biobanking is not only about blood.

- **Biobanking is not only about blood, bio does not mean only blood**

07—I believe it is not only about blood, talking about blood when they test the person and the person is fit to donate it is possible for the blood not to be compatible with that of the person that needs it, I have had the experience before, they will still go ahead and take the blood and replace it with another one from the blood bank that is compatible with the blood of the person that needs the blood. I think they refrigerate the blood in the blood bank, I can say precisely how they do it. In case the relate of the person that needs blood cannot donate they may have to pay or if the person needs more than a pint of blood and they can take all from the relative they will pay for the other pints because there is limit to the volume of blood they can take from a person at a time.

**- How important is biobanking to medical breakthrough?**

03 –Biobanking is very important to medical breakthrough, I know that some people voluntarily donate blood which they bank. In case of emergency they can use these blood even though the family of the person may have to pay for it or donate to replace it but as at the time they need the blood it was available and they are able to save a soul. Another one is about kidney, if they have it in bank and there is something that needs a kidney they will use it to save the life of such person.

04 – Just like my people have explained, bio banking is good. If not for people that voluntarily donate blood that they keep in the hospital those patients when bring to the hospital on emergency, there wont be blood to be used for them that is the reason why biobanking is very good. This is the reason we should have it in mind to always donate blood whenever we have the opportunity to do so.

**- Are you aware of any policy or law guiding biobanking?**

06 – I will not call it law, I will rather say culture, there are some religious sects that are against it, they don’t take blood they will rather die than to take blood. They don’t take and they don’t give, I don’t know where they get believe or culure. Just like the others have said it is a way of helping people if we can donate. I got an information maybe I read it or heard about it they said those that have died they still make use of some parts of their body. I can really say much about this, I heard that pregnant women that go through cesarean section to give birth, they don’t use ordinary thread to close them up but veins from the dead are useful for these.

Biobanking is good, our God is not against it, any culture that is against it does not love.

07- What I want to add to it is that, if anyone wants to donation his specimen, they make it confidential. I can say this is a policy of biobanking. Maybe the person want to donate one of his organs that are in twos like the kidney, they make it confidential if that is what the person that donates it want. I heard about the late Michael Jackson that they researched on some parts of his body and this was made know to the public. I believe that biobanking help science to improve and how we people evolved from time to time and period to period.

03-- If I heard you right, you said law

**Interviewer-- Yes**

03 – Anyone that want to donate they will first test the blood to be sure it is not infected, and to be sure the person has enough blood, that is a policy. Then before they transfuse anyone they will make sure the blood they are giving the person is compatible with the person’s blood if it is not compatible with the person’s blood they will not use it for the person. Another thing is that about kidney they don’t just transplant kidney, they don’t just transplant kidney they make sure the kidney they are transplanting matches with the one they are replacing it with. So, they have laws that guide them so that there won’t be more casualties.

1. – **Can you explain what you understand by precision medicine?**

03- What I understand by precision medicine is that if we go the hospital to see a doctor and the doctor is attending to us he will not see two people at a time the doctor will see us one after the other, and the question the doctor will ask us will not be the same even though we have the same condition and the investigations the doctors write for us to do may not be the same. More importantly the drugs the doctor will prescribe for us may not be the same. That is what I understand by precision medicine.

04 – We don’t know more than what he said, let us continue with our discussion

- **Benefits of precision medicine.**

04- What I see to it according the man that spoke just now if they do test, or they ask question from the patients the drugs they will prescribe will not be the same for instance now the nifedipine they prescribed for me they said I should use two in the morning and one at night and there is someone we attend clinic together that uses the same nifedipine they said he should use one in the morning and one at night, we complain of the same thing. That is what we think that we have the same problem but the doctors know better based on the investigations we do.

- **What is now the benefit**
- 04- The benefit is that people should not ask for drugs or used the drugs prescribed for other people, they should go to the doctor. There was a time things were tight for me, my children didn’t have money to care for me. One of my children now gave me one nifedipine that was 20mg whereas I use 30mg, I used my initiative since I use two 30mg in the morning, I then make it three 20mg in the morning and I looked for money to buy little tablets of 30mg for night since I use one 30mg at night. When I got to the doctor and explained what I did he condemned it and said it is not good at all. I also went to a pharmacist he told me if I am not care the act can cause mental illness. So, it is good to always go to the doctor for advice, I will never collect drugs from anyone since then.

05 – I believe in truth it is only God that can cure, the doctors are just trying, any drug that is prescribed for us let us put our trust in God and pray. It is God that cures.

07 – I want to use myself as an example of the benefits of precision medicine, when I was discharged from FMC, my mother discussed with her friend and that friend told my mother that her son had mental illness too that she took him to mental clinic at Oke-Ilewo that was where her son was cured that my mother should take me there. When I got there they took my history and all that and they gave me a drug olanzapine that I should use 10mg daily and come back again in two weeks. The drug helped to sleep because I was unable to sleep before, the sleep was even getting too much. When I went back after two weeks or so I told then the drug helped and that I sleep too much. They said I should cut one into two. From this my experience I can say that precision medicine is good and very important for patient as well as the doctor.

- **Is precision medicine important in Africa?**

07 – It is important in Africa, we too have to improve on our technology like the western countries.

03 – Yes, we need precision medicine there are some mistake our father and mother have made in time past and they didn’t see it as mistake because they didn’t have the knowledge. Precision medicine will help us to correct this mistake and by so our health will improve.

- **Source of information**

07 – We read it from books, we also hear from people that have knowledge more than us. We also heard in our churches and mosques from our pastors and alfas. Those that are aged heard from their children and so on.

04 – In addition to what he said, I as a person since I have this challenge and I have been to the hospital to see the doctor I believe so much in hospital care there is no time they book for clinic that I don’t come for it, nothing anyone tells me outside I must come to see the doctors here, I have a brother, he is a doctor too he said if I don’t visit FMC I will not be at peace. I said this is where I was cured, I can’t go to another place.

1. – I can’t say precisely

02-I don’t come to clinic all the time and the reason is that I don’t have money, yesterday my daughter in law insisted that I must come today she is the one that gave me #1000 so that I would be able to do whatever they ask me to do in the hospital in case it will involve money. If I have money of my own I will always come for clinic and I don’t have anyone that is working for me.

06- According to your question that where did we hear from that our source of information. I said I hardly go to the hospital even if someone is playing with me and I noticed needle in his hand I will run away from him, I don’t play with people that use needle in any way. I know someone that come to FMC from Badagry, I came to see the person here because I have not seen him for a long time, I came into the hospital and went to wait for him at his clinic. While waiting I was listening to health talk that was going on for ulcer patients. I realized that ignorance is our major killer most of the foods we eat are poisons. Since that day any time I am opportune I came to the clinic either I have appointment or not and I will join any clinic where they give health talk, I know that I will go home with one or two things. I used to advice people to do likewise, police will not arrest you for doing that, you don’t have to pay for card or for any test before you can listen to health talk. From there I have gained a lot of things that are useful.

- **Awareness go any policy or law guiding precision medicine.**

05- I didn’t like going to the hospital but when this challenge started, I met myself in this hospital. Like I said earlier that it is God that cures but the doctors are trying. When I got here they said we must listen to health talk, it is from the health talk that I learnt we should not use traditional medicine, no herbs we should always use the drugs the doctors prescribe for us, we should not buy just any drug over the counter because is an anti-hypertensive. I learnt all these from here and I follow it, they also said the human body is full of different things but there is nothing happening to the body that those not have cure, when they tell you to do this and you followed it, it is possible you get cured but if you do follow the doctors’ advice it is the disease that will kill the person.

01- In short what I can say about the policy of precision medicine is that if the doctor prescribe a drug and say you should use one per day though the drug may be small one have to follow instruction. If you now thing because the drug is small you will take two so that it can work fast that is self-medication. A times they may say we should use the drugs for two weeks and come back for observation we have to follow the policy and law the professional medical doctor gives us. May the Lord help us

**4. What do you understand by brain donation for research purpose?**

01- I am not sure of what I am about to say because I just heard from people mouth I don’t know how far it is true. I heard it I didn’t read it anywhere, they said our father late Obafemi Awolowo before he died said after his death they should harvest his brain and keep it. That is all I heard and can say about brain donation.

06- I can say I have never heard about brain donation until this morning but the question you asked earlier have answered this question, all part of our body can be useful after death. That is the work of the medical people, they still find most of these parts useful and it is a good thing.

05- I heard but I have never seen or read it anywhere, it is just a hearsay no record of it, they said that when Dr Tai Solarin was about to die he said his skeleton should be kept in a laboratory to teach medical student.

**- Complexity of the procedure.**

**0**7- The procedure is not easy at all, the reasons it is not easy are up to two or three. The first is that if the person is not someone that is very strong like our two fathers they mentioned earlier, Dr. Tai Solarin and Chief Obafemi Awolowo even though what they said are hearsays we will see that it is not common to see people that can support or believe in things like this among the blacks. Also if at all there is someone that volunteer to be part of such, that willingly said he will donate his brain pressure from parents, our culture and belief will not let it happen. After death the family will not want to release the corpse even though he willingly agreed to it before he died. That is why it is a very hard for black race to fully support it even though we know it is good and we see that it is good.

- **Why did you say it is good?**

07- We have mentioned the benefits earlier, it will make scientific research improve, look at the lines on our palm it is science that make us to know that though the line may look alike but it is different, throughout the world there are no two people that have similar lines on their palms. It is research that made us know, if not that they did research to that level they will not be able to say it. It is good and all of us here know that it is good but we are not there yet in Africa.

-**Cultural, social and religious belief on donating brain for research purpose.**

06- Our culture does not support it, it is not only our culture. My thought is that all these things we are talking about though once we die that is the end but we don’t see it like that ordinary sponge they use to birth the dead body if any takes it they will believe he wants to go and use it they prefer to bury the dead body with the sponge talk less of removing a finger from the dead, but it possible they do that at the mortuary. I know one old woman that cried out before she died that they should not take her corpse to the morgue I wonder what made her to say that before she died. I think she believed that any dead body they take to the morgue would not be complete when they bring it back home. So therefore our culture does not support it, if someone says he is exposed and volunteer to be part of it the family will disagree. That is it, but it is a good thing.

07- Let me say something about religion, I don’t know much Islamic religion but as a Christian, donating body parts is viewed as a sin by some Christian, to them it is like you are challenging God by doing so. So, religion does not fully support it, I don’t know about Islamic religion but in Christianity they believe the dead body should be buried as a whole.

04-Islamic religion too does not support it, you can joking faint in front of a muslim, if you do that by the time you open your eyes you are already in the grave. If someone dies in the morning before 4pm they must bury the corpse. Islamic religion does not support it.

01-Firstly about culture, our culture does not rule it out in any way that we should not donate brain or any part of the body, this has to do with the individual, if anyone likes it he can go for it.

Likewise the issue of keeping the dead in the morgue, there is no law or policy that say dead bodies must be kept in the morgue. It is our ignorance that make our old parents that say they do not want to be kept in the morgue. About our religion, Christianity has different denominations, I am an Anglican and we are not against blood or organ donation or keeping corpse in the morgue. Also in Anglican it is not a most to bury the dead the same day or in a hurry, it is when the family are ready, when it is convenient for them to bury their dead, but the one thing is that we don’t do burial on Saturdays so that it will not disturb another service. Some religion don’t support.

03-Talking about religion, there is no religion that supports organ donation and use, what I can say about culture is that we use to hear that when a traditionalist dies they use to remove something from the body. I have never witness it but we use to hear that when kings die they use to remove something from their body. Even though people do not want it but they still do it.

- **What factors promote brain donation?**

03-Orientation is very important, may the Lord help our government, if they do not orientate the people so that they understand what we are saying, it is not going to be easy. For instance, in my community, they organized a program and ask people to contribute money towards it. The program was a very good program but people refuse to contribute with the excuse that they didn’t hear about it. We then said the executive should go out with gong and megaphone to inform the people also from house to house to collect the money. So, our government through the media should get our people informed.

- **Personal willingness to donate brain. You as a person can you willingly donate your brain.**

03-I can’t donate my brain

07- It is going to be hard

06- If I heard you right you said it is after death that they will use the brain, it is not something that everyone will agree to but some enlightened people in the family may support you.

- **Either your family will agree to it or not, you as a person can you willingly donate your brain.**

06- Okay, if we understand our religion very well we will know that it is our spirit that God needs. I have never travelled out of Abeokuta but I heard that in some countries they use to burn their dead and keep the ashes inside bottle or envelop for remembrance. They have the knowledge that we are sand and we will go back to sand either you use it or not within a week if not for the injection they give these days to preserve the dead after a month go and check maggots would have eaten it. It is easy to willingly donate by people that already have the knowledge, and it will be in secret not that it will be open to every. So far the spirit is gone you can do whatever you want to do with the remains if it will be useful but make the body neat when you harvest what you want from the body not that when the body is brought out people will know that it has been tampered with and use it to abuse the children that they buried half of their father’s corpse thy have sold the remaining have. If it going to be in secret it is good.

07- I said earlier that confidentiality is very important. In Africa here they call something stigma, this will not let people that wish, people that have the knowledge to come out to say he want to donate for research. If anyone hears about it they will say it out even though the person is gone his family will be the one to feel the shame.

- **In this gathering I want to do head count how many of us can willingly donate brain for research**

1. Four of us.

**5. Share with us your opinion and thought about blood sample donation for stroke genetic research.**

05- I am aware of blood donation, I have the knowledge I can willingly donate blood for researchbecause I know the blood will go a long way to save a life.

03- I will be alive when they will take my blood I can donate blood, I shared my experience the other time I have donated blood before.

**- What do you see as barriers that could hinder your donation of blood sample for stroke genetic research?**

03- What can be barrier for me is if I don’t have enough blood because if they take from the blood that is not enough for me it will affect me and I am alive I am not dead.

- **Parental influence**

05-Family can only have control on a dead body because the dead is gone he does not know anything again but blood blood donation is done when we are alive the person that is donating owns he or her body the family cannot have any control over him.

04-Family cannot stop the person, he owns himself

03- Noone will go and announce to the family that he is going to donate blood.

07-We have the right to know the outcome of the research they use our blood for if we are still alive, am I correct? I want you to answer.

**- What do you perceive as benefits of giving blood sample for stroke genetic research that could promote your willingnss to donate?**

07 – Atimes one can ask for monetary benefit in returns for the blood he donate, that is another benefit.

**- What can you say about your family member or other member of the community illingness to give blood sample for stroke genetic research?**

03 – When people have orientation as we too got orientation today they would surrender. Now that we know about we understand many things. Many people still believe that if they donate blood they may die but by the time they have the knowledge they will know that blood donation does not cause any harm.

- **What could be done to make you and more people give blood sample for research: mass media, husband consent etc.**

07- For a man or woman that has a spouse, the consent of the spouse is important before he or she will donate blood. The man or woman have to discuss with their partner his or her willinglyness to donate blood for research. The extent in which science is going now you can know the history of you family through blood donation.

1. If my wife wants to donate blood voluntarily, maybe she hears about it and she wishes to partake, she must seek my consent first, but if she wants to donate for the family either mine or yours she does not need to seek my consent because I must have known about it. If is for people outside or she gos to the hospital to donate voluntarily without seeking for my consent first it is possible she will not breturn back to my house.
2. What I see to this is that we are doing this to help someone, to save a soul, I don’t see anything wrong in it if we have the grace to donate blood we should do it. There is a verse in the Bible that say anyone you have the means to help is your partner take care of him. I don’t think it is necessary to go to the spouse to seek consent before blood donation. Anywhere I see that I can’t be of help by donating blood, I will go there to donate.
3. It is good to seek the spouse consent before donation in case another happen it is the spouse that will be called, so in the onset the spouse should be involved.

- **Having a donors group, will it make you and more people donate.**

05- Yes having a donor group will be useful if our people have the knowledge, so they have to make people aware first but public announcement.

**6. Explain current practice about blood sample donation for research.**

03 – To my understanding when they take blood from someone they don’t just use it like that, the blood go through some processing bfore it will be used. For instance if we want to eat rice we don’t just take the raw rice and begin to eat. The rice will have to go through some processing like boiling of water, washing of rice into the boiling water, adding of salt and onions and so on. So also blood goes through processing before they use it.

**8. Tell us what you know about informed consent.**

03 – Informed consent has to do with having understanding of what you want to do. When you have the knowledge of what you are about to get involved in, it will be easy to give consent.

01 – Informed consent will help the researchers to improve on the research work. Informed consent is useful in a research for the researcher as well as the participants.

**- What do you know about the consent process for genetic research?**

06 – If you give consent to what you do not understand when you get home you will blame yourself whatever decision you have taken you will change it. Understanding is very important before you give consent, if you understand you will be able to explain to other people that do not understand so that they will cooperate. You just gave us paper to sign we didn’t understand why. So enlightenment is very important.

03 – Also the language you use to explain must be the language the majority understand, the consent should not be in English it is not everyone that understand English even though we go to school English is not easy to digest immediately. The people at the rural are do not understand English language. Any information the government want to pass to the public must be in Yoruba otherwise majority of the people will not be informed. The people in the rural area listen to radio most of the time, any time English program is on they switch off their radio.

01- My father listens to 9am morning news in Yoruba, 3.30pm, 5pm and 9pm after that there is no program he listens to again.

**- Type of informed consent preferred**

05 – All is good. I prefer the broad

1. – The question you asked now made me to remember when I was on admission in the hospital, some people came to me that hey would like to take my sample for a research, I gave them the go ahead since I know it is all for good. Like you explained I prefer the first one, the broad type. To me I don’t see any thing wrong in using my sample for two or more researches if it is possible since it is for our own good. I prefer the broad if you want me to sign for it now I will do so.

03 –I prefer the broad type the reason is that if I go for restricted or tiered whenever they want to use my sample again I may not be around to give another consent, and since the broad is a general thing like the general hospital whatever they want to use it for they should go ahead and use it for it. As we are here today, I am suppose to be somewhere very important, the person has been calling me, I have to send a message to him that I am in the hospital when I finish from here I will go and see him.

07 – I prefer the restricted or dynamic the reason for my choice is that at this junction I realsed that we need more orientation. If I want to donate blood for a course I must discuss with my wife, if she does not accept I will not go ahead with it. I know that they can know a family history, family trend through the blood science has gotten to that now. It is because we don’t have the knowledge that is why we are saying the broad, I don’t blame those that say they prefer the broad but for me idont want the broad consent. I prefer the restricted or the tiered.

02 –I don’t understand anyone of it.

01 – I raised my hand the other time I wanted to say something but now our time is far spent but I will still say what I wanted to say to be able to answer this question. The bible says Jesus is the head of the church just like the husband is the head of the wife. So anything the right hand is doing the left hand must know about it because we are the head, the lesson we learnt made us to understand that our wife is the body, so anywhere you are going and you do not involve your wife it means you have left your body at home, head and body must work together. That is why I seek for the advice of my wife before I do anything because we are one, before we do anything we talk about it and we involve God, we talk to God about it.

I prefer the restricted but that is not my final choice because I still have to discuss it with my wife and if she says know, it means no.

06 – If there is no going to be any implication I pefer the broad since you already have the blood with you it is not that you are going to take another one from me you can use it for anything you want to use it for.

**- Person to be involve before participation.**

01 – My wife.

07 – My wife and parent

03 – If it is necessary my wife and my children.

**- What do you think about generic consent for the community.**

01 – For me to donate my sample for research does not concern my community leader, even my family head it does not concern him. The only person that it concerns is my wife.

02 – Only my children, it does not concern the community leader.

**9. What is your opinion on storage of blood sample and blood fraction for future use?**

03- We have talked about it

1. – We have talked about it, it is to save life. Let us move on.

06- Whatever you like you do with it

**10. Tell us your opinion about sharing of data, blood samples, and brain images or brain tissue samples.**

06 – There is nothing wrong in that because it is the research some people do that gives us the knowledge of how to treat such diseases. So if they do our own too and find out the causes of our disease and how the disease can be treated and send it to another country I don’t see anything wrong with that if it will not involve mentioning our names.

07-The issue of confidentiality is very important because if data is not properly managed there cold be leakage which have legal implication that may result into suing ourselves. Like my daddies here talked about Dr Tai Solarin and Chief Obafemi Awolowo if not that the information leaked out people were not supposed to hear about it, it is suppose to be strictly between the family and the people involved in the agreement. So, if they would be able to manage the data properly why not cross examination is good for further studies.

1. Last year during the world stroke we were invited and they gave lecture on the prevalence of stroke. If not that they share data they would not be able to make that comparison and we would not know able to know about it, they did not mention any name they only told us the rate from country to country. It is good to share data.
2. I wanted to use something someone sent to me on whatzapp to answer you question but I don’t know where I save it on my phone. The human system to explain the nutitional value of what we eat and how it goes through our system. It is a picture of someone that he used I do not know the person it has nothing to do with anyone since there is no name attached to the picture. All these research will be useful, I don’t see anything bad in sharing

**- What do you think about commercial and non-commercial use of stored data, blood/blood fractions, brain images and brain tissue?**

01 – I don’t support commercial use of data, they will now turn it into commercial ventrure for some people and it will not be available to people that genuinely want to use it.

06 – If iunderstand you well, this broadcast on whatzapp is part of commercial, they used it to advertise some supplements that the foods we don’t see to eat especially fruit are combined to produce the supplement. If we take a tablet in a day all the nutrient we are to get from fruits and vegetables are there, I believe this is part of commercial, I don’t think anything is wrong in that.

**11. Share with us your thoughts about return of individual research results and incidental findings**

06 –I mentioned ‘’family and friends’’ when I was talking, it is a program I use to attend every July, they will take samples from us and promised to send the resukt of the findings to us through our phone numbers. I went the first and second year and I didn’t hear anything from them, I decided not to go again. The one they did this year I didn’t go there. It is good to return the result of findings to people that donate so that they will know if there is anything wrong with them and they will be able to seek medical care.

**- Opinion on desire for feedback of research result.**

06 – We are in the era of internet, they should use any means that is convenient for everyone, it can be whatzapp, SMS on our phones and it can be email. When you are taking the sample you will ask individual what they have, the most important thing is getting it across to us.

03 – It is compulsory that you return the result of findigs through the internet according to daddy.

04 – I support what others said, for those that do not have whatzapp or email they should send it as SMS to their phone.

05- The healthcare workers should give feedback

04- The healthcare workers are the ones that did the research and they are the ones that know what they see there that they want us to know about.

**- What are the ethical, legal and social issues relating to returning individual research results and incidental findings generated by genetic research?**

06 – I don’t think there is any law that is against you returning the result of your findings that is how I understand your question. If your findings is something that is positive that is not bad you can send it through email or message but if your findings is not good it is better you invite the person and explain your findings to him in person and counsel him on how to deal with it.

01 – With the explanation of daddy the waywe present issues is very important, for instance they say the finding is tuberculosis or HIV, they should use a word that will not make the person feel bad. Everyone knows HIV to be a sexual transmitted disease they can use another word for it that will not be too hard. There was a time I was diagnosed for tuberculosis, the doctor wrote cough, I never knew it was TB until later.

**12. Explain what you understand by Biorights**

03 – Biorights has to do with obedient, if doctor says don’t eat too much sugar it is for your own good, if they say don’t drink this for our good as well.

06 – We have been donating blood for a while if there is anything new in donating blood let us know.

1. – Human right does not have limit, Yoruba says if someone says his father’s masquerade would come out and dance and the masquerade does not dance no police will arrest you for it. This should also go for the specimen one donates, if someone donate a specimen for research and the outcome helps the whole country. The person that donate the specimen may not be alive again but his children should benefit from it. The people that are doing the research do not know to what extent the discoveries of the specimen will get to if they had known it there is no reason for them to do the research. There are some people that their religion restricted them and they see these things we are talking about as sin and can hinder them from entering heaven. I believe that when Adam sinned and God sent him out of the garden of Eden, all is plan thereafter was evil, it may be difficult for me to convince about that, if I try to elaborate on my reason we will not leave here because I did not just come up with that. So, in Africa there are some things our black face ie hindering us from but if we open up to research we will discover things that will be beneficial to the whole world.

The more we know about this the better for us and the whole world

04 –Me as a person once I have willingly give my sample, I don’t think I will look back.

- **How should autonomy right be balanced with societal benefits**

04- It is the person that donate his specimen that suppose to get more benefits.

**13. What is your opinion about governance and regulation of biobanking?**

07 – There should be human right law, whoever donates his specimen should be entitled to the discoveries because he is the owner of the specimen that brought about the discoveries.

01 – There should be law that will make it confidential.

05 – I want to call your attention to something, you are the one that know the reason you are doing this, we are only here to contribute to it, I now want to beg you to do this is a way that we that participated would be proud. In the olden days when a child is born they used to check the child’s fortune how is future will be like, it is true it really works but later people started using it to do evil that is why it is not common again. This is what I am calling your attention to, as good as what you are doing is, if you use it in a way that is not legal, it will hinder the success of another program you have.

**14. Explain possible intervention for implementation of biobanking.**

07 – We have to do a lot of awareness on this disease because it increases by the day. In the olden days they said stroke is a disease of old people from 70 years and above, it is not like that again, I am not up to 50 years and I have stroke. If not for my religious believe that is against somethings, I wish to know the root cause of this disease in fact I can donate my whole body for research to get to the root of the cause of the disease, I know that by so doing I am helping the generation of my children because since the disease is hereditaryand I have it at this young age what is the probability God forbide that they will not have it at a younger age than mine. This is one of the reason we must give you support so that those in the position to do further research will be encouraged. Whatever you think we can do too as our responsilities you should tell so that we can both be at the win-win side of the agreement.

04 – I think that through churches and mosques those of us that have had the challenges should try to enlighten our members on what we know.

05 – My suggestion is not different from what baba they always say experience is the best teacher, if you advertise medicine for a Hausa man he will ask you if you have used the medicine for the same illness before. The best way is to call people into order about what they eat and drink that is why I don’t like missing the health talk anytime I come to clinic, I have learnt a lot of things from there. Those of us that have had stroke should try to always call our people to do, this will help to prvent the disease. Before I had stroke I used to believe it is caused be lack of sleep, excessive thinking, I am a pastor I don’t do all that and I still have stroke I usd to ask myself where do I get it from.

1. – What someone do not know about he cannot do it well, if people understand these things they will willingly surrender. I said before I don’t use to take injection but now I know that the benefit is more than the pain, I even ask for it now.

03 –What I can say to that is that the Bible says those that are saved should look for the salvation of others, it also says we should seek for the peace of Jerusalem, those that want it shall be prosper. What I see to it is in two parts, the first part is that when I have this challenge some people came to meet me that which drug am I using I told them to go the hospital, those that have been discharged from the hospital I encourage them to go for physiotherapy what I am trying to say is that we should pass information across to people, we should tell people about our experience and encourage them to seek medical care. The second is that you should go to media house and make public announcement, enlightenment program and explain to the people by this they will have the knowledge, this is what will help this program.

01 – We should create awareness for ourselves. Education, just like uncle said, now that we know anyone that come to us we will enlighten them also.
