# Supplementary material for: Research participants’ perception of ethical issues in stroke genomics and neurobiobanking research in Africa
Source: PLoS One. 2025 May 6;20(5):e0292906. doi: 10.1371/journal.pone.0292906 (PMC12054916; doi:10.1371/journal.pone.0292906)
Supplement: S3 File — (ZIP) [file pone.0292906.s003.zip › Files for PLOS ONE - updated March 2025/FGD_Community Advisory Board_Abeokuta.docx]

**Interviewer- what do understand by genetic research?**

R3- my understanding of genetic research is we are going down to the cellular level of human being, trying to find out the core of some of the issues that are affecting human being… diseases and all that sort, so we are going down …and the genetics mean at the cellular level, the basic unit of life, how does it contribute to whatever its happening to human being

R1- we have a lot of problem associated with this…., it is a pity, you said there is something private that should not be heard or linked to anybody. To me I am nothing but God knows me, truth is bitter but say it and you will find out that at the end lie can go on for 20 years but one day truth will come out, so this genetics hmm is a big problem in Africa, there in Europe, I will say their problem is……, if our own is 100 percent, their own will be 2 percent, that place, they have no problem.

**Interviewer- so you agreed that Genetic issue is a big problem in Africa**

R1- it is a big problem

**Interviewer- so, what do you know about genetic research**

R1- it is associated with so many diseases but now you only bring out stroke

**Interviewer- have you heard about genetics research before**

R6- yes, I have seen it on papers……laugh……. But I don’t have knowledge about it

All respondent- ………. laugh………..

**Interviewer- you have heard about genetic research, where did you hear about that**

R3- thank you very much, I am in the health sector I have ben involved with………………., for two decades and we have had issues in which particularly people with permanent disability like cerebral palsy in which some of their parent are looking at finding a lasting solution, so some of them read articles on stem cell therapy, so we have some of this patient that decided to take their wards to Europe, is it India or whatever, that should be about 2 years ago, trying to try out this stem cell therapy, that affords me the opportunity to read about it, it’s like trying to go down the tissue and maybe they can do a transplant and do something about it

R4- the issue of genetic research, it has started quite a long time but unfortunately, Africa is way way back , most especially in our settings, resource limited country like Nigeria, when we talk about genetic research, let us look at it from the biblical points of view, it says that “**search for knowledge, it will set you free”**, a lot of diseases that we have in Nigeria are what those in the western world have done away with them or they have been able to manage them effectively and efficiently but unfortunately in Africa because of our settings and because of resource limitations, we have not been able to make head way and the issue of genetic research came into being about 25 years ago in the western world but we are just keying into that technology and that the technological drive resources and structure is not there on ground and It’s like we are just trying to build it up now. Now, genetic research is like a house that has been built and there is a small crack somewhere in which you don’t know where it is precisely, not until you break down the whole house to the unit level, that is when you will be able to identify where the problem is and if you are able to do it in time, that cracks will not become bigger, so that it doesn’t break the whole house, so the earlier the better you identify, the better it’s for the whole structure, now it’s about going down to the unit level of it and seeing where the problem lies and what causes the problem, why does the problem arise, when does it arise, there is something in epidemiology we call, the questions: where; how; when; what; and probably who, so now, answering those 5 basics questions, we can use genetics to answer those questions. Taking for instance, diabetes, we can use genetics to answer those five questions; precisely; who get affected the most in the population for diabetes, why is this particular person predispose to this disease, why am I not having it, then when, diabetes in a particular population does not start at the same time, in those days we use to know that whoever has diabetes is ah.. is an elderly person, whoever is having diabetes is Elderly, we considered the elderly as 65 and above but now because of genetics we have been able to know that things are coming down, now 35, 25, 20 and all this thing are having diabetes, why is that happening? because they use to eat fruit those days, they do a lot of exercises, they don’t have the junk food, so these are epidemiological questions that genetics, going down to the basis will help to answer those questions.

**Interviewer- what do you know about genetics research in stroke**

**R4-** genetics research in stroke is…….to be economical with it…. is non-existent in the African settings. Why am I say so? Let us look at the basis, an average man on the street, if you ask them what do you know about stroke, they will be giving some varied information, varied answers about stroke, but precisely they might not be able to say, not in medical term but the real answer about stroke is non-existent in an average individual, there was this study that was conducted even amongst high calibre individual, civil servant, we are talking about level 10, 11, 12 and above, it was so amazing that even as high as those calibre of people were, they don’t the basic information about stroke, diabetes, hypertension

**R6-** I want to pick something from what he said, the stroke project we have, some of the information that I pick from there, it has…….., within the medical cycle, it is known that some people are more disposed to having stroke than others, for instance, blacks are more disposed to having stroke than the white and maybe female more than males, so if that be the case, there must have been some research done to determine that

**R7-** I also think that to some extent, it has to do with what I can refer to maybe hereditary, something that you can find in a particular family, maybe your parent or grandparent has something like that and then it is expected that you would have it because when it comes to probing, then we want to know what really cause it, what is really happening and then sometimes it is traceable to our family lines.

**What do you think are the roles/benefits of genetic research in medicine?**

**R2-** just like he said, that genetic research is about maybe you have built a house and there is a crack and you really want to know what cause that crack, off course if you do know what cause it, you should be able to prevent it from happening to another house, so that when you are building that house, the problem in the first one is not repeated, so I want to believe that the advantage of this is more of prevention than curing, so if you are able to identify that this and this are what happens that made this to happen because generally I have discover that in most of our hospital, when you go and complain of certain things, the first question they want to ask you is whether it has happened before in your family and I think science has gone beyond that, what happen to my father may not happen to me or what may happen to me may not have happen to my father, so I think generally when you carry out research, it goes beyond hereditary or what have you, once you know the reason for a particular problem, then preventing it becomes easier. I believe this kind of research will give an idea

**R3-** it is a very strong benefit because it shows that man can interfere with the way human body is structured at the cellular level, …….not clear….., a nobel laureate, an American Egyptian, so his work was studying molecular reaction at a speed of 10-^15^, that speed shows that they can now study the level at which molecules are broken down into atoms and the bindings, you know, the initial knowledge we have before is that matter can never be created or that….., so the study have given them the opportunity to see the cleaving of atoms into electrons and ions and the binding, so the benefit of that knowledge which is the genetics which means they can alter the binding, which is a very strong contributions to medicine, probably we can cure a lot of hereditary diseases

R1-uhnnn, somebody mentioned that, somebody on level, I will say level EPA

**EVERYBODY- laugh**

**R2- baba**, you will have to interpret what that one means because I wanted to react to what my brothers just said because he is taking us beyond the level some of us understand. I want to beg the moderator to ask my brother to break this things down as much as possible, so that some of us can understand what he is talking about because this is a community based research, so if they don’t break it down as much as possible, even me as educated as I think I am, I will be lost, not to talk of other people

**EVERYBODY- laugh**

**R1-** as a community leader, we have a lot of problem, sometimes when you have some sort of sickness, they go the clinics, they said you have to pay so so, she hasn’t got the money, they will come to me, treat, I will pay, so with that, if he is quickly attended to, that reaction will be minimal but if not attended to in time, you know it will prolong, it can even lead to death

R4- Apart from the preventive aspect and the curative aspect that has been mentioned, we have the promotive aspect of it, genetics banking can also help, genetics can also help in promoting health, now, in the food that we eat, the population is ever increasing, the farmlands, the rate at which the population is increasing is not commensurate with the farmland and a lot of people are not going to the farms and what have you, genetics, we can use it to increase the yield, that is in Agric, in medicine, we can also use genetics to promote people’s health, a lot of people are having diabetes, sorry, that is what comes to my head, a lot of people are having diabetes, now you can use the genetics findings in one person to solve the problems of another person that are alike, that are on the same platform, then apart from that, you can also use it to protect people, like all this long lasting insecticides bed net that we are using, a lot of those things are impregnated with some chemicals that repels the mosquitoes and all these things, even research has gone into making mosquitoes non-transmittable of the malaria parasites, which means when mosquitoes bite MR A who is having the malaria parasites and comes to bite me, because of those things genetically imbibed in the mosquitoes, that parasites will not be able to survive on his/her own blood, so that by the it bites me, it will not be able to infect me, that is protecting me against. Then, we also have rehabilitation, now somebody has suffered from stroke or somebody is having this diabetes or somebody is having whatever diseases and all these things. The process of bringing the person into a person to the which he or she was like the physiotherapist now, you can use genetics to help that person overcome he or her challenges, that is rehabilitation and bringing them back to their position ab initio. So, genetics has a lot of benefits

**R6-** it will also help in bringing precision to medical approach to diseases.

**Interviewer- what do you mean by precision**

**R6- yes,** I was trying to run around what his been saying, like we know now that….., Like what I have heard, coming around here that diseases like diabetes, stroke, they are not condition that you can say take this drugs and then you are okay, you manage them, if anybody has that, you manage them, maybe an advancement in genetics research in Nigeria or in Africa would one day or can one day address that problem and somebody who has diabetes can say okay, this what they have found out and then you can take this drugs and its heal completely, you know, such precision can happen through this. Then he mentioned something about food and that got me worried because I remembered I read something about genetically modified produced food or something and I was told they are not healthy that they cause a lot of problems to the human system, you know, maybe we would get to that later sha

**R1-** I am happy you talked about food, this additional diseases, we are having in Africa is imported from Europe, you know I have 3 children oversea, my second born will say daddy, he has been there for about 28 years now, when he came, he said daddy, you are very happy here, you are very lucky here, you are eating something better. Now you born a cow like a child today, it will grow to become eatable in 3 months times and somebody will buy that one and import it to this country. Something which should last about 2 or 3 years before you can eat, within 3 months, I work in a place, when I was there, that was 1965, it is the end of a train, it is a train route, one day, I don’t know all the cheat, excreta, the whole excreta in London, banking, you understand me now

**Respondent-** everyone laughed

**R1-** I never knew, so on Sunday, you take tea every hour or whatever it is, I just look far, I saw some, just like demarcations, you understand me now, and then I was asking, what is happening there, it’s excreta, my own excreta, everyone one of us is there, what is the use of that excreta? What do we use for planting here today? That will help your plant to grow well: fertilizers. They said it is use for fertilizer. ……Laugh…, we have different kind of disease and it is a bank somewhere else**.**

**Interviewer- can you explain what you understand by biobanking**

**R7-** the word has already explained itself, off course BIO is life and banking is storage, so biobanking is the study of storing life.

**Interviewer- so is it a study? Biobanking, because the question is what do you understand by biobanking**

**R7-** that is why I said life storage, storage of human parts, like for example, at least for a long time in Nigeria when you get to hospital, you hear blood bank, blood bank and I know today we have blood bank, so that is a place where blood is being kept**.**

**Interviewer- how much are you aware of bio banking, you have mentioned blood banking, any other**

**R3-** within this area, I have not seen but I have been hearing about also keeping the heart, the heart can be harvested for those are about to die or something like that, they harvest kidney too, I have just been hearing about it but Nigeria or African I don’t know to what extent after blood banking are we into other types of tissue banking, I am not aware but we know in other places, they can harvest somebody that is dying and the heart is still functioning. They can harvest it and keep it for somebody that will need it in the nearest future**.**

**Interviewer- what do you think about that concept, what is your perception**

**R6-** there is a myth that the late Tai Solarin was too brilliant and when he passed away, maybe he was the one who said it or somebody who said that when he had died that his brain was not supposed to be left to decay, that they find a way to preserve it or maybe give it to somebody else who might needs it, that is just by the way but like my big brother said, uhm, I have also heard it that human parts can be harvested, preserved to be used for some other people especially people who died at young ages, so their parts, their eye, their heart and all that can be preserved for onward use but I am not aware of its practicality in Nigeria, though I know of blood banking. I have even donated blood in the past but that anybody has shown me or have told me that in this hospital or in this facility ooh, there are human eyes, if anybody need eyes, they can go there and pick one, I have never heard about that

Respondent- laughter from some respondent

R2- let me just add something very funny to all this, because sometimes, we use our knowledge wrongly, those in the occultic world as we have been hearing, just like maybe when an oba dies, you know, is not just buried, his body part are done away it…….not very clear…….,like I heard as in manners….., we would have done a research as to why was that but rather that doing that, we allow our new faith to make us condemned that practice of our fore fathers. I am not justifying it. I don’t belong to any secret cults or whatever.

**Interruption R1- I am one of the condemners**

R2 continues - Now if they were doing it, for what purpose, maybe that would have been the question, look, now we are referring to the developed world as to keeping all these body parts. What are they keeping it for? So that it may be use by somebody who may need it later on and I know they didn’t start from that, there would have been some practices in times past that were not good enough but they modernise it.

I want to believe that there is something with our part that we need to do away with some cultural cleavages before we can moves on because if you tell my mum, my mother is late any way, if you tell those in the villages that they are going to do something with their body, they will rather their children, make sure if I die today, keep me very well, don’t let anybody remove…, what is he going to need the kidney for after he has stopped breathing but because of some cultural believes and what have you. Then I want to believe that our fore fathers were aware of the importance of this but maybe in a way that we could not really understand it because they will say that if I am an oba dies before the next oba can come the throne, he will have to eat the kidney of the late Oba or the heart. So, why was that, there must be something behind it but those things behind it is what we do not put our mind to. We just condemn in totality this practice, we say it’s ennnn, I don’t want to call it any whatever, even daddy said he is one the condemner. Maybe he may explain to us why he condemns it

**Interviewer- looking at this modern concept, what do you think about that concept, what is your perception on keeping organ or body parts in a bank. Is it something you are okay with, are you at home with it?**

R2- personally before the people in the medical lines comes in, I have no problem with it but I see a lot of obstacles base on culture, perception in this part of the world. See, like I was saying, I just came…, thank God daddy live abroad for a long time, I just came back from the US, last months, I think we are far behind the world in terms of modern science, we are still in the country where you can go to the pharmacy and tell the person on the counter, I have malaria and they begin to give you drugs, nobody does that in America and I know that not many doctors will do that here, if you go the doctor and tell him you have malaria, he will ask you what is wrong with you, tell me how you are feeling, don’t tell me you have malaria because it is until you tell the person the way you are feeling, maybe some test are even carried out before they actually determine, so I believe that this research will go along a way, but we have a lot of things to do, thank God we have a community leaders here, to educate the people as to understanding, maybe because of our level of the level of our education too, we need a lot of enlightenment, I don’t have problem with it, honestly because I know that it is one of the thing that is keeping us back from moving to where we supposed to be and because we don’t have this kinds of research, we are suffering when we don’t need to suffer, personally, let me just say this, I was with a doctor last week, who said how I wish you were my patient 20 years ago, I felt, but because of the way he said it, somehow, is a kind of doctor that you may get irritated but I just love him. I felt within my spirit, he was saying the truth that he wanted to help me but he said don’t worry, we can still manage it, this is something personal now, he said I wish you were my patient 20 years ago. Why did he say that? And why have not complained? Because I didn’t have the opportunity of the modern science to tell me this thing is a problem. I just thought is normal because of the environment and cultural believe or maybe faith because I mentioned faith to the gentle man, you understand, I am a pastor, a lot of times, we just believe prayer can solve it, miracle can happen, there are somethings that ….., even the science itself is miracles, if we make good use of it because it is there in the bible. The herbs and everything that God created they are meant for our good, using them and what they brings about is science itself , so why do we now condemn what God has done, even holding God hand from performing the miracle

R7- my own….. personally now, sincerely speaking as a great contribution to helping others to have more quality of life. We are talking about bio banking, for instance I want to die and I have been told…., there was a time I had a patient here, who happens to be a church member in this hospital, when we were doing all these praying, I just walked up to the doctor one day, I said doctor, toor, please, what, what are expecting, he said which patient and when I mentioned the name, he said oooh!!!, no!no!no!, we are not treating her to get her well, we are not treating him to get her well, in fact we are not applying anything to heal or to cure anything, we are only doing what we call palliative, just to make death less painful for her, he said that is what we are doing, he now used a language, he said on the arrival, we told you that she is long gone, you don’t know the meaning o she is long gone, so he said that I said wow! I am hearing this for the first time, he said no no, the husband knew, go and call the husband and I called the husband to his office and I said ok, my brother, N gbo, are you thinking that your wife will get well, we are not treating her to get well, bla bla bla, all that, this is what I am trying to say, if I knew and I find myself in such situation, sincerely speaking and I know, instead of going with these body, to go and just rot away because eventually base on my understanding, my believe and what I do, what I stand for, this, all this body is useless, whether you die handicapped, whether you die in accident, why you die wholly or fractionally, anyway……laugh….everything is still useless,

so for somebody like me, it will not be a problem, sincerely I see it as still contributing to life after I am supposedly death but you know the community where I come from, it is a community of “**bo dele, ko ki ile”(when you get home, greet them at home) bo dona, ko bere ona(when you get to the road, ask of the road) ate te dele, ise eni lo ko je( getting home quickly, )**

R2- and to do that, you need all the part

R7- off course, you wouldn’t believe even to a very large extent, cutlass is placed in the hand of the corpse in grave that he will use it to fight its killer, so if you now tell such people who have such believe that… as a way of contributing to human life after you might have passed on, we want to do this, it’s like you are calling for the wrath……laugh…in our society.

**Interviewer- how does biobanking operates?**

R3- I just want to establishes that you know cultural believes can be strong barrier to biobanking, knowing fully well now that even the yahoo, yahoo are using human parts for so many rituals, so how are we sure that if we start keeping something in the bank, they will not come and break the bank and steal all the parts for rituals

R6- actually the area of concern I have is two. One, is that Nigeria today, medicine in Nigeria, has it advanced to the level that they can remove some eye and put it in another eye successfully, ok, the eye in the bank, another person needs it, can they do it successfully, without taking him abroad I mean, even other human parts, they can do it successfully. Number two is if that would happen at all, I think the consent of the persons part that is to be harvested must be taken before he or she dies, that is the area I have issue with

Interviewer- you have mentioned a lot about biobanking, what are your source of information

R4- let me take some steps backward, one of us mentioned about the king, what they do with their body part and…, I live in a town nearby who lost the paramount ruler, a first class Oba and another one get enthroned, the second person that got enthroned, we were discussing one day and a big friend of mine just ask. Did you eat the heart, he said yes off course, it has always been like that and the questions that follows, he said how do you feel like, he said, I felt like a man, which means that for those heritage, for the different kings that got enthroned, fpr them to have eaten the heart of the previous one, it gives them much more confident, two, you are manly. That leads to Fulani men, while young, what the Fulani boys do, they lick and drink the blood of the cow, that give the audacity to do, to perpetrate a lot of things. So, that is about that,

Then about the biobanking, we have two operational biobanking, the crude which is our tradition and what happens to King is a crude form of biobanking. Culturally, what they do is that they hang the body upside down, so that it drains all the waters, so it mortifies the cadaver, it thus become dries like dried fish, it can be kept like that for years, what they do……., I am not supposed to say all this things. Then we have the ritualistic part of it, some people takes body parts and all these things but for the refined part is where you have the liquefied Nitrogen Oxide, where you keep the whole body and all these things. Now, somebody raise the issue of ethical issues, that would the people allowed all these. It all boys down to our orientation. A lot of people, probably 2 out of 10, 10 out of 100 knows about biobanking in African settings or in our local settings here. Now, we have been privileged enough to have lot of information but unfortunately this information we are sitting on we have not tapped into it. Sir, a lot of these information about stroke, diabetes, most of these diseases were carried out in the western’s world, using the western man and limited information is on the African and the African settings. Now, they use their own drugs to treat us, now for us to really get information about ourselves, we need this biobanking that retrospectively going back 10, 20, 30 years back, if we have those tissues, if we have those body parts, we could go into them and start seeing progressively, how have this things develop, so that we know how to key in and how to block it, so it’s like a circle, you know how the circle goes and you know where you can go, block and stop the process of disease progression

R1- this man, Dr Christopher Bernard, he was the first doctor to transplant heart in South Africa, so now what happens to that particularly man, he died, what types of diseases, it was not written but we know that when they have a motor accident, people who are just enn, you understand me now, they just remove their heart and keep you know where they kept them. Then for transplant, this is the…., I don’t know if they do that in this hospital, if I don’t know if it has happened. You see when somebody dies with different diseases, you are in position, now, my brother was talking about mosquitoes but I don’t want to say anything but I used mosquitoes to know the …….loud laugh…..the situation of my blood, so when I am praying, you know I use to sit down……not clear….., I was telling a reverend some days ago, I said this Buhari, the man would have been punished if he died in LONDON but if he can displayed himself as true leader, a muslim

**Interviewer- how is biobanking important to medical breakthrough**

R4- we have a knowledge base, and 2 is about research, and 3 is about education. By the time, a group of doctors come in, you use this biobanking to teach them what has happened several years back, they get to know what is in and probably postulate what can happens in the future. Then, also it is money

Interviewer- money in what sense

R4- ma, for you to have access to information, you can sell information that you have, if I have a private biobanking facility and SWISS pharm, they want to come into African to develop drugs for one of these diseases, diabetes or all these things, if I have that facility, they can just come freehand. Definitely, they will have to pay for those things because I am using money to preserve these things tissue, all those samples and all this thing over the year

**Interviewer-what are your sources of information**

R4- I read a lot

R3- reading and exchange of information with other colleagues around the world

**Interviewer-** I want to ask us about our awareness and understanding on brain banking

R2- brain?

**Interviewer-** yes

R2- no more biobanking

R4- it’s a component

**Interviewer-** are you aware of barin banking

R6- I, I am aware, like the mentioned that time of Tai Solarin and Micheal Jackson

**Interviewer-** what is your perception about it

R6- the thing is they don’t want those people to go with their intelligence and talents, they believe they can preserve it but what they want to do about it, I don’t know, maybe they want to give it to somebody else or maybe they want to replicate, if there is a way to replicate the intelligence, I think I heard something like that, Oyinbos do it

**Interviewer- are you aware of any policy or law guiding bio banking**

R2- I am not aware of any but I want to believe that in these modern days, if that is not captured in the law that guides medical profession, then legislation can be made but in making legislation to guide it or protect it, we must also take into consideration our own prejudice, our own society and the law must be done I such a way that allowing it, it will not be abused because in this part pf the world, the major problem I have discovered is that our problem is not about law, our problem is about ourselves. We seems not to love each other in this….., because there are some things the western world have taken for granted and just believe it cannot happen that still happens here, you know, you are in UK, you are in America, you just take it for granted that you are protected, you don’t need to be walking and be looking who is coming behind, you sleep with your two eyes closed and you don’t see policemen everywhere carrying gun but you just know, the consciousness is just there for you that you are protected. Why? Because naturally they love each other, they don’t see putting somebody to suffering as something that should happens, you don’t hear of kidnapping, you don’t hear of ritual killings and what have you but in our part of the world, would someone have thought that somebody would be stealing pant to make rituals. It couldn’t have occur to any lawyer to draft a law that would say stealing pant is an offence because that could not have come to our consciousness that it could ever happens, so in Nigeria a lot of things happens that are so inimical to our growth but this one is something that is science based that could be used to prevent diseases or what have you or help us, therefore in bringing the law, we would look at it from that perspectives but we also must know that shey you **…….not very clear….but SOUND LIKE** don’t solve part that somebody that want to perform rituals will come and buy, how do you safeguard that? that must be in the consciousness of the drafter of such law, I am not aware there is a legislation for now but there can be one which if this policy is shown to the government and government accepts, then you can go back to national assembly and draft a law as long as it is not against the constitution of the country. I am not aware, maybe those of you in the medical science may know but I don’t maybe there is any law that protect it.

R4- there is a policy and the policy is crudely implemented, if somebody dies, before any of your body parts could be accessed, there must have been a written document, your will that I wish to donate my whole or body parts, macro or micro anatomy of myself. Two, this particular specification like Babcock, I know some people that have died and have donated their body parts to that institutions, so, it is like I want to donate my body parts to the anatomy department of the university of Ibadan for research and education and…, so those are the policy on ground. Outside of that, there is none nationally

**Interviewer- can you explain what you understand by precision medicine**

**R3-** precision medicine, accuracy and in Yoruba**: ki nkan se dede, ki nkan je dogba dogba pelu nkan to fe se, ta ba ge Iho kan to je 100 hundred metres, nkan ta fe fi replace e, ki ohun na se deede 100 hundred metres(accuracy, if we dig a hole of 100 hundred metres, whatever we use to replace it must also be 100 metres)** so we are striving towards making sure that the precise medication, treatment for whatever is what is given to a particular disease condition not speculations, for some times what we have been doing before the advent of diagnostics machines is speculations, you assume this but right now, with investigations with endoscopy and many other ones that allows ones to get to the exact point where the problem without moving through corners. That is precision, I am very aware of that and in other part of the world, they are almost there and I hope that we would be there in Nigeria.

R2- what I do understand with his explanation also is that precision is opposed to trial and error because a lot of times people don’t really know, let us assume, this may be this, let us try this, let’s try this and I think that is majorly the problem we have in this part of the world and that is why this rich people go abroad for treatment because they don’t even the best of the teaching hospital in Nigeria, they say they just want to assume, they are not sure but when you go to UK, American particularly Germany, they will tell you if the life in you is 2% and you manage to get to a German hospital, the possibility that you will live is very high because they will not just perform anything on you, they go straight, they identify what the problem is and luckily enough for them, I think they have gotten enough equipment that they can just set on you that will read the whole body and they will be telling this what is wrong with the brains, eye, this is what is wrong with the lungs, with the hearts and if you are able to get what is wrong, then giving you a precise treatment for the diseases becomes easier. So, I want to believe that Precision is opposed to trial and error

**Interviewer- what do you think about the concept of Precision Medicine? What is your perception of the concept?**

**R4-** when we talk in terms of precision medicine just as it’s been mentioned, it is not beating about the bush and there, you have promptness promptly, efficiently and accurately, going to where the problem and solving the problem and the concept ma, today, medical world in Nigeria or Africa as it were, we have well trained personnel but the facilities is not supporting. Now, for us to diagnose precisely, since the facility is not there to support your intellect, it’s like you have to start guessing this and this and that, so that is the problem on ground. Now, the concept is there in the book, the concept is there in practice but the actualisation is not what is on ground, the resources is not there to support, the political will is not to really back you up. As it were, we have very few government hospitals, who can really go straight to say we want to practice precision medicine but we have a lot of laboratories, some few days ago. The last ward round that we have, we had a ground rounds, there was this company that came to advertise some of those investigations that we……, these are high stuff investigations that we can call precision Medicine but unfortunately, there are none available in government hospitals, so, except you send those patients out to go and access. That is where the problem is

**Interviewer- what are the demerits of this precision medicine. What are the demerits? does it have demerits**

**R4-** it does have demerit, whatever has merit will always have demerit, precision medicine, the demerit are very few, one, for those people who capitalize, coerce and extort patient, there wouldn’t be any ground for them. Now, it is like I am seeing a patient, I don’t know what it entails, for me because of precision medicine**, I** will start beating about the bush, pay this money, bring that money, we want to do that investigation, we want to do this investigation, a whole gamut of investigations and I am trying to enrich myself indirectly through that but in precision medicine, there is no room for that. Two, in precision medicine, everybody will have to be on ground, you must to be worth your salt because once the patient come to you and say I am having this and you say it is malaria, the patient can go underneath, take his phone and google something under it and send the information to another clinician elsewhere across the world and that person will come back with another diagnosis, so there might be clash of opinion, ohh, this is my primary manager, he says I am having malaria, I sent information to Ghana or to Germany and that one says it is another thing entirely, so there might clash of opinion, so in precision medicine, there is no primary owner of a patient, in precision medicine, information does not resides with one person, it is a global thing because whatever……., if you key in an information into the system about a patient and all that , trying to arrive at a diagnosis, all other clinician across the world they have the same information and they would put in their diagnosis, so it is not the one that is managing me that is the only owner of the patient, all other people have information about this same patient , so there is no confidentiality

R6- merit: it can be time saving since you don’t have to do multiple test before the diagnosis is done, the other thing to add, he mentioned the issue of cost the other time, as soon as he mentioned it, something came to my mind, truly the way you painted the cost, it can be less costly but if you look at it from another angle, it can become costly, let me give you an example, Afe Babalola University (ABUAD), I had something about their hospital, they were to import some medical facility for the hospital and they brought the machine and it got to the time to clear it at the port in Nigeria, can you believe that the money that is being demanded for clearing is more than the money that was used to purchase the facility, eventually not all those facility got into Nigeria, some had to be taking back, imagine if all those facility got into the country but then it will be at high cost

**Interviewer- do you think this precision medicine is important in Africa**

**R2-** I think it is important

**Interviewer-** so that we don’t continue to kill ourselves unknowingly or rather we don’t continue to die prematurely, you see, research has shown that not everybody that died, died before their time, a lot of things that are causing death in Nigeria, let me limit myself to Nigeria now, are caused by Ignorance, ignorance of the unknown, you see, I was discussing with a doctor and the question I put to him is there no other way to this? He said no, because I believe that this precision we are talking about can also leads to surgery, if they discovered that this your problem is not about medication, what you need to do is operation, let’s just take it out and you will be okay, if the doctor could…….., you know as we always say, you don’t lie to your doctor, you don’t lie to your pastor, you don’t lie to a lawyer, so if the doctor that I have confidence in could be sure because sometimes doctor may not be sure, if he says I am 100 percent certain this is the solution, then you don’t need to doubt him but a lot of time, we don’t have access to such accuracy and a lot of time when you go into herbal medicine/ traditional medicine, they don’t really have…….., even when you are drinking Agbo……, I remembered when we were growing up, Agbo doesn’t even have quantity, you can drink as much as you like but you have discovered now that too much of everything is bad, no matter how good that thing is, even eating food, you may be enjoying it but it is everything you enjoy that is good for your body, before we don’t have all these things but doctors are now telling us.

Let me say that when I was in abroad, I discovered I was healthier than when I was in Nigeria, I was asking myself could it be because of the kind of food I was eating. Honestly, I want to be sincere with you, food is number one, environment is number 2

**Interviewer- can precision medicine be applied to stroke disease**

**R2-** let doctor answer that one

**R3-** I believe it should be applicable to stroke disease since we have major predisposing factors and again presently we have the 4-hours window which they can use the blood busters for Ischaemic stroke which the diagnosis can be done early and you discover where the clot is in the brain, so before you start losing tissues, if you can bust that clot and restore blood supply to those places, then the person will be as normal as possible, so it is going to benefit…, because stroke in particular, the disability and the permanence of the disability is killing and disheartening, a survivor can tell you what they go through, your life has changed, so if you have medicine that can restore you back, that can reverse the process, it is the best thing that can ever happen to humanity

**Interviewer- are you aware of any policy or law guiding precision medicine**

Respondent- shakings of head

**Interviewer- what do you understand about brain donation for research purpose? How many of us are aware of such**

**R6-** well, I am hearing one for the first time which daddy mentioned, you know some people……

R1- how can you donate your brain?

R7- you write it in your will “don’t buried me with my brain”

R6- ……for those people who have terminal disease

R1- we, Africans don’t do that, you don’t want any part of your body to mess

R2- cultural that is what we are talking about before

R7- Africans

Respondent- everyone laughs

R1- you can wholeheartedly and you go back just like that

R6- so, for those who knows they have terminal disease and they will soon be gone, you know, they might decide to will their brains for research purposes, when daddy mentioned it the other time, I was thinking the donation was made to be given to other people, you now mentioned that it was given to the hospital itself for research, so meaning it is for educational purposes and it is a good thing

R3- to me, I am not…. I think donating, it’s not really African, but maybe education and awareness could be changing things but I also still believe that there would be conflicts between the family and the will, they can contest it…..

R2- they would have even buried the man before they read the will

R3- ehen

Respondent- loud laugh from everyone

R2- unless you who are at the forefront can tell us about how far we have moved with donating the body parts in Africa and as against our cultural and religious belief also

**Interviewer-for those that are aware, what is our perception of the complexity of the procedure**

**R2-** you can simplify it, there is nothing complex about life, it is about the society, I believe this kind of things you are starting can simplify what we think is complex, then we just a lot of awareness, talking to people, educating the people, as a matter of fact, particularly those of who are quite religious like my daddy here(R1), I am a pastor(r2), he is a pastor(R7) and I know many of us go to church or mosque, so we really……, you see ,those things, those our believe, I am beginning to understand that some of our believe, some of the thing we even preach, they are limiting education, that is why you discovered that if you are sending your children abroad, if they are not well groomed before they leave Nigeria , in the next few month, he/she will call you back and say Dad, continue with your……, but you see Christianity, Muslim or whatever still have their own place, because to me, Christianity in particular which I am at home with is not against science, Christianity is not against education at all, in fact as a matter of fact, you discovered that most of the schools and hospitals that we have all over the world were started by missionaries, so why should they be against anything that promote all this knowledge but it’s awareness, awareness. Daddy is talking about use of codeine many few years ago but few years ago, most of the crime we are talking about were not there, there was no kidnapping many years ago. And again in year 2019, I can say from my experience the uneducated in Nigeria today are more than what we had in 1960, we have a lot of illiterate, go to our garages, those of us who are in politics, go to political campaign, you will see thugs they will outnumbered those who are reasonable at the gathering

R6- then seeking the consent of the person who is donating is very important, so that the lawyers can have something to use when the family starts making trouble, so it is always good for the people donating to give their consent

R2- to give their consent in written form before they die

**Interviewer- what are the other benefit of brain donation that you think of**

**R1-** is it in Africa context or…..

**Interviewer- yes sir**

**R1-** I think, these one is being practiced in Europe, not in Africa here, walahi, I have never heard it

**Interviewer- if we do it in Africa, is it a good thing, what is the benefit that is there**

**R1-** I am telling you we are importing disease from there to Africa, so many diseases, we are having today is imported

**Interviewer-what are the misconceptions that we see**

**R2-** that is part of what daddy is saying

**Interviewer-any other**

R1- my own challenges about brain donation is how do you make use of…... they are something that is still mysterious to me. I am not against it but I am just looking at…….not very clear but think he said practicality of it….. Because brain is still alive, you want to open somebody skull and put another brain there, I don’t know if the person can still live, personally….

R7- it may not be…. For study

Interviewer- it is for research

R2- so what are you going to use it for

R7- it is just for study

R2- you want to use for somebody that is still alive

R7- it is just for study

R2- what are you going to study in it?

R7- many things

**Interviewer- a lot, just like any other part**

R1- if somebody does that, will go to hell straight

R2- let me correct that

**Interviewer- it is his personal opinion**

R2- it is his personal opinion, I am not saying he is wrong, he has said it, if somebody says after I am gone and you put it in written form, in sacrosanct, it must be carried out because it is his will. Now, on the other side….

R1-can you do that

Interviewer- it is my next question

R2-…….before you go to personal willingness, I want to say this because this is faith based, if the bible tells us that when we die, our body goes to the ground and rust away there, that what goes to the maker is our souls, then of what use is the body, what we make the body go to hell again, if the body is already in the ground and that partly answers daddy assertion. The bible tells me that after death, it is judgement, what happen to my body after death could not determine where I go. That is my belief after I have gone but then writing it down could also have happened before I die…..laugh……, I don’t know if that will constitute sin, I don’t know

R4- brain banking, it is not that high technology, after death or prior death, there must have been a written will and a video coverage, two basic things: a written will and a video coverage and in the video coverage, there would be a lawyer probably and a witness, then a representative of the recipient, probably a research institute or whoever is representing that body to be the signatory there and that makes it sacrosanct. Then after death…, at the point, there must be somebody to certify that this person has actually died clinically, so there is no sign of life, after that, they go ahead to open up the cranium, the brain box and the brain is carefully removed…..

Respondent- laughter

R4- not the head, it is just the content, the content is removed and there is ….at the base of ……, this the brain is not just the content in the cranium, it still extend to the 4^th^ Cervical, so there is base of the brain, it is carefully severed off and the whole content is put in formalin. Now, going to it use, education benefit, you can use it for research, you can use it for education, you can also use it to monitor. Now, let me explain monitoring, the older you grow, the less the weight of the brain in proportion to the body, now a small child at birth, the brain weigh about half of the total body weight of the child but as you grows, the brain body proportion not that it shrinks but it becomes larger and larger like that, then you can use it to monitor. Then, in disease state or disease progression, you might want to see if there is an increment or decrement in the weight of the brain or in structure of the brain in relation to the disease process, so those are the things you want to see, so basically, it is for research purpose

R5- now what happen, we are Africans, and the owner of the body agreed that his brain should be taken for research purpose, the family is aware. If the family takes him away and he dies, you don’t know where the corpses is, what happen?

R4- now before it leaves the hospital or office as it were, this surgery must have been performed on the cadaver, not the living body again but if the family takes away the body in the pretence that they want him to be…, they can be charged, they can be challenged in the law court, we all know that but to the perception that we come whole and we go back whole. Sir, for men, we all read the bible, there was this instruction to Abraham that he must perform certain procedure on all his male. Now, that certain procedure is it not part of the body, where does it go, so it means certain part of the body can be done away

R7- sorry, my contribution may be funny because barrister started it, when he was mentioning the part of the king in Yoruba land. A further deep research into our traditional rituals especially surrounding the kingship in Yoruba land will tell you that what you are discussing, they are carrying it out “Oba ki gbe Ori re losorun”(Kings don’t take their head to heaven), go and find out. Like in my hometown because we have just talk about the issue of the heart, you know what I want to bring out is this, if for certain belief understanding, they do that, for a more better understanding for education and for advancement of medicine and health. I think our people will be able to do

**Interviewer- would you be willing to donate sir**

**R7-** YES! You know that is what I am talking about, I have answered you, I don’t need, I won’t be going to heaven with this body, that is my believe, those that BOKO HARAM killed

R2- I don’t need it, in addition to what he said, a very senior person, former governor once told me, he said have you seen where they buried king, have seen a king in casket and they are saying this is the king we are going to burial ground. He said do you know what happens to the body. You see the problem we have in Africa is that we don’t develop our tradition, we don’t take it beyond where it was several years ago, ask why did they do it, we just discard with it because of the new things and new whatever and we think this thing they are doing is bad. It may not be bad after all

**Interviewer-Culturally, what do you think could be the cultural belief of brain donation for research purpose?**

**R1-** I want to ask one question? What do you mean by **“SAINT?”**

**Interviewer- let us ask the Imam and pastors**

**R1-** because you now ahh……..silence ……..

**Respondent-** everyone laughs and was curious to hear what R1 want to say

**R2-** Baba say what you want to say, the word of the elders is the word of wisdom

**R1-…… I want to ask one question, what do you mean by Saint, because you now you are……**when you hear of Saint Augustine, Saint Paul, Saint Peter ahhhh….laugh….., when they die, they died natural death, you know they were buried in the church compound, so after about twenty years, they opened their grave, to me I have a lot of things in this Abeokuta, when this your governor was pulling down people houses for roads which water can wash away in one day, you know the time of Noah, you were thinking yes, the dry season is gone, not knowing that the earth is going to be buried 31: 23

**R2-** let me **ask** Baba**,** are you opposed to any part of the body being tempered with?

R1- uhn ahhhhh!!!, by the grace of God, I will tell you something, when I was in London, my wife had Ulcer, I was in another polytechnics, that was 1968, when she was trying to go to work, she collapse, I wish I was a whiteman, what can I do now. She was taken to the hospital and the doctor said they will do operations but the doctor later told me that we may not do operations but that this our Nigerian women they like work, they can work for money till night for a week without rest because of money but if she can keep away from work for a month she will have no problem. The doctor did not do the operations but gave medicine and say don’t do this, don’t do that

**Interviewer- what are the factors that inhibit brain donations. Could it be influenced by peer value and parental influence?**

R2: must we finish this discussion today

R3: to me peer value or parent or whatever is still within the context of the environment, culture and religion, whatever the submission is, whether we believe that, that thing could be done. I think most thing now is faith based, even the cultural aspect of it has been watered down but the issue is strongly based on faith that is it allowed that a part of my body can be taken away before I am buried. If it agrees with your faith, I don’t think you have any problem. So whether we are peer, whether we are professors, it has to do more now with faith, denomination that I belong to, how can I reconcile science with faith

**Interviewer- do you think that familiarity with medical research and knowledge of where to do can be a factor to promote brain donation**

**R3- yes**

**Interviewer-what are the factors that promote brain donation**

**R4-** monetary, if it get monetized

R7- off course, money can promote

R3- knowledge, for people who have travelled and have seen where it is happening and sometimes it gives your ability to resolve your fevers,

**Interviewer- what do you understand by blood sample donation for genetics research**

**R4-** donating blood or volunteering your blood sample to be used for research purposes

**Interviewer-**what is your source of information on that

**R4-** internet

**R2-** I got my information from one of the doctors here

**Interviewer- what are the uses of blood sample donated for research**

**R2-** I believe they use it to know reasons for a particular illness or medical conditions

**Interviewer-what are the cultural and religious belief associated with blood donation for genetics research**

**R2-** well!!, people could be sceptical but with education, enlightenment because they may not even even know anything about it anyway, so if I don’t know why you are taking my blood

R3- I think the challenge trust, do I trust this people that they are going to use it for the purpose for which they are asking it for, especially in this environment, trust is a very……not clear…, you have cases in which medical doctors conniving with people to perpetrate some evils. That is the basic underlining issue, trust is a very strong….not clear…

R5- and in addition, the Ajeri(Jehovah witness), they are strongly out of it, they will never accept blood transfusion and they will never give out

**Interviewer-are you aware of any policy guiding blood sample donations for research and storage**

**R4-** policy and ethics: now for you…. in the course of your training, you must have been taught to solicit, take, store and dispense, so all those pathways, you must have been trained on all those things, so if some issues crops up, in the law court, what they will demand is does your ethics trained you on this or this but there is no definite law that say don’t do this

**Interviewer- share with us your opinion and thoughts on blood sample donation for stroke genetics research. Will you be willing to be involved in such research?**

Respondent- ALL yes

**Interviewer-what do you think as barriers that could hinder your blood sample donation for stroke genetics research**

R3- personal barriers. Trust

**Interviewer- could family member be a barrier**

R7- not at this level

**Interviewer-religion, peer values, culture**

**R7-** I think most essentially it is sincerity of purpose and that has to do with trust, if I know it is for this purpose, for study….

R3- from what it is meant, if it is not going to cause me any harm

R2- off course by our law, there is maturity in age, once you are beyond 21, you can do anything by yourself, so whatever I do now nobody………

R7- that is what I mean by not at this level

**Interviewer-so what do you perceive as benefit of giving blood sample for stroke genetic research that could promote your willingness to donate**

**R2-** now if I get the question, those of us who don’t have stroke and by the grace of God we will not have, did they need our blood for anything, is it not the blood of those who have stroke that they will need to know really what caused it

**R4-** for most of the diseases that we are trying to address, we call them non communicable diseases, hypertension, diabetes, it’s like a gun that have been loaded and there is something that will triggers it off, everyone has an element of hypertension, everyone has an element of diabetes within, so that is the cork gun but it takes one singular to triggers it off and that one singular thing could be exercise, disease…….

R3- that is why they are called it apparently healthy because you are not sick now

**Interviewer- what can you say about your family member or other member of the community willingness to give blood sample for sample for stroke genetic research**

Respondent- awareness

R2- if they are aware, maybe they will be willing but for those that daddy mention, even if they are aware, they will still not do

R7- that is religion

**Interviewer- for those that information can help, how do you think we can get this information to them?**

**R3-** I think to me, that is where giving us information… progress of research outcomes, success rate, infact, major challenges, I have somebody now who is contemplating a spinal surgery and he wants to do it in Nigeria, he is trying to check what is the success rate of spinal surgery, particularly in UCH, which will give him some level of confidence before he subjects himself to that…, otherwise

**R2-** something is coming to my mind, is it possible to study as a subject in the school, maybe in secondary school, just the way we studied biology or you make it part of the study of biology because knowledge is power, Nigeria is complex now that if you say you want to go to town for awareness campaign, it may not be so practicable. How many people will listen the way many of us have sat down here since morning? But if it is a subject that is taught, it is not whether the student likes it or not, he must sit down in the class and learn it…..

R3- on that, that is why most of the time, they make it local content, and they transform it into the language, as much as it is not done in English only, if you tune in, they are some Yoruba programme that you can phone in, you will notice the rate at which people call in because they belief in the language that is spoken, the words are communicated in their language, so what we need to do is to make it local content so that they can understand the benefit

**Interviewer- do you think mass media could be helpful**

Respondent- yes

**Interviewer- Husband consent for women?**

R7- religious leader…

R2- if you get the Baale’s

**R7-** if you work with religious leaders, you will go far because I remember the time that the malaria thing, they had to work with the religious leaders and they confessed that they have come to realise that for pastors and Imam, they work better than radio and television because when we were taught, we were given lecture on malaria, what cause malaria, symptoms of malaria, prevention and its treatment and all those things that we were taught, I made it two Sunday sermons because my religion as a pastor is to be well with my spirit, my soul and my body

R2- as at today in Redeem, we have been compelled to go and do comprehensive medical check to the extent that the church is giving is pastor 100 thousand naira to help. So like he said, if you can partner with religious bodies and makes it vigorous so that we takes this thing to them

Interviewer- family consent, donors group

R3- Education, liberation, individualism, family is not….., well unless the person doesn’t have the ability to understand, read or write but I think individually, people can make their decision once you are adult

**Interviewer- tell us what you know about informed consent**

R2- informed consent? Consent that is given out of knowledgeable, for the fact that you know, not that you were compelled or you are forced or you are coerced. When something is informed, it means you have been told what it entails. You know what you are doing, you are not just giving the consent out of any inducement, it is done voluntarily, it is done by knowledge, it is done with full information at your disposal

**Interviewer- so what do you know about consent process for genetics research**

**R2-** I don’t know

**Interviewer- does anybody know, consent process for genetics research**

**R3-** is it different from every other consent process

**Interviewer- Just tell us the one you know**

**R3-** I know the one he has described, if you want to participate in a research, they tell you what it is about, they now ask you whether you are willing to participate.

**Interviewer- what type of informed consent would you preferred. We have Broad, restricted, tiered, and dynamics informed consent**

**Broad- as the name implies, it cuts across, I am giving consent to do anything,Restricted- I am giving consent for this. I am restricting my consent to this particular, Tiered- is like in layers, from this levels to this level, Dynamics- as the name implies. Free flowing**

**Interviewer- so which we you preferred?**

R2- it depends on the situation, my consent will be given because certain information is at my disposal, maybe they explain to me that if you do this it will help humanity and what have you. I may ok fine, go ahead but

R7- to me broad will still achieve more because we are talking about culture…. Ahn! Where did you about before? Has it ever happen in our land before? Abroad one will still help people to now that something like this is going

R3- it depends on the situation, probably their might be a kind of research in which you get to certain layers, you might really not be interested anymore, so it depends on the situations

**Interviewer- what are the persons that you need to involve before participation in genetics research**

R3- if it is something that will not affect anything, I can give without the consent of anybody, I can give….if I am married, my wife is the next person to me, if I am old, my children can be my witness particularly this brain, my children, my family, my brother too, my extended family because that one too might come and challenges them and say you cannot buried our brother in parts

R7- for me, my pastor also will be included because those are the one that actually perform the burial ceremony so that they will not become surprise and ask why they cut me on the head……laugh….., my pastor should be carry along, this is what I have done and for this purpose and so on

**Interviewer- data use in the incident of death and why**

R4- data that is generated is of no use if it is not use and in such instances, it could be use across board either for research, for education or for anything that **is** applicable and since there is this issue of anonymity for samples that are taken, it is always silent on the name so that they wouldn’t say say this belong to this or this. So data use can be across board, unrestricted

R3- in the interest of humanity, as long it is use in the advancement of the interest of humanity

**Interviewer- what will be your support for generic consent for community**

R2- that will not work, you know you are talking about my body, you have to be personal, and person that you are going to work on must be the person that will give the consent, anything that is general would not even work

R3- it could backfire

**Interviewer- what are your opinions on storage of blood sample and blood fractions**

**R4-** blood storage in Nigeria facility we have not yet gotten there, except it is improved on, the facility that we use is some kind of third generation’s facility presently, where you depend on generator to power to power storage facilities, where there is blackout, where there is this and that. In most places 1^st^ grade facilities, they don’t depend on public power supplies and all that, it is probably solar generated or nuclear generated electricity and all this this things. What powers, what drive our facility is what matter most, so we have to be aggressive in addressing that

**Interviewer-tell us what you know about sharing of data, blood or blood fractions, brain images as well as brain tissue sample. What do you know about sharing of data?**

**R4-** the hind thing now is data pooling, people pool data and you just subscribe to that particular organisation and access those data. For instance now, you mention brain banking, blood fractions and blood storage and so, now a particular agency can be in charge and others whatever information they generate, this could be shared, instead of multiplication of all this facility all around the places. Like if you have some place in UCH, all the teaching hospital in Nigeria can access those data, rather than each hospital or tertiary institution putting in place their own personal or institutional…, so it is better. It is the hind thing now

**Interviewer- what is your opinion on sharing of data, blood or blood fractions, brain images as well as brain tissue sample locally and internationally**

**R2-** the essence of knowledge is to pass it across, so you can shared it either locally or internationally

**R3-** you need the assistance of other expertise in the world, we are doing that already, people want to go for surgery abroad, you have passed across all the images, investigations, so that they can make informed decision about what they want to do.

**Interviewer- are you comfortable with that**

R2- yes

R3- it’s even beyond our abilities, you don’t have an option in some cases than to allow the data to go

R2- and most of things we Google and read this days is out of research and we are benefitting from them, we should also be ready to give out

**Interviewer- tell me your opinion on commercial or non-commercial use of stored data, blood or blood fractions, brain images and brain tissues**

**R3-** my opinion is that people that are donating this data for free, while we organisations be selling it**.** Is it ethical issue now or trust like we said? So, I believe if for free, unless you are buying the data, the information from those who are given it to you, which is purely business.

**Interviewer- share with us your thoughts about return of individual research results and incidental finding. What are the ways you think one can receive the result of genetics research**

R3- publication

R4- I think it is logically relevant that to whom something is taken, you must give a feed back because, it is like a asking a question or taking tissue sample from someone for investigation, the result or the outcome of that investigation must be fed back, so it is a two thing, it is like communication and that person should be acknowledge

R2- by mail,

**Interviewer- any other way, what phone calls, letters,**

R2- messages, whatsapp but letter must be confidential, we are talking of medical report now, which I think the medical profession always makes the findings for individual very confidential. I believe you don’t give it to a third party

**Interviewer- which way will be your most preferred means of communication.**

R2- anyway as long as it is confidential

**Interviewer- would you want it from a clinician or researcher**

R3- at that level it doesn’t makes a difference, unless there is an underlying statement that needs to be communicated, probably there is an adverse effect of the findings and there is a need to have a way of disseminating the information to the person, if eventually you went for a study and you discovered somebody has cancer or whatever, how do you pass that information and the person is not aware, just gave a free donation and it come out with something that is not……..

R2- you first of all call the person for counselling

R3-….., especially the issue of HIV/AIDS, people are just donating for test and a negative outcome which the person is not even suspecting or thinking about

**Interviewer- apart from confidentiality as one of the ethical issue to be considered, are there any other one to be considered when returning results**

R2- you just mention something that maybe if someone has a life threatening or whatever, there is nobody that will hear of the day of his death, you don’t take such things likely, I am thinking for example if somebody has HIV for instance, Cancer might not be a big issue, HIV, you don’t just send it to him by mail, you are HIV positive, you need to call such a person and counselled: what we find out is wrong with you is quite unexpected but then it happened, you have to take it, This is our findings, off course the person might first faint in your presence but off course you are prepared for that and make sure you can manage the situation

R3- and probably those kind of research might need during the process of getting informed consent to have the phone number of a relative or third person, so that, that information can be first of all…….., I don’t know it is going to be, so that you can lessen the burden or you can someone that will support the person when the information is been disseminated

R4- in the medical parlance, you don’t give an information without giving an intervention, somebody is having HIV, just don’t break the news, you go the next step to give the intervention option, oh, you can access medicine, you can access this, you can access that, you have to give options available for that person so that it can helps in buffering the depression that the person may likely go in to

**Interviewer- explain your understanding of bio-right**

**Respondent- everyone was thinking to get the understanding of bio-right**

**Interviewer- how much control should an individual have over how their biological specimen will be used in research?**

All respondent- 100 percent

**Interviewer- what right should an individuals who provide their specimen for research have over their specimen?**

R4- 100 percent right. Absolute right

**Interviewer- absolute right**

**R7- yes**

R2- let me get the question right, I have donated this for research, are you talking of what right I have over the result of the research

**Interviewer- yes, over the specimen, how they are use in research**

R2- that would have been told me before I donated it, so once I have surrendered it , I am surrendering too you that you will use it for the purpose for which you ask it for, if you go ahead and use it for different purpose, I will not know but I can sue you for it

R7- that means you still have absolute

R4- that means you still have absolute

R2- in proportion to the consent I gave you and this bring in a lot of issue but we don’t have the time. In this part of the world, a doctor dare makes mistakes in the develop world, you will see what will happen to him but here……

**Interviewer- how should autonomy right be best balance with societal benefit that is derived from use of human specimen in research**

**R3-** that is what I was trying to raise ab-initio that if it is going to be in the interest of humanity, we would balance it and it is not going to cause me any harm in whatever way, neither will it cause my family or anybody surrounding any harm, then I think they should be use

R2- let me just say this, how I wish we can radically change our perception about things, this thing will benefit the society, we have a lot of cultural limit or cultural whatever which if education is properly disseminated, this will be taking care of, Nigeria is no more religious than American, if you go American and see the number of people that comes to worship on Sunday, you will know these people worship God but they have this understanding that your faith should not debar science. We have a lot of issues, this autonomy and societal whatever, it is all about enlightenment, if the people get knowledge, and they have understanding that this thing have to do with my belief in God or not, there will not be issue but we still have a lot of enlightenment to do

R3- there was a book written by foresight for Ojukwu and he was asking a question that at what point did the white man left his counterpart black brother behind in the race to civilization and the answer is the white man and black man relationship with their God, the black man sees his own God as God of retribution, a violent man that is untouchable, that is reserved, that is why when we see mountains, we worship the mountains but the white man see his God as friendly, appreciable God, a God that allows you to conquer, so when they see sea, they want to see what it’s on the other side of the sea

**Interviewer- what is your opinion on governance and regulation of bio banking**

R2- our leaders should come out clean

R3- and they should be well informed, how much did they know of science, what they don’t know, they can’t give a clear policy

R2- you saw what happens to our president in the first half, when he said I have never been this sick,, he remembered the country called UK when sickness came hitting him, so that knowledge, they should bring it to….., our leaders should be up and doing

**Interviewer-what do you think about the need for ethical committee approval on future use of stored data. Blood or brain tissue research**

R4- definitely for every process, there must be ethical approval for it not to be abused

R3- yes they serve as protector of the right of the client and o the researcher

**Interviewer- how about the need to set up a regulatory body/ board**

Respondent- good

R4- most university, most teaching hospitals, they have ethical committee in place but that should be a board that will regulate even the regulators

**Interviewer- what should that be domicile**

R3- that is government

Interviewer- explain possible intervention for implementation of bio-banking

R2- grant from government, NGO,

**R3-** They should also be sure of how they do it, so people should go and acquire knowledge, they should fund people to get the knowledge and the facility that will be used. Otherwise we have …., a typical example we want to start **stays,** they brought in all the machines without the manpower, so what happens to the machine, they start selling it one by one, so there must be a balance between the technology and the manpower who will implement it

**R4-** another thing is awareness even among the medical personnel, basic awareness, let everybody be on the same page. From the medical personnel we can start infiltrating all other fields, the religion, the economy or whatever but the basic thing is everybody in the medical field should have knowledge about all this things. Basic knowledge

R2- grant also form religious body because a lot of time, we waste our money on what are not useful. Somebody was telling me, a member of CAN, he said CAN wants to build secretariat, they went to one of our GO and he donated 200 million to them and that is just one of our religious organisation. We need to get our priority right in this country, such money could be used for research and which we save lives, whether we like it or not, no matter how much you believe in God, you will die one day but let us see whether we can preserves lives through science. Me. I am so much….., but my problem is about implementations, Nigeria we can do research, we can talk, we can make a lot of noise but when it comes to implementation, delivery, a lot of times, it becomes zero

**Interviewer-you mentioned awareness, I want to find out suggestion you have that can help raise awareness and improve attitude towards blood sample or brain donation for research**

**R3-** we have route through which people access information, one of them……, the major barriers that we have been seeing now is culture and belief. Those are the two major barriers, so who are the key people in this area, if the religious leader are well informed and they understand what it is, sometimes most of the times, whatever they tell their followers , they believe unquestionable

**R4-** baba mentioned something the other times, baale mention saint the other time, we know how our environment is, first to really make headway, it takes one person to really do the extra ordinary and when one person does that, the thing just blossom, it takes one person to do something. The Yoruba man will say “enikan lo ma n la ona opo eyan lo ma gba” it takes one person to lead the way and the person becomes saint and others emulate. If we can have people to set examples, act as pacesetters, others will follow.

**R7-** I think we are dealing with public health, going through the rural communities because some of this things we do on internet, radio, television, there is a limit to its reach, there is a limit to its reach to be sincere, the people don’t really know some of the thing we are talking about. I think if we can go to the rural dwellers or also through their leaders whether religion, traditional or political. Then organise seminar, teach them, let them ask questions. You know I did something……, let me say this as a pastor, in 2016, the formal church I pastor, for three Sundays, I brought medical doctors to come and preach. That time, one Sunday, hypertension, the other Sunday, diabetes, do you know the funniest thing, with all the expository sermon I have been preaching, my people rarely ask question. They don’t ask question on why is Peter life like that. No. But on this very Sundays, we normally supposed to close by 12pm but nobody stood up till 1pm, they want to know the way out. I believe if we take such thing to the rural people capturing the leaders, the religion, traditional or political leaders and so forth, it will go far than all this….., all this methods only help the educated ones

R3- to a very large extent where there is primary health care, people do go there to access information at any point in time, those route to, so what you need to do is to revolutionalise in a way of making sure that even the health care, health attendant, everybody like you said have some element of information about this things, so that when you sit down with them, there are sometimes that the persons who will have more influence might be a wars orderly over the people in that community that even when the expert talk to that community people, that person can go back there and say don’t answer them, they want to do a different things o, they are selling it to the white ni o, don’t say I am the one that told you o, I am just informing you ni o. the person who has earlier agreed will just come back again to say he is not interested. So, every one of them must be enlightened enough to know the merit and demerit of this thing

R1- a trusted leader

R4- there is another crop of individual gatekeepers that our health systems has not come to term with and those are the traditional birth attendant, skilled birth attendant, traditional healers association, a large percentage of the people that comes to the hospital, they don’t come early enough because they have first gone to those people, patronise them, then they get push out, now these are crop of people that we have not co-opted into the health system and by and large they are not doing the health system any good, so the earlier the better the patient/client comes to the hospital and report his/herself, the better for the prognosis ie outcome of the diseases. Now if carried this people along, we empower them with enough information, though our people will go to them, now with enough information, they will be able to determine or they would be able to catalyse the patient reporting to the hospital early enough

R2- this may sound funny, get all this national road and the rest of them in the garages involved, they have a lot of followers and they have a lot of influence, please let see how we can them involve because they also need to take all this information to the garages and to the people we will consider as lower echelon of the society

Interviewer- thank you so much for your time.
