# Supplementary material for: Research participants’ perception of ethical issues in stroke genomics and neurobiobanking research in Africa
Source: PLoS One. 2025 May 6;20(5):e0292906. doi: 10.1371/journal.pone.0292906 (PMC12054916; doi:10.1371/journal.pone.0292906)
Supplement: S3 File — (ZIP) [file pone.0292906.s003.zip › Files for PLOS ONE - updated March 2025/FGD_Stroke Free Controls_Abeokuta.docx]

FOCUS GROUPS: # PARTICIPANTS___6___ . # MALES__4__ # FEMALES___2____

**Interviewer: Good afternoon once again, you are welcome to this discussion, let me start by asking the first question straight away. I will like you to tell us what you know about genetic research, what do you know about genetic research? in yoruba,  so awon ti e mo ni pa iwadi gene. when they say they want to do a research based on somebody's gene. won fe se iwadi ni pa gene, ki ni e kon to wa so kan wa, ki le mo e pa e, se e ti gbo ri?**

R2: Genetic research i say is just a study about someone's gene, probably if there are anything they can trace from the person's gene, i believe if they are trying to look for something in my own gene, they might have to look through my family line and stuff like that, so probably there's a standard that..they are trying to also weigh my gene against. so that's what I think about it.

Interviewer: Thank you, genetic research..

R3: what i really understand is about the gene is what what i ehhh inherit from my either my mother or from my father

**Interviewer: what you inherit from..**

R3: yes, either from from my mother or from my father, or this one that has been passed..so what is.. what is doing me that started from them, so that why i understand about gene

Interviewer: Thank you very much, e se

R1: what I know about gene is what has happened to the father or mother…., what we eat or what we drink can also cause it

**Interviewer: what do you understand by genetic research?**

R4: it's just like ehhh a research, a check on your genetics as in maybe they will start from maybe from your gran gran parents, from grandparents to your own parents , if possi..if they are still alive to your own parents if they are alive, then maybe your siblings, research, gradually then you, it can even go to your own children if possible. that's what I know about genetic research.

**Interviewer: Thank you very much, do we have anybody here that have experience directly or you know others have participating. That have participated in this type of study before? Do we have the experience or have we participated in such study or we have someone who have participated in genetic research that we are talking about, have we participate in such study before or maybe you have a friend or somebody close or far from you that has participated in this type of research before? do you have anybody that has anybody? ko si? (anybody?) when we are now talking about genetic research as regards stroke, what comes to your mind? what do you understand about genetic research in stroke? we've talked about genetic research generally and all of us have shared what we know about it, now when we are talking about genetic research in the context of stroke, what comes to your mind?**

R2: A..ehhh what comes to my mind when we are talking about genetic research and as regards stroke is maybe we are trying to see if there's anything in a gene that can ta..tell us that this person has stroke or might have stroke or maybe trying to look through their family line if they have history of people that have strokes, probably there is something particularly in their gene that is causing that, so that's what I understand.

**Interviewer: genetic research, if we say we are doing genetic research that has to do with people with stroke, what comes to your mind**

R3: yes, what comes to my mind is you know is possibly for those one that have passed, is possibly for them not to have stroke and they may have diabetics, they may have hypertension and we know that these are.. these are causes of the stroke but if we want to make the research of those people that is having stroke , is possibly that it may be from the gene for them to have either hypertension or diabetes, for them to have this stroke but it may not happen like that in those one that has passed, they may have diabetics, they may have hypertension by not including stroke. so that's what i understand

**Interviewer: Thank you, if they say genetic research on stroke, what comes to your mind**

R1; anybody that will have stroke, if they check the person, he would have had high blood pressure and diabetes, so if they placed the person on drugs and he refused to use the drug regularly, that is when it results into stroke

**Interviewer:  if they now said they are doing research to know if the person has stroke through genetic research, what are they trying to do**

R1: if they say they are doing genetics research, though…., the person will first come to the hospital, they will check blood pressure, if the blood pressure is high, they will place the person on drugs, so the person will be using the drugs regularly and also visit the hospital regularly for checkup

**Interviewer:: do you have anybody that what to add.. what do you understand you understand by genetic research in stroke, do we have any other adding? I the absence of none, do you..**

R2: let me try, maybe people that currently have stroke thay will try to look through their gene, then also known sample s of people that don't currently have stroke to see if there is any correlation that can say ok this is really a pointer, that can use probably in the future for people that might... that are susceptible to have that stroke, so maybe they have... if they're going to be an early detection from their gene, they will be checking.. or taking something against it. so that's all I can add

**Interviewer: OK, thank you. what are now the benefits of genetic research in medicine?**

**. do you think there are benefits of genetic research? what are some of it?**

R2: ya ha.. ahmm i think i would link this to something like this vaccines we have, for example polio, the they already know that due to some particular reasons people can have polio within a certain age, so if this research.. what result will come out with will be able to help people to prevent or advert having stroke in the future. so if we know the caus..the causati.. causative agent then we can actually say ok, we can treat these people before it comes out, so everybody will have a better life, none will be falling from stroke, there will be... the productivity of everybody will always be up there every time, so there's no need for us to be afraid that people can lose their jobs or maybe their family because of Stroke.

**Interviewer: Thank you very much**

R3: it will give us a a direct medicine to use, not be parabulating, so we just.. so far that there's a research on it, there will be a guideline towards it

**Interviewer: do you think there is any benefit in carrying out genetics research in medicine. What are the benefits that can come from it**

R1: there is benefit there, there are some people that have it and they don’t know, they just keep walking about, the person may just hit something and fall down, since the person did not know but if anyone comes to the hospital and they examine the blood pressure, they will know if the person has it, so that they can place the person on drugs, that is the benefit there, the person will be using the drugs regularly, so that he will not have it

**Interviewer: who wants to add any other benefits for now or I should continue? Thank you. Let me go to the next question, what can you say about biobanking? what do you know about Biobanking? let me explain, bio is when you are talking biology, is like body parts, all of us know bank, we have money for bank, we keep something there, so when we are now talking about biobanking, its keeping part of the body that may be useful in a place. then we are now asking what can you talk about biobanking. what can you say about biobanking, when I say part of the body, we believe it's still living and its useful.**

R1-what I know about it is that, people will be coming to the hospital, his blood pressure will be checked, he will be placed on drugs and he will come regularly for check up

**Interviewer: Thank you, when am talking about biobanking, I’m talking about keeping part of the body in a place, either for future use or for research, have you heard about it before? what do you know about it.**

R3- the only thing we have... the only thing I heard about it is about blood, so we have our blood banking here that if they want to use, I’m..I’m.. working at dialysis, so any time that we want to dialyze and that person doesn't have enough blood maybe they check the blood and it's not enough we will go to ehhh blood bank to collect blood and pay. so, they will use it for the fellow, I only knows about blood but I haven't heard about the kidney or any part of the body being kept somewhere.

R2- what I know about biobanking is probably keeping some part of our body or organs alive for research purposes and also should in case somebody needs it for a replacement it can also be used. so it's also important we ..we look into that area, I know it's not really common in our side here in Nigeria or let me say Africa at large. for example i have someone that has to have his kidney replaced and it's a challenge, they have to try and move him to Asia because of that. so we have also heard about a lot of Nigerians going outside the country because we don't actually have it here. so, there is no point burying a lot of people alive with functional organs when they can be used for research work and all. just like you have cadaver for this..all these young doctors when they are in medical school, we can also have such for people for research and also to allow some people to stay alive.

**Interviewer: Then how does biobanking operate? do you know about the operations of biobanking, do you know the process?**

R2- I feel..the fear and the little idea I have about it is that whatever organ or part of the ..some that’s ..the body parts that needs to be preserved..ya that can be biobanked would actually be something that is not defective, which means the person has to be eating some particular kind of food, you understand that can make the body part to be of the right standard for medical purpose. so i ..i think..if...then also i have to be interested in it, it's not something that can be forced, if i don't want you to use my body for your research work, of course you need my consent, so i have to agree, then i will align with whatever method or procedure that you give me. so I think the first thing will be the agreement first then, before any other thing.

**Interviewer: do we have anybody that want to add to that? How does biobanking operate, the operation, the procedure and guidelines that has to do with biobanking Ok. how important is biobanking to medical breakthrough? do you think it's important at all?**

R3: it’s very very important, because like that of the blood I mentioned ehhh if there's no enough blood they cannot dialyse and the importance of having blood bank is to go there for those people that doesn’t have enough blood to carry out what they want to do, they will go there and get blood so that they would do the dialysing successfully. so ehhh biobanking is very very  very good.

**Interviewer: Thank you. addition. What is the benefit of biobanking to medical breakthrough? Do you think there is any benefit?**

R1: the benefit that is in banking of body parts, there is benefit there and the benefit is that if you keep it, if you keep blood and you now need……, there are just many benefit there

R2: yes biobanking is beneficial for medical breakthrough because it's going to remove a lot of ehhh cloud from what we currently know, so if we have more research done then the future of human life will be preserved, people might be able to live longer, you understand so it's very important for medical breakthrough.

**Interviewer: Thank you. you want to add anything more?**

R5: ya biobanking is very essential to the medical world, in the sense that it.. provide the researcher the opportunity to have something to work on in terms of investigations and it's also..opportunity to have something to work on when you doing investigation. if you are investigating a particular issue, they can actually take a sample from the biobank and use for the analysis without necessarily venturing into a life human being . another thing I think is very important for keeping..eh having a biobank is that it can actually be used for people that are...that needed the particular part of the body in the future. in the case of emergency , you don't have to be looking for a donor you just approach a biobank if they have this particular organ and they will give to you and save the life..thats all.

**Interviewer: thank you very much. What are now the belief, thought, opinion relating to biobanking.  what are the opinion, belief of people, their thoughts relating to biobanking.**

R2: ok, i think ehh in this part of the world they first thing that might come to people's head if you try to tell them preserving their..part of their body might be...maybe you want to use it for rituals, stuffs like that. ya ya..you have to think about that one..maybe you want to use your body..part of your body for making money or whatever. but i think with ehhh ehh enlightenment, more education and all we will understand that it is important for us to do that. so that's just of the things that can come to my head right now, so probably for some juju, voodoo whatever.(laughs)

**Interviewer:. What are now the belief, thought, opinion relating to biobanking. What do you think will be thought of people if they hears something like this**

R1: what I think is the belief of people is that if you keep body parts, somebody will not have stroke, one will have kidney diseases, one will not have high blood pressure, that is what is think

**Interviewer: what do you think are belief, thought, opinion relating to biobanking?**

R5: ehhhh there are some religious beliefs that dont believe in keeping their body parts, even when the person dies they don't believe in embalming the person in the mortuary, they feel its against their religious belief..it's sacrilege for you to keep a body part, that as the person has come to this world the person should be buried and go back to God in that way, so there are some people that believe that way, so those people would not want to either be a donor..or even accept to use what has been donated. so religious belief is a very major factor. Thank you.

**Interviewer:Thank you very much. you want to say something?**

R3: That's what i want to say because there are some even if they are dying like this they will not allow you to to get blood for them for use. so they will like to remain like that either to die in the position of that sickness or to live, they will not accept any blood or any thing related to that. so that's some..there are some people they really believe in religion, so their religion is what they are being barrier for them not to be using such.

**Interviewer:thank you very much. what is your awareness or understanding or rather perception of brain banking? brain banking.**

 R1: whose brain?

**Interviewer: have you heard about it before? What is your perception? What is your thoughts? What is your opinion?**

R5: ehhhh, yes i've heard about it before. ehhhh I think one of the reasons why they do brain banking is that they want to preserve the intellectual that some people carried. you know i've heard it before that some people said they have to preserve the brain of Chief Obafemi Awolowo, so that it may be that if those kind of people can be preserved that can be used even for other people and they will have the same kind of intellectual, the type of exposure that those people have, so that is what i think that why they do brain banking so they will make use of the same brain and achieve the same level of intelligence that the brain carries even in other people.

Interviewer: Thank you. well awareness and understanding about brain banking

R2: well i think i've just heard it as a joke, maybe when i was in school like when you talk about Isaac Newton saying saying his brain was preserved and all such but really i dont think its happening, i don't know..i don't know.. i'm not sure it's possible to preserve someone's brain, i don't know.

**Interviewer: Have you heard about it before? What is your understanding by brain banking**

**Interviewer: No 4 are you are aware?**

R1: I have not heard about it before but the way I understand brain banking maybe, someone should be careful to avoid hitting the head on the ground and should be resting. I have not heard about it

**Interviewer: we are talking about awareness of brain banking? Have you heard it before, are you aware that it's something that is in existence? Or what?**

R4: ah I’ve not heard but i don't know whether i will be correct, let me just say..i've not heard of it, but i just want to say something if am correct or not. maybe it's just like where things are stored, memory..abi what will i say..your senses, everything memories stored there...

**Interviewer:Have you heard about banking that brain?**

R4: Banking the brain? Eh

**Interviewer: where you bank your money, you keep your money..**

R4: ya you keep the brain, keep the brain..

**Interviewer: where?**

R4: Ahhh, that's the thing (laughs)

**Interviewer: Thank you very much. Are you aware of any policy or law guiding biobanking? do you know maybe there is a law guiding biobanking?**

R5: yes i think there are laws ehhh guiding biobanking because ehhh you have people that are called.(inaudible). they indicated that in case of their demise, that they want them to preserve their organs and those have to be documented and even in..in ehhh non...in advanced clan they use to carry some kind of tag, maybe..maybe on their ..on their NHIS details or on their details that this person is a donor, so in case the person died in a place that is far from...so they will see that this person has indicated that he wants to be a donor. so for somebody to have indicated that he wants to be donor there must be some kind of laws that guide that kind of ehhh ehhh thought.

**Interviewer: any addition? your awareness of any policy or law guiding biobanking**

R2: just like the case of ehhh banking of blood so ehhh there are regulations guiding that, so there will be regulation of course for donor... ehhh of body parts as well.

**Interviewer:Thank you..next question can you explain what you understand by precision medicine, individualized medicine, precision medicine**

R2: I think precision medicine is simply about matchmaking..making sure that you have the right match for the ehhh patient that you what to use for. for example in case of donation of blood, you should sure this person matches this other person, so if I want to donate my kidney, we have to run some kind of test to be sure that whoever im giving my kidney to has the same working condition of..whatever kind of medical terms it is called.

**Interviewer: you understanding of what is called precision medicine**

R5: ehhh aside the fact that you have to ensure that there’s a proper match for what you are using, there are also supposed to be a proper expertise to administer the match as in people that have specific knowledge in that area, to administer such med..medical proceedings.

**Interviewer:precision medicine, what comes to your mind, immediately i mention precision medicine and i still explain by saying individualized medicine  what comes to your mind?**

R3: our director use to give precision to the ..to the patient so that's why i believe, for a doctor that will give a precision to a patient must be a good doctor that will give something that match what is doing the patient ...direct to the patient.

**Interviewer:Thank you. when im talking about precision, i'm not talking about prescription. im talking about P. R. E. C. I. S. I. O. N. because individualized medicine, what comes to mind . with the little that you've said do you think theres any benefit of this medicine and is it important in Africa?**

R2: ya its very very important to get whatever we are doing right, you understand? because we have seen people been given the wrong treatment or medicine for whatever it's wrong with them. its very important that our medical practitioner...our expert..they have the expertise, so that at every time that they are delivering their services its always right.. they do the right thing so that whoever has ehh. let's say you can be giving the drugs for someone that has kidney issues to someone that has diabetes, so it’s very important that there's precision in medicine.

**Interviewer: Thank you. precision medicine, those it have any benefit, is it important in africa?**

R5: ehhh precision medicine per se its more of.. is more of the medicine that is...that is applicable in the advance world, in Africa we are still struggling with the basic medical treatment, not..talk less of when you have to customise a particular type of treatment for a particular patient but i think in africa now people that..that desire precision medicine usually travel abroad but ehh maybe ...maybe..in the nearest future rather than get to the situation, where we can we can we can have tailored made medical ehhh treatment for such people.

**Interviewer:The this precision medicine that we are talking about, can it be applied stroke disease, can it be applied to stroke precision medicine?**

R2: if we have to go back from where we started about bio and everything, it is ..precision medicine can be applied in stroke disease because if you know what is wrong then we just go straight to the point, there is no need for us to be doing unnecessary observations here and there, so , so suffer, I know this person has stroke then i will just intervene by giving him the kind of medical assistance that he needs so it's very important that we have precision medicine in treatment of stroke.

**Interviewer:Precision medicine, can it be applied to stroke disease?**

R3: yes we can use it, because there are some..those one that have stroke that they cannot be walking all around especially coming to the hospital, it can be something of a problem to them, so the precision medicine can be good for those people.

**Interviewer:Thank you. addition precision medicine can it be applied to stroke disease**

R5: ehhh ehhh for the much i know that ehhh, i don't know if stroke is curable and i know that its manageable, you know ehhh if it is curable, I don't know if the medicine has ..if the.. if the research had gotten to somewhere they can cure...they can cure it, then applying precision medicine will be very important because I think ehhh customising the particular need for the particular person to cure his own case will be very good. in fact i think it will be efficient, on the premise that it is curable. I don't have that information.

**Interviewer:Thank you. what are the beliefs, thoughts, opinion relating to precision medicine, what are people's belief, their thoughts relating to precision medicine? what are their beliefs.**

R4: ehhh, like we said, or you said, I don't know that it's not something in this country, that its something outside, so some people believe ahhh going out of the country to get it might be difficult, maybe the financing, how they will get there or something like that. that it will be difficult for them, it's better 'ahhh, me i can't go ', because of what is attached to it, maybe the money for them to go there, so that's some people belief. they cannot afford it.

**Interviewer:Thank you. any other contribution?**

R2: precision medicine, actually in this part of the world.. I don't know.. maybe it's because of the ehhh doctors or nurses or whatever, at times you find out that once they give you some drugs to cure something, what you expect might not be happening so over time we...sometimes just believe we are just going to all these hospitals just for the sake of going.. you understand.. maybe they need to do more in that regard so if I’m having headache be precise enough to address whatever is wrong with me.

**Interviewer:Thank you. Are you aware of any policy or law guiding precision medicine? do we have law or policy guiding precision medicine? Are you aware? let's talk even if you are not aware..i want to hear yes or no.**

R5: im not aware of any

R2: im not aware because like i said before we just leave it to God, if you go to the hospital and you are killed there, you just say its Gods will, thats why, i'm not sure there's any law there.

**Interviewer: Thank you. my next question, what do you understand by brain donation for research purposes? What is your understanding on donating brain for research purpose? you can answer me in either yoruba or english language based on the one you prefer. what do you understand by brain donation for research purposes?**

R2: I’m sorry, is it possible for us to ask you a question?

**Interviewer: you can ask me after.. what do you understand by brain donation for research purposes?**

R5: ehhh just like we said earlier, that ehhh that the biobank preserve important organs that can be useful for thereafter. so donating brain for research purpose is ..is when you know people that.. when people die and they ...they agree that they should keep their organs for use after..you know most of..most of the issues that people have especially when it comes to strokes that has to do with brain, you know it would be easier for them to have a specimen to work on, so when they see a case you know they want to stimulate that ..the same scenario that they are seeing in a patient.. you know they can not be stimulating..they may not be stimulating the patient brain but they can get a stored brain you know and do it in form of.. or something that has happened to the patient, probably the patient died and they want to know what really happened, why did the patient die, so they can take a living brain that is stored to you know, to just work around and see you know.. to know what happened.

**Interviewer: Thank you.. what do you understand by brain donation for research purposes?**

R6: to my own understanding, I look at it that ehmmm while person is alive or dead but if you have seek the consent of the family to to use such ..such a person for ehh research and you are granted, i think ehhh its part of it and they can be living body, ehhh they can be a dead body to get more research on what you.. what ehh has affected the community or or or a country at large

**Interviewer: Thank you. Our understanding about brain donation for research purpose.**

R2: I think donating my brain for research to my understanding should start from when I’m alive as you can actually study maybe the way I do things using my brain from now my way of life, then of course you might continue with it after I’m dead if consent is granted. so that means I’ve actually donated my brain for you to use.

**Interviewer: Thank you. I want to ask about the complexity of this procedure of donating ones brain and the benefit of brain donation. what is the complexity of the procedure of donating one's brain and the benefit?**

R2: I don't know the procedure, so I don't know whether it is complex or not. that's it

**Interviewer: what about the benefit, do you perceive any benefit in donating one brains?**

R2: yes, I think there will be a lot of benefits, maybe for future students, they might not need to stay too long reading to be able to get some things. maybe people will be able to manage their health better if they understand how to use their brain.

**Interviewer: what are the benefits of brain donation?**

R6: as the person has said that i personally i don't know the procedure, but I’ve know that as ehh a matter of fact if such research is carried out , it will reduce further occurrence of other things should have happened for like the issue of ebola, when it came and the the researchers were on top of the situation and at the same time of HIV and AIDs, as a result of that those things has reduced more now.

**Interviewer: Thank you. what are now the misconception and personal willingness to donate? do you understand my question?**

R3: why I’m doing that is there are many people that doesn’t understand what we are trying to discuss amidst us. they don't understand it. so, they would agree to donate willing.

R2: I think, we don't have any knowledge about this or maybe it’s not well circulated, at least i have been through school for some years and I’ve never heard about people donating their brains. so maybe if there are more campaigns, maybe if it’s taken to schools, to community meeting and all, people have more understanding and they may be able to probably subscribe to it if they want to.

**Interviewer: do you think that any cultural or social or religious belief has regards donating brain for research purposes?**

R5: ehhh yes, in this part of our world, you know people see it as a taboo to give your part out. you know even..even if the person, even the donor..after the donor die you see that family will find it difficult to accept the brain to be used or to be stored in a in a.. bank for research purposes. it's just a cultural perception that you know, is ehh is a taboo sort of to give out your..part of your body.

**Interviewer: Thank you. any other addition? cultural, social or religious belief as regards donating brain for research purpose.**

R2: I think ehmm, like one of my contribution earlier, the issue of trust..just lets look at this opay thing thats is coming now..they gave you helmet for safety, people are not putting it on because they believe that propably they put the helmet on, they might find themselves somewhere or wherever, so people will see it as ...they are still thinking maybe they want to use their body parts for rituals or whatever. then another thing is i think maybe if we start it , like i said earlier, if there are campaign, maybe there might be change. then culturally also even just imagine someone dies in an accident or in a fire, people still try to pack the remains and say they want to bury. people just find...they just need something to bury, so it might be hard for them to release part of the body.

**Interviewer: Thank you. what are now the things..he has said some..that can promote brain donation?**

R5: I think education, awareness, and ehhh and education and awareness basically. because when people get to know that you.. when you donate their brain for research work is not that you will bring ehhh curse on even the family, so they have to have that understanding that it's just for research work to improve humanity. so they would be able to oblige.

**Interviewer: what are the other that can make people to willingly donate their brain**

R2: in addition to what he has said, in Nigeria right now, if you want to use anything from anybody, incentive is part of it. they might want to know..are they been paid for this, is there any benefit that accrues to them..am I going to gain something from, is there any naira and kobo  at the end of the day. so it's just not going to be...nobody wants to do anything , like they say nothing is free, so it might be a hindrance. or I don't know the modalities in advanced climes but i think the financial issue, what you gain from it might want to be administered too.

**Interviewer: Any other contribution as regard factors to promote brain donation?**

R3: just like no 4 has said that we need to educate our people, so if educating them and you give them free mind to donate.

**Interviewer: What do you understand by blood sample donation for genetic research? blood sample donation for genetic research.**

R5: ehhh a lot of information are stored in the gene and if you want to access those information most time you need the blood because the blood virtually carries a life of the person, so you can get so much information about the person from the blood. so many ehhh investigations are done through the blood, if you want to ..you know you talk about ehhh even the...as long as even the ehhh biological and (inaudible), those information are stored in the blood because it's the blood that carry information to the brain, so if you really need any information of the person, the best way is to get the blood sample, which carries the ehmmm genomic material.

**Interviewer: Thank you.**

R2: genetic research through the blood is important, let me just cite a simple example of this sickle cell issue when people want to get married, so it's ..with that kind of information they would know whether they should continue. so with our..if..with more genetic research of blood we would be able to help in future to probably ..like some people that have some kind of deficiencies or so may be able to manage their lives better, the issue of diabetes and all.

**Interviewer: Thank you, donating blood for genetic research, genetic research, what does it mean? genetic research..donating blood for genetic research.**

R6: i..i.. look at it that it's something that is very important in ehhh the way that make ehhh the life of ehh this dispensation easy, like the issue of genotype of the wife of the husband, if both of them tested they would be able to know their know their genotype but most of the time hmmm as we are too religious even knowing self, some of us. Some go ahead and be saying God is in control and ..and the end of the day things goes wrong.

**Interviewer: Thank you. do you now think there are benefits of donating blood for genetic research? we've made mention of few of it but i think we can get more benefits. do you think that there more benefits of donating blood for genetic research?**

R3: there's a lot of benefits because like what No 6 has said, that there are some that...like now i heard about that AS with AS can not marry each other that there will be SS gene among them. so if there's no research on that there's not how i can know, maybe im AS and my ehhh partner is also AS, and apart from that those ones that work with Diabetes, there's no how you can know, as no5 has said also that ehhh many things that is happening to to man can be...the research can be taken out from God. so there’s benefit a lot in donating our blood for research on gene.

**Interviewer: Thank you.**

R2: i think there's a lot of benefit in it, i might just cite a few personal example..like ehmmm before my own generation with my grandparents, some of my grand uncles and all, i know they have a lot of history of people having asthma, so but coming to the grand children, we just have only one case, you understand, so you probably ..so i've been thing about it to like maybe there should be a reason why some of us while some of them have it..something like that. maybe with that we will be able to know what to do, what not to do, why is it that some of them have while there some that does not have it. of course we that God that we don't have it but you know probably using more of science we will be able to understand why. also there was a case of one of my aunt, they don't know that she had diabetes until when she wanted to give bath. so if we have such research work done, she probably.. she would probably have been able to know she as this kind of issue or not. so i think it's going to bring a lot of benefits for us..we not been...there would not be crisis unnecessarily, we would not just find ourselves in problem, we would know that ok we knew this before and ehhh and we will be able to take care of this and all that

**Interviewer: Thank you. Other benefits**

R6: yes. the other one i wanted to say is that these ehhh these would safe us from ehhhh health superstitious belief that is the mother of so so, that  killed the child, ehmmm ehmmm it was witches and wizards that ehhh kill my son or daughter. through this ..it will be easy for us to know that human life is not all about witches and wizards but life is more of research ..of making sure that we live a better life.

**Interviewer: Thank you. The benefit that is associated with blood sample donation for genetics research. What are the other benefits that is there?**

R1. the benefit that is there is that if they examine the blood, one will know maybe it is AS or SS, so if they do blood test, the person that has tuberculosis will be identify……not too clear…..

**Interviewer: what do you think are the cultural, social and religious belief on donating blood for genetic research?**

R5: when you get to blood in the part of the world it's a very...people are very skeptical when it comes to issues of the blood because they think blood is one of the things that people use to do them, so they don't really want to..anything that have to do with blood, they don't want to play with it and again people..some people feel that ehhh by taking somebody's blood into their body that ehhh, they are taking part of that person. I know of people that they don't take blood, not because of religion, but some people because of religion, some people just feel that taking blood is means that you are taking a portion of somebody's life into your body.

**Interviewer: Thank you. Belief..**

R4: ehhhh like i know of ehhh, permit me to mention name...i know of ehhh one of these religious body like the Jehovah Witness people, they belief they don't donate blood and it will result to...i have seen...as in i have witness somebody that is from that particular..ehhh maybe ehhh religious body, the woman wanted to give birth so she was short of blood so they said she should..somebody should donate blood for her, they tested the husband, tested some other people, the thing could not match, so they now...they were looking for donor so they got one, so she said No but at the end of the day, the woman died, because that is their believe. some people they are ahhh.. that's their own belief, they don't receive maybe another person..maybe collect another person blood. she can only collect from maybe your husband or something, that resulted to her death. and some people are ..what  are this people taking my blood for...noi ahh, me i'm scared maybe they want to use it for something...because they will feel i will not be there when they are using this thing so why will they be taking it, i don't know the end of it. so that's some people's...

**Interviewer: Thank you.**

R2: I think when it comes to religion, social or whatever and everything, the most..the only thing that can prevail or penetrate every circle is education. if people are educated about it, they will be able to be open more, then also when we talk about education, it shouldn't be only four walls, you understand,that can campaigns, even movies that we watch you can find a way to induce it into it. look at this ehhhh white ehhh people in (inaudible) they have movies like..even though its not human whatever, they have movies like vampire movies that they fill a lot with blood and even from the blood they are able to get their history once they are drinking their, you know they show it to us in the movie. these are also subtle ways of making people to open up concerning research about their blood. so we can penetrate religion, we can penetrate traditional, cultural, whatever if we put more into our education.

**Interviewer: Thank you. lets go to the next question. share with us your opinion and thoughts about blood sample donation for stroke genetic research. when we are talking about donating research...donating for research purposes, now when its comes to donating blood for stroke research purpose, you are sitting here, if we should just tell you that will you be willing to donate your blood, let me explain, for genetic research purposes? this is what we are trying to say, when you give out your blood it may be a medium tracing..maybe there's likelihood of having stroke in the future. if we now say we want to start that study, will you be willing to participate? What I am saying is that we want to know your opinion, we want you to share your opinion and thought about blood sample donation for stroke genetic research, will you be willing to participate in such study or research? will you be willing to participate in giving out your blood, donate it for stroke genetic research?**

R6: I want to say that ehhh due to cultural beliefs and we are too religious in this part of the world that when it comes to test of knowing what is the cause of our...of one challenge or problem, we feel that God can solve it, why will I be giving blood for ...sample of it all the time, like ehhh for like the problem of HIV and AIDS people refuses to go for test which is free..that i don't want to know..and ehh i don't want to know what is going to kill me...but if one knows it it will save one's life than dying before time or sometimes we feel like going in for test God is able..let me go and pray ..and at the end of the day, the person ends up dying and ehhh if such person dies the sample of the blood maybe there are some research that died too, that cannot give the accurate result of what they should have get when he or she is alive.

**Interviewer: would you like to donate?**

R6: I like to donate but ehh to sincere not all the time, and at the same time, i should know what exactly you want me to donate it for. because there are some things that i don't think i have..my family ...ehhhh you look at it that i didn't have so I don't want to look for something that is not lost.

**Interviewer: Thank you.**

R4: as for me personally i've never done it and ..

**Interviewer: would you be willing to donate your blood for such research?**

R4: it depends, is it for...

**Interviewer: for genetic..for stroke genetic research**

R4: yes, I will, but i've never..i've never donated blood before. but before i will donate...well i will, i will.

R5: I will surely do, because what you are looking at is very important. we know hypertension leads to stroke and hypertension can be genetical, it can be you know, it can be hereditary, so if ehhh by donating will help the research work to know ehhh the history and as a result be able to you know , make some good discoveries, so why not, i will willing donate at all the time.

**Interviewer: Thank you**

R3: I will donate

**Interviewer: ok**

R2: I will donate because these days now even guys of my age  they just come back and say they have high bp, really im getting a bit worried because normally I use to think its because people are overwhelmed with... there was a time I was always checking my bp too because those period i was not really sleeping so i was getting scared like i hope i'm not going..having any problem you understand, i think we need to, because..just move around, when i came to the hospital today, I was surprised to see a lot of people so I think we need to start doing more, we can't keep on flying outside the country for all these things, we need to start helping ourselves. so i'm willing to donate.

**Interviewer: would you willing to donate your blood for stroke genetic research**

R1: I can give my blood if my body is capable of it

**Interviewer: what are the barriers that could hinder your donation of blood sample for stroke genetic research?**

R4: the hindrance to my ehh.. maybe because before you donate there will surely be tested..they will test if your blood matches and one of the hindrances is if they ..after testing you your blood does not match it might not donate, that is one. and probably the person wants to donate but if your blood does not match, you might not donate. not only that, if they test it and find any other thing apart from not matching because is the people that..those that are involved in doing it, they know what they are looking for, so by the time they test it and they see..they notice any other thing which is not ok by them. those are part of the things that will stop the person from donating.

Interviewer: what other thing can hinder us? can family members, cultural or religious reason, peer values, parental influence?

R5: yes, you know ehhh in everything people ehhhh people you are close to tend to influence you in what you do and they will influence based on their level and education about the subject matter. you know if peradventure you seek the counsel of somebody that "oh i want to donate blood for particular reason", if the person is not so informed or knowledgeable about such thing he can discourage you, so that's why ..or if you have a religious belief that does not support donating blood you know it can discourage you. so it depends on the level of education, exposure, religious belief of people that close to you.

**Interviewer: any further barriers apart from the one that you have just mentioned? any other barriers?**

R3: if..if im not ok, maybe i have malaria, i cannot donate.

R2: I think one of the things will be..probably if there's no policy like we were talking about family and norms, someone that is not up to age might not understand what you are trying to do, maybe there should be a policy first of all to educate the person that wants to donate then the person should be of age, so he will understand whats his trying to do.so thats what i can say.

R6: ehhh.. first of all i want to say that if the people that are coming for the research are genuine, I will not donate because of there are so many things going on thereby one may say 'I am a researcher' and where taking your own blood to, you don't even know, so sure people should be people that know one or two of them. like this one you are doing now, look at the researcher and the people that are part of the team, I know that these are the people that i can go on with them.

**Interviewer: any other person? What are those things that can hinders us from donating our blood sample**

Respondent- no response

**Interviewer- what are the benefits of giving blood sample for stroke genetic research that could promote your willingness to donate?**

R5: personally if i would be opportune to see the outcome of the research work and probably (unclear) to my person, to be able to see the result carried upon my own sample, so that I will be able to be aware that 'oh by donating..' and i will be able to know that 'oh in my lineage ehh there's a tendency..there’s no tendency..for me to you know, for us to have this.. so i will be willing 'oh since i'm going to know something about myself, my family, and I will be able to guide against it', so that will be a motivation for me.

**Interviewer: what are other benefits?**

R2: I think personally i will love to get involved like i said before, there are a lot of issues happening around young people right now, so i will like to know especially the result, the outcome of my own then how it might also relate with other people, so as to prevent maybe stroke in the future. so i will be interested to know the outcome, so of course that should be a motivation for me to participate.

**Interviewer: what do you think are the benefits that you can gain from it, that can make you to willingly donate your blood for this stroke genetic research? any other person?**

Respondent- silence

**Interviewer- Now what do you think can be done, you've mentioned some of it but i want you to add more, to make more people give blood sample for research?**

R3: we educate them as per what we want to do and I hope been educating them, they will copy and willingly to donate.

R5: there is ehhh, there's a policy in FMC here, which ive gone through before, in fact it was at when I wanted to have my second kid, that as a father you are forced to donate blood for your wife during delivery, so it's a subtle way of ensuring that people donate and imbibe the attitude of donating blood aside the enlightenment and education and other things, the government should find a way to subtly force people to donate..to donate blood.

**Interviewer: Thank you. what can be done to make you and others agree to donate their blood for research**

R2: I think there should...there should be a policy around this. Look at for example if you are going to get a job in this country you have to..as a degree holder you have to go through the NYSC scheme, so there should be a policy that can kind of make quite a number of people in the population to be able to partake in it.  so a policy should come up maybe when you are trying to do your national ID card, this is going to be done or whatever, then also immediate benefit should also be a way, maybe incentive..i don't know what they do for people that come to donate blood as in the voluntary donors, maybe incentives or whatever can make people to to to come for it. or maybe a national, of course you can't give people national honor for this but something that can you know, that can make them to walk proudly in the society and say that they gone and be able to this.

**Interviewer: what do you think can be done to make you and others agree to willingly donate their blood for research. What can we do to encourage or make people willingly decide to donate their blood for research?**

R1: what I think we can do to make people donate their blood is……., the way the society is now, if money is involve, if money will come from it, even the person that don’t have much blood will come out to say he wants to donate, if it involves money but if it’s free, those who even can donate will not release themselves

**Interviewer: No 6 you want to say something?**

R6: yes i want to say that ehhh most of the time, the enlightenment is not enough because on..over the radio, televisions we don't really give this ehhh pronounced announcement all the time and it should have been a work of national orientation agency to ehhh give it out that and it should have been a way that everywhere you go from time to time, people listen to it over the radio, in the paper and through that the testimony of the people that have benefited from sure programme would be able to give people ehmmm more concern of coming to donate and those that are donating should be taken care or because something have taken out of you at that point in time.

R3: there was a programme we heard in this FMC then, that made us to come out to donate blood. so at the processing, they've prepared tea..Milo tea with milk, before we..before we sleep on bed, we will take bread and tea or..and if you like meat pie they will give you, then after they will now give you t-shirt and ehh Malta with milk again. so if someone that wants to donate see all those things they will be willing to donate or you just want to take blood from somebody just with main mouth? no milk, no tea, nothing nothing? that person will not..will not donate but if there is any donation that we can make so that people will come to donate, I thought that day we are many that did that programme and we donated, everybody donated blood supply. so at all there should be a motivation for..if there should be the program for it.

**Interviewer: Thank you very much. Now tell us what you know about informed consent? before we start this conversation, we passed out a sheet in which we tell you that (inaudible), now i want to know..that is what we call informed consent, i want to know what you understand by this consent..informed consent. what do you understand by it?**

R5: ehhh, informed consent means that everything that you are doing is out of your own free will, you know nobody is forcing you to do it, every function you are given, you are doing it freely and you are not doing it under duress. it's just like you sign up before doing surgery, that you agree to this procedure, that you agree with this research , you agree to partake in this discussion, you agree to partake in this seminar, so those things thats what what we call informed consent. so when it comes to donating blood, you know, or organs for research, they are an agreement that you sign, you know, that will show that you partook of it out of your own volition, it's not under duress.

**Interviewer:Informed consent**

R2: what i understand by informed consent is ehhh before I join you in doing whatever you bring to me, you share the information about what we want to do and you make sure i understand it, you get my feedback and i understand it, then I will be able to agree if i can continue with you or not. if I want to continue with you that means i’m duly informed about what's going to happen, what's likely to happen and I agree to do it. like in the case of Nigeria army, you consent form that you are willing to go through this rigross, if you die in the process, you die, so whatever that is in it the person that is bringing it to me will be open, so I understand what we are doing, then I will be able to agree if i want to continue or not.

**Interviewer: we are talking about informed consent, that I agreed to participate in something, what do you by informed consent, any other person? informed consent, these are the things we sign**

**R**3: before I came here, you didnt force me, its my friend that.. Osi ..she's the one that came to me and tell me about this and i believe that as No 5 is talking, No2 is talking, No 6,No 4, No 1, im gaining some things that i dont know before as they were talking. so i think, im agree willingly

**Interviewer: Thank you. what do you know about the consent process for genetic research? you know all what we have been ruminating about is, is about genetic research, then when we are now talking about consent process for genetic research, what do you know about it?**

R2: I don't know anything about the consent process for genetic research.

R5: I don't have any idea about a particular consent process for genetic research

**Interviewer: Thank you. we have different types of informed consent. i will explain it to you, base on my explanation, then i will ask you which one do you prefer. There are different types of informed consent dynamic informed consent, in the simple term is a consent which allow you to participate, you participant to have interaction..interactive relationship with the custodians of biobank, you've agreed to participate in the research, to donate part of your body and in this you have a grace of interacting with the person that is the custodian of the part that you donate and with the research community, that is what you call dynamic, where you put your part, you have access to interact with people that is keeping it, that will biobank it and with the community where the research is taking place. another one is what you call tiered, this is a consent in which participants are given option which allow them to select any agreement on how they want to participate in the research, a consent in which you are given different options and you now select..in only this part, is only this that i will like to participate as the aspect of the research, do we understand?. (3) we have restricted informed consent, is a consent where..i mean you as a donor, person that donated any part of the body or any part of the organ for research purpose, you restrict the use of that sample collected from you to that research they are doing alone, it must not and can not be extended to other research, that they will now take the part when they want to conduct another type of research but for that research that they doing, that is restricted. the fourth one is broad, this is a consent given by you participant to allow the collection, storage of any..any material or blood that use donate for use and for transfer to any other places. you know it's different restricted - for that research alone, but broad for any other research in any other part of the world that they want to use it. and the last one is generic, which focuses to see the participant agreement or blessing on the broad or overall focus or scope of the research. dynamic is a consent that will have you to have access to the part that you donate, with where it is been banked and the community where the research is been taking place. tiered is consent in which you will be given different option, then you agree on the aspect you want to participate in. restricted is for the sample donated to be used for that research alone and not to be extended to another one. then broad is for it to used in any other research and in another part of the world.**

participant: (inaudible)

**Interviewer: type of informed consent that you now prefer..which one? all of us need to choose one..**

5: ehhhh, I suppose to choose just one?

**Interviewer: yes..the most preferred**

5: ok, the most preferred. ehhhh, i prefer the broad,

**Interviewer: why?**

R5: yes, because you know, i ..i want the medical world to continue to improve, as long as it will continue to lead to the advancement in the medical world, im ok with it. the only...the only part I would have loved to like the first one is it will give me opportunity to to..i'm not really interested in where they keep the organ but to know..to interact with the community where the research is being carried out so that i can have a form of feedback on ok 'this research I was part of, this is the outcome that is been generated' that's the..why I would have preferred the first one but most preferred i prefer the broad version.

**Interviewer:Thank you**

R6: I prefer the second one which is the tiered, which say that ehmmm there are some places it should be restricted and ..because of course if we are interacting i am to know what is going on, if you are using it for sample and i will know and whatever that is going will..will be able..if you have said that you want to use my head for sample and it can't be possible for now, and i can't die now and except...so i preferred that tiered one which some area i will be able to or where i can not.

**Interviewer: Thank you**

R2: well i wish there’s an hybrid between dynamic and broad, since we don't have, when I'm alive I will prefer the dynamic, so maybe when I'm going to die, I will let you continue to use it for whatever. so dynamic one is ok, i will be interested to see what is happening, to know and all

R3: i prefer broad

**Interviewer: reason for your choice?**

R3: reason for my choice? yes i broad because my blood also can be used for another person.

R1: Broad

**Interviewer: why?**

R1: i prefer broad

R4: I prefer the broad.

**Interviewer: Thank you. do you think that there are people to be involved before you participate?**

All: yes

**Interviewer: There are people that you need to involve? maybe your family member, maybe your wife, your relatives, your friend..that you want to tell before you want to partake in this type of study.**

R4: as for me, ahhh yes, I will want to discuss it with my husband and if ..let me hear his own reaction, I will even like to..if ..i will still tell 'tor i want to tell my parents, should i?' if he says yes, I will want to tell them. that's for my own side

**Interviewer: Thank you**

R3: I can only tell my husband that I want to donate blood and i know he cannot restrict me from donating my blood.

R2: its personal, i'm not informing anybody about it. and of course im not married, so im not obliged to (laughs).

R5: ya. its personal, i'm not telling anybody if..if I have to tell my wife its just ..its just in passing its not as if im seeking her approval to do it. you know its a research work, it's not injurious to me, it's not ehhh there's no hazard, there's no side effect, so it's personal, i wouldn't tell anybody.

**Interviewer: Thank you. what is your opinion on blood or storage of blood sample and blood factions for genetic research? what is your opinion on blood storage, on storage of blood sample and blood factions for genetic research, what is your opinion?**

R2: like all what we have been saying since, it is important for our future, then also i think ehhh genetic research is also creating jobs for people, so it's one of the ways to get people engaged, you understand. of course people will be there, they are working helping us to improve our future life. so my opinion, i agree with whatever it is.

**Interviewer: your opinion on storage of blood sample and blood fractions for genetic research what is your opinion?**

R5: well I think it a good thing ehhh im possibly dispose to it , because the blood sample been kept is going to be used for research purposes that will benefit humanity so im positively disposed to it.

**Interviewer: Any other person? your opinion on storage of blood sample and blood fractions for genetic research**

R6: let us proceed as we have said i have concurred to what they have said.

Interviewer: Thank you. Tell us what you know about sharing of data, blood, or blood fractions, brain images as well as brain tissue samples, what is your opinion on sharing of data, of blood samples, brain images or brain tissues with other researchers locally and internationally?

R5: ok information sharing is very important, it helps to avoid unnecessary duplication of research work. if research has been conducted on a particular ehh field of study and you have an hypothesis out and  you can say this is conclusion, there's no point in conducting the same research somewhere else if ehhh if culture and environment is not a ..is not a ..is not an important factor, you know, you just share the...share what you have with other people and they can make use of you result, so that will make research work to be faster and ehhh easier.

R2: data is life right now, it's very important I have ..probably someone has information and i'm able to get it as fast as possible, and that will also help us to improve on what somebody else has done, you understand, if im working on something and someone in australia has worked on it before, then i can just pick it up from there and continue the research, so it's very important that we share our data. there should be a linkage between research institute and all, very important.

**Interviewer: your opinion on sharing of data, blood sample, brain images or brain tissues with other researchers locally and international. what about your opinion...No 6, you want to say something?**

R6: No ma

**Interviewer: what is your opinion on commercial or non-commercial use of stored data, blood and blood fractions, brain images and brain tissues? your opinion on commercial or non-commercial use of the stored blood, or blood samples, brain images? the use of that stored blood, what is your opinion on it? either they use it commercially, or non-commercially..**

R2: if you are going to sell my blood, i need to know, because if i'm donating freely, i wouldn't agree for you to sell my blood. but if you are going to share, you know, im scientist too when i was doing some research, for me to get some things i have to pay, because some people have done some work, so if you are going to pay for the work that someone has done about me, i think it's worth it, then something should be coming to me as well, i think. if you are going to be making money from me, then something should be coming to me but its my blood, if im giving you for free, then you should be giving it out for free, but since you have done some things in your lab, worked on it, you've invested some money, so i believe you should be able to recoup some money from it, of course you might not really pay me like that, but getting me involved in research will be good.

**Interviewer: any other person?**

R5: i..i totally disagree with ehhh commercialing samples for research work, you know, if you have a sample that you have taken for possess of the research, it should be used strictly for that purpose. that does not mean that, just like the other guy said, if talking about the output of the research work, you know , you can make money from it, because you've used your expertise, you have your resources, you have to use you staff to get an output, you know, but when it comes to using the samples that you took from people, it should not be commercialised, in fact, if you want to give..you can even donate to other people that need it for research but it should not be commercialised since you get free.

**Interviewer: any other person? thank you very much. share with us your thoughts about the return of individual research results and incidental findings. what are the ways that you think one can receive the result of genetic research? we are asking you, what are the ways that you think one can receive his or her result of genetic research? if somebody participated in a genetic, what are the ways in which you think that person can receive his or her results?**

R2: if the results of the research would cause an upset to the person, you can send an sms, emailing it, no problem, but if it's going to cause an upset, if it's something that you might need to work discreetly about, you understand, you might need to call the person, inform..let's assume you find out in the process of trying to look for something in my blood, and you now discover that ok, this guy has HIV, then you know there needs to be another procedure about that, so you can not break the news to the person like that, maybe the person has tuberculosis or something, there should be a way you have to help the person against what might happen from the result. so if the result might cause some form of depression or whatever then the proper medical procedure should be followed instead of, you know, you can send me malaria test result now through email, i might not necessarily come to collect it, so that one is still mild but something that you know is really detrimental to my health, we might need you to call me, maybe counseling or whatever to do that.

**Interviewer: Thank you. what are the ways that you think one can receive the result of genetic research?**

R5: it's actually good if possible for people donate sample to have feedback on whatever you discover in their blood or other samples. but just like he said, it is important that, that result is communicated professionally and appropriately.

**Interviewer: any other contribution?**

R3: this what we are discussing made me to have the...made me to remember what happened at the dialysis, there we are doing the,,doing a...a kind of a year that we establish the dialysis centre, so now their making some tests, so when they want to give tests to individual they now call the woman that she have chronic kidney disease, but i want to tell you that the woman doesn't use 3 months before he died. because immediately she heard about the result of the test, she collapsed immediately, and this is the person before we did the test she ..if you see how she use to make up, she use to dress, very lovely... and nobody knows that she carry something like that, so I will just try to say that anybody that... there is a test..the test is not ehhh its not ..its something of joy to hear about it, you call the person individually, and what that person can do, someone will direct the person.

**Interviewer: Thank you. What way do you think one can receive the result of genetic research on blood sample donated? Is it through letter, face to face, through health workers? Which way?**

R1: the way I think one can collect result is if one go to laboratory and test was done, one will go back to that laboratory to collect result. That is how I understand it.

**Interviewer: what are you thoughts on returning individual research results and incidental findings? it's part of what you people have said because when we are asking for ways, you've personalised it to yourself. Thank you. what is your opinion on desired feedback on research results? which feedback will you like? I think you've personalised it as well to yourself. then another thing is like what you've said but you can still buttress on it, what are the ways in which you would prefer to get your feedback? and I think you've siad it that if it is not detrimental to you health can be through phone calls, can be.. Thank you very much.**

**then what are the ethical, legal and social issues relating to returning individual results, research results and incidental findings generated from genetic research? do you know of any ethical, legal or social issues that is relating to returning individual research results?**

R5: you know, when you are returning someone result or filing it, you know the health worker has to be very careful, because ehhh most medical cases are supposed to be personal and im sure even the medical practitioners are under oath not to diverge somebody information to another person, you know, so why i said you should be careful is that you can not claim that it was done in error, you have to be careful because ehh the person can sue you if you have diverge an information about the person to a third party, that can harm the person. it doesn't matter how bad it is, its his personal information so you are not suppose to diverge it to the third party, so there can be consequences if errors are made.

R2: I think since we signed something..i signed a consent before I started, I can of course take you up legally. so another thing for medical people is they might need to start involving technology, like you gave us a code here today, you called me 02, so probably after i come..when i come for my ..i don't know, for card or whatever that you guys do here, maybe after the registration, then straightaway assign a code to my name, then log it into your system, then any time you are carrying files about, you don't have people's names on your file, you only have the code, so whoever is going to have the information back, just goes to the code and does that. so it's very important we keep things ehhmmm as tightly as possible.

**Interviewer: any other contribution? as regards the ethical, legal and socials issues relating to returning individual research results? next question: explain your understanding of biorights. what do you understand by biorights?**

R2: Biorights?

**Interviewer: B I O R I G H T, biorights.**

R2: maybe right to life.

**Interviewer: your biorights..explain what you know about bioright. you know we have child right? then we are talking about bioright**

R4: Is it B I O?

**Interviewer: B I O..yes. What do you understand...**

R2: I will just talk it at surface level. in biology we talk about ..biology study of living things and all, so bio life, my right to life, I will just put it that way. so the right i have to live, so bio right . so whether any medical condition in everything that happen, i have the right to live, so you have to do anything possible for me to make sure that am living. I think that's it.

Interviewer: any other person?

R5: ehhh bio right, in context of what we have been discussing, i think ehhh i should have a right to protect whatever thing that comes out of my body to a researcher. so that has to do with.. there should be a legal..i should  protect legally  everything that I donate whether samples or organs. I think that's how I understand it.

**Interviewer: Thank you. how much control should an individual have or can have regarding how their biological specimen will be used in research? how much control do you think an individual should have or can have as regarding how their biological specimen will be used in the research?**

R2: I think everything is still wrapped in the consent, so if i've given you the permission to use it, you should use it within the context or framework of what we signed, so if you are going outside, I can actually take it up with you. so just within whatever we signed just act not otherwise.

**Interviewer: any other person? we talk about how much control we have on the biological specimen that will be used in the research. now i want to ask this way, what right... what right do individuals who provide their specimen for research, have over their specimen? maybe how they are been used in the research, or any profit from research discovery, how much right do you think you as person giving out your specimen for research have over that specimen you are giving out?**

R5: well ehh i dont think there should be any serious right, if you are giving out the specimen, part of you, for research work and you have done that, the only time that there will be an issue if the compromise on what they asked that they should use it for. as long as they use it for what you ask them to do, you don't need to ask them for money, you don't need to ehhh demand for a return, it's not an investment, it's a free will donations, i dont think its an investment. so you should not be asking for any right in terms of fringe benefit or so.

R2: i just thought of something now, because sometimes as scientist people might tend to want to do some unnecessary experiment, you understand, so since you are doing this for human beings, I will also understand that you need to limit it to human beings, not that you are going to be using my own sample with animals, so I wouldn't want to participate in something thats is ehhh a taboo. so just use it within whatever i've signed with you.

**Interviewer: what right did the donor have over what was donated for research, what right did you think the person can have over the biological samples collected for research? any other person? Thank you. what is your opinion about governance and regulation of biobanking? what is your opinion about governance and regulation of biobanking? what is your opinion? maybe as regards the need for ethical committee approval, of future use of stored data, blood, brain tissue resources for research?**

R5: well ehhh, i dont know of any regulation in place but I would think that, you know, every one or every research (inaudible) be embarking on research that has to do with bio should have necessary government regulations and control, you know. I think it's just like you have government regulating so many things, I think they should have..they should come under the regulation of a particular health institute that is government managed. that's what i think.

R2: also i don't know if there's anything on ground now but the stakeholders should come together to formulate something as regards this, then also maybe from this kind of discussion or this kind of research work that you are doing on the surface also, you can get some information that can help to formulate something that we can use, because right now i don't have knowledge of any..if there is ..i don't have any knowledge.

**Interviewer: do you think there's need to set a regulatory board? do you think there's need to set up a regulatory board?**

R2: yes there is a need to set up a regulatory board, like all what we have been saying, there is still a lot of work to be done in our own clime, so there's a need to do that. there's a need.

**Interviewer: any other person?**

R6: there is need , because any organization that there is no control, everything goes.

R5: there’s need to have a regulatory board, you know, because ehh just to avoid abuse. when there's no control over anything, abuse will be inevitable, abuse will be inevitable if there's no control over everything. so, I think there's need to have regulation.

**Interviewer: any other person? do you think there's need to set up a regulatory board?**

R4: there's a need, there's need.

**Interviewer: Thank you. why do you say that there's need? explain possible intervention for implementation of biobanking? what do you think are the possible interventions that we can have for implementation of biobanking? do you understand? ok. what suggestions do you have that help raise awareness and improve attitude towards blood sample and brain donation? we've been talking about it, but we want you to buttress the more on it.**

R2: ok, earlier on we talk about education, then we can also involve nollywood in this , trying to...people are always glued to their tv, they try to show movies that are also focused on things like this, you understand. you can remember the time, in the days of 'i need to know' that Funke Akindele was part of, you know a lot of household use to watch that and gained a lot of things from it about teenage pregnancy, about HIV and AIDS, about tuberculosis and co, so you can also do something like that. formal education, education..you can also...education out of formal settings, then these days of social media, you can also use the social media for this. then of course you can go to communities, maybe through all these CDA meetings, you can also do that.

**Interviewer: what are your recommendations concerning biobanking**

R4: campaigning, maybe you can use campaign, maybe involve people, maybe like youths, youths are people that are agile these days, you use them for like campaigning. people will pay more attention to them. then we can go to churches, talk about, maybe bring somebody that will speak on it, talk about it, mosques, do the same thing. and also we can use fliers, involve of fliers. and also maybe we can just do something like audio recording of... so that you play it, maybe in a gathering or any other places, in a marketplace like that.

**Interviewer: Thank you. Any other contribution?**

R3: you can make a caption. yes

R5: I think ehhh government should also deliberately make an attempt to get people informed about these things. it will not be out of place if the government can make a vote for it under the ministry that is concerned, probably the ministry of health, the ministry of information and culture. you know there should be a deliberate attempt from the government you know to push out this information, and they should sponsor it.

**Interviewer: thank you. no 6? lastly any other major concern or recommendation on the use of blood or brain tissue for research in Nigeria? any other major concern or you want to make recommendation on the use of blood or brain tissue for research in Nigeria?**

R5: ehhhh, for as much as i know, i know we are not so much therefore in terms of numerical advancement and even though we have so many ehhh medical..well trained medical personnel in Nigeria, you know, i know we have ehhh medical institutions that have people that are highly experienced, people that have gone out there to acquire so much knowledge, you know, you discover that most of those knowledge. the only way that they can actually put them to use is still outside Nigeria, why because most of those facilities that can help us..help them to be able to put  those knowledge to use Nigeria, are not just there. if we are talking about biobank, a biobank will require that you have a 24/7 power, and some  other medical condition to preserve them, maybe that are not ..maybe i don't have the knowledge of, so the government has to take frantic effort in ensuring that our health institutions and look at.. you know they should make sure that they work towards the UN benchmark for funding health and education, because health and education they actually go together. they should ensure that they put frantic effort in ensuring that the budgetary allocation to health and to education is up to what UN recommend, you know, so that infrastructure that would support having a bio lab, medical research, help us to do brain and tissue will be able to be sustain in our clime here. Thank you.

**Interviewer: any other major concern or recommendation on the use of blood or brain tissue for research in Nigeria?**

R3: to my own thinking they should please, there are some people that they want to go for all these, the government should try..or can i say each ehhh tertiary, let me say like this FMC now, they should have a space for training on this, so that those people that they have interest of doing it, they will volunteer themselves for the training. so thats my own thinking sir.

**Interviewer: contribution?**

R2: what i just want to say is, currently in our country, we shouldn’t..we know we are not really advance that much right now, even though we are looking at people that are much more advance we can start gradually and scale up later, so that it will not be overwhelming in terms of ehh human resources, in terms of the facilities required and all. they can start gradually and upgrade later, so i think that will help, along the line we would not have a collapse. you know the issue of budgeting and all in this country, so if we start gradually, maybe the people in government will begin to see the need and all, with time they will increase more funding and all, so it's not good for us to have an institution where our brains and part of our bodies are stored like he said and there is break down of power. so, you can start with what you can manage, then you scale up from there. you start with the number of people you can employ, with the number of equipments you can use then begin to build from there.

**Interviewer: contributions? recommendations? Recommendation that you have concerning the use of blood sample for research in this country, in the absence of none we want to thank all of us for participating in this little discussion. Thank you.**
