# Supplementary material for: Research participants’ perception of ethical issues in stroke genomics and neurobiobanking research in Africa
Source: PLoS One. 2025 May 6;20(5):e0292906. doi: 10.1371/journal.pone.0292906 (PMC12054916; doi:10.1371/journal.pone.0292906)
Supplement: S3 File — (ZIP) [file pone.0292906.s003.zip › Files for PLOS ONE - updated March 2025/Ibadan_CAB Members_FGD.docx]

**CAB MEMBERS IBADAN**

1. **Interviewer: Tell us what you know about genetic research?**

004- Genetic research as I know is about some diseases that run in the family or familiar diseases, they are mostly not communicable. There have been lots of or series of researches going on around UCH here and some of our communities too and in that research I know that there are samples that have been taken you know, they bring in the parents, the offspring sometimes to the second and third generation, maybe the mother, the daughter and daughter’s daughter something like that. You know some samples are taken from them, the mother might be hypertensive, diabetic, having stroke, so the will obtain the samples from the mother or the real patient, then the daughter they want to see the factors that may be present in her blood that might be related to that of her mother and then they can take that of the third generation if she has grandchildren and then go ahead to see whether there are similarities in them that might probably bring it up in the child or in the child’s child. So, genetic research is working towards reducing most of these non-communicable diseases in the other generations so that by the time they are able to get all these factors that are responsible, they work towards reducing it in their children since they are not yet coming down with the illness and then maybe in their children’s children. That is what I know about genetic research.

003- I have heard about it, and beyond the medical circle. It has been on traditionally or I may say culturally. In those days, I don’t know how many of our people are still doing that today. In those days, when someone wants to get married, the family of the woman may want to find out some things about the man, is there a particular disease or challenge or challenges that are known with the family. If there is a particular disease that is common they will still try to trace from which side the problem is coming from, and in the course of doing that, the family of the wife to be will now look at it that if peradventure our daughter get married to this man how are we sure that this problem that has been in this family or the trace of this problem will not re-occur in the new family. So, that one has been done, I think the essence of doing that is to find a way of reducing the spread of such a problem so that it will not be further encouraged. So, I know that traditionally or culturally, it has been there and it is a thing that people still encourage today, even in the Christian circle we still encourage people to find out something about their yet to be spouse before they go ahead and marry.

007- I think from the grassroots level, the outbreak of HIV/AIDS syndrome revived the issue of being apprehensive of getting potential partners to come together. Individual parents want to see that they are secured not giving their child away into a family that is invested with HIV virus so that is one thing, and the current campaign about sickle cell is another thing that is making people sceptical in engaging or having marital relationship with discordant partner. So, those are the things. When we say genetic, thinking as a lay person, we think of hereditary diseases that one should be careful in having such perpetrated in the community, that is the way I understand it and my participation in this exercise now is to offer my community sincere observation and what we are thinking as a way out that can always help us forge ahead because you medical people have been telling us that we can always manage some ailments, that is we can live long if we have them, but back to the community we are sceptical about that so maybe at the end of this exercise we will be able to carry something meaningful to my people and may shape our orientation back home.

006- when we talk about genetic research, I am a layman myself, all of us are made up of genes. So, primarily it is not about diseases in my own understanding it is about make-up, hereditary that is passed from one generation to another that is in my own opinion. Now, we should not go to all the technical jargons that the scientists have written, genetic research is trying to look into how our genes that have been passed on from one generation to the other help us in doing something or prevent us from doing something, help us in building resistance against some infections. When we now talk of research in this area, it is trying to look at our makeup and relate it to our past and project it to our future and make some predictions to say that in so year so and so is likely to happen in this lineage if that continues. Simple example is the sickle cell anaemia, it is research that told us that if somebody is SS, he should not marry SS because if SS marries SS they are likely to give birth to SS. So there are recommendations, I don’t know whether it is through genetic research this time around or not you have to correct me, I am a layman. So the SS syndrome and that particular research tells us that if an SS person wants to marry and does not want to give birth to a sickle cell anaemia child, the person should do a blood test and that of the spouse to be and make sure the spouse to be is an AA so that even when they marry they are not going to give birth to a sickle cell child, the worst that could happen to them is an AS. In those days when we had faith and our faith was very strong nobody was going for that but in all the religious organization in the Christian faith today they encourage their members to go for test to avoid such occurrence.

005- Apart from the SIREN I participated in, from the assignment I have covered over the years in the health sector, I have come to realise that genetic research is important to step down the spread of diseases unlike what we have before. More people are becoming aware about these diseases that can be passed on from one generation to the other, for instance the example of cancer, when I started covering I realised that they always advocate that people whose family member had have cancer at a point in time should come for testing especially when they areb40 years and above to quickly detect if they have cancer or not, the same thing is applicable to hypertension, eye problem, glycoma and the rest of them. As this research is been carried out over the years they are helping to pick out people early who might later come down with these diseases too prevent them from coming down. I think genetic research is very good and it is going to help our generation and generations coming after ours.

001-I think that as humans we are made up of what scientists call genes and to a large extent they determine who we are, historically the modern practice of genetic science is linked to the Israelite armies when they want to ignorantly terminate the Jews and they gain a lot of insight into human biological components, that is as far as history is concerned. In law, which is of course my own area of my competence there is resemblance of what we call genetic that is what we call prohibited decrees of consanguinity, this means certain set of people must not marry. In customary law, in the south western part you can’t marry your cousins, before you can marry from people you are related to, that will be about 2^nd^ or 3^rd^ generation. I don’t know to the extent in which that is dying out because modernization have affected many things but I do know that in the north cousins do marry, and in Law in Nigeria we still teach Islamic law but not as customary law anymore. Under the marriage acts which has to do with English law regulating marriage which we call statutory marriage, it is behind that statute that we have prohibited decrees of consanguinity. I think the wisdom behind is apart from the fact that one of the volatile emotion is love and brothers may start fighting about their sister, is to avoid perpetrating diseases. If you marry from a family far from yours you are not likely to perpetrate family diseases.

011- What I know is mostly from what I read from the culture of our people. Genetic research as I read is related to every living organ both man and plants. In agriculture there has been a lot of researches just to make a change and reduce a lot of challenges that may occur due to a particular gene in a plant or animal. When researches are conducted into this area it will give opportunity for development in the management of different diseases and in the management of sound health too, it has given a lot of directions to that. Culture too recognises genetic research that is why some families forbid their children marrying into a certain family. Culture also believe that individual can be part of the oncoming generation in their way of life, this makes it very sound that something is needed to be done about genetic to let people know that it is very important and to continue to make researches about the gene for a better living and development.

009- Culturally, we have been missing way, I want to look at it from this angle that our population is increasing. Then, house to house was not far, you can’t deny your home in those days because you will be easily traced out, who will trace your home now. There are some homes in which they have psychiatry problem how do we trace that? Genetic research is a good research.

010- back then when we are in secondary school, we were taught about gene as hormone being passed from the parents to their offspring given example of Charles Darwin. I am a living witness to research, I remembered when I was young I had appendectomy and later my sister had the same problem. Also I am a stroke survivor, when I did CT scan they said I had high BP, I don’t smoke nor drink likewise my father he too had the same issue. I appreciate you people for bringing this type of program to educate us and to educate the community.

008- when talking about gene, we are talking about blood and I know that blood is life. We have local science and modern science, the problem of local science is ignorance. What you don’t know you ask but our fathers in those days do not know where to ask from, modern science has not been introduced. Talking about modern science, we talk about medical intervention. Concerning genetic research we are talking about, we have to orientate people about genetic factors.

**Interviewer: Tell us about any experiences you or others you know have had with participating in genetic research.**

006- I participated in SIREN research, I participated in the writing of extra-mural grant, the extra- mural aspect of the grant I wrote it, if that is participation.

011- When SIREN project started, we were involved in educating the community first, then we have outreaches. We also participated in ensuring that ethics are followed which was our roles, we also ensured that standard was maintained. I don’t know if that is what you mean by participation.

**Interviewer: what do you know about genetic research in stroke?**

007- If we think in term of high blood pressure and it effect on human physiology, I have domestic experience, my step mother was admitted into this hospital as a result of high blood pressure, palpitating heart, she was admitted and discharged alive but later passed on because the warning given to us was not accurately adhered to, that is the little I can say relating to stroke.

008- Stroke a times may not be genetic, I have a friend that married a troublesome woman, he had stroke due to high blood pressure and depression. So, stroke may not be genetic based on that experience.

004- Genetic research about stroke, like daddy said about SIREN, I too participated in SIREN, talking about prevention of stroke and its re-occurrence, and preventing it in the oncoming generation. I participated in THRIVES too as a nurse education of those with stroke, their care givers and family. So, when talking about genetic study in stroke, it is a totality of looking at some of the risk factors that could lead to stroke especially some of these non-communicable diseases like diabetes, hypertension and then life style of individual that could bring up stroke, and then history of stroke in the family. Stroke is actually a genetic illness but not withstanding we still involve the caregivers especially the children so that it will not just be the stroke patients that we will educate, mostly we educate them about their lifestyle, diet, salt intake, smoking etc which could lead to hypertension. When talking about genetic research in stroke you don’t just face the patient only, it has to involve the family members so that they could live a healthy lifestyle and probably going for screening, early screening of an individual can tell if there is danger signs to be controlled before it leads to stroke.

010- I am a living example to stroke, to me stroke is not hereditary because there is no known case of stroke in my family, I am the first. What usually leads to stroke is ignorance, I said ignorance because we don’t have time for ourselves, we look for money all the time, just to take little time off and check our health status we would not do that. I am always afraid whenever I want to check my blood pressure. That is why I said ignorance and then stress, I don’t take alcohol, I don’t smoke but I know I stress myself a lot.

009- My own little knowledge about stroke as a medical officer is that the structure which God gave to each one of us differs and I can tell you that every bone in human body about 206 in number has expiry date, so as we grow, we should have it at the back of our mind that we are getting old and we can’t be healthy as when we were young, in children there is regeneration but in adult nothing like that. We need to understand what the body is requesting for, we don’t eat nutritious food anymore, and we all depend on fast food. We should know that when we are 40 years and above we have to do less of stressful work.

006- Someone said he is the first in his family to have stroke, so it is not hereditary, there is an adage in Yoruba that says it is only the mother that knows the father of a child, meaning that because it has not happened in a family before those not mean that it cannot happen. Pressure from home, from work, from the community can trigger anything, but the fact is that if I don’t have beams in my stomach, I can’t vomit beans, that is my lay man understanding. So, if pressure is going to trigger anything, it will trigger what we have in our DNA.

001-What I want you to understand is there may be other causes of stroke but our genetic make-up is one of them, we are not saying the totality of what causes stroke is gene, so we have to get it right. Another thing is that one of those things that often lead to stroke is high blood pressure and Africans generally are prone to high blood pressure which means that it has a kind of racial dimension to it, which of course has to do with genetic, so we can’t completely rule out genetic but it may be wrong to say genetic alone explains it.

002-That is exactly what I want to buttress because it is a study we are not here to bring out that conclusion, you will bring out the conclusion after the study, and we can’t conclusively say it is not hereditary but definitely some of the risk factor like hypertension has been traced to re-occur in certain family, so we can’t rule out that fact.

**2. Interviewer: Can you explain what you understand by bio banking?**

004- Bio banking are two words combined, bio which is from biology and banking which means gathering and keeping safe in a place. Talking about bio, it has to do with life, living things, in form of laboratory samples that we take and then kept somewhere for future use.

006- My understanding as a pure layman is that banking the bio, take the bio, give it to a banker to keep for you.

009- My own little knowledge about it is keeping useful parts of human beings donated under consent to be used for another person when needed.

007- His definition has led to another issue, keeping for future use. I am happy that he added the word consent. There has been a problem in the Muslim world for years regarding making use of human parts, they see it as unethical and unreligious because I remembered when I was invited to come and discuss cloning at the university of Ife then, so my research helped the situation because the medical people there said they have been cloning some part of human being in the laboratory and the Muslims were afraid of stepping beyond their bound as far as their faith is concerned, I went round to research on this and my conclusion was that if it is therapeutical it is allowed but situation where by you allotted godliness to yourself is unislamic that is one thing. Talking about bio banking, for what purpose are you keeping human biology, but fortunately for this exercise it is meant to intervene in health issues of human beings by virtue of that we are in safe hands because it is meant to save life, there is a verse in the Quran that says whoever saves a soul saves the entire humanity, if you kill a soul, you kill the entire humanity. The understanding we have about bio banking is keeping human parts for future use.

**Interviewer: How does bio banking operate?**

009- Bio banking has been on for a long time but was not given the full name, like a very close example the blood bank is near every one of us and it has been operating even since research has not been elaborated to this extent, I want to quickly point to one fact to add to what daddy said, there is the man, a Jehovah witness man and their religion says they should not be transfused, it happened here in this hospital, the management had to sue the parents of this young boy, a boy under 18years, he could not decide on his own and the parents insisted that he should not be transfused, the doctor knew that if the boy is transfused the problem is solved. So, the management rallied round the boy and took the necessary step and the boy was transfused and a life was saved. So it is a way of saving life when we look at it the way we suppose though there are a lot of manipulations elsewhere, negligently they can change blood, if those are been addressed properly it should be a way of extending life.

003- let me just say this in line with other speakers, essentially in the aspect of blood banking that my brother just spoken about, I know that it is a way of preserving life what could have terminated someone’s life prematurely bio banking has been a source of help for such an individual. There is something daddy said about the religious aspect of this, I have a case of someone who brought his child to the hospital, by the time they were talking about blood transfusion he said no that his faith does not support that. Along the line he lost the child for that poor understanding. There is another one still talking about the issue of faith some religious belief, I am on a case now, the lady is working in our college, she is having fibroid and it has gotten to a stage that she is bleeding, she was counselled and encouraged to pay attention to medical instructions, by the time she agreed to the that the husband said no that he would not sign for the operation. The lady’s family said this is a way of saving her life, and the family said they would do it after all she is their daughter they signed and the operation was done on her, the man decided to abscond because his religious is against operation, the man abandoned his family, they are still working on that, the joyful aspect of it is that the woman is alive and she is fine. So, the bio banking of a thing we are talking about should be encouraged, but I was discussing with an aged person recently I said what is bad in him after being examined willing say any part of you that is useful when you die be removed and used for another person. I think at a particular stage in a man’s life he should be able to think along that direction that when I am gone release my body to the hospital and let the vital aspect of my body be taken for the welfare of the generation to come. If we can easily donate our library either to school or seminary, I think the part of our body that is very relevant and useful after we might have gone should equally be released, this does not mean you belong to a cult or an evil group, if this thing is going to be useful to the coming generation let it be. I think such a thing should be encouraged in our society.

009- Research will soon die, we work on living things and if the living things we work on says don’t work on living things, if living things say don’t work on me how do we bring out all these things we are talking about. We went for cadaver in UI and we could see that when a dies he is completely useless and everyone wants to raise and train a doctor, if you and I want our children to be trained as doctors and we do not want them to work on you how will your daughter graduate as a doctor. Everyone wants to be a scientist and you do not want anyone to work on your blood, and human blood is quite different from animals, how do we come about facts. So it is something we need to look carefully into though there might be a strong policy guiding what we are saying because I see no reason why I would leave this world and my heart can be used for someone who is under age that can still live some years and I would not want to give such things.

011- I am aware that there is bio banking for research but I am much more familiar with bio banking in the biology laboratory which also has to do with living things. What we do is that we continue to look at what it is in comparison with new bodies that are brought into the laboratory and let people learn from such discoveries and products that are kept, they are just kept for sightseeing, and they are discovery from one group to the other. I think it is majorly something that has to be encouraged.

007- Since bio banking is for saving life, I support it. My understanding of it is like saving for the raining day, like we have blood bank in the case of emergency that a patient is in need of blood urgently if there is no blood bank such life can be lost, that is one thing that Islam play premium upon. Whatever you can do to keep alive is very serious and should be adhered to. Bio banking is like saving for the raining day, even if it is only the data or material that is going to be used, the knowledge derived from studying that materials can always be used to save lives because putting yourself in a position to prevent the perpetration of such illness is a very laudable effort that every reasonable human being should adhere to and that is what Islam stand for. Though we may have some miscreant among the uninformed Muslim cleric misrepresenting or misinterpreting, but I am telling you categorically that from the in- depth study of Islam which I have the opportunity of undergoing there is nothing stopping us from having bio banking. There is this issue of Coroner investigation which I think is related to this, there have been a lot of issues that I have been invited to come and intervene when there are corpse here from the Muslim community and they try to develop a scene that their deceased should not be worked upon. So, by my own humble understanding and research and education, I was able to convince many of my Muslim people approaching this institution. So, bio banking is welcome islamically.

006- Let me talk like an ethic person now, when we talk about bio banking, the people that want to know about the important of bio banking come all the way from America, Japan from Britain to take samples from us here in African to say they want to do research, when they get the breakthrough do they give us the breakthrough on a platter of peace as they get the samples from us? This is an ethical question. Now if we are going to get benefit from banking the bio as we said that we all want it, we must ask them a question what are the expected benefit of this project of banking the bio, thereafter we must ask them how the primary participant are going to benefit from this project in their community, in their lineage and most of the time we don’t have the negotiating power of saying if you cannot meet this criteria, we cannot release our specimen to you. With that they we say if they cannot play ball in Nigeria, they will go to Republic of Togo, we will go to Benin those ones are not as informed as Nigeria, if you are not going to play ball in south west, we will go to the north, not north east this time around where there are no people as informed as you are that we question us. I know there is a research going on here and it is banking the hair, some people are cutting the hair and doing some genetics molecular analysis of the hair, how many of us can cut part of our hair and donate it for research without asking questions. So, the point is that we have to ask questions if we are going to support bio banking, because if it is not going to benefit us we should not support it.

003- overnight I was just meditating on certain thing, and the topic I was working on overnight is how to develop capacity for sharing in other to enjoy God’s provision, I looked at it and I discovered that giving is a challenge, it is a problem, naturally human being does not like to give. I believe when we talk about donating or giving, it is biblical the Bible says give and it shall be given unto you, good measure, press down shaking together and running over shall men give unto you. Giving is a thing the Bible encourage us to do and if we are talking about the important of this, I think the honour is on us to educate our people and encourage them. If you want others to live well, from what you have, from the well of your own blessing you are to share with others. So, if we all agree that this is good, which I agree, I am in support that it is good, in as much as it is good, there should be a way of encouraging ourselves. I participated in SIREN and I see this as a rider on it, something happened in one of our university, we have the believe that every member of our denomination should contribute minimum of a thousand naira towards the growth of the university but it has not been an easy thing to get this money out of my people you will hear them saying why should I give this contribution when I do not have the capacity to send my child to that university, but what I always tell people is this, if your child do not go to that university, he can work there tomorrow. I think selfishness should be taken out of man if we want to sustain this bio banking, and if we want to encourage the important of this program, self must be removed and giving encouraged so that people at the end of the day will be part of the what will promote the health of the generation to coming behind.

**Interviewer: How important is bio banking to medical breakthroughs?**

004- Bio banking is very important, I want to appreciate some of the researchers, I think most of these things they are doing now is basically for sub-Sahara Africa and I am sure if there is any bio banking they want to do now they cannot carry it to US or UK to go and use the organs donated there. They have done most of these researches over there and they were able to save lives with the organs donated through bio banking. Anyway coming to Africa now, we are going to require lots of education because in African countries our belief is that when you are gone the whole of your body should go with you in case of re-incarnation so that the part donated will not be missing when you re-incarnate. We see a lot, especially those with gangrenous diabetic foot mostly Muslims, these legs are bad, they are as toxic as serpent bite, by the time we tell them we will have to amputate the leg because the toxicity of the gangrene is going to eventually kill them, most of them would say as a Muslim no part of their body should be missing. So, they will not give consent until they eventually give up the ghost with the gangrenous leg. What I am saying in essence is if there is no education,

Because so many people they have amputated their legs and years after they are still living, in fact there are artificial leg they can put there and they will still be able to fulfil their future despite the fact that they have amputated their legs. What are we now saying, in bio banking too, education has to come in, the people have to know that donating a part of them does not mean they will be butchered bringing out a part, maybe there is a fatal accident and medically they believe this person might not survive but mind you initially that person must have had a consent, written saying if about to die and there is any part of his body still useful to the coming generation they should remove and keep save. Or someone go into unconsciousness and discovered brain dead, and maybe the heart, the lungs are still in shape, most of these part of the body can be saved and keep for people that have these part in their body diseased. So, what I am saying in essence is education have to come in and our religious leaders too have to come in to encourage their followers. So, bio banking is very important but education has to come in.

011- I believe it is very important because science is research and without research there is no science, and medicine deals with science, they are all interacting one cannot be remove. So, it is basically correct that bio banking will definitely improve our medical science.

**Interviewer: awareness, understanding/perception of brain banking**

002- Definitely that is banking of brain, the only thing I will say is exploring your paper here, it says that we are exploring the ethical, legal and social issues around it. Definitely education has to come in and I think at the same time the researchers have to be held to the highest standard. There is a scourge going on in some part of Africa now where they harvest the organs of people that are living. This is some of the things that causing the wars between the community and medical field which we are trying to close that gap. In Nigeria, we are bad in storing data and it has spread over every sector, some of the things we do at immigration we still repeat at bank. In some other places if you do it once, it is done. I am just trying to say that ethical issues will help the community get more involved because we cannot deny that people hear things and there are certain reality of such things happening all over the world.

009- I want to suggest like he rightly said and we are really omitting his point that are we not working for someone else. This is our country, Nigeria, so the research is here with us to know how useful is parts of our body to each other and globally we can hear what is going on around the way people they are harvesting human organs. And in Africa we are very strong and better than people in other part of the world. So, let us look into it very well, if any research is been brought to Nigeria, let it stay here and let us find out how possible, how preventable, how useable and easy it is going to be when we are going to be in need of it.

004- When it comes to brain banking, I think as my brother has rightly said the dignity of being a medical practitioner must come in, like those that they have to harvest their brain now, it means that apart from the brain nothing is working again in that person. So, it is not a matter of you just find out that this person is unconscious and because he has sign for brain banking or brain donation immediately you just take him to the theatre to remove the brain. They should have done necessary investigation and be able to say apart from the brain that is working in this person no other part is working and can work again so we have to save the brain for use for other younger person, that is one. Then secondly in Nigeria especially, electricity issue is something that matters, even in our morgue here if you are passing the smell that you perceive all over the place is something and this is because electricity is not 24/7, in fact if you go to blood bank sometimes, they have to discard some spoilt blood at the centre at total garden, for the one at UCH, there is a standby generator. So, if now we are going to be encouraging our people to be donating their brain there must be power supply 24/7 and nothing must happen to the freezers and refrigerators or whatsoever where they want to keep them, so they must be able to provide energy and then be able to keep, not that they will harvest a brain and at the end of the day all the part will spoil and got wasted and have to be discarded without being used for the purpose it is meant for.

003- Before I say anything about this, there is a question I will like to ask as a layman. Now, after a person has passed away, for how many hours after can the brain still be working? The reason for asking this question is because of what my sister said that if the medical personnel said no other system of the body is functioning except his brain, how can someone that cannot raise hand sign anything?

004- He might have signed before then. It is like making a will, that if peradventure there is an accident and they find out that my brain can still be useful while other parts of the body are not functioning. In the oversea countries they do that even when they are conscious they donates parts of their body.

004- The aspect is very serious in the sense that caution have to be taken so that we will not be killing ourselves prematurely.

002- The way we are discussing it, it is like the person will die first before they can harvest the brain.

004- Like I was trying to say if we are able to know that when a person is certified dead for how many hours will the brain still function, an hour or two then that is okay but if we are saying that the person is dying or then person is unconscious but the brain is functional I wouldn’t know if that will not generate a fight in the home and in the family even if he had sign. In Africa, we have some traditional belief that if you donate certain aspect of your system, it is occultic, talk less of the brain. I thought of that some time ago, I was in a program with one of my professor and this man is of age but the brain is still there when he was talking , I was thinking, how I wish I could transfer the man’s brain to a younger person so that the thing can continue. Immediately, I begin to answer myself that do I pray the man should die or we want to get his brain from him before his time. These are a number of questions. And I think this requires serious thinking and education before it can be fully supported.

001-I just want to say one or two, someone raised the issue of electricity, I think the problem lies with the institution itself, why would they contract nearly everything out. We have engineers, why didn’t they generate electricity, even simple carpentry work they contract it out, if you are too professorial to do menial work what is the whole essence. This institution has to think of generating electricity, waiting for the government to supply electricity is wrong.

Then the second point has to do with ethics for example if someone has cancer in his private part and you want to do genetic study of it, and you want to keep the private part for the world to see, do you think it will go well with the children knowing that this is their mother private part. It has a lot of ethical issues apart from the fact that we don’t like to give but even sometimes when you want to give, you can’t give the totality of you self, even the religious doctrine only say love your neighbour as yourself not love your neighbour more than yourself. And don’t forget one thing when you raise question about personal opinion, you derive opinion from different sources, religion is one which is may also be your source of moral view on issues, someone who is a Jehovah witness may not believe in bio banking if someone does not take blood you can’t expect him to donate blood. Then, your profession can be a source, someone who studied philosophy will be more critical and analytic about it. Then, someone who has a religious background or anthropological background or belong to cult may not support donation of organs. So, you can derive your opinion from different sources.

Researchers now also needs education, it is not enough to say the community need to be educated, you too needs to be educated. Let me give you an example about Islam, I am an expert in Islamic jurisprudence there is no where someone will tell you it is a must to go to heaven with your complete body, because you are a Muslim does not make you an expert in Islamic law. In fact Professor Anderson and Joseph Charles, Anderson was a reverend father and he was an expert in Islamic law, Joseph Charles was a Christian and he was an expert in Islamic law. So you may be a Christian and be an expert in Islamic law, and you may be a Muslim without having any knowledge of Islamic law, so those researchers too should read about these religions, researchers should be people of plan mind, they need information too, they need education not only those out there.

006- The way I am looking at it we are pulling the cart before the horse, there is a document in front of us which probably you have to take us through to explain, when we are talking about education we need to understand which perspective this research is coming from, we are talking about the ethical, social and legal issues, so other speakers have raised issues about the social norms, if somebody want to give anything at all we talk about blood, they can donate blood, when we talk about kidney yes it is to save life but the moment we talk about brain it is going to generate a lot of issues. So, we have to do a kind of think tank in the social norms to form the basis of how to approach the communities about it, we need to go into sociology, anthropology, history and philosophy to be about to convince anybody to donate his brain. I cannot say as a religious person, a preacher, an educationist or a layman will be able to convince anybody to donate brain, a lot of people we ask me question, when do I join people that use head of human, this is going to generation a lot of reactions.

007- Regarding this issue of donating brain, in the course of our Quran study we pumped upon a story of a professor that was involved in an accident around North and South America, before he passed, he said they should make use of his brain, in order to be so sure before using it on human being his colleagues planted the professor’s brain in a chimpanzee. They succeeded and the chimpanzee started talking like a five years old that is the outcome of the research on brain transplant. My concern now in my study group we always look at current issues from Quranic perspective and misunderstanding from the science perspective, there was the issue of cloning, this will answer the question that how long those a brain survive, it has been established that it is only the non-functioning of the brain and the stoppage of the heart beating that is always concluded as clinical death but the other organs take some days before they diminished or defoliated. Japanese have worked on toad, frog after death for about a month and the frog came back alive, I believe the same thing can be done on human being. I said it in the beginning that anything that is therapeutic is supported by Islam because there are some genius that they need to be perpetrated, so if such is found we can always support making use of them for as long as God decide because we are just trying our own effort and from the Islamic view we think those of us in research even the evil doers we are all instrument in the hands of God. It is an argument among the scholars that God is omnipotent, why are we arrogating whether good or bad everything emanate from Almighty, God is the creator of everything, we are nothing but pencil in the hands of God. We pray that God we use us to be agent of good and not evil, so God can always work through any medium or media. By virtue of clonic study they are working seriously to make human being, the fear is that if those who have died are brought back to live, can this earth plane contain us. So my own conclusion regarding brain banking is to help beneficial brain to be perpetrated, we should not let our gurus just die like that with their brain if we can always make use of the brain, it should be supported. I always sound this note of warning whether we support it or not, whatever will be will because God is ever busy, as we are here today, God brings us together, God works through every mean, that is what we are made to understand, so whatever will be will be, if we do not do it here some people are working tirelessly on it. I could remember the days we booked at the cyber café overnight to browse but now we are browsing at our convenience in our rooms and connecting to the whole world, it is working of some group of people with God working behind them, we need to understand the way we relate with our religion and science.

**Interviewer: awareness of any law or policy guiding bio banking**

001-As far as Nigeria is concerned, there is no specific law in term of Acts of the National assembly or law of the state, also we do not have a specific policy, we have health policy generally but we do not have a specific policy on bio banking. However, some of our research the way we conduct them can give rise to legal consequences. Now Nigeria is not the only country, because it is a new phase it is developing, even in some other part of the world they keep on changing their law, associations regulating them keep on changing their ethics because they also have rules of ethics. So, it is not really a stable field, it is developing and growing.

011- I know of specific policies within the institutions, you can just do things as a researcher without an approval. So, there are local policies and guiding rules which must be obtained before you can go on.

007- There is the issue of ethic committee, it is a global issue and Nigeria is part of that global entity that brought about that law. So, some of us are much aware, and writing exams for membership of research committee in the science world. So, there is standing law regarding what we supposed to do to safeguard the interest of our people. I am much aware of that personally because I belong to that global research ethics committee.

001-let me just add one or two things to that very quickly, now if you are conducting research, I am supervising PhDs, before you do anything on human or whatsoever you must get ethical approval but that is not specific for bio banking, even university can approval and it will still give rise to legal consequences. We are talking of the one laid down by the government, which you can rely on to safeguard your interest, we don’t have that on ground. Universities have ethical committee on that, you seek approval but the ethical committee itself could be sued that is what I am saying. When we are say specific, it must relate to bio banking itself, in physics they have, in biology they have, even in law we have, there are certain questions you cannot ask people if it is going to violet their moral sense, their sensibility, they will say no, this question is out of it, you can’t ask this, then you can’t perform this with children below this age. We have all these ones, but when you are talking of the one specific for bio banking, no.

006- Let me add to that, in ethics we have all those regulations, but I am not a friend neither am I a foe, I want to put it to you sir that where there is no law, is there any sin?

001-Listen, I clarified something, I said where you do not have specific law and policies, your actions can give rise to legal consequences. For example the issue of brain, baba raised one question, you need to have died, if somebody had truly signed and the person have not yet died really and then you harvest his brain of course you are a murderer, law of murderer is there that have given rise to legal consequences. For example we have consent form, but you didn’t explain everything in the consent form that is what we call misrepresentation in law which is tortious, there is what we call law of tort.

006- That is law of wrong

001-you see criminal is also wrong but that is not criminal, it is tortious that means it is a civil wrong. So, the very fact that this person does not have a true representation of what he is giving his consent to, it will give rise to what we call misrepresentation. There is also what we call doctrine of constructive trust, for example, I said I will get something done for you, especially those that use agent and the agent acted against their interest, the law says there is a trust relationship. If you get blood or tissue from people and you keep it, the very fact that it is in your custody there is what we call constructive trust, even if there is no agreement, there is that principle of constructive trust, you are holding it in trust for this people and you must not do anything against their interest. That is why I said though we do not have a policy but your action can have legal consequences.

006- That bring me to the issue of ‘baddoo’ boys what they do is that they cut people head and harvest their brain and the ‘baddoo’ boys are in Nigeria. So I want this research team to look into the ‘baddoo’ boys’ syndrome before they start to think they are ‘baddoo’ boys.

**3: Interviewer: What do you understand by precision medicine?**

006- My understanding on precision medicine is that you don’t use drug of headache to cure stomach-ache that is precision, that means if anybody is hungry give him food don’t give him water that is precision, so, meaning that what you have to use to cure a particular sickness is what is designed to cure that particular sickness that brings me to one issue our sister raised the other time, she said somebody had diabetes and they said the leg was going to be amputated, I beg to disagree. Some years back in this very hospital my father had diabetes and a gangrenous foot and they said they wanted to amputate, my father refused against doctors’ advice, do you know that it was a man in a local hospital that took care of the leg and the leg was functional properly until my father died, he used about 20years after that challenge. Unfortunately the person that took care of the leg is now dead because he was elderly then I would have referred you to him, I am not saying he did that with hospital drugs alone I don’t know what he used maybe he used some local drugs whatever or not I don’t know, all I know is that he treated the leg and the leg was not cut.

011- My understanding is that you are looking at issues and you are taking steps based on findings. For example people that have stroke, you cannot just treat everybody with the same treatment because they are all stroke patients, there must be investigations, there must be understanding, and there must be findings before you can decide on type of treatment.

004- Precision medicine can be applied to stroke, like we are talking about genetics now from their discoveries from genes of an individual what might have gone wrong to cause the stroke in the individual or the hereditary factor. So, based on these now, they can use it in the management of others, having done the researches finding out the basis for the cause and working towards using the findings to procure management.

003- I believe precision medicine is okay, it is good but however there are questions that one might need to ask. One, who is prescribing the medicine, and what is the knowledge he has about the problem on ground before prescribing, today for instance to go to motor park and someone is feeling feverish, he will not go to the hospital, he would rather go to a vendor that will give him several drugs in one to use in a go (akapo), prescribing medicine but this a wrong person prescribing without diagnosing what is wrong with the person. So as good as precision medicine is the aspect of who is prescribing what and for who is another thing that is where the issue of self-treatment must be discouraged as much as possible.

**Moderator: it is precision medicine, from precise.**

003- Thank you very much. It is okay, at least we will know that this is what is needed for an individual, but when it comes to the herbs aspect the problem is that this medicine can take care of four problems. Precision medicine is a good thing.

**Interviewer: awareness of any law guiding precision medicine**

011- I would hide under the National Health law because under the national health laws those things are stated, you cannot be a professional and claim to be a consultant, you cannot be a professional and continue to take care of people, and there are procedures set down for all set of things that you need to do in medicine that must be guaranteed before you take a step. That is why there is a different between a medical professional and a quack, a quack operate under no law but under the national health laws all these are stated for all professionals in medical professions, surgeon, community medicine person or whatever.

**4. Interviewer: What do you understand by brain donation for research purpose?**

**Interviewer: What factors can inhibit brain donation?**

004- Personal factors, many people do not have the courage

001-Individual wellbeing, cultural factors, religious factors, social factors, professional

006- Not to be disclosed. Some people will tell you I know the reason why I don’t want to do it but I cannot tell you, confidential factors.

**Interviewer: Factors that can promote brain donation**

009- Education, if you are not informed, you will deform. Get people properly informed

004- Public enlightenment using the mass media and we can go from community to community using their community leaders to get to the members of the community.

007- Content of that information is very important, the benefit of brain donation must be emphasized, what is there for them to gain and how would their community benefit from doing so. So, it is important to make it clear to all and sundry

006- What I want to say please correct me if I am wrong, I think pace setting by the principal investigators should be number one, if you say I should do something do it first and show evident that you have done it.

004- Target religious leaders too, they can get to their members.

**5 Interviewer: Share with us your opinion and thoughts about blood sample donation for stroke genetic research.**

003- Personally I can easily donate I practice that, but the only barrier is that I must be sure that what I am giving out will be used appropriately or correctly, another thing is that the blood I want to donate is well preserved, we are have talking about that since because if one get to the blood bank at total garden and you discovered that the gift that some people gave are messed up you will not be encouraged, whenever you are called upon to do that again you will turn it down, because the one you gave before was not well preserved, I think that should be looked into.

004- I am willing to be involve, to partake in blood sample donation for stroke genetic research but the demerit now is the issue of not giving back to the participants, you see once this is carried on, there should be a way of relaying back the findings to the participants. Then the merit is that getting involved will give better chances in future to the management of other stroke patients.

011- I am willing. For people to be willing depends on how much they are informed, then orientation and the back ground of the people are very important, what does the community think about giving blood wont they think about witches and witchcraft that will suck the blood being taken, if you go to the to the community you will see them shouting you take 2 bottles it is too much do you want to go and use their blood, there is the perception that they could be harmed.

009- The people that will collect the sample should be given orientation, now you want to take sample from me don’t try to bribe me or be rude to me, if you don’t want to give go on your useless way. You see to give blood or whatever is all about life. They should be friendly, they should not come with the mind that if you don’t give that is your problem we only want to help you.

011- Some people are willing but they can’t tolerate needle, so if there is any way to take sample without needle they should explore that.

008- The volume of sample to collect must be taken into consideration because I could remember in SIREN project we went to a community and the people in the community were saying the sample collected from them was to much. So, they have to put that into consideration.

005- I am happy to be part of this study because I know the outcome is going to be beneficial to the whole country, and then apart from that it is going to enlightened members of the public, so many of them don’t know all we talk about like bio bank and all of these they don’t understand like we connected it with the issues of rituals and all of that, it is going to debunk the rumour that the sample that have been collected will be used for the benefit of the people not for ritual purposes. So, I am happy to be part of this study.

006- Let me say I am non-compliance to say I am not going to support donation of blood for this research, why? I am not going to support because I know that most of these samples collected are not banked here in Nigeria and they are not going to be analysed for research purpose in Nigeria, they are going to be exported, we are just like sample collectors here they are going to take it there. I am going to support donation of blood for this research only if the funders are going to ensure that the put up a laboratory here where they are going to do analysis here and develop the capacity of researchers here to analyse those data and it is only the end product of the analysis of the data that would be shared. Otherwise I am not going to support it.

009- Another thing to be considered is when you take a sample from someone and the person has the tendency of coming down with a stroke, just to add to what my brother just said, will they bring the result or the result and samples are just for fun.

**8. Interviewer: Tell us what you know about informed consent.**

004- I think consent is respecting an individual and taking a support for whatsoever you want to do for the person either as a medical person or as a researcher, so in which you respect that person and the person gives his or her full support into what you want to do especially if he want to be your participant or your patient. Having said that, in informed consent, everything have to be explained about that particular thing you want to do either the treatment nor discoveries of illness of that person, the type of treatment and the choices all over which either the patient or that person can give into, that is informed, you have to inform the person properly. Then, there are different type of informed consent, it can either be verbal in which the person can say okay, I support verbally without writing anything down or it can be written, whereby when everything have been explained to you, you put your signature and date.

003- let me say briefly that informed consent is written, it is ethical, it is legal and also social, if we try to get consent or someone has the knowledge of what it means to give consent over anything, it means once it is being carried out number one the researcher has the legal backing if my learned person here is in support of what I am saying, and socially it is in writing. I think informed consent should be encouraged, we should not take ourselves for granted because in a situation where somebody just give you a story and you go ahead to use that story without the person backing even religiously we don’t do that, when someone share something with you and you want to give it as example under normal circumstances you have to approach that individual and take permission that the thing that you said to me can I go ahead and tell it as a story or use it to support the message I, want to present if they say no, then you don’t have the right to use it, if you use it you can be sued. I think it is a thing that should be encouraged and it is a thing that is necessary in every research.

006- Let me add to it, the issue of informed consent is very complicated, the language of informed consent should be very explicit so that nobody would say I did not understand a particular phrase or a particular clause that is why they say the language of informed consent a primary six certificate holder should be able to read the document and understand it, not that you tell me bio banking that terminology should not be in informed consent instead you say ‘’yiyo opolo yin kuro ninu agbari yin lati fi pamo fun ise iwadi’’, the language must be very clear, that is number one. Number two is that you must translate it to the local language of the person. I was joking with a colleague researcher in the UK recently, he wrote something and I wrote to him and said he is a veteran, he replied asked me if he is that old to be called a veteran, I said the last time I checked I think a veteran could mean an expert, I said well of course English language is not my mother’s tongue. He wrote and said he think I was write that he was just joking. You see, there not use a language that would be ambiguous otherwise there could be problem, but translate it into a local language and make sure that everyone understand. You know when we got here you said I should sign I said no, I have to read, understand, digest and agree before I can put my signature, no one can coerce me or instruct me to do what I don’t want to do that is not the process of informed consent, it is not good to coerce anybody, that brings us from consent to accent. Somebody that is mentally okay and of age that could take decision for himself is the person that could sign consent, but otherwise somebody gives accent on behave of that person, underage and people that are not medically fit to give consent.

**Interviewer: : Types of informed consent preferred**

009- To me the dynamic is preferred

001-If you are a researcher you will definitely prefer the broad consent it makes your work convenient and easy, if we take your consent now we don’t need to bother again if we want to do a similar research we can use it, we don’t have to get back to you, that is the broadest level of consent that anyone can give, I have given you my sample you can use it to any length, it is convenient for the researcher but it does not protect the interest of the participant. The restricted protect the interest of the participant more but can be easy for the researcher, because to you the sample for a similar research, you have to go back to the participant to take another consent, if the person is dead at the time it means the sample is useless. Then for the tiered I think to a large extent protect the interest of the researcher as well as the participant, the last one favours the participant completely, but sometimes the outcome of a research is not immediate, it can take 12, 15, 20 years. So, you give sample today after six months you are calling to know the outcome, then a year later you are calling to know the outcome, outcome that may not come in twenty years.

I think the tiered somehow tried to balance the researcher does not have that blanket power and the participant is not completely left in the dark as in what is happening. So, the tiered on somehow.

006- For me I prefer the restricted especially in the African settings because when you give them a mile, they take several miles.

007- Well restricted one is my choice, it is universal, it is not only in Africa, it is all over, it is human nature, we don’t know our limit.

009- To me I prefer dynamic

011- I prefer the dynamic, it is not that you will go and sleep with the researcher you can give him a call.

008- The dynamic consent

005- The dynamic because I can call the researcher anytime and ask him what is happening to my sample.

004- Well let me say I will prefer the broad type because as a medical person, if this is given and a similar research comes up, it is not necessary to back to take another consent since it is to help humanity definitely I think the broad one would be better. Then I would have preferred the dynamic but has he rightly said a research can be on for 15, 20 years even more than that before the conclusion of that research and when you keep on bothering the researcher one will nearly become a nuisance.

003- Personally I will prefer the dynamic.

002- I prefer the tiered, it balance the interest of the participant and researcher.

**Interviewer: People to be involved before participation**

004- Relating to me or how

001-You mean if for any reason I don’t have capacity to give consent, if that happen I won’t give anybody the right to give consent on my behave it simply means I opt out, no body give consent on my behave as long as no one can decide on my will for me.

006- My brother have spoken as a legal authority but most of the time the head is there but there are some necks holding the head at the right, left and centre, so in my humble opinion I am married, I will involve my wife and my children, you know by the grace of God I live in a girls hostel, they can give consent on my behave.

**Interviewer: Data in the incident of death and why.**

002- I think we should use the standard procedure here in incident of death it is always the next of kin.

**Interviewer: Support for generic consent for community.**

No response

**Interviewer: What is your opinion on storage of blood sample and blood fractions?**

No response

**Interviewer: Tell us what you know about sharing of data, blood/blood fractions, brain images (CT/MRI) as well as brain tissue samples.**

**Interviewer: opinion on sharing data, blood samples, brain images or brain tissue samples with other researcher locally and internationally.**

**003**- Personally I want to say the data gathering in a research like this can be shared with others where it is relevant, just like what my brother said the other time, once it is carried out and we have the outcome of it, then it should be for the benefit of others and it can also be a source of raw material for another research for instance SIREN research is gone and we are seeing a rider on it. What we had from that study can be relevant for this one and it should be allowed to be used. Data can shared locally and internationally.

**Interviewer: commercial or non-commercial use of sored data, blood fractions, brain images and brain tissue.**

003- Commercialising of data, I think if commercialising of data is going to be, the participants of that research should be taken to heart. If they sign consent and you have their consent like we said at the beginning I think there is no stop in doing that, it is a good thing

007- we must be careful in getting involved in a way like people who are commercialising blood donation, if material incentive is not properly handled it can be misconstrued to be a kind of those who are running after human parts to make money that is, I sell my part to you give me a million dollar or what have you. So, the ideal is to have the mind that you are contributing to perpetration of humanity.

**Interviewer: Share with us your thoughts about return of individual research results and incidental findings**

006- I think if you find out about anything in research you should get back to the person where you have taken the sample, and there are samples that you take that you can never publish, if you discover that in Y village, the people there are because of molecular genetic analysis they have the tendency to run mad, it is unethical for you to ever publish it and the research ends there. So I think we should take individual life into consideration when you are reporting, but if you go to the person and the person now say you can publish it anonymously that is ok. So there should be feedback otherwise the research will not go on well.

**Interviewer: way prefer to get feedback**

006- Person to person interaction

004- It can be sent through letter.

006- Letter is not the best because somebody will type the letter another person will staple it another will deliver it.

007- When we are talking of old time, old time is when you delegate such type of duties, most researchers are used to the computer, they can handle it on their own without seconding it to any secretary, typing it out because not all of us are connected to the internet, hard copy of the finding can printed out and posted by the researcher himself or herself or personally handed to the participant. There is no big deal about it.

009- The result should be stapled.

006- Your result can never be opened in your presence anywhere in this hospital, they will staple it and give it to you to go home and read it or take it to someone that can better explain or interpret it to you.

Probe: what are the ethical, legal and social issues relating to returning individual research results and incidental findings generated by genetic research?

010- A lawyer is here.

006- The legal issue is that it has to be extremely confidential, daddy have said it. The PI himself should handle the result. There is a problem, you will be eating fat in all these research because a lot of people have access to individual data, which is not supposed to be so.

001-let me response to this, when some of these questions were being prepared I read some of these issues, now they have international standard practise about collection of data, about saving them. If the scientist save them in line with international best practices they are not likely to be held liable, but if not, if there are lapses on their part, obviously they have opened the front gate of litigation for themselves.

**Interviewer: Explain your understanding of Bio rights.**

007- Bio right is an extension of human right because I have the right to protect what I think is mine. So, it is an extension of human right and whatever law governing human right should also govern bio rights.

Probe: how much control can individuals have regarding how their biological specimens will be used in research?

007- The answers given earlier on has answered that, when we are talking about consent, I think I subscribed to dynamic which says the participants should be revisited from time to time. So the participants has the right to be revisited.

13. What is your opinion about governance and regulation of bio banking?

007- That is the essence of having government in place, they should always legislate to what is beneficial before any law should be formulated there should be public hearing whereby something shall be added to it from the people in the society, so once the society have subscribed to such bill the legislator sit on it and pass it, and the governor of that particular location will append his signature. So, that is the essence of having government in place. So governance is very crucial to this because they are to produce law guiding this exercise.

14. **Interviewer:**  **Explain possible intervention for implementation of bio banking?**

001- First and foremost, intervention begins with having good laws in place that encourage the practice at the same time protect the interest of the participant. Apart from good law, the government have to spend enormously on research, when the government fund research enormously, that is also an intervention. With those two things in place, the sky will be the limit.

007- Sincerity of the parties concerned, sincerity is very crucial. They should be sincere to the people involved and to themselves.

009- The government should be in full of control, they should be monitoring it, not just funding them and not looking at what they are using the money to do. They should monitor it.

001-They should have a monitoring team. Accountability

011- Funds for researches should be made available to the appropriate authority and utilised

004- Power supply 24/7 and utilisation to the appropriate channels

15: **Interviewer:**  **What other major concern or recommendation do you have related to the used of blood for research in Nigeria.**

004- I recommend that blood samples taken for research especially in stroke management should be encouraged because what is done in this generation now is going to help other generations to come. I recommend that it should be practice.

005- 24/7 power supply otherwise the samples are going to waste.

006- Like they have said, power supply and awareness, let people know about it, and if there is any issue they should go to the appropriate authority to make their complaints.
