# Supplementary material for: Research participants’ perception of ethical issues in stroke genomics and neurobiobanking research in Africa
Source: PLoS One. 2025 May 6;20(5):e0292906. doi: 10.1371/journal.pone.0292906 (PMC12054916; doi:10.1371/journal.pone.0292906)
Supplement: S3 File — (ZIP) [file pone.0292906.s003.zip › Files for PLOS ONE - updated March 2025/Ibadan_SIREN Caregivers_FGD.docx]

**FGD Caregivers (UCH)**

**Moderator-PE**

**Notetaker-DA**

**Interviewer: Tell us what you know about genetic research**

01-what I can say that I know about genetic research is that those days when we were in school, we learnt about gene, we were taught about chromosome, the pairing of chromosome, if a father is short it is possible to see a child that will be short in the third generation after the father. I don’t know more than that

02- I don’t think I know much about but from my understanding, I think it might have to do with my gene, my mother’s gene, my father’s gene, and because of genetics there are some sickness that might be passed down maybe high B.P or something, that is the way I see it.

05- I think, just like the other person said, it is something a child inherit from the parent either the father or the mother, or from the great parents maybe blood genotype, blood group stature, anything the child takes from the parent.

**Interviewer: Tell us about any experiences you or others you know had participating in genetic research**

All – no experience

**Interviewer: What do you know about genetic research in stroke**

02 –Maybe they want to find out what the person had that might load to her having stroke.

04 – Maybe the person does not have enough rest or does not sleep well or think a lot by putting a lot of concerns on his mind.

06 – If the person works hard and does a lot of strenuous work, they may want to find out why the person has stroke.

01- They may want to find out maybe the stroke was caused by high cholesterol in the blood which might have blocked the veins and blood could not pass through.

05 - It is possible a times that the stroke is inheritance maybe the mother or father of the person has it.

01 – To prevent the occurrence of stroke

05 – To know the exact cause to each individual, you know what causes it for one person may not be the cause for the other person.

02 – to know if there is deficiency in the person that made him to come down with such, so that it will not repeat itself in another person, so when they do the research they will be able to give the person something that will stop it.

**Interviewer: Can you explain what you understand by bio banking**

01 – like blood donation let say someone needs blood and you are called to come and donate blood for the person to save the person’s life.

04 – Kidney too, maybe someone’s kidney is bad they can change it.

02 – like maybe organ donation, or harvesting of organ from a sick person to keep somewhere

5– I heard the case of a woman helping another woman to carry her baby in her womb till birth, something like that

**Interviewer: How important is bio banking to medical breakthrough?**

01- it saves life

03- It saves life

05- It helps in research

**Interviewer: Belief/thought/opinion relating to bio banking**

02- some people believe it is not Biblical, so anything that has to do with human pants should not be tampered with.

05- some people think, like the kidney for instance if they donate one of their kidney there may be complications.

**Interviewer: Awareness, understanding/perception of brain banking**

01- That should be after death

04- I think this can only be possible after death.

**Interviewer: Awareness of policy or law guiding bio banking**

01-Yes, like somebody that wants to donate blood now he has to pass through some examination, they will take sample and test for HIV or other diseases in his/her body that will not allow him/her to be part of that process

02-In addition to what he said there is age limit for blood donation and if a lady is menstruating she can’t donate blood.

**Interviewer: Can you explain what you understand by precision medicine?**

01- what is happening to one person is different from another, the drug that would be prescribed for me may be different from that of my brother even though we came out from the same womb our makeup may be different.

05- Like the issue of stroke from my little knowledge about it, if you put 3 people having stroke together, the cause of each will be different if you carry out test, so because 5 people have stroke it does not mean that they have to be treated the same way, they can’t be given the same treatment because the cause of one is different from what led to the stroke of another person.

04-Just like our sister said, what I think about it is that many people cause sickness for themselves due to what they eat or drink, like people that drink alcohol or do things that are not healthy that can cause disease. So people that drink alcohol and had stroke will be given different drugs from people that do not drink.

**Interviewer: Benefit of precision medicine**

01-It prevent relapse of that sickness

**Interviewer: Is it important in Africa?**

01-Yes

**Interviewer: Source of information**

01-I learnt about it when I came to the hospital from doctors’ discussion with the patients.

02- I do research from the internet

**Interviewer: Awareness of policy or law guiding precision medicin**

01-whoever is going to be involve in precision medicine must be a licensed medical practitioner.

**Interviewer: What do you understand by brain donation for research purpose?**

02-like I said before things like this will aid in future research

05-It is not the language I don’t understand, it is the term. I have never heart that one can donate brain

04-Is it possible for someone that is alive to donate his/her brain?

**Interviewer: Share with us your opinion and thought about blood sample donation for stroke genetic research.**

02-They will take blood sample from stroke victim and they will assess it and do whatever they think should be done to it, to see what could have cause the stroke.

01-They will take blood sample and check the lipid to know the amount of fat in the stroke patient. As the same time to know whether the patient has a high sugar on his/her blood.

05-I think the major purpose is the time they do research and ruin test on the blood, it will help the research in further treatment of other people. By the time they run the test they would be able to know the cause of the disease and this will help the research in the treatment of other patient in the future

**Interviewer: willingness to be involved in stroke genetic research**

01-why not if I am fit to donate blood

05-if it is save, I will be willing to donate, if it will not affect the person that is donating negatively.

03-Yes, so that I can know if I have any problem

**Interviewer: Benefit of giving blood for stroke genetic research that could promote your willingness**

05-it is beneficial to the person that is donating for the research in the sense that it will help the person to know his/her health status, you know there are time you think you are okay but you are not okay. So, by the time test is run on the blood they will know and there is a treatment you are supposed to have you will know.

**Interviewer: Barriers that could hinder blood donation for stroke genetic research**

-05-if I have a low PCV

03-I am scared of being pricked by needle

02-If someone has a life threatening disease

**Interviewer: what can you say about your family member or other member of your community willingness to give blood for stroke genetic research.**

06-I don’t think they will want to give blood because they will be thinking there will be side effect.

**Interviewer: what could be done to make you and more people give blood sample for stroke genetic research**

05- I am not a medical people I don’t know much about all these, the medical people should enlighten us about the pros and cons, the benefit if you do it, and if you don’t what are the other sides to it.

01-proper orientation, go to churches, to mosques, markets and let the people know the advantages and disadvantages

03-organise seminars in schools, universities to inform them.

**Interviewer: Tell us what you know about informed consent**

02-what it means is that you are informed about what you are doing before

You give your consent

04-It is a way of enlightening people about the program on ground

05-It is like consenting, appending your signature, telling the person that you are ready to be involved; but with the little knowledge I have about it is that if I decide to leave at any time I can leave

01- It would aid the research.

02- It is just like you know what you don’t know much about something you can’t make an informed decision, if you don’t know what you are consenting to. That is how I see it

**Interviewer: Type of informed consent preferred**

01- To me broad type is preferable.

02-Broad

05-Broad

03-Broad
06-Broad

**Interviewer: Reasons for choice**

05- The condition is that one should be pre-informed so that you know what you are consenting to

01- When I am no more, they can continue to use it once I have given it and consented to the use

02- It is good because it will benefit mankind

01-It will say life

04-It will be beneficial to a lot of people

05- They have said it all, I know of someone that was not okay financially and needed blood transfer someone just came to his recues and donate blood. So, there is nothing wrong in it so far the person is fit.

**Interviewer: Tell us what you know about sharing of data, blood/blood fractions, brain images as well as brain tissue samples**

01-I think the essence of our sitting down is because the information we are giving will be useful, so I support sharing of data internationally, it should go out. We also read about outcome of researches on the internet

05-Data can go to any length as for as it is useful

04-Data that is useful in Nigeria can as well be useful abroad, so it can be share internationally

**Interviewer: Share with us your thoughts about return of individual research results and incidental findings**

01-Through WhatsApp

02-Through phone call

03-Through SMS

05-Information like this should be made confidential, it can be sent through a medical personnel to come visiting and deliver and explain the result or we can be invited to come to the hospital, it is not something they can just send through SMS

**Interviewer: what are the challenges of returning individual results.**

04-what I perceived as challenge is when the result does not come In as at the time we are excepting, and when it comes we do not know how to go about it.

**Interviewer: What are the ethical, legal and social issues relating to returning individual research results and incidental findings generated by genetic research?**

01-Legally, individual research result should be returned by people that are experience in medicine that can explain the outcome not just anyone.

**Interviewer: Explain your understanding of Biorights**

01- I think bioright means an individual has the right to take care of his/her health; sleep well have enough rest and care for his/her health

01-I don’t think individual has further right on the blood once he has given the blood.

04-His right ends when he gives the sample once he has any consent and append his signature.

**Interviewer: How much control should/can individuals have regarding how their biological specimens will be used in research?**

04-I don’t think he has any control again since he has willingly given the sample, it is now left for the researcher to use it the way it should be used.

01-He does not have much control because the person that gives it does not even know how to use it, it is the people that collected it that know what they want to use it for

05-The person that given the sample will not be there again when they are using the person; it is left for the researchers to follow the written agreement he consented to, they are not allowed to do otherwise.

04-The person that donate has the right to ask the researcher how he is going to benefit from the research.

06-He has the right to ask for his benefit before he signed anything

05-The greatest benefit in a research is the fact that it saves life and is a service to humanity

**Interviewer: What is your opinion about governance and regulation of biobanking?**

01-My opinion is that the governance is not encouraging, for instance if you bring a patients to UCH, you will donate blood and you still have to pay; they said the money is to screen the blood you donate; this is not encouraging.

02-yes, there is need for a regulation heard so that to monitor the use of materials donated.

05-There should be a strict law that will guide the use of organs donated, so that it will be use solely for the purpose in which it is donated for. I heard a case of someone that donated kidney for a relative and eventually they didn’t use the donated kidney for the person; not in Nigeria anyway. So, there should be a strict law guiding it so that organ donated will be used for the purpose in which it is donated for.

**Interviewer: What suggestions do you have that can help raise awareness and improve attitude towards blood sample or brain donation for research and encourage people to adopt the practice?**

01-People that are trained should be involved in the process not just anyhow people and there should be law backing them and guiding them as well

02-They should go to school market public places and generally where people gather and sensitize them on the benefit of brain donate.

03-They should do public enlightenment.

04-Many people are at the local areas that do not know anything about it; they should start the enlightenment program at the grassroots, this will help

05-The major thing is public enlightenment, go to public place and enlightened them. Some people when they hear blood, they assumed it is going to harm them or have negative effect on them; so once they are enlightened; go to rural areas, school even some educated people are enlightened. For instance many people do not know that one kidney can function as well as two kidneys. If there is enlightenment people will be willing.

06-They should go to rural areas and talk to them, 4 they are willing they will donate.

04- people should be encouraged, they should be given something.

05- people that donate blood should be given something not just incentive maybe milk or something that will make him/her regard strength.

01- House to house orientation.

**Interviewer: Any other major concern or recommendation on use of blood or brain tissue for research in Nigeria**

01-They should make posters and billboard and put it in hospitals both public and private hospitals.

04- Make use of the internet

01-Television too can be used to publicize it

05- If information like this is placed on the internet it will go a long way

01-Produce pamphlet and distribute it to people that can read, schools, tertiary institution.

04- Organize seminars and take it from place to place
