# Supplementary material for: Research participants’ perception of ethical issues in stroke genomics and neurobiobanking research in Africa
Source: PLoS One. 2025 May 6;20(5):e0292906. doi: 10.1371/journal.pone.0292906 (PMC12054916; doi:10.1371/journal.pone.0292906)
Supplement: S3 File — (ZIP) [file pone.0292906.s003.zip › Files for PLOS ONE - updated March 2025/Ibadan_SIREN Stroke Cases_FGD.docx]

**FGD CASE (UCH)**

**MODERATOR-PE**

**NOTE TAKER- YO**

**Interviewer: Tell us what you know about genetic research**

01- Genetic research in when you try to find out how my gene is related to my father’s gene. That is to say if you have an hereditary problem, how it can affect your offspring, through research it will be known if your children gene is related to your gene. For instance there are people with SS that always have problem with their health; this is a big issue. The background of it is when one of the parents is SS; and the other AS or they are both AS, the best is for an AS or SS parent to marry AA. This problem arises from the gene, through genetic research the issue can be prevented. In the olden days when there are no research many people have fallen victim of birthing SS, but now people are enlightened they go to the hospital to check their blood group and genotype before they marry

06- Gene is inside the blood, it has to do with growth ability to give birth to male or female child. Like now, maybe high blood pressure runs in the family that is someone has it in the family, some of the offspring might likely have it; or even stroke. So, gene has to do a lot with human being life. That is the little I can say about it as a layman.

**Interviewer: What do you know about genetic research in stroke?**

06-The research about stroke has been going on for a very long time, what they mean by research is to verify what leads to something. For a stroke person now, maybe he has a severe headache and other symptoms of stroke like dizziness, pains in the leg and arm. So, they will find out what exactly is causing all these maybe stress, lack of sleeping, smoking, drinking all these can lead to stroke. So that is what the research is doing to get to the root of these and find out proper and appropriate answer to stop to the rampart of stroke in the society

01-what I can say to it is that if someone notice that he/she has headache from time to time, stroke is coming. So, If someone has high blood pressure it is better to check one’s blood pressure all the time and take drugs and that is not done the pressure can lead to stroke and goes to the hospital and use drug stroke will go. I don’t agree that stroke is genetic because there are people that stroke victims are in the family and they do not have stroke. I, for instance, nobody ever had stroke in my family I am the first person that would have stroke in my family. It is possible for parents not to have stroke and their children would have it. So, stroke is not genetic at all.

**Interviewer: What do you think are the roles/benefits of genetic research in medicine?**

02- It is good to do research to put an end to stoke because stroke is very common in our society now. So, it is good to do the research so that we will know the drugs to take to prevent it, or vaccine to take so that we will not have it, it is good to do researches about stroke.

01- There are lots of benefits that are attached to research, it helps to identity the major cause of something. In the olden days stoke is not common, I didn’t hear anything called stroke as a teenager, as time goes by, stroke becomes a threat and very rampart. There need for us to know the major cause so that we can find a cure and lasting solution to it.

**Interviewer: Can you explain what you understand by biobanking?**

06-Biobanking as I understand it is more or less like money banking keeping valuable thing for future use. When we are talking about bio banking you know when you get to hematology they keep blood for blood transfusion. Likewise, they keep heart, kidney and brain. Let say for instance I have an accident had God forbid I may give up in a couple of hours, I can call my physician and ask him to pick up organs that are still functionary in my body and keep it for use for people that would need it, that is as much I understand about bio banking.

**Interviewer:** How **important is biobanking to medical breakthroughs?**

03- it is very important, there was a time I needed kidney UCH was on strike then, one of my kidneys was bad, it was not functioning well, so it important to keep kidney for kidney related issues.

02- It is important to keep organs for use so that anytime we need it, it will be at reach and life will not be wasted, for example blood, kidney so that anytime there is use for it they will just go there and get it for the person in need of it to save life.

**Interviewer: Belief/thought/opinion relating to biobanking**

06- There are different opinions relating to bio banking, like I said earlier that at the point of death I can call for the harvesting of my brain but my family will not agree because people believe everything belongs to God if you say you will die and God says No definitely the person will live our people believe even through the doctors says the person will die, they believe he can still live. So in a situation like this, the doctor involve must search the heart of the donor very well because without doing that litigation can occur and everything must be put in black and white, event through they know the person will die and the organ will not be useful for him when he is being laid to rest it must be put in black and white for any litigation that might arise from the family.

02- Belief of people is that this can cause harm to them and cut their life short.

**Interviewer: Awareness, understanding/perception of brain banking**

02- I have never heard of brain banking

06- In advanced countries, they do harvest brain and bank it, a lot of vital processes have to be undergone. If we come back to African, anything we want to bank except blood and kidney have to do with after death, like 80% after death, 20% when the person is almost gone or living for a very short period that is they know the person cannot survive. Like the heart now if they want to bank it they know the person cannot survive all the other part of the body is dead but the heart is still functioning can we harvest it and keep it for the person that we use it in the nearest future. If he oblige they can harvest it. The same thing applicable to the brain. In advanced countries. They have started harvesting brain. Like I read sometimes in 1993 of a very brilliant man. He had a crash and he had very little time to live. The man was a genius, they contracted him to donate his brain because all the other part of his body was condemned, they counseled him and he was ready to give the brain but the family objected to it, and the man died waste fully. The doctor then met with the family and told then his brain would have been useful if it has harvested. Here in Africa, I don’t think it will work out but in advanced countries they do it; that is what I can say.

01-I have heard of situation whereby human parts are taken and kept for use, but in Africa we only can harvest the kidney and maybe the liver, I don’t think we can harvest the brain. However, it can be possible but the possibility had to do with creating the awareness to the public because in the African setting they call it taboo how can your brain be removed perhaps they do it in ogboni fraternity, they cut the head of their deceased members so, if you donate your brain to be harvested it means something is wrong with you.

That is the major aspect that can lead to mobility of Africans to be involved in brain banking.

02-I have never heard of it, this is the first time.

**Interviewer: Awareness of any policy or law guiding biobanking**

06-I am not a lawyer but I know there are rules and regulations guiding bio banking

1. I know to a certain extent there are laws guiding, the blood, kidney, heart banking in the Nigeria setting, it has to pass through the normal protocol before these things can be done. Like I said earlier the patient is consent must be properly sought; if the patient consent is not properly sought and you go against his will, it will bounce physician. So, if these is no properly guidelines biobanking it will not be possible. That is why I said there are rules and regulations guiding it, if I am to be into law now, I would tell the section of the law to support it.

**Interviewer: Can you explain what you understand by precision medicine?**

02- I don’t know what to say.

03- Doctor’s prescription.

02- I don’t understand.

01- One on one dialogue between the doctor and the patient. That is my own understand.

- 06- precision medicine to me is like a doctor acting in the area of his specialization. For instance if a patient tells his doctors this what is wrong with me if he can handle it he handles it, if he can’t he refers him to another doctor that can. You know in stroke treatment, we have neurologist, we have cardiologist and so on. So, he if the doctor cannot handle the case, he will refer it to another doctor that specializes in the case for proper and genuine to be given so that the patient can get well and be healthy on time.

**Interviewer: Benefit of precision medicine**

06-The benefit now is that, for instance a stroke patient through the CT scan it was discovered that high BP have caused a damaged, it is through this they will know the actual drugs to prescribe to the person but without precision medicine they will just be using headache drug to cure stomach pain. So, there are lots of benefits to it.

**Interviewer: Demerit of precision medicine**

06- There are disadvantages but the benefits are more than the disadvantages. If there are many patients to be attended to, it would take a lot of time; this is one of the disadvantages. It takes time because all the patient will be attended.

**Interviewer: can it be applied to stroke medicine?**

- 06- Yes it can be applied to stroke

------ Awareness of any policy or law guiding precision medicine

- 06- I am not a lawyer, but to every good medication to every good program there is always a law guiding each step of the treatment being received.

**Interviewer: What do you understand by brain donation for research purpose?**

06- I have said it before that in African we are not there yet but in advanced countries they are already there. Let me say like these twins that fused together if they want to separated them they will do it. In advanced countries, they are there already, they are donating brain but here, we have not got there yet because of the spiritual and traditional belief we have, for instance, if someone should ask for your brain what will you say, even though if the person is dying the family will object to it. I don’t know if it is in your question that is there any law guiding brain donation in Nigeria, if it is in your question I would say no, nobody can make such law for us in Nigeria but if it is in advanced counties I will say yes.

**Interviewer: cultural, social and religious belief on donating brain for research purpose**

- 06- like stroke now, people would say stroke is an attack, stroke is not an attack or whatever they call it. Stroke is a disease of the brain because all the wire work that starts from the toe are all connected to the brain; there are three compartments in the brain; the medulla oblongata, the cerebrum and the cerebellum, all these three thing are in charge of all the activities of the body. Cerebrum is the father; that has to do with the thinking the thinking faculty you know, all these stuff. So, if you affected with stroke, if it is the left side, the left side will be totally paralyze, if it is the right, the right be totally paralyze meaning the veins and tissues that connect to the brain and dead for that period. It means the brain is not giving normal sign, it is not communicating properly

**Interviewer: What factors promote brain donation?**

06- Through proper orientation, we have to be ambassadors to promote, we have to orientate and educate people. I am a stroke survivor now, I am a stroke ambassador, like she said, she is a stroke ambassador, also mama and anybody that has stroke.

Educate people the Imams, the pastors, Baales and Obas. Let them know that the old people that are about to die around them in the community their brain can be useful, it can be harvested and keep for use. With time they will be the one to advice and encourage people to donate their brain.

**Interviewer: Share with us your opinion and thoughts about blood sample donation for stroke genetic research.**

02- My opinion is that it is good to donate blood because it is useful to people in the society. Also blood donation for testing is very good; it will help to know what exactly is wrong with the patient if investigations are carried on the blood sample collected from the patient; when I was sick, my sample was taken more than five times for investigation to know what actually caused my stroke. It is good to donate blood so that research can be done on it to know the cause of one’s problem. If someone has HIV and he refuses to donate blood for testing how would they know he/she has HIV. It is good to donate blood

**Interviewer: Willingness to be involved in such research**

- 01- As a person being affected by stroke I know what it means to donate blood. I know what I suffered but through the help of the medical team I was able to get out of it. So, if it is blood that is need because of the experience I had I will be willing to donate it.

**Interviewer: What do you see as the barrier(s) that could hinder your donation of blood sample for stroke genetic research**:

06- There should not be any barrier but benefits.

**Interviewer: What do you perceive as benefit(s) of giving blood sample for stroke genetic research that could promote your willingness to donate:**

There are lots of benefits in donating blood for research, if not for my survival, what about the survival and quick recovery of other people suffering from stroke it very important. Stroke affect a lot of things in the body, and it has many effects on the person. It is through the blood the will know the amount of water content, sugar content and salt level in the blood. So, without donating the blood they would not be able to do proper research to get result.

**Interviewer: What can you say about your family member or other members of the community willingness to give blood sample for stroke genetic research?**

06-I have my life to live, and when you are 18 years you can stand on your own, so a 40 years and above men to be waiting for family before taking a decision that would save life of others, there is no barrier that could hinder me, or stop me from donating sample for stroke.

**Interviewer: What could be done to make you and more people give blood sample for research: mass media, husband consent, family consent, donors group, peers**?

06 –I stand on my own, but when it comes to group of people there will be mixed reactions, some may be willing while others may not. For this to work we have to orientate people on the major benefit that it is used to save life; so with proper understanding and knowledge they will give their consent.

**Interviewer: Awareness of national guideline recommendations**

06- There is both local, national and international level. At the national I know there is a bill that is passed at the senate on this thing. It is only the brain that is a no go area for the national and local. I know there is a law perfectly guiding blood donation.

**Interviewer: Tell us what you know about informed consent?**

01- It is about taking the consent of an individual that have suffered stroke, with that you have a mindset that you are going to sensitize people on how this thing can be prevented

04- I don’t know anything about it

06- Informed consent is a form that is placed in front of you to sign and date that during the research if there is anything that happens to you or the sample you give there won’t be any problem or litigation that would arise from it, that is informed consent. For instance when I wanted to do CT scan to know the actual thing that caused the stroke, I was unable to write, my wife have to sign the consent for me before they could perform the experiment on me. So it brings a merger between the physician or researcher and the donor so that there won’t be any litigation. That is it.

**Interviewer: Type of informed consent preferred**

06- I will prefer the written consent. On the issue of long or short, there are some researches you thinK will take short period of time, if it takes a longer period of time, it takes a longer period what will you tell the donor and the donor want to have the result on time. As a donor I will prefer the written consent and broad type

01- I prefer the written consent, but the consent you took this morning you didn’t explain to us if it is for a long period or short

02- Written consent, In which they will inform us as the research is going

04- I prefer the oral consent

**Interviewer: What is your opinion on storage of blood sample and blood fractions**

02- Storing of blood sample is good if it is stored properly in a safe place.

06- My opinion is straight forward, it is to safeguard future occurrences it is good.

**Interviewer: Tell us what you know about sharing of data, blood/blood fractions, brain images (CT scan/MRI) as well as brain tissue samples**

06- It is very good, all the research that have seen done, the essence of it is to share the result; if the result is not shared it is exercise in futility, your knowledge that have been put in line will be wasted. It is accurate record and data collected on research or experiment that will be collated and if maybe shared with other researchers

**Interviewer: Share with us your thoughts about return of individual research results and incidental findings**

06- I will like mine to be save in a CD and send to me

01 – It might take time if you are to send it. It can be send through SMS; one can check it at anytime

02- My opinion is that, you can call us together like it and talk to us about it

01-Through text message e-mail

04- Through phone call or you call invite us to come

05- I know it is God that gives result, let us leave everything to God. You can tell me on phone

**Interviewer: Explain your understanding of Biorights**

01-The right of an individual live the lifestyle that will help or connect with others, and be able to access information. Bio-rights make people accessible to information that concern them

06- I have the right to know the result of the sample I donated for research, and I have the right to collect the result but the right does not go beyond the consent. I don’t have the right to go and be disturbing the researchers, through the consent I would have been informed how long the result will take if it will be six months, I have the right to ask after six months, and if the period is going to be extend beyond the time of the consent, I have the right to be informed by the researcher about the elongation of time.

**Interviewer: What is your opinion about governance and regulation of biobanking?**

06- to every citizen there must be a governance but we are not enjoying good governance in Nigeria. Attitudes toward bio banking is bad. Let me put it the way in advance countries, Let me say London to be precise, as a stroke patient you have entitlement to free drugs and free treatment but have in Nigeria there is nothing like that you have to buy your drugs yourself, they don’t care about the patients. So now, let us bring it to research, they are not concerned about it

02 – Our government have to show concern about biobanking because it a way of saving the life of the people they govern

**Interviewer: What suggestions do you have that can help raise awareness and improve attitude towards blood sample or brain donation for research and encourage people to adopt the practice?**

06- I have said it earlier, firstly there must be properly orientation. Then the researcher must have a very accessible place to keep the materials, body parts that have been donated to prevent it from damaging because if it damages it will be useless. There must be proper banking for the organs donated.

01- I think before a country can come up with biobanking they must have prepared for it very well. On the other hand, there must be proper awareness through the media to be aware of the development, and it should not be limited to the hospital alone, it has to be given a wider network, go to the media to give awareness about it, where to go to donate and how you can surrender yourself for cross examination where to go to get needed information.

**Interviewer: Any other major concern or recommendation on use of blood or brain tissue for research in Nigeria**

06- The researchers must protect the samples. We know that blood sample needs to be refrigerated and power supply here is poor, what can we do? We need to get a stand by generating power set just protect the specimen, his interest in the research and lastly the interest of the donor.

Then about the record, the gene we are studying today, the work started in the 17th century by Charles Darwins, if he didn’t keep a proper record of how he did it then, there is nothing we would be talking about in biology study. So, they should have a proper record for the benefits of future usage.
