# Supplementary material for: Research participants’ perception of ethical issues in stroke genomics and neurobiobanking research in Africa
Source: PLoS One. 2025 May 6;20(5):e0292906. doi: 10.1371/journal.pone.0292906 (PMC12054916; doi:10.1371/journal.pone.0292906)
Supplement: S3 File — (ZIP) [file pone.0292906.s003.zip › Files for PLOS ONE - updated March 2025/IB_Stroke Free Controls_FGD.docx]

**Interviewer: Tell us what you know about genetic research.**

03- What I understand by genetic research is carrying out a research on parts of the body that look alike. For instance, for those that are short in height. If a family has a baby, looking out for what is in the mother that is associated with the baby is what we call genetic research.

01 – What I can say about it is that maybe there is a disease that usually occur in a family, and the same disease shown in their fourth child. They can carry out research to see if the disease has happened in their generation either to the father or grandfather. They can do research to find out if the disease is in the gene of that child. That is what I know about genetic, to find out if a disease is in the gene.

02- As I understand it, may be a mark is found on the body of a new born baby, elders in the family now observe that their great-grandfather had such mark. They may want to investigate the cause of it may be to stop it re-occurrence or for it to continue.

06- What I understand about it is that when I was much younger then in our village, there was a family that used to have still-birth every year. They may want to find the cause. In fact, I wished I was a doctor so that I can help find out the reason and stop the cause.

**Interviewer: Have we all heard about genetic?**

04- I never heard of it

05- I never heard of it

**Interviewer: Who has heard about it?**

03- Through the aforementioned examples I know there will be genetic research to know the reason for some events. For example, when we were told that there is a mark on a baby which may cause still birth, they will make finding probably the cause is from their linage. It will prompt genetic research in such family.

**Interviewer: In this gathering, do we have anyone who has participated or know someone who had participated in previous genetic research?**

No.

**Interviewer: Did you know or have you heard about stroke genetic research?**

01- What I can say about genetic research in stroke is that I went for a seminar sometimes ago they told us about genotype that if AS and AS marry it is possible, they have a SS child that may likely suffer from an ailment which may likely be stroke. Also, I have someone who is suffering from rheumatism and the child suffers as well. The child always takes injection on it from time to time. In my view, one needs to make research on it because it is possible that the person’s grandchild will have it and it will continue like, and rheumatism can lead to stroke.

02- What I can say it that is that sometimes ago a group came to our community to educate us on eating habit and the damage it could cause to our body and that it can lead to stroke. They also told us that when we notice blurred vision, weakness of the lower limp or general weakness of the body, this may be signs of stroke. That is what I have to say.

**Interviewer: What are the roles or benefits of genetic research in medicine?**

02- I believe it has benefit, when a research is carried out on stroke, and the type of drug to be used, that can be applied to arms and legs is known and given to the patient, it is through research that this can be achieved. Or through body check-up, the drug that whoever is showing symptoms of stroke can use to have immediate care to prevent it from happening. So, there are lot of benefits.

06- The benefit I can say of genetic research is that it will help to put an end to the occurrence of stroke. Like now there is vaccine for children to immune them unforeseen ailments. I believe through genetic research they can get a vaccine for stroke that will put an end to its occurrence.

01- The advantage that is that it will reduce doctors’ burden. They will know what exactly to do to prevent stroke instead of having many trials.

04- The advantage is that through stroke genetic research people that do not know they that they have stroke will know. It will prevent those who might be at risk of having stroke in future. Also, this study will put an end to do not eat this or that or sometimes overdose can become a problem for a person with who is sick so it is better that they should do research on it so as to live a long life.

03- In addition, research helps to know the reason why some things are happening and the way out. If the doctors diagnose someone to have a stroke, they will be able to enlighten the public, may be through social media on the preventive measure to take to control the disease.

**Interviewer: What can you say about bio-banking?**

06- Bio-banking? I have never heard of it. What I heard when I was young is that when surgery is perform on people they can remove someone else’s veins and use it stitch the spot where the surgery was done.

01- The way I understand bio-banking is that it is research that will assist us to bio-bank. We know that a lot of medical doctors have passed on and the research they did then is still assisting the upcoming doctors to do well. If they had not done anything there will be nothing for the upcoming doctors to rely on.

02- If thorough research could be carried out in a way that if one has the opportunity to bio-bank, it will not be as if the person is being forced. I have never seen it in real life but in movies. If someone is sick and in need of an organ and another person is dead but there is something that will still be useful in the dead person’s body such organ can be taken for the sick person’s survival.

01- Donating blood is as good as keeping something alive. I believe it is part of bio-banking because there is a saying that to says ‘blood is life’. Blood banking is part of bio-banking.

**Interviewer: Source of the Information**

01- There was a time my brother was sick and we brought him to here, a lady was running up and down looking for donor who will donate blood for her sick mother. All the available blood and donor did not match the woman’s blood. People that donate blood are trying they are saving life. I learnt about it here in UCH.

**Interviewer: How does bio-banking organs operate or preserved?**

02- Is not that I have seen it in Nigeria, but in one of the Korean movies I watched. I can describe where they kept the organs, like kidney, brain and the likes as a very secure place meant for professionals only. The place is like a cool-room. They have a specific clothe and shoe they wear and they cover their body well before they can access the place where live organs are kept. The organs are well packaged so that it will not be exposed and contaminated

04-My experience with bio-banking was with blood. My boss and I came to donate blood here in UCH. When we got there, they first clean our hand with cotton wool and spirit, later they brought bag for the blood and asked us to lie down, later they collected our blood into one sachet and preserve it in a freezer. I also have the privilege to donate for someone at Jericho some years back.

06- Everything they have said is true about blood donation which they must preserve in a refrigerator till when there will be need for it

**Interviewer: How important is bio-banking to medical breakthrough?**

02- It is very important to medical breakthrough in the sense that, maybe someone is in dire need of kidney transplant and since it has been kept somewhere, what they need do is to go to where it has been kept to take it. The only thing they need to do is to know the type that will match the person. This will make their work easier, not to be scrambling around for donor.

03- It reduces death rate.

04- Doctors did a great deal of help in setting up research to reduce untimely death. For instance, if someone has low blood volume or loss of blood through accident and he does not get help the person may die.

01-The advantage I noticed is that it gives improvement to medical profession and our nation in the nearest future. If late Chief OB didn’t have plan for a good future for us in this nation, UCH wouldn’t have been here for us and not everyone of us can afford to go for treatment abroad. If someone is sick and they have taken the person to various hospitals, if at the end of the day such person is brought to UCH he/she will be taken care of and will be well, provided if his/her condition is not spiritual or the person days on earth is up. Therefore, bio-banking is good and will help the future of our nation.

**Interviewer: What are your beliefs, thought or opinion in respect to bio-banking?**

02- My belief is that when organs are biobank it will save someone’s life when such person is in need of it.

07- My belief about bio-banking is that the sample kept will be useful in the future. If something bad happened to someone, the one that they have kept will be useful.

03- My belief is that it will always be useful to others. For example, if anyone is in need of kidney or blood they will have to take from the ones they banked. Also, if someone’s blood is infected and needed to be changed, it is the one that has been kept that will be rely upon.

01-Bio-banking will help those coming behind (like doctors) to have a way out when they are at a crossroad because what they need is available as their predecessors have helped them to solve their problems and gave them hope for tomorrow.

**Interviewer: What is your knowledge or understanding about brain banking?**

01- The idea of keeping the brain is different from keeping blood because Brain is not like blood. Blood can be stored but I don’t think that of brain can be possible. I have never seen a human brain before. We can see blood when someone donated it. In my view, the only way one can donate brain is through research. When someone work on a research or project, another person can build on the work of such fellow and use his work as a reference material, that is how we can bank brain.

02- As I said earlier, in the Korean film I watched, a man had an accident, when he got home after he was discharged from the hospital, his brain was not as active as he was before the accident. He was taken back to the hospital for better treatment and to have his brain checked. He went through surgery. They had to operate the brain, remove it and fix another one. The same way compatibility test is being done before they use blood was how compatibility test is carried out before they know the one to replace. After he came out of coma they started teaching him as if they are teaching a child till his brain came back to life.

04- What you are asking us made me to remember Dr. T. S. Dr. T.S said that if he died he should be placed in a museum where his brain would be used for research purposes. A doctor at the University of Ibadan also requested that his brain too kept in UI. Now that you asked make me remember that brain can still be useful after death. Brain banking is very good, especially if the owner of the brain was not fulfilled before he died and he is a very brilliant person they can keep the brain and use it for development of this country and human existence.

**Interviewer: Do you know if there is any law or regulation guiding bio-banking.**

01- I believe there is law. Whoever does not practise medicine must not collect blood and keep in his/her house for use. Even, petroleum, that does not involve life, there are laws guiding it storage because of the havoc it could cause. So just anybody cannot keep any part of the body, I believe there is law.

02- I believe there is law, in as much as there is law guiding medical professions to set up a hospital, there is also a law for bio-banking.

03- There is a law that requires them to inspect test blood for compatibility and properly screened before they transfuse it.

**Interviewer: Can you explain what is called precision medicine?**

01- Precision medicine is when an individual has a doctor to cater for him/her. Those who are interested in getting this done can visit the hospital so as not to be treated the same way other patients are treated. Those at the helm of affair in the country or the rich have personal doctors. They may decide not to be admitted in a general ward but in their house. I watched a TV program on BCOS, and the doctor they interviewed said he was a former president personal doctor. So, I think precision medicine is when someone has his/her own personal doctor that takes care of him/her whenever he/she is sick just like I have only one mechanic that repairs my car.

02- To my understanding maybe a patient that does not like coming to the hospital now have a personal doctor that goes to his/her house to treat him/her. Or, maybe as we have many doctors here in your garden, the one that have a private hospital among them. Such hospital may be closer to people more than the general hospital. With time, people will be patronising the hospital for one-on-one appointment. The doctor attends to a patient in a day.

03- It is when a doctor specialises in the treatment of a disease. He does not treat another disease only. Those that have such a disease will be asked to be treated by the doctor.

**Interviewer: What are the advantages and disadvantages of precision medicine?**

06- The benefit is that a doctor will treat the disease he/she knows about, unlike the traditional herbs that can cure several diseases with only one herb. That is the benefit therein.

04- The benefit is that, atimes, there is something else that should not be obvious to many people that only the doctor treating that patient will know about it. This will help to keep the secret from too many people.

**Interviewer: Disadvantages of precision medicine**

03- If the doctor is the only specialist in that field, there may be many patients waiting on him for treatment which may in turn cause over-burden of job. Individual medicine wastes time.

01- The assumption that there is possibility that if a doctor only treats a patient for a particular sickness, and if another sickness which the doctor does not know the treatment surfaces in the patient, the doctor may want to treat it as well. This can lead to more complications for the patient.

02- Precision medicine is time consuming; a doctor cannot single-handedly care for a patient. He/she will consult other doctors or senior colleagues. During the consultation, it is possible the condition of the patient get worse.

**Interviewer: Does individual medical care important in Africa.**

01- It's Important if we want to make our country better. There was a time we brought someone to UCH for treatment, before they commence treatment, many tests were carried out, but we felt they are wasting our time. The idea is if it were a private hospital they might start with injection. The benefits it will bring will improve our country, and we will be able to compete side by side with the rest of the world. If we are patient enough, the future of our country will be great.

**Interviewer: Can precision medicine be applied to stroke?**

06- Precision medicine is useful and important in Africa. In this hospital (UCH) there are different specialties. When we first arrive they will direct us to where we will go, outpatient clinic. After necessary questioning, the person will then be referred to the clinic he/she belongs to. Precision medicine is very important and can be used in treating stroke.

01- It can be used for the treatment stroke. Precision medicine will help us to know the cause of the stroke. If stroke catches a person, not only the arm and legs that will paralyse, it will damage the body, ear and can upset the brain. If the benefits in precision medicine is not utilised, if the arm and leg are healed and the brain is down, will the patient move on with ‘dumped brain’?

02- There is proverb in Yoruba that says ‘The person that knows better should be allowed to dictate’. The reason for that proverb is that precision medicine can be used to treat stroke because it means that the doctor that knows about it will be allowed to handle it. Taking a general survey in this hospital right now, you will notice that a doctor in Orthopaedic departments cannot work in the labour room. It will be that the doctor that knows about stroke is the one stroke patient will be referred to.

04- It is possible according to my experience and what I heard. God should allow us to meet the right people, if you meet the right people that know how to take care of stroke, it will be better, very beneficial

**Interviewer: What is your Belief, thought or opinion to precision medicine?**

01- My belief is that there won’t be ‘misappropriation or misapplication of treatment’. When a patient goes to the doctor that specialises on his/her ailment, his/her treatment will be fast and recovery as well.

06- My believe in precision medicine is that it is good. A doctor will not manage different conditions.

07- My belief is that precision medicine will let us know where to go for help when we need medical attention.

**Interviewer: Are you aware of any policy or law guiding precision medicine?**

02- There are policies guiding precision medicine. Before someone can say he is a medical doctor and he want to care for patient or start a private hospital, he must have studied to a certain level. It has nothing to do with being rich or influential. The person must study medicine and qualify as a medical doctor.

**Interviewer: What do you know about brain donation for research?**

06- Initially, I thought that when one dies the brain also dies. I don’t think the brain will be useful again. Talking about brain donation or banking, I have never heard of it.

03- What I think brain donation for research is all about is how brain works, functions and the various ways in which we use brain.

**Interviewer: How difficult it is to donate brain for research?**

02- In our country, it will be difficult. Reason being that in western countries they understand the benefits of brain donation. That is why it will be easy for them to walk into the hospital and say they want to donation after death but here in Nigeria we are not enlightened to that extent. I got the knowledge from the Korean movies I watched. This is the first time most of us seated here will hear about it. So, it will be a bit difficult in Nigeria. If they are to send people out to talk to people about brain donation, if twenty people should go out, just five will return unbeaten, the remaining fifteen will be beaten blue and black by the people in the community because this will be very strange to them.

06- It will be difficult in the sense that brain is one, it cannot be divided into two, unlike kidney which is two where if one is removed the other one can still function very well. How can it be removed if not that the person is dead?

04- I talked about some doctors the other time. Brain donation is going to be a bit difficult because we blacks believe that when someone dies it is possible for such a person to come back, but if the person’s brain has been removed there is every possibility that the person will not come back as human. For instance a friend of ours died here in UCH, when the corpse was brought back home they noticed that the head was bandaged. People started to ask questions. When they examined very well they noticed the head had been tampered with; surgery has been done on it. The family wanted to raise alarm but they later let it go. It was obvious they removed something from the head without informing the family.

01- It will be very difficult to do that in Africa because we do not have the knowledge yet. If we had the knowledge according to previous speaker (no 2), when someone is old to a stage, he/she should be able to donate the brain or other part of the body for the future of the country. For example, some people willingly donate blood without thinking of who will use it. They just donate willingly. In Africa some fathers are so stingy. A story was told of a man who swallowed his car keys, when he was about to die. So, a man who does not want to leave anything for his children to inherit, will he willingly donate his brain after death? It is going to be a bit difficult in this country,

**Interviewer: What are the benefits of donating brain?**

02- It is going to be beneficial if there is an enlightenment programme in which the people are educated on the benefits of brain donation. It is going to be beneficial to the nation if the aged can donate their brains after death provided they have been earlier enlightened on the benefit it will have on younger generation. It is even possible that the person that will benefit from it will come from the donor’s family. So, it is going to be beneficial if we have the knowledge and are exposed to the benefits.

01 – The benefit I perceived is that insanity will be reduced in our community. Those that have been insane for long would be treated, and they will be able to change their brain. Also, in the categories of learning where some are referred to as slow learners, while some are fast leaners. If the brain of our scientists that are very good are taken and kept our country will be developed the more because they will not be buried with their brain.

**Interviewer: Can you willingly donate your brain for research?**

05- In my opinion, if we have achieved in life and have grown old to a certain age, let’s say like 70years and all our children are doing well we can donate our brain. If we are above 70years there is nothing more we want to do.

01- I cannot donate my brain because our country is not good to that extent. It is the wealthy that will buy it except they will use if for my family members or give it away for free. If we donate now and our family need brain in the future they will ask us to bring money before we can get what we donated for free. If they will use if for my family or they make it free for people I can donate like TB now the treatment is free and an organization is in charge of that, if I have money today I can support them if it is going to be for free I can donate.

02- I can donate if God gives me the grace of long life, because brain donation is definitely after death. Then, we will sign an agreement and also supported it with a little curse on anyone that changes anything in the agreement. The reason why I said this is that in this country people are so cruel to the extent that if people voluntarily donate something they will sell it with a huge amount of money. The person that willingly donated that thing and his family will not gain from it. That is the reason why I cannot donate my brain.

03- Since it is for the benefit of other people I can donate if God gives me the grace and I live well and age well.

04- It is possible to donate but just like the others said it has to be on record that it was donated by someone, as we come here everyone wants to have a good name. Like those fathers I mentioned earlier their names will always be mentioned that they contributed to the development of the nation.

06- I cannot donate because for someone to say he wants to donate it means the person is ready to die.

07- I can't, and I won't donate because Nigeria has become something else.

**Interviewer: Roles of cultural, social and religious belief on brain donation for research purpose.**

02- Our religion has a lot to do with brain donation. Let me talk about Christianity, there are some Christian denomination that will never receive blood transfusion no matter how it may be, they will rather die than to accept it. I know someone that has died in that manner. Similarly, Muslims cannot leave their brains. They will rather quote sharia law for you and will be saying ‘*awusubilahi’*, which means ‘God forbids’

04- The only religion that can easily donate their brains is the people in occult group. That is the only sect that donates parts of the body at death, so removing the head is not a big deal to them. So someone who has allowed them to cut his head does not have a say over his brain.

01- We understand religion from different perspectives. Some people are too zealous with religion to the extent that they cannot take drug; like paracetamol. They prefer to pray whenever they are ill. But if we understand health, there is nothing wrong in doing things according the laid down rules but others have given more faith than it should, depending on how religion is embraced and as people understand religion.

**Interviewer: What are the factors that can inhibit brain donation?**

01- When number six was talking the other time he said he can‘t donate brain, anyone that donates brain is ready to die. If one donates blood there will still be blood in his body and there are things he can use to replenish, but brain that is the end.

**Interviewer: The researcher explains that brain donation is only possible after death.**

01- When a person is dead. When someone dies the brain too will die. Will they be carrying dead brain around?

02- In my opinion, the factor that can inhibit brain donation is lack of knowledge about it. People that do not have the knowledge of brain donation at a younger age cannot willingly donate it at old age. If the person has the knowledge and he willingly donate, they can let him sign or record his voice about his willingness. Once there is evidence that the person willingly do it, the family will not have a say, but lack of knowledge about it will inhibit willingness to donate.

04- What I observed that will make it not to inhibit is if there is seminar on it frequently, may be on the television, to announce that if there is anyone that can donate organs, such person’s name will not be forgotten. But taking organs of the dead without the knowledge of their family is illegal, if the family is a strong family they will sue the hospital.

**Interviewer: Do you think peer values and parental influence can inhibit brain donation?**

01- As we are here now let say you give us form to sign and I agree to donate my brain without the consent of my wife and children, if after death you came to remove my brain there will be uproar. They will tell you they do not know about it. So there must be an agreement with the family. Wife and children must be involved, except if the person is an occult member. They do cut off their heads when they die, and they know why they subject themselves to such things.

**Interviewer: Can you share with us your opinion and thoughts about blood sample donation for stroke genetic research.**

01- I can only donate blood if the sample they will take will not be more than a syringe. If it will be more than that like a pint of blood, I cannot donate.

02- Some years back, someone came to our church. They educated us and took our blood samples. God used them to save many lives because many people had high BP and diabetes and they didn’t know. They called these people and enlightened them on what to eat and drink, also they prescribed the medication they were to take. They didn’t take much blood from us, I think like four small bottles, which is not too much to donate.

**Interviewer: Can you willingly donate for research?**

03- I can donate blood for research purposes

04 - I can donate blood for research purposes

05- I can donate blood for research purposes

06- I can donate blood for research purposes

07- I can donate blood for research purposes

**Interviewer: What can be barriers that can hinder you from donating blood sample for stroke genetic research?**

02- The only thing that can hinder my donation is if I have an ailment and they checked found that I do not have enough blood.

06-The only thing that can hinder my donate is that I know the sickness I am nursing right now in my body, so it is possible that my blood may not be useful.

03- The only reason I should not give blood is If the group carrying out the research is not a genuine group which I know very well.

04- Nothing can stop me from donating blood

05- If am not feeling fine

06- If I don’t have enough blood in my body

**Interviewer: What are the benefits of donating blood sample to stroke genetic research?**

01-The benefit is that it will make someone to know on time if he/she will have the disease and will be able to prevent it.

02- It will help one to know the state one’s health, whether or not they have it and how to take it.

03- The advantage is that it will let them know how to get rid of it and help to prevent the it.

**Interviewer: What can you say about your family member or other member of the community’s willingness to give blood sample for stroke genetic research?**

04- What I can say is that if there has been a seminar and have informed them that the programme is from the government. All the people want peace, they always agree to participate.

01 Everyman has authority over his home. As I am here now and already have the orientation, it will be easy for me to convince my family members to donate blood than for a strange or health worker they do not know.

02- In the case of our church, w hen those people came, at first people got up and left. They said they cannot donate their blood but when the person that invited them explain the benefits, all the people that left started coming back, they all donate and people benefitted from it. With adequate information, it will be convenience for people to come with me.

**Interviewer: What could be done to make you and more people give blood for research?**

05- This type of programme should be organized from time to time. We are a set now, another set should be invited next time, and we will go home and tell our people about it and its benefits. From there, more people will know about it.

06- The only thing that can be done is to broadcast it on radio and television so people can hear and learn more about it.

07- Public awareness program, announcement on radio and television so that more people will hear about it.

**Interviewer: Can you explain the current practice about blood sample donation for research?**

06- If someone willingly wants to donate blood, they will test the person to be sure if he has enough blood. I heard of a man that went to Oluyoro hospital to donate blood for someone. When he got there and they tested him they admitted him immediately because it was confirmed that he too needed blood. So they will first test the person that want to donate to know if his/her blood is enough and useful as well.

**Interviewer: Do you know the current practice about blood sample donation for stroke genetic research?**

01-I don't know

02- I don't know anything about it

03- I don't know

04- I don't know

06- I don’t know

**Interviewer: Are you aware of any system currently in place at this hospital for enhancing blood sample donation for genetic research?**

01-I don't know

02 - The group I mentioned earlier that came to my church came from this hospital. They talked to us about stroke and how to prevent it, and they also took sample from us.

03- I don't know about you

04 - Then we know, but not now

05- I don't know

06- No, I don't know

07- I don't know

**Interviewer: In your estimation, what proportion of patients is aware of blood sample donation?.**

01 - They are not many, let us say 20%

02- They are few

03- I think most patient know they will do test when they come to the hospital, so I will say majority know about blood sample donation

04 - Not enough patients know about it

05- Very few

06- small

07 - They won't be many

**Interviewer: Do you know law or guideline recommendations either here or in overseas about blood donation for genetic research?**

04- I have been able to donate blood, and the only thing I saw there was that they came in to check our genotype and blood group, it was after that they started taking our blood.

**Interviewer: What do you know about informed consent in participating in a research study?**

03- Before someone can give his/her consent to participate in a research he/she must have the knowledge of what the research is all about. If he/she is satisfied then he/she will give his/her consent.

06- Before a person gives consent, he/she must know about what he/she wants to get into and the benefits he/she will derive from it, the important of the research, what he/she is going to gain from it before he/she can agree or disagree.

04- What I do know about informed consent is that the person who gives consent must be someone who has knowledge of what he/she is participating in.

**Interviewer: Which type of informed consent do you preferred?**

01-I prefer the first one, broad. This is because it makes people aware of the disease in his/her body, as we know that there is no one without sickness so they can use the sample for various researches.

02- I agree with the broad, the one they can use blood for research because it will allow someone to know the problem he/she has in his/her body through various researches it can be used for.

03- Broad

04- Broad, we have to agree before I will let them take my sample, they will compensate me for the sample donated. Only God knows how everyone will end his/her live.

05- Broad

06- Consent given by number three is what I want. I have come to the clinic where they take my blood and I never set my eyes on the result. I will like them to call me from time to time to inform me. I have donated sample for research before till today no result from them, and they took 9 bottles of blood from me no result.

07- I prefer number one, the broad type

**Interviewer: Who are the people you need to inform before you before participate?**

02- Before I can participate in such research, I will tell my husband and also inform my mother. I don’t hid things from her.

01- I will tell my parents

03- Family and friends close to me

04- Wife and children

05- I will tell my children and my family

06- My children and my wife

07 - My husband and children

**Interviewer: Data use in the incident of death and why**

02- If I understand your question very well before they can take sample from anyone they have to test the person and be sure the person is healthy and in good condition to donate blood, if the person is not healthy they will not take the sample from him because they will not want to risk the life of the person.

04- If they collect blood from someone and the person is dead, they should continue with the sample.

03 - They should continue using it

02- What one has donate from when one was young, they should continue using it

01 - They should continue using it

07 - They should continue using it

06 - They should continue using it

05 - They should continue using it

**Interviewer: What are the Support for blood donation in our society?**

04- It is good

03- The support I expect is that anyone that donate sample should be given something to replenish so that he can recover.

02- You should support the person who donates blood because the patient will not get up as soon as he/she donates blood, he/she will need to rest for some minutes. So I believe that there should be something to eat or drink that will give energy after donation.

**Interviewer: What is your opinion on storage of blood sample and blood fractions?**

02- My idea about storing blood is that it is better to keep blood for use, because if we find someone who needs blood then it will be easy to find blood that matches such person, otherwise the person might die.

01-My opinion is that as the day passes knowledge are increasing, I think keeping blood on a regular basis will help doctors whenever there is need to use blood and make their easy.

06-it is good to store blood because of emergency. It will not be that it is when they are in need of it that they will go about looking for it. If you go to blood bank now you will see people waiting on donors that will donate blood for them but had it been that the blood has been stored it will be easy they will not have to suffer it.

**Interviewer: Tell us what you know about sharing of data, blood/blood fractions, brain images as well as brain tissue samples.**

01- You can see that technology makes communication easy. For example, you called us here, and as we came this morning we have given you our Whatsapp number, so the organization that is in charge of the program can either call us or send message to us.

02- The way I understand your question, we learn a lot through Whatsapp. I t is better and easy to send message through it. There is nothing bad in sending message to us through WhatsApp. If you even want to publish our photographs there is no big deal, once you have let us know before you do it.

**Interviewer: The Researcher should ask the questions further**

02-What I can say about sharing of data is that there is nothing bad in that, since I willingly participate in the research and willingly donate whatever they want to do with the data or sample they can do, nothing again.

01- In as much as we want to help with the medical and health care system there is nothing wrong with that, they can do whatever they want to do.

**Interviewer: Commercial or non-commercial use of stored data, blood/blood fractions, brain images and brain tissues.**

01-I am not in support of that

03- If a person has donated blood there is no need to start asking how the blood will be used, we should just take our eyes off it.

**Interviewer: Share with us your thoughts about return of individual research results and incidental findings.**

01-I have said it before that they can use our numbers since you already have our numbers if you want to return individual research result you can call the person on the phone or send it through WhatsApp.

02- You can call the person through his/her mobile phone.

06-You can call the person or send a text message

**Interviewer: What are your thoughts on returning individual research results and incidental findings?**

01-If someone knows that he/she has an ailment he/she will almost die before he/she is cured. So, for that category of people, we should not inform them of any incidental findings so that they can have long life.

**Interviewer: What are the ethical, legal and social issues relating to returning individual research results and incidental findings generated by genetic research?**

01-There are people even though they return their research result and incidental findings, they will not seek help, they will tell you they don’t use drug, that is a social issue it has a lot to do with returning individual research result.

**Interviewer: How much control should individuals have regarding how their biological specimen will be used in research?**

01-The control an individual has does not pass what he/she writes down or what we both agree on. If you use the sample for another thing we won’t know since we are not there. The control we have is what we write and agree on.

02- What I understand is that it is what I have that I have power over but what has been taken from me I do not have power over it again. In our country here the donor do not have power over what he/she donated. If it were to be oversees, people have power to investigate what they donated for research and it must be used for what it is donated for but here we do not have that right to question or query the researcher.

**Interviewer: What right should individual who provide their specimens have**

02- When there is a agreement that is binding and the purpose for which the organ was donated that was what it was used for, I don’t think there is need to take it up legally but if there is breach of agreement the individual can involve the law.

**Interviewer: Is there any profit for the person that donate specimen for research from research proceeds?**

01-the gain I think the person would have is that the organizations that came to ask for the organ are to be honest and use it for that purpose but if they do not use it but the donor is updated on the process I think that will be rewarding enough.

02-The organization I told you that came to my church when we did not hear from them after 3 weeks, we challenged the person that invited them, but later they started calling people that have issues and told them how to go about seeking treatment. Had it been that those people did not donate their sample they would not know that they have problems, maybe by the time they would know it would have gone beyond treatment, I believe that is a profit.

03-The person will know in case he has any disease, and whatever they offer him after donating specimen is part of his profits.

04-The person may need help later in the future, the organization should be able to help him/her.

**Interviewer: What is your opinion about governance and regulation of bio-banking?**

01-My thought is that our government should form a board that will be in charge of bio-banking, but most of the time they use these materials anyhow and it does not go round. Things that are meant for the masses do not get to them it disappears in transit. Our government should work on this.

02- The government needs to be serious because if that is the case, there should be real plan for donors. Do you know that the person who left his/her brain has died and that is the reason why he/she donates his/her brain? What would be the benefit of your family on the fact that a father or mother left a brain for human use, and if such a thing would happen it should have government support, that is the grace that should be available.

**Interviewer: Is there need for ethical committee approval on future use of stored data blood or brain tissue resource for research?**

01-In my opinion there should be, it is compulsory because those of us that came here today, we left our job and businesses to be here today, after this sitting and discussion if they do not make use of it, it will be just a waste of resources. If we do not have work or business to attend to, we can take our time to sleep at least blood will circulate round our body. Had it been that you will give us a million naira each now, it will meet some of our needs, but instead you give us pure water and biscuit, there is nothing you give that can compensate for the time we spared to be here. There must be a board that will work thoroughly and make use of what we discussed here.

02- The board should know, it is necessary to have a board because if there is no board all the things we are doing here is just a waste of time but if we have a board and there are people who would want to know about what they donated, it is the board that they need to go through. Someone whose parents donated organs after death and if they need to find out about one or two things will just go through the board instead of shouting in the hospital.

04- It is important to have a board even the bible confirms it without a board there will not be a success, so there must be a board

**Interviewer: What suggestions does the person who can help improve the lives of people by donating blood or brain for research work?**

**Interviewer: What suggestions do you have that can help raise awareness and improve people’s attitude towards blood sample or brain donation for research?**

02- It is not all the people you see on the street that are normal. The thought of what to eat and drink is what many people carry about as burden. If you want to engage such people in discussion of donation for research, the first thing to do is to meet their needs, this will make them listen to you. Is like someone going out for evangelism, he must go out with money, you win more souls with money because most of the households you will go to win souls you will meet the children crying of hunger if you want the parents to listen to you will have to first feed the children and you will need money to do that.

01-My suggestion is that our government should make provision for all people, provision of good health system that will go round the cities and villages. The western countries make provision even for the future of their people but in Nigeria the masses are suffering. If we donate brain, donate organs it is the rich that will enjoy the benefits. Our government should provide for everyone, let everyone have access to the resources of this nation. That is all I have to say.

**Interviewer: What other major concern or recommendations do you have related to use of blood for research in Nigeria?**

02- My suggestion is that if we find anyone who wishes to donate blood and later refused to donate, such person should not be forced to donate. The person who refused today might agree tomorrow. Nobody should be forced to donate blood.

04 - My suggestion is that, if it is not only on stroke alone, if we see anybody who need blood which is emergency they should not wait for the family before they can give such person blood, they need to commence treatment. It is very important for the government to intervene and rescue the entire community.
