# Supplementary material for: Research participants’ perception of ethical issues in stroke genomics and neurobiobanking research in Africa
Source: PLoS One. 2025 May 6;20(5):e0292906. doi: 10.1371/journal.pone.0292906 (PMC12054916; doi:10.1371/journal.pone.0292906)
Supplement: S3 File — (ZIP) [file pone.0292906.s003.zip › Files for PLOS ONE - updated March 2025/Ilorin_ Caregivers_FGD.docx]

**TRANSCRIPT OF FGD FOR CARE GIVERS**

**Moderator: M.A**

**Note Taker: B. F**

1. ***Moderator: Can you tell us what you know about genetic research.***

Participant 5. When you talk of genetic research, it is something you inherit, that is inheritance from let’s say any gene.

Participant 6. I quite agree with her, it is an inheritance; what you possess and pass to your offspring.

***Moderator* : That is what genetics is about but the research will be what?**

Participant 6. The research will be on the gene path, from the origin, lets say father, mother, step father, the whole family generally.

Participant 1. Genetic research means finding out or proffer solution to any related disease or sickness that can be inherited..

Participant 5. We can say endocrine system or hormones.

***Moderator*: *Does anybody have any experiences or others you know have had with participating in genetic research.***

*Participants shake heads to respond in negative*

***Moderator*: Do *you know about genetic research in stroke?***

*Participants shake heads to respond in negative*

***Moderator* : *Okay,so nobody does?***

*Participants shake heads to respond in negative*

***Moderator* : *What do we think is or can be the role/benefit of genetic research in medicine***

Participant 5. The benefits is it helps to know how to take care of stroke patients, the type of medicine, the advise, their diet and exercise. I think those are the benefits that can be gotten from it.

Participant 1. The benefits of this research will give further information or data on how to take care or prevent the occurrence of genetic illness.

2. The major benefit to me is that it will make the researcher to know the cure of the disease. It will make the patient to adapt to the prescribed drug that he or she will take.

Participant 6. The benefits are many. It will help the researcher in some areas like the status, what to do and patient to know his status as he doesn’t have to wait to get the disease before he knows he can have it.

Participant 3. Research is to help and guide stroke patients to take their drugs and make stroke patients happy. People should be around them all the time, they should not leave them alone.

Participant 4. There is a way to take care of stroke patients so that they will not be upset, angry or depressed.

**QUESTION 2**

***Moderator: Can we explain what you know about biobanking? What is our opinion about it? Have you heard about it? If yes, what is your source of information?***

Participant 3: I am hearing this for the first time.

Participant 2: I have heard it in hospitals like Ikorodu General hospital Lagos and Ikeja General hospital, so I’ve heard it several times . The major benefit is that it would enhance the researchers or the doctors to use all these things to cure the further diseases which is very important. However for thorough investigation because after keeping all these things they can still investigate on it. It will aid further investigation.

***Moderator: Do you know how it works?***

I do not know how it works. My perception is that it is good to keep it for further investigation.

Participant 5. How do we keep cells of the body? How do we do biobanking?

***Moderator: It will be stored with the use of machines and preserved for further use such that it does not go bad.***

Participant 1: Are we talking of importance of biobanking? I have heard about it as keeping of blood is also part of biobanking. It is very good but has to be properly monitored and not for personal use of doctors because of other things we have heard in the news, some doctors sell blood for personal gains. I am aware of policy guiding storage of blood based on my conversation with a doctor on storage of blood. Is it only blood that can be stored.

***Moderator: Context we are talking about is about is use of any living tissue for research.***

Participant 5: Regarding rules, screening must be done. The person must be screened for HIV before keeping the blood.

**QUESTION 3**

***Moderator: Can you explain what you know or understand about precision medicine?***

*Precision medicine had to be defined and explained to all the participants as none of them knew what it meant.*

Participant 1: I heard about it in this hospital (UITH) and some other hospitals. Although human beings are alike but we have individual differences that make us different. So the drug prescribed for Mr. A may be as a result of the test carried out on him so the drug may not be appropriate for Mr B.

I think precision medicine is good. I can’t think of any disadvantage because it is said that we should not practice self medication. It can be applied to management of stroke based on the experience I had while here, my dad was given Augmentin but they (doctors) stopped it at their own prescribed time.

Participant 5: I heard of it in a local government hospital that we should always take what is prescribed to us not for another person.

Participant 6: Self medication is what most people are used to. I remember when I was treating my uncle when he had stroke, many people came and said they were using different drugs and herbal medicines saying he should take but I told him it is best to take drugs prescribed for him. I think precision medicine is good.

Participant 1: In Nigerian law, use of unprescribed drug is called drug abuse and drug abuse is punishable under the Nigerian law.

Chorus response: Yes it is.

Participant 2: I personally do not like the idea of self medication as it can harm the body. In the hospital environment, treating patients according to their illness is good. For example in case of tuberculosis, individuals are treated according to their illness that means it is based on their body. I think it is very good.

*Participant 5 nods in agreement to number 2’s comment.*

Participant 6: Precision medicine is good. Prescription of drugs has to do with stages of the disease and I think it is good.

Participant 3: Even last week that I came to the hospital with my mother, one drug was discontinued due to cough.

Participant 5: I had an experience where Aldomet was changed because I was reacting to it by feeling dizzy.

Participant 7: My mother is also coughing but I don’t want to practice self medication even though she is still coughing..

QUESTION 4

***Moderator: What is your opinion about brain donation for research purposes? Are you aware of it?***

*Participants 1, 3, 4 and 7 gasp in surprise as Moderator explains more on the subject matter.*

Participant 5: I have heard of it through the media. They donate to assist others who are in need of it or for research. The benefit is tt can be used to save lives and helps the researcher to know more. It is good and I am willing to donate if I have the opportunity. It is good.

All what I have said is socially. Some religions will not accept it like Jehovah Witness and Deeper life. Advising and giving lectures can help create awareness can help promote awareness to make people to be willing to donate.

Participant 2: It depends on individuals, their perception. My religion Islam is against it, it does not support the removal of body parts after death for further research. Culturally it is a big issue, my culture (Yoruba/Ijebu) we cannot do that although some people might do it.

Sensitization can promote this awareness. People should be made to know the benefits via radio, Television and seminars. Individuals and educational institutions as well as work place, hospitals can serve as a channel. The community too can be used to create awareness.

Participant 6: Let us be sincere donation of brain is not easy. I cannot do it as it is against my religion (Christianity) and culture (Kogi). So how will I tell my family that when I die my brain should be removed? People will say “ Is this man mad” People will assume you belong to a cult that is why you want the brain removed. As an individual, I don’t buy the idea at all. It is of no use at all.

Participant *5 cuts in .....A person has gone (dead)*

Participant 6:.....When someone dies, the cells die so it can’t be used. The brain is dead already so what kind of research do you want to do? The brain is dead already.

Participant 5: I see it in movies that it is done.

Participants 6,7,3 *cuts in....that is in the movies*

Participant 6: It cannot be promoted in our culture. My family will not agree. There is no way such a thing can be promoted in our culture.

*Participant 4 shakes his head with a facial expression of disbelief at the thought.*

Participant 5: Remember I said socially it can be done.

Participant 6: The family ethics will not agree even if it is written.

Participant 3: (*shakes his head)* I cannot do it. Islam does not permit it.

***Moderator: Are there other ways to encourage people to donate?***

Participant 2: We are entitled to our opinions. Incentives can be attached to it to aid people to accept this. I mean monetary incentives.

QUESTION 5

***Moderator: What do you understand by blood sample for research? I am talking about donating blood for genetic research; I do not mean giving blood for donation to another person who needs transfusion.***

Participant 5: Is it about Rhesus factor?

***Moderator explains what it means***

Participant 1: I heard about it while a test ERC was done, I went for further information on this test. This gave me knowledge that blood is used for research. Genetic research through blood sampling is very good. I asked a question when I came in, I asked if stroke can be discovered through blood.

***Moderator: What are your beliefs cultural, social or religious?***

Participant 1: It is good if it is used for important things. My religion accepts it.

Participant 5: Socially I believe in it because it helps to save lives.

Participant 2: To a layman, it is good as it helps the individual as it may be useful in the case of emergency. Socially it is good, my culture encourages it, and my religion is not against it.

Participant 3: I think it is good to save lives and so I think it is good.

Participant 4: My religion is not against the use of blood to save lives. I think it is good.

Participant 6: I believe it is good if it is judiciously used. A lot of people outside here are scared as the trust is not there. Some people believe their blood can be used for money rituals or some other things. Some religions like Jehovah witness will not donate for studies.

Participant 7: Some people will not want to be part of it because they are HIV positive.

**QUESTION 6**

***Moderator: I want your opinion about blood sample donation strictly for stroke genetic research in particular.***

Participant 4: I will ask for the reason for the research.

Participant 2: The idea is good and I believe every individual will be willing and I am willing to be a part of it. The benefit is for the family as they will be sure that an individual is free from stroke. What may discourage people is delay in feedback; no monetary burden attached to it; point for specimen collection should be close to the participants and convenient. Family members too will be willing because it is very reasonable.

Participant 6: I think stroke is the most dreadful disease in Nigeria. I will donate but I want prompt feedback. My family will be willing to donate.

Participant 1: It is good and I will donate if it is convenient and financial incentives or food is attached.

Participant 5: Yes I will donate as it helps to know the status of the individual.

Participant 6: If it is mainly stroke people will do it. Stroke is rampant in our society.

Participant 3: It (stroke) is rampant so people will donate.

Participant 2: Communication should be done in language people understand.

QUESTION 7

***Moderator: Now moving on, we want to talk about informed consent. What do we know about informed consent?***

Participant 2: We can call it awareness.

Participant 1: Creating awareness is important.

*Moderator explains as participants do not know what the subject matter means. All the participants nod and show facial expressions reflecting an understanding as the Moderator explains informed consent and the 4 types)*

Participant 1: I prefer restricted informed consent because I want to be contacted for any other research. There is no need to involve any other individual. Data collected from me can be used in the event of my death.

***Moderator: I want everyone to respond to this question***

Participant 2: I prefer dynamic, I want the information in real time as I can change my mind at any time. I would want my children and wife involved before participation. I do not want my data to be used in the event of death.

***Moderator: Why?***

Participant 2: My information is personal.

Participant 3: I prefer restricted because I don’t want people to use my data after death but broad consent while I’m alive.

Participant 4: I prefer broad consent. I want my husband and children involved before participation.

Participant 5: I prefer broad because I want people to gain more. After death, data can still be used. I want my family members to be involved.

Participant 6: I prefer dynamic and data can be used after death.

Participant 7: I want my family to be involved in the consent process. I prefer broad consent so people can have knowledge. ( *laughs when probed on the use of data after death)*

**QUESTION 8**

***Moderator: I want to know what your opinions are on storing blood and blood fractions for genetic research. That is blood and part of blood.***

Participant 1: I think we have answered that question. It is good.

Participant 2: It is good.

Participant 3: It is good.

Participant 5: It is good.

**QUESTION 9**

***Moderator: Do you know anything about sharing data among researchers? What do you feel about sharing of information obtained from research?***

Participant 6: I think it is good because area of specialization is important.

***Moderator: With that being said, what will be your opinion on it being used commercially? Is it okay for me as a researcher to make profit from it?***

Participant 6: I do not buy the idea of commercializing it.

Participant 5: If it is commercialized part of the gain should be mine.

***Moderator: You are yet to tell me your opinion on sharing the data***

Participant 5: Sharing is good.

Participant 9: Sharing is good. Regarding commercializing, it is okay if many people will gain.

Participant 2: Sharing is good but I should gain if it is commercialized.

*General laughter*

**QUESTION 10**

***Moderator: A number of us have talked about the need for feedback, now I want to know your thoughts about feedback. What method or ways would you prefer for feedback if it is desired?***

Participant 2: Through comprehensive address (home address), mobile or social media

***Moderator: Social media? How?***

Participant 2: Through facebook.

***Moderator: Do you want the result through facebook?***

Participant 2: No, social media is to contact the person and agree on where to meet to be informed of the result. I prefer to be told in person.

***Moderator: If there are incidental findings, would you like to be informed of these findings?***

Participant 2: I want complete feedback.

Participant 1: I want feedback and I prefer phone contact as it may be difficult to meet face to face, the message can be given over the phone.

***Moderator: If there are incidental findings, would you like to be informed of these findings?***

Participant 1: I prefer to be informed of incidental findings.

***Moderator: What challenge do you think the researcher may face in getting back to you?***

Participant 1: Having my contact may be a challenge; so many channels of contact can be made. I will include social media so you may not have any challenge.

***Moderator: Are you aware of any social, ethical or legal issue that may be associated with feedback?***

Participant 1: I’m aware of social issue like bad network and things like that.

***Moderator: Thank you sir***

Participant 6: The joy of any researcher is to get the best results. I would like to get the feedback. I would prefer a written feedback via email.

Participant 9: The written is better. I would prefer written via SMS.

***Moderator: Can anyone think of any social, ethical or legal issue that may be associated with feedback?***

Participant 5: Communication issues.

**QUESTION 11**

***Moderator: What do you understand by bio rights? How much control should an individual have concerning the use of their specimen in research?***

Participant 2: It should be 50-50 rights between participants and researcher.

*Participant 1 explains it further and*

Participant *2:* It should be 100% rights to the participant.

Participant 6: I don’t think I have any right, all I want is feedback.

Participant 5: I should also have feedback. I should have 50-60% rights to sample use. If there is any profit, it should be shared 50-50.

**QUESTION 12**

***Moderator: What is your opinion on the use of biological specimens, on how it is regulated or governed? Do you feel that there should be a body overseeing how it is being used?***

Participant 1: Yes, there should be a regulatory body.

Participant 2: there should be a body.

Participant 3: There must be a body

Participant 9: There should be a body.

Participant 5: I agree there should be a body.

*Other participants nod in agreement.*

**QUESTION 13**

***Moderator: What suggestions do you have that we can use to raise awareness or improve peoples’ attitude towards blood and brain donation for research? I know we have talked briefly about it earlier.***

Participant 1: Environment (Nigeria) is largely determinant. Mass media should be used to sensitize people on how important. Financial incentives would also be of benefit in this environment.(food items like milk can be used).

***Moderator: Thank you sir***

Participant 2. Mass media. Religious leaders, lecturers/teachers, village chiefs, political leaders, associations can be used to sensitize. Religious leaders are very important.

Participant 5. One-on-one talk can also be used.

Participant 9. Awareness should also be at low level as well as even to children. This is to educate people and education has no limitation.

Participant 6. Awareness should be at grass root level. It may be easy to get people to donate blood but brain??! No it will be very difficult as it can be seen as a means to make money rituals. There is no amount of money that can be offered to make people donate.

***Moderator: Any other contribution?***

Participant 4:It may be hard to get people to donate brain specimens

***Moderator: But how can we get people to donate?***

Participant 4: I do not think there is any way to get people to agree to sort.

***Moderator: Thank you everyone for coming. We appreciate your time and effort.***
