# Supplementary material for: Research participants’ perception of ethical issues in stroke genomics and neurobiobanking research in Africa
Source: PLoS One. 2025 May 6;20(5):e0292906. doi: 10.1371/journal.pone.0292906 (PMC12054916; doi:10.1371/journal.pone.0292906)
Supplement: S3 File — (ZIP) [file pone.0292906.s003.zip › Files for PLOS ONE - updated March 2025/Ilorin_ Community Advisory Board_FGD.docx]

**TRANSCRIPT OF FOCUS GROUP DISCUSSION COMMUNITY ADVISORY BOARD**

**Moderator: O. A.**

**Note takers: M.A**

1. ***Moderator: Do I have your permission to start recording?***

**Chorus:** Yes

***Moderator: Do you have any questions before we start?***

Number 1: Yes, the question I have is that can one answer the question with another question?

***Moderator: Okay sir, you can answer any how you deem fit but l may not be able to offer you answer in the course of this discussion. You are the ones talking to us. Maybe somebody else in the group wants to answer, they will answer. Have I answered your question sir?***

**Number 1:** Yes

***Moderator: Thanks you sir. Any more questions? So we are starting now. The date is 15th July 2019 and the Time is 10:15am. My name is Dr. Olubiyi and I welcome you all to this focused group discussion. We want you to tell us about what you know about genetic research. Genetic research is the scientific study of our DNA, genes that make up a whole person. Have you read about it and what do you know about it?***

**Number 1:** In my understanding of gene along with research, is characters are transferred from parents to children and it can go on like that. So if a parent has a sickness so to say, it can manifest in the children too. That is my understanding.

***Moderator: Thank you very much sir. Number 4 have you heard about it and what you know about it***

**Number 4:** What I understand by genetic research is an experiment carried out either by Blood or through the chromosomes so that if, for example what we are talking about now, about the stroke, it can be hereditary disease that is from parents to children.

**Moderator: Thank you ma, anymore?**

**Number 3:** What I understand by genetic research is research on the genes, on how genes are transferred or how genes are inherited by generations of families. Maybe, through the family or through the paternal or maternal line or mixture of the two in a particular person.

***Moderator: Thank you ma.***

**Number 2:** Based on the fact that the gene encodes information and that information is what in most cases, is what make apparent in our daily lives. So when we talk about genetic research, it means they want to go into the source and see whether some of those things in the source are also reflecting outside or not.

***Moderator: Thank you sir, anymore?***

**Number 7:** To my own understanding, I feel this is a research based on those people who have certain disease or their own gene whether that thing is from the line of their parents so that it cannot relay to the oncoming ones that is the purpose of the research, like I've said my fore fathers had this it has nothing to do with them or it has something to do with them because I may have it from them but the purpose of this now is the people coming behind may not have it. That is the purpose of the research.

***Moderator: Thank you sir, so let's go a bit further to ask about if you have any experiences; what are your personal experiences or others that is participating in genetic research. Have you or people you know have participated in genetic research?***

**Number 2:** Yes, I have had the cause to relate with those who have been working on genetic research. And myself as well, I have also done a little it.

***Moderator: What is your experience, sir***?

**Number 2:** I feel it is a real thing because you have to go into the bases of life and you will be able to bring out a lot of information from it. However, it’s a cumbersome and sensitive research procedure.

**Number 5:** I've also had the opportunity to be part of a genomic research, breast cancer precisely which was conducted in UI (University of Ibadan), I was part of the research as an investigator as well. So I only came in to look at the views of the Numbers as to the autonomy in their participation in that research, so I have a limitation in the participation in that research. But, out of my own curiosity and personal zeal, I had the opportunity be informed of some level of the research after I had finished with them. Generally, genomic study or genetic study is a very flexible. Or how will I say? It is a very delicate study because, from the ethical perspective, it's a study that can break a whole community if information is not well kept. And the study then, the one I was talking of in Ibadan, specimen were being taken out through this “MTA” (material transfer agreement) agreement to US (United States) for analysis and it revealed some traits with the locality, with the rural environment that the Numbers were from. So they could be very helpful in getting very good information about the history of a set of people and as to how it relate with some of their ailment and the possible ways to channel the oncoming generation, just like baba (**Number 7)** said towards correcting those traits, in their gene.

***Moderator: Thank you very much sir***.

**Number 7:** I don't actually know if what I'm going to say relate to this sitting but I had to participate with professor Edungbola on research that has to do with people contacting some diseases. And we went through the whole lot of this country.

***Moderator: Was it a genomic research sir? Or a genetic research***?

**Number 7:** I am handicapped.

Collective laughter

***Moderator: Number 6? Number 9? Number*** ***8?***

**Number 8:** I have none.

***Moderator: What about genetic research in stroke? I know you've talked about breast cancer but has anybody been in stroke genomic research?***

**Number 7:** Don’t pass through that, in fact, I'm highly interested. Stroke, when we talk about stroke, we want to talk about genes; supposing we don't have it in relating to genes and it's stroke

***Moderator: Yes sir, that is why we are conducting this research***

**Number 7:** Because, I have my reasons, I am the patient of it and if you want me to tell you the background it is a long history.

***Moderator: Yes sir we are still going to ask more questions, right now I'm actually asking whether you know about genetic research in Stroke.***

**Number 7:** No.

***Moderator: Any other person? Does anyone know about genetic research in stroke? So what do you think are the roles or benefits of genetic research in medicine, I know you talked about breast cancer benefits that it may have so does anybody also have anything to say about benefits genetic research in medicine?***

**Number 4:** Apart from stroke, for so many years, several years ago, we worked in a genetic setup like this sickle cell anaemia that was in the North. So if you find out about this sickle cell too, it is a genetic problem that we are having Nigeria today.

***Moderator: What was the benefit of that research?***

**Number 4:** The benefit is that most of these children that have this sickle cell, they used to have their clinics, they used to treat them, and they advise them and guide the parents too.

**Number 2:** I feel the positive side of it, would be to improve mortality with the life expectancy and then to also help tailor this having this individual been treated as they are, not as in general. Taking the single thing to treat many people as the rate of their presentation and it also help to also reduce spread of diseases that are also genetically linked so it will reduce it and then like the sickle-cell mentioned. Once they know as this is this and counsel along that direction. That is the positive side of it, because some other thing may also come up from the genetic research that maybe complex to handle.

***Moderator: Yes sir, thank you sir.***

**Number 7:** The little idea of this that I have it's very beneficial, is of high advantage. Reasons are that one could draw from experience of what had happened in the past to what may happen to the people still coming behind, or like if I may, put in a little example, sometimes ago I mentioned about professor Edungbola, am sure my oga (Prof Wahab) knows him very well, and you know his discipline sir, I think …

***Moderator: Please sir, what was his discipline?***

**Number 7:** Epidemiology, in epidemiology, you have to study quite a lot of things. When we talk about epidemic, it means something that also happened somewhere, could it also happen elsewhere. And that was why we had to introduce it to President as at then (General Gowon), in trying to know what goes on in a particular place. We found out that people were having diseases in certain parts and how do we take care, so that it will not affect others in other areas. We went as far as drilling boreholes for them, outside giving them drugs. I am not in medicine but we were all introduced to it. So what we take for this side may not be useful for the other side so we had to know what could be good for that side before going into it.

**Moderator: Sir in essence benefit of this genetic researching in medicine.**

**Number 7:** It improved a lot of people; in fact today we cannot get any of these diseases in the areas we had gone. And I left the place some, maybe about 8 years ago and we realized that up till today they will still phone and tell me that there is nothing like that again, so this genetic research that we are doing is very helpful, long lasting and super.

**Number 1:** Well, to my mind, it can provide clarity and infer diagnosis of the disease being studied. And it helps in the selection of therapy and monitoring.

***Moderator: Thank you very much sir, Numbers 6, 9 and 8 please I want to hear from you too as well.***

**Number 3:** The benefits of genetic research are much, just like we have said, we talked about life expectancy, and it will increase the life expectancy of people when diseases are reduced. And then quality of life, people will know what is in their genes and how to increase their life quality, how they live their life, what they should take and they shouldn't take, maybe in terms of food or other intake and then exercise and the rest of it, how to live their life generally. Then, it also reduces the transfer of, sorry to say, “bad genes” from family, form parents down the generation. For example now genotype, the research on genotype that have done and also blood group, it has reduced drastically the Number of sickle cell in the community, because I know. For example, in my family, we have a lot of sickle cell but since we all know about genotype thing, all of us are now aware. Like me and my sisters, we are all AS, my dad and my mum were just lucky not to have the sickle cell in my own line, on my father’s and my mom’s, children. I think it is going to help a lot, for people to know their limitations, even in terms of marriage who they will partner with, who they will marry and the rest of it, maybe if they have similar genes.

***Moderator: Thank you very much ma, anymore?***

**Number 9:** I believe it is going to be very beneficial, not just now but in the long term. I want a situation where, I'm just recovering from stroke, my father died of it, two of my uncles and aunt died of it. I think it get to a stage where science will be able to tell my children that you will have stroke at so so so (particular) time and you have to prepare for it.

***Moderator: Thank you very much.***

**Number 6:** In short form, it is very good for early detection. That in a family circle, one would have known that you are prone to a certain ailment, for example the stroke itself. Presently as I am speaking, my mum has it and through the going all about for the treatment, we are been told that probably if care is not taken, that we the generation, that is the children or the grandchildren could easily develop it.

**Number 8:** Actually, the issue of this genetics research is very important, mostly to we human, more importantly to we businessmen. I lost my dad 2001 due to complication of stroke; it was Prof Wahab, that took care of him. Assuming there is a proper research on the issue of stroke, at least or with the little knowledge I have about it "most Dr. says they care but God cure" I put everything on that "care" You will be able to manage, before the incoming of the stroke or symptoms, you will be able to take care of it so that the thing will not happen. That is the purpose of the research to me mainly for caring to prevent stroke.

1. ***Moderator: Can you explain what you understand by Bio-banking? So bio-banking from the word, bio is like life, things from our body; banking is the storage. So, storage of biological material from the body. What do you understand by it? Are you aware that it exists? And what's your understanding or what do you feel about the concept of bio banking. (Translated to Yoruba language)***

**Number 5:** Like you so mentioned, bio banking, my understanding about bio baking is that it is the storage of part or whole of life that is whether tissues of the body, blood sample, liquid sample or what have you. Actually, in this part of the world it hasn't been a very common thing most of what we have like, that we could call bio banking, that is storage for particularly long period of time, is done outside the country, mostly US (United State) and the UK(United Kingdom), Europe. Although, we also have some nature of what we could call bio banking in our research institute like the EMRAT in which we were told of the ethical approval when you fill the consent form. They have malaria genetic study, and they have some other genomic study too whereby they try to improvise. One of the major factor for not having this so common around us here is that the environment that we keep this we don’t have stable electricity. There are some that you keep even below 0^o^C and for a long period of time consistently so those are the thing that may not give us the opportunity to have most of these things. There is the need, in most time before you transferred this bio-information that is whether blood sample or what have you, you ethically sign an agreement which is for the material transfer agreement (MTA) you transfer those specimen outside the country. But I think, there are also rules governing these agreement too, which are so much cumbersome and sometimes they go far beyond your ability here because the rules and regulations that govern where those things are there. Sometimes, when you don't have a full capacity as to ensuring that, then it tends to polarize or to make more un-confidential, the information that is been gotten from the specimen that are analyzed there because they don't only store it, they store it to get some analysis from it.

***Moderator: I'm still going to ask later about how bio-banking operates but I want others to answer their level of awareness.***

**Number 3:** Bio-banking to my understanding is body part like tissue, blood and other part of the body, to remove the organs like the parts of and the rest of it, that are kept in safe place, by experts, under certain condition, like the room temperature and the rest of it which must be regulated for the purpose of long and short term research.

**Number 4:** To my understanding, most of people don't know much about it. More so like Number 5 has said, in Nigeria we haven't developed up to that level because of the logistics like electricity and other things that we are lacking so we wouldn't know much about this bio-banking, except, thank God for this research that we have started today. Maybe as we go on now.

***Moderator: So you are trying to say you personally you don't know much about it.***

**Number 4:** More, so we are handicap because where we found ourselves there is no electricity and no equipment for that. As time goes on, we will still get there.

**Number 2:** From the word bio and bank just as you've mentioned, bio is just the tissue or fluid in the body of human being that are kept, collected it relate tissue, body fluid, preserve it and also use it for research. We retrieve those information from the bank and carry out a lot of research on it, like a reference center where they will be able to get information and predict this is this and also it can be used as a means of intervention and also for further research. Because without bank, you may not be able to, you know, we have this human genome that was done, was sequenced the whole of us. That we are not from there we are from here, so we may have differences so they try to say okay what accumulate all the differences and put in a place so when they see anybody if a research is done somewhere they want to match it up with this. Does it relate with what we have so they will be able to predict that if this happens here, it is a likelihood of this also happening in another whatever. So without that bank, the research, anything that has to do with gene cannot be conducted. They must have a bank and that is a reference where everybody can access and say okay they have done this one and we have also done it, it is also is existing and it doesn’t cause anything, they have done this one it is existing and it can cause something so that is the center that going to be a collection, preservation and retrieval where needs be.

**Number 9:** Mine is a question, do we have any level of bio banking in this environment?

**Number 1:** I feel it is just all about maintaining the sample technique, anything you collect how can we maintain it so that research can be carried out to enhance our understanding of health impacts?

***Moderator: For those that have talked, what is your source of information how did you come to know about bio banking?***

**Number 5:** Like I mentioned earlier, I partly participated in a genomic research breast cancer so I knew how we collect our tissue and how they are been transferred, so I was part of that. I had the knowledge of some other genomic study within because before I settled for the grant that was given my research, the program I went for in UI (University of Ibadan) was streamlined to only genomic study, so I had to look for every possible genomic study before I later finalized to what I settled for. So that was what made me know some other of genomic studies, like that of the malaria study and others. So I knew the way they were temporary preserving their own materials before they later transfer.

**Number 2:** I'm a student of knowledge, I have also try to update myself, I've attended a lot of international seminars on bio-informatics and I have also done some research on gene, that was what made me to have an idea about what bio banking means.

**Number 3:** I got information from some of these movies that we watch that they talk about transfer of bio materials and then, I am also a member of ethical review committee where we were given agreement to go through on transfer of bio-materials from one phase to another so when I saw it for the first time I had to go online to know more about it.

**Number 7:** I knew this a little bit time ago like I said from Professor Edungbola, we had to go into schisto(somiasis), oncho(ceriasis), lymphatic and even drancunculiasis. And going through this we had to store a lot of things in some places, in fact we got a sample that Jimmy Carter came into Nigeria and took it away to his bank and he kept it until tomorrow. He went through the proper channels, in fact the president (General Gowon) of that time, was involved. We got worm from somebody’s breast who was feeding a child and the longest worm ever caught was that, about 7ft 6" and the child was feeding on it that was why he had to take it and he took permission before he took it away.

***Moderator: Having talked about the existence of bio banks, how does bio banking operate?***

**Number 7**: I think we have all said it all here, when you get a disease from somebody whether you cut a part of the body or urine or blood or whatsoever, is normally sent for research and once they determined that the disease is real whether it is available in that place or available elsewhere it will be stored in a particular place under certain temperature so that it will keep for long and other people from other places can go there and find out what that thing looks like.

**Number 2:** Like I mentioned the other time this is a bank, once they collect the appropriate tissue or fluid, they go into analysis and once they analyzed it, most genetic whatever they sequence and see the genetic makeup and how it align so that is where they keep it. And when there is need to retrieve information from it, sometimes they code it in form of mathematical representation such that once you have a similar thing you can go into that and try to access from there. For example, the EMBL, all those centers where they have done the banking and they have done the analysis, they allow the information be known. The simple thing is like the blood bank that we have people go there donate they have O, A, AB, B they have it there so if anybody needs A, they will bring out A for that person. And sometimes, if you may want to carry research on A and they will be able to bring out who A1, A2, A3 and A whatever and group them to know that they are not ordinary A, there are other sub group of A, neither ordinary B there are other sub group of B. That is the essence of the bank, they have to do analysis and make sure that all information that partake to that thing is recorded. So that if we have a bank that has a Bombay now, for example, in Nigeria we are not of Bombay blood group, once they see there is a centre where they store blood and they have Bombay there if people from Bombay came to Nigeria and they need blood they will say okay you can only get blood from that bank where they have that Bombay blood group so that's the way they operate. It is information that has to be open so that anybody can have access that information, not information that is kept.

**Number 3:** I will liken it to a regular money bank where is well organized with the hierarchy of people working there maybe scientist, technicians, doctors and lab, you know, and a lot of people work there with proper safe, refrigerator because we know the body part can easily deteriorate and get decayed easily so they need all these like freezer, refrigerator and the rest of it, to keep those things that were either taken with permission from people or donated by people for the purpose of research. So the place is usually under some certain conditions whereby maybe everybody have to keep to some regulations like keeping clean, not wearing shoes in some particular places and the rest of it.

***Moderator: Thank you ma. Any other person?***

**Number 6:** I want to ask a question. All we are saying now is do we have a specific place for that in Nigeria presently?

***Moderator: Yes ma. Thank you for your question. Does anybody want to respond to that?***

**Number 5:** Like I did mention earlier or like Imam mentioned earlier Number two, he said this bio-banking, he just categorized it into three things you collate, you preserve and you retrieve for usage. So those are the three major elements that are involved in bio-banking. So when your collation, like I mentioned earlier, while I was speaking, I said even for our own a breast cancer genomic study, it is not immediately we collected the tissue from the patient, that we start flying it abroad, we have some levels of bio banking, they may not be to standard, they may, they may not take too long time. But at least we have some level of a bio-banking that exists within the country. So that’s the answer to it.

***Moderator: Thank you, Number 5. Number 3?***

**Number 3:** I think in this is our environment, we have some kind of bio-banking Like the blood bank, then the cancer center, I think cancer registry where they keep some samples taken from cancer patients. That’s the little I can think of.

***Moderator: Thank you ma. Okay so how important, we’ve talked about that so, everybody has talked about that. So how important is bio-banking to medical breakthroughs.***

**Number 5:** It is. Bio-banking is exceptionally very, very important to genetic or genomics study. Number one, like, just like we say, like Number three said earlier, there is no how you can store or there's, there's no you can use any part of the human body, or even non-human body animal without a having to store it, because it takes some time before you look at all that you need to look at. So there is the possibility that it's going to decay. And if it may not be palatable using it because of the odour and other things you will continue to perceive from it if you don’t store it well. So that's the essence. So there's almost not doable, a genetic study that you achieve without that storage, that bio-banking.

***Moderator: Thank you sir.***

**Number 3:** It is very important to medical breakthrough because it helps continuous research. Because sometimes when you take tissue from certain people, you will not be able to see those people again. Like, maybe those of us in this room now decide to donate or take part in research, some people may leave this environment and the rest of that and we may not see that particular kind of tissue or bio material again. So I think it’s very important to medicine and medical breakthrough. It helps even sometimes, later in the future, some other people, some other researchers may not need to still come back and take material but they can continue the research. This research we are doing now, somebody else can take it up from where he stopped and also go more into it and may not need to look for tissues and bio materials anymore but rather would rely on what you have taken or what you have in the bio bank.

**Number 7:** It’s very crucial. Like I said, initially if what we do is not well kept, I don't think research will come up and when you bank it, it means others can go into that. In fact, going to it, to find out that what you have done is very essential. They can, you can get research and somebody will refute, and tell you it’s a pure mess. Some drugs that have been used today are quite different from the words that have been used in the past. And again you’ll realize that like we talked about genes, drugs that are used for my grandfather may not be good for me maybe because I didn't take to his genes. I'm just using myself as an example. So it’s very, very I don't know how I can qualify that very. It is very useful, essential and we can we cannot do anything good without going through that.

***Moderator: Thank you very much. So let me come on to each one every one of us and so I expect that everybody will tell me how they feel about this. What is your own belief, your opinion about bio banking?***

**Number 7:** What I feel like I wanted to ask that time, I had Stroke some three and a half years ago. And none of my parents ever had it. My dad lived for 117 years he did not have it. My mother lived for 98 years, she did not have it. Maybe if they would have lived for longer than that longer than that they may have had it, I don’t know. My own came; from I don't know how many people will agree with me that there is such. I had stroke; we were 4 us struggling for one single post and three of us has had stroke. And one of us doesn’t sit, up till today they still carry him to toilet to defecate. They carry him everywhere to do everything. I thank God All Mighty. I had mine three and a half years ago. By this morning, I just told my wife I wanted to go for something, please put my water in the bath, I took my bath myself, dressed up myself, drove myself here and that was it.

***Moderator: And so what's your opinion about bio-banking***

**Number 7:** My opinion about bio banking is if research is kindly done and done well we may know or researchers will find out even if it is not in this country find out that this thing is not only through genes, because we never had it. So research may prove that there are other ways by which one to contact high blood pressure to come to stroke.

***Moderator: Thank you sir. Everybody is going to talk because I need to hear what you believe in, your thoughts regarding this***.

**Number 4:** Talking about this bio banking I think it is very important you for started. At least now that we have started, we should improve in it. So that this bio banking can be a reference that anybody can refer to in future. Not presently but in future refer, ok o go to so and so place and find so and so thing so on, so on so forth. And even the students that are still coming will refer to this bio banking and they will now know more about bio banking and other things too.

**Number 1:** To me, it is very, very good, very ethical, because it is all out to save life and save humanity from untimely death. It’s very, very good.

**Number 5:** I wouldn’t have talked but arising from what the last speaker, the last person said, you see they are very [pause], my view about bio banking has always been that they are very, very many ethical issues that surround Bio banking. Right from the collection of the tissue till the preservation of the tissue, it's a very laudable thing, particularly because it's science. And what science does is to look at what is happening, what has been happening till date to ensuring that there is better life for people tomorrow. So but there needs to be a very high level of respect for persons when it comes to obtaining tissue from anyone. It goes beyond after spoken like all that we are spoken now goes beyond that. Because you could tomorrow, tell me that from what you have collected, it can stigmatize my children; children yet unborn by saying your grandfather, your great grandfather has this. So there has to be very strict rules and regulations that guide the activities with bio banking. All of us want to make it good, make it nice, want to make the information, the bio information we have want to be accessible to anybody? But what are the things that govern assessment?

***Moderator: In addition to that, are you aware of any laws policy or guiding bio-banking in Nigeria?***

**Number 5:** Yes. There are but not in Nigeria here. Mostly in US (United State).

***Moderator: Thank you sir.***

**Number 8:** Actually bio-banking, it is just a little different between the commercial banking. does it be very, very easier to, to take reference simply because the word banking is simply for safety custody So if it's properly taken up, according to the name, it will be very easy for the research to go in there.

***Moderator: Okay, so let's move on. Any additions?***

**Number 3:** Just like I’ve said earlier, it’s a bank for future generations to research more for genetics and other matters relating to health and then in Nigeria, it’s good that we are also towing that line now you can see that people are already talking about bio banking, and other things. I think, I can’t remember where but I heard somebody talking about it one time, sometime ago about having bio banking in Nigeria the someone was saying that MTN, or something like that. I’m not sure, I didn’t ask for research further on that. Then it will help medicine, breakthrough in medical treatment and the rest of it. I’ve seen people donating their body parts when they are about to die like I heard of somebody who has donated his eye because he said his eyesight even up to the age of 70 that he had very good eyesight, he wasn’t using glasses, he said when he dies, he is a Muslim in Ilorin here, he has decided that when he dies he will donate his eyes for any other person who is need of it. And I have heard of people who have donated their hearts for health because they know that it will be kept somewhere where it can be of future or better use in medicine and then apart from that there are other, in every situation you have some ethical and non-ethical, those people the bad eggs in every profession who may want to who may want to start using those things despite, so having a bio bank must be very careful not allowing anybody lay their hands on it and start using it for something else like cloning, I don’t know much about it, but I think cloning might have its good and bad sides you know there is no how we don’t have bad eggs who will be involved, who will start taking such things and doing things that are unethical with it as much as it is a very good and laudable thing to do in medicine, we still have to be very careful so that the bad eggs will not start using it anyhow.

***Moderator: Thank you ma. So moving on to an aspect of bio banking, that is brain banking, so are you aware of brain banking? What is your opinion and how do you feel about it?***

**Number 5:** I think I have only heard of it but I have not had the curiosity to research to verify about it I don’t think exists around here and if it exists in some of these bio banks I would have had firsthand information about it. I don’t know much about it.

**Number 3**: Since any part of the body can be banked now like the heart, the eye, tissues, the kidney, a lot of parts of the body, brain is not an exception in this, just like any part of the body, it can be banked to be of better use. But what I always think is[laughs], if somebody else’s brain is transferred to another person, will it change the attitude of that person, or behavior or someone like me that is not so good in math, and then I have one[laughs] and becomes an expert in math, if I get brain transfer from maybe a math genius something.

**Number 6:** My own is a question again. The sustainability, how can it be sustained?

**Number 4:** Please I want to ask question on the brain. Can brain be transferred from one person to another? This is my question or somebody that is dead you now remove the brain, store it in a bio banking so somebody now.

***Moderator: For further research.***

**Number 7** interrupts: The fellow will not be dead before transferring it. It’s something else that makes him to live. It’s not the brain that makes him to live.

***Moderator: Please hold on sir. I’m not sure she done asking her question.***

**Number 7:** I want to correct her; I want her to be corrected

***Moderator: Number 7 sir***

**Number 7:** That is it the brain that makes you survive? No, I don’t think so. To say we want to transfer the brain to make that fellow survive.

***Moderator: We are still talking about brain banking; we’ve not talked about brain donation.***

**Number 7:** I think the purpose of banking it is to know what must have happened to that is to know what must have happened to that individual and so that others will learn, researchers will go out to people, not only within this country or not only within that country, that its being taken, it will go round the whole world, and people will learn from it. I think that's the purpose. If there is anything that is wrong with the other brain, this can be corrected.

I don't think the purpose is to transfer this brain; the brain is not banked for the purpose of transferring this brain to another person. If it is then I still need to ask a question. Does it mean to make the fellow act like what she said, non-mathematician to become a mathematician genius.

***Moderator: Thank you sir. Number 3 ma.***

**Number 3:** My question has not been answered?

***Moderator: Yes, Ma, as I said earlier, people in the group will answer the questions that you raise. I am the moderator. I'm not providing the answers. Thank you ma.***

**Number 3:** I know that the brain, what we are talking about here, brain banking, may just be for the purpose of research but it was just my fancy thinking that took me along that line, but I know that technology has gone beyond fancy thinking, a lot of things that we feel is not possible is now very, very, possible. So the possibility of transferring brain that is not dead, maybe if they are able to bring it out while the heart may have stopped but the brain is not yet dead, I think it takes a lot, it’s not immediately a person dies that everything in the body just stops midway like that. The heart may stop completely but I think maybe some other part of the body may still if it is taken out maybe under a particular Number of hours, to my, at least from what I have been reading about, it’s not immediately everything just goes dead like that. So definitely if it is taken out, on time and is kept in a condition that is fit for it to continue to live it can be transferred to another person. I think technology; technology has gone way, far beyond, we may not be so lucky to have good technology yet in Nigeria, but what we can do, or what our doctors can do. If they get environment, that are better off than this will be surprised. And I believe I strongly believe that the brain can be transferred to another person. I may not know whether if I get a brain transfer if I’ll become a mathematician, a genius in math, but I believe that just like the heart, the eye and other parts of the body, the kidney, the rest of it and I know a day will come, I heard that the liver cannot be transferred yet or something, I’m not so sure. I think we’ll get to that time when every part of the body can be transferred from one person to another. So the brain banking, tome it’s a very superb project which will go a long way to help continuity of life.

***Moderator: Thank you very much ma. So any more contributions?***

**Number 1:** my understanding of brain like it’s been mentioned, it has compartments, and it has sections, certain of it controls human functions. So I don’t see transferring a whole brain from one person to another person. So another idea, my understanding of bio banking, is like a bank becoming a warehouse of human parts. So if a section of brain is damaged, they can just go there look for that section, which must have been numbered too and replaced. But to remove the whole brain, and take it to another man might be somehow.

***Moderator: Any more contributions***?

**Number 6:** Excuse me. All these we’ve been saying, for the brain, that has never happened before in Nigeria presently, I’ve never heard, because why I’m just hammering on the safety, everything safety even the tissues and everything is an experience I just had about a common vaccine. You know the vaccines we give to children when they come to the clinic, you know recently they were shouting about they find polio somewhere in Kwara, like three places now, they said they find polio virus almost every, in some parts of Kwara, so that’s why they are doing this campaign of immunization. And just of recent again they find out that the vaccine for polio that they brought has already expired again. The one they even wanted to use for the campaign.

***Moderator: You are talking about sustainability***

**Number 6:** That’s what I’m saying.

**Number 4:** Contrary to what she said about this polio vaccine, the polio vaccine that they brought to us recently because I participate in the program. It is not an expired vaccine. The Out Break Response, this Out Break Response that we have just completed is not an expired vaccine because we had

***Moderator: Thank you for the information ma. This is not for this.***

**Number 4** interrupts: Contrary to what she has said, I’m now

***Moderator: After this you can now exchange information***

**Number 9:** I think we can safely exclude the issue of brain from our agenda. Because it is so futuristic, storing brain in particular. i have learnt in the media, in about 20,25 years, people will have choice to live forever because virtually all tissues can be manufactured, can be created in the lab. So like Number 3 said, there are possibilities, unlimited possibilities, but we are limiting ourselves to storage of tissues that are possible to stop. Am I right?

***Moderator: Yes sir***

**Number 3:** Just to say something about this, I think because of this research is on stroke and the brain has something to do with stroke. I think that’s the reason why this question on brain banking is coming up because there is no how you will talk about stroke that we will not talk about the brain.

1. ***Moderator: Thank you madam. So let’s move on please. What can you understand by precision medicine? Precision medicine is ok health needs are offered to patients based on their specifications, tailored to the needs of that particular patient. So what do you understand by precision medicine? Okay let me explain it again. It is a medical model that proposes customization of health care with medical decision, treatment and practices been tailored for the individual patient needs, disease condition and its own peculiarities. So what do you understand by it?***

**Number 7**: I think there is a need for both medical line and non-medical line to come together to understand each other to learn from one another before that treatment will be conducted and conducted very well otherwise, it would lead to something very different.

***Moderator: Sir you can tell us about the merits and demerits, the advantages and disadvantages***

**Number 7:** The advantage of it is that a lot of people will be taken care of and taken care off very well. The disadvantage of it is like so many nurses doctors are not in the but I didn't know who, those who follow up are being taught what to do.

***Moderator: Sir are we still talking about precision medicine? Each individual need tailored to that person. So what do you think are the advantages and the disadvantages of that***?

**Number 7**: The advantages of it is that one who live a lot of people will be saved for me but few may not and of this few that may not I happen to be one . When I was, admitted here in the hospital, I did not know I was coming to hospital because stroke had set in. I was brought in and people on my right and some people on my left were dying within minutes. I was quickly moved out from that bed, emergency to another place. In the cause of try to save this hand and this leg, this joint removed, this kneecap removed and that's why you see me still using this staff till today. Not only that, they were trying to save me or save this part. What I'm actually trying to drive at is when I went into physio, I had a different thing. When I went into physio, I was asked to bring ice blocks and they were placing ice block here and on my thigh. Blood refused to flow down. I had this place blocked, it became ice block that, no I'm being very honest, that you can't press it at all. So each time I come to my consultant, he will say hold, I couldn’t hold him. I have to use this right hand to hold the left one to go close to him. I thank God today that I can move it. So I'm saying, I don't know maybe our people don't follow the right course. What is good for “A” may not be good for “B”? Unless you know his genes. Study his genes very well before you knew how to apply paracetamol otherwise they will not call it paracetamol again, but adulterated paracetamol.

***Moderator: Thank you very much sir***

**Number 3:** Precision medicine to me is very good because just like you’ve been saying on and on, we have different body make up so, different people need different treatment. It’s just like in grooming our children, or the way we bring up our children we say that this particular child, you are to train this particular child in a particular way different from other people. That’s what I see with this thing. Precision medicine to me you have to look at the person holistically, this is what will be of benefit to this person. Not generalized treatment. This person will need this, this person will need a higher dose of a particular medicine or another person will need a lower dose of a particular medicine you know and then maybe different ways of handling their cases.

***Moderator: Thank you ma***

**Number 2:** If I get it right, the precision medicine is more or less personalized and individual. it would give prompt you know intervention you treat as appropriately and the person would recover rapidly, not trying this then later we change the diagnosis to this, change diagnosis to this and sometimes patient will be on conservative management for months, for three months until we get to ohh this is where the problem is and start to now manage. So it’s going to enhance quick recovery, it is personalized. It is going to be economical to save cost cause you can see spend a lot of money done a lot of things and then before they now come back to tetracycline can even take care of this instead of going to Augmentin or you know, cefuroxime and all these ones. The other side of this is it is also going to be expensive because if twenty patients come and you have to consider giving different drug for all of them, that means you have to stock a lot of different drugs, a lot of management and personnel, I mean staffing too. One person will manage this, another person will go to this one, go to this one and it will make it a little bit complex and there is also going to be expensive. You know while trying to say you want to personalize you have to do a lot of things before you do because no individual, we are unique, no matter how personalized we want to be, we are unique. Even if we are from the same father or the same mother or siblings or whatever we are still unique. No individuals are the same. So it is also going to create another complexity, and it will also mislead in some cases. So in trying to personalize you have to open another thing that did not even exist , trying to make sure that they want you to be different from this in terms of management so..

**Number 9:** So from my experience in the ward and after my discharge, my interactions with the doctors. I believe it takes two to really, it takes the patient and the doctors to really interact and get the best result. For example I have for the last three appointments, I have seen Professor Wahab and he comes, I’m able to open up. In fact my wife is always by my side. Some things I forget to say, she will say it. And because of that Prof has been able to tailor my drugs to my need and as at today my blood pressure is very okay, in fact the past one week very okay because of the interaction and his ability to my presentation and to adjust to my needs. So it takes the patient and the doctor.

***Moderator: Thank you very much sir. So I have been able to understand even from here that that a form of precision medicine was practiced with you and then Sir that if you had done, precision medicine type of care had been done for probably you wouldn’t have used a general method they used for everybody. Okay. Thank you very much sir. So, are you aware of any law guiding the practice of precision medicine? And what is your source of information as regards precision medicine? Where did we hear about precision medicine?***

Anybody? Are you aware of any law?

**ALL: (everyone shook their head).**

***Moderator: No one is aware of any law.***

1. ***Moderator: So, let us move on now to the part where we decided to leave before. What factors promote brain donation? Probe for cultural and religious reasons, peer values, parental influence, level of awareness, legal issues involved, knowledge of where it can be done, familiarity with medical and research settings***

**Number 7:** We have to talk about it if we know about it. I don’t think I have anybody who knows anything about it. I don’t think so. Please I am excluding the all the professionals.

***Moderator: Does anybody want to talk about the level of awareness of brain donation before we move to the cultural and personal beliefs about brain donation and the religious beliefs***

**Number 3:** Just like I have said before, maybe I used the wrong word before saying transfer. But I believe that technology is now very very advanced and by the grace of God, by the mercy of God that all these things are being known in medicine and it is for the benefit of the human race. I think when God, God will just, I think the white people, God will not just give them all these for maybe for fun of it but for the help of humanity and I hope that even in Nigeria, we have a lot of doctors thinking along the line of this, it is a major work anyway. So, brain donation to me is not out of the way, just like any other donation, I have seen the heart been kept in a particular place and it was beating. It may not be, it’s on, was it not on CNN or Aljazeera, I was watching the other day. The hospital setting was so fantastic, you’d think, Ha! I was thinking that is this really a hospital? I was a little bit down on that day after watching it. Sincerely, with medicine, technology, anything is possible. The brain can be donated just like a person can donate the eye. We know that the eye is not just there. I know that there will be a lot of little teeny veins and tissues and the rest of it that have to be joined together from removing one and putting in another one. Just like that, the brain too, you know there are so many complicated operations being carried out now apart from maybe just bringing out a child or so many other things.,There are a lot of complicated when my father in-law was abroad for an operation, they said it took 14 hours for the operation. So, I know that definitely there are this kind of brain donation will not be just *moinmoin* (simple; means bean-cake literally) kind of operation, where you just go in and maybe two hours later they say ha! Congratulations! *A ti pari* (we have finished), I know it’s going to be a long thing where all, a lot of doctors will be involved, those that will be monitoring the heart, those that will be monitoring the temperature, and a lot of things. A lot of things will be involved definitely. I think it is a very very possible project.

***Moderator: Thank you ma. Please I want to hear from every one of us because even if you have not heard or seen anything about brain banking, you should be able to tell us if there are misconceptions about it. If you are willing to donate your brain for research. So, I want to actually hear from everybody.***

**Number 8:** Actually, this is a funny thing. (laughs and everyone laughs). The issue of religious aspects is the most the major problem. Even if you make any this thing before you give up. Your religion, maybe in Islam, they would say it’s not allowed. So whatever you, whatever you wrote down, they may say No, No, No we can’t do that.

***Moderator: Is there a hadith or Quran verse that forbids it?***

**Number 8:** No, no, no. even in Christianity, we have many believe, beliefs are different. Some may say in our church, we don’t allow that. So definitely maybe you are the first son or whatever they say in Yoruba this thing… or in Africa, or maybe the *Olori ebi* (head of the family) says No you can’t do that. On the issue of religion, so, that will be the most important issue.

***Moderator: Will you be willing to donate your brain/your willingness to donate***

**Number 8:** Presently I have to go, you know I am a learner here. I have to read a lot of books, a lot of hadiths before I will sign. I know both the Christians and Muslims believe that it is God that gives the life and the bad habits too belong to the. So presently, I can’t say.

**Number 7:** I want to talk about whether one can do it. I am old, I can’t give it. I had stroke I can’t give it. So, I have many things standing against me

***Moderator: What gives you the impression that because you are old or has had stroke you can’t give it?***

**Number 7:** Do you want to give my brain to another old person? Or you make my brain younger?

***Moderator: We are talking about brain donation for research. It’s not brain transplantation.***

**Number 7:** If it is for research, I can give out any part of my body because at this age, I don't need any child. I don't need any wife. I don't need any girlfriend. I don’t need…what do I need? Only to wake up in the morning, take bath, eat and go back to bed. Initially. before I had stroke, I used to go out and take one bottle of beer. It won’t be more than one at any day and it won’t be an everyday thing. So why do I need to take beer. Right now, if you give me coke, I won’t take again, coke, sprite, Fanta (shakes head meaning No). I won’t take. So, at this age I can donate any part; donate my whole self for research. I am waiting for you to say come now, let’s go. (Everybody laughs)

***Moderator: More contributions?***

**Number 6:** Islamically, me, I don’t know much about hadith, what hadith say but I know of Quran. That I can’t donate anything. I want to go back fully.

**Moderator: Is it written in the Quran that you must go back fully with nothing missing.**

**Number 6:** I just want to go back like that and I pray almighty Allah will let me go back like that

***Moderator: Please I want to clarify if the Quran said that or you personally will not want to donate.***

**Number 6:** I personally will not want to donate

**Number 5:** It’s so funny, the aspect where we are in now. Actually, I know that to some, to some, for donation when you are talking about donation to some religious groups, they do not want anything mingled with their original body. I know those of the Jehovah’s witnesses. They don’t take blood and it’s confirmed. They do not take blood, they do not accept blood. But so, apart from that, I think, I wrote, I wrote a I had to research on Islamic ethics. On donation, receiver and having part of your body for research while I was on study, while I was studying bioethics. From my findings, from my findings, we had some schools of thought. I am talking from Islamic perspectives now. Because even the Genomic Study that I made mention of earlier. I, it was my final thesis while I was doing my bioethics study. And it was, it had some religious undertones. I, because at the centre there, there hasn’t been much research on religious views about some of these ethical concepts. So, I, I sacrifice myself to delve into some of those areas. In fact, while I got there I discovered that although I’m a Muslim and a little bit knowledgeable, but when it comes to some of these very recent modern ethical issues, I was a novice. So, I had to go into looking at the write-up of some of these… some of our scholars who have written on some of these ethical issues like Al-Ghazali, ?Benzi and some of them like that. So, there was some diversity of opinions in some of the spots. Most, we have some of these general views that are acceptable by when you talk of orthodox Islam.

To, the area, what I was able to observe, what I was able to conclude on, you know, you argue forth, you argue for and at the end of the day you state your own position. That’s normally what you do in ethics. There's almost nothing that is 100% correct or not correct, you look at the view of those who say it is correct, you look at the view of those who say it is not correct and you bring your own personal view. So, I, my personal view about some of these, like the issue of Euthanasia and some of these very complex issues were that even these, what do they call it? Even this surrogacy and some of these very critical issues, were that, in fact for what we are discussing, for the sake of humanity, for the sake of humanity, and I want to underline that, that once it is detected, that you are not going to suffer any, any harm. I want to define that any too, because there needs to be some levels before you say, it is not like you are not going to lose anything at all, any serious, I want to find the word to use for the “serious” exactly! any critical backwardness in your existence or on your life. Every life is sacrosanct. Every life is, is, is valuable to God. So, I believe you can assist any other human to sustain his /her own life documents to see his own life. I remember I reviewed words on some of these some of these critical issues, one of our courses was Medical Law. So, in those in, in that course, we normally look at issues that are being brought and how they were settled, then we critique their settlement, we look at your own views about this went and how that went. There was an issue.

***Moderator: Please I am sorry to cut you short because I am losing the train of thought. What we want to know is that as a person will you be willing to donate. What are the religious issues around it? What are the social issues around brain donation in particular. So that we don’t digress***

**Number 5:** See, it is because of the complexity of the issue. And that's why I have to go there, go there, go there, go there before I settle. Said, but if you can perceive from what I have been saying, I am for the FOR. I'm willing, I'm willing and I'm trying to bring here and there where I can justify my position particularly from religious point of view. Let me just give an instance and from that, I think I should rest my case, I have my imam here who can better settle on that discuss.

There is a time that during the life of Prophet Mohammed who we take as our model wherein they were passing through some levels of hardship and they don't want to disclose fully. Yeah. And it got to the point that some categories of people were almost losing their lives, were almost losing their lives, they were in need of water to sustain their life on the battlefield and they were about to… the water was being brought for someone to sustain himself.

As he was about collecting that water, he heard the cry of somebody else close to him that he was shouting of the need for water for sustainability. He then said, go and give it to the next person. He sacrificed his thirst and he knew he was almost dying. He knew practically that for him to leave, giving or taking this water is (means) almost dying. So, they were… as they were approaching the next person, as they were almost giving him too, he too heard the cry of somebody around him too that was saying, I need water for sustainability. He said, go and give it to that third person. By the time they took it…got it to the third person, they discovered that the person was already dead. They were trying to quickly bring it back to the second person, they discovered that he too was already dead. They were trying to bring it back to the first person and they discovered that he too was already dead. So that’s an instance to show that you can go to any extent to sacrifice what will sustain your life for the sustainability of another person.

**Number 3:** Personally, personally, personally, I can donate any part, any body part, even the brain if I get to certain age. Yeah! I may even make a will on that. If I get to a certain age, if God-willing I will be able to give it. And if my brain is still functioning very well or if it is going to be of benefit to humanity. Underline that, benefit to humanity. I am sure God will not punish me for wanting to sacrifice for the benefit of humanity. I get it, is it not ants and the rest of it that will eat the body? And that is my own thinking. That if I get to a certain age, and I’m able to reach that age and then death comes, if they are able to remove my brain, keep it somewhere for use.

**Number 4:** Thank you very much. It depends on individual, as we have been saying since morning. It depends on individuals. Some will go against it and some will support. As for me, as she (Number 3) has said, if I am old I can donate anything, any part of the body, because some people, they are donating kidneys, even when they are alive, they donate kidneys, they donate, other parts of the body. Even blood, you will see volunteers that donate blood just to save lives. So, it depends on the individual that wants to donate brain or not. But my first question was, how can we donate, or I mean… transfer brain to another person.

***Moderator: What are those factors that we think can promote brain donation and what are those factors that can inhibit or prevents brain donation (culture, religion, friends, family, the knowledge of where it is done)***

**Number 2:** I won’t take much time. I just want to raise these two points. There is difference between quality of life and prolonged life. We cannot prolong life, we cannot prolong any life, but we can improve, we can, we can, you know, make quality of life, you know improve, make, we can improve on the quality of life. That is the belief as far as Islam is concerned.

So, if we know we cannot prolong life as far as Islam is concerned, there's no premature death. As far as Islam is concerned, premature deaths or death before time or untimely death as far as Islam is concerned, everybody is with appointed time. Whether diseased or non-diseased.

So, if we are talking about quality of life, Islam supports quality of life to improve quality of life. But in terms of donating brain, organs, as long as it's not going to affect the person donating. But the brain as it is, the fundamental rule of Sharia in Islam is: you cannot donate your body for anybody after your death. After your death, you cannot say transfer my brain to this. It is wrong in Sharia. They are not expected to touch your body once you are dead. Nothing should be removed from your body once you, you stop breathing.

***Moderator: So, in essence, Islam is against brain donation?***

**Number 2:** Once you stop breathing, they must not touch anything in your body. They bury you like that, that's the fundamental rule.

***Moderator: Do you have Quranic verses to back it up?***

**Number 2:** Yes, Hadiths. It’s in Buhari, it’s in Muslim that said that none of you should touch or break the bone of a dead person. So, it's a classical, an expressed statement. And that is why if a pregnant woman dies, as long as the baby is not surviving again, I mean if the baby is dead… you know that if the woman died now even before we remove, the baby would also die because (of) the time. You don't touch that woman, you bury the two together. It's wrong in Sharia to say that, after all, they are already dead let us separate the mother, no, you bury the two together. It is also a very express statement and you have to bury them together.

***Moderator: So, religion may be a reason to prevent brain donation. Can other people talk about the other factors?***

**Number 2:** Sorry. Apart from that, we are talking; we must also differentiate between cells that are in active proliferation. Blood, you donate blood now, another blood is coming on. Donate at intervals and the bone marrow will produce more. And you cannot they will not bleed you, you can’t donate blood when you, when they know you're not so sufficient or you also enter go to coma. You cannot save life and also jeopardize your own life. Like blood donation is a safe; it pays the donor, it also pays the recipients. So, whatever will now affect the donor, they will not want it. So, talk less of going to brain.

***Moderator: You know in brain donation; the person is already dead. The brain donation is for research purpose.***

**Number 2:** You don’t touch it

***Moderator: I thank you very much. Any more contributions? None. Okay. Let’s move on.***

1. ***Moderator: What do you understand by blood sample donation for genetic research? We have talked about the brain. now let us talk about the blood sample. What do you think about this? What is your source of information?***

**Number 2:** Blood like I said, because of the nature of blood, there are a lot of information that is that you can deduce from blood when you carry research on it. More or less, it is central tissue that once you get that information from there, you can also, you know, go on and go on to also make deductions from it. So, taking blood for genetic studies is highly, highly recommended in Sharia, in Islam. Highly recommended, very recommended. Even when you are fasting as long as it's for research, you can collect the sample. Even when fasting you has to release because Islam also promotes, you know a proven knowledge.

***Moderator: Numbers 6, 9 and 8, you have not been talking. I want you to talk. What are the uses of blood samples donated for research and your sources of information?***

**Number 9:** I recall when I was on admission, the doctors brought so many bottles and collected so many samples. I believe it is for research. It is not as if I had the choice. You get me. It was not only for treatment but also for the purpose of research. Am I right? (asks the lead investigator who confirmed it). He explained to me. I know from experience generally, sometimes you get apprehensive if blood is taken, for fear of they may have diseases that may stigmatize them or things like that. Otherwise, I think it’s, it’s, it’s very helpful, it’s very useful for people to willingly give blood for research.

***Moderator: Does anyone want to say anything? No one. So, let us move on. He has talked about the Islamic aspects of religion; you have talked about Christians and the Jehovah witnesses who would not donate. Are there any other cultural factors that can affect blood donation for research and then, is there any law guiding all these blood donations?***

**Number 8:** The issue of blood donation, the major, the major set-back on the issue is the awareness, the awareness is poor. If the awareness is properly done, and it takes less to go to hospital and donate blood, but the awareness is poor. It is only those in the town or even within Ilorin, you will find it very difficult to tell some, for someone to come from elsewhere to go and donate blood in the hospital. So, the awareness is poor. On the issue of religion or with the government, the awareness is very, very poor.

**Number 3:** For, there are no legal impacts under the law. But under the law, I think the only thing the law is just concerned about is Consent. You must take consent for anything you want to do. Just like we have done this morning, you have taken consent before bringing us here. You must get the consent. Because once any person says this was done to me under duress or forcefully taken from me, it’s going to be a problem. There is no way, it’s very difficult to prove when the person stands on his feet and says, No, you didn’t take my consent. You say, okay but we told you that we are going to put the needle, we are going to draw your blood. And he said, but you didn’t tell me you were going to take, you know anybody can come up, lawyers can come up with any issue. Call *kinikan* (something ) anything and they will fly with it and they can get away with it under the law. So, consent is very important to explain the minutest, you know issue that will be involved in the blood sample donation and also any donation at any place.

**Number 3:** Under the law, you must take consent before anything is done. Once anyone says something was taken under duress or forcefully, it is a problem. Lawyers may be involved. It will be difficult to prove, once the person stands to say you didn’t take my consent, but we told you that we are going to use needles on you, anybody can come up with anything. Lawyers can make up any issue, they will fly with it. Let people know, naturally people want to save lives, if they are have the opportunity. Let them know that there is no adverse effect.

**Number 2:** Some of the challenges that will be associated are our cultural belief and getting sample, ordinarily, in Africa, a little thing we just give it another colour. They may belief that taking sample may take their future and if it is ordinary pants, it is becoming a case. If it is research now, they will say they already know their future and they want to use their future. Unfortunately, if you now give incentives, they will say, you see they are buying your future. If the child cannot pass secondary school exam, they will say it was the blood collected earlier that caused it.

**Number 1:** What is needed is proper and adequate enlightenment, let them know they are helping to save lives.

1. ***Moderator: Quickly, let’s share our opinions and thoughts on donation of blood sample donation for stroke genetic research, particularly for stroke genetic research.***

***Are you willing? What do you see as barriers to it? What are its benefits? And if you can go forth to talk about how our family members will feel about it? At times in African setting, it is not about us, it also concerns our family members? What if at that point you are unable to take decisions yourself, can our family members do so on our behalf? And how do they decide?***

**Number 7:** For stroke, I am prepared any moment even now to donate myself wholeheartedly. Nothing is against my donation. I know my God is not unkind God and he will permit me.

**Number 2:** I think everybody would love to partake in this stroke research because we are all victims and nobody is immune. Nobody expect it, it can just happen suddenly. Research in it will be well received, if it happens to a member of the family, it becomes a burden and cause of worry that you have to cater for the person, so if there is research on it, I feel it we be accepted almost 100% and people will coming out to freely donates, especially anybody above 45 years, the young ones may not say consider it as serious but those 45 and above, because they will have had one or two experience about it. Religious group may play a big role, because they are the one that can appeal to people. The media may not be as helpful as they may see it as a political issue and they would have mentioned a lot of things to contact if you will go to collect blood.

**Number 5:** In addition in our locality, we tend to leave in a communal social way of life as against the individual way that exists in western world. Considering this, the willingness to donate blood sample for stroke or genetic study, this might prevent people from doing it. Personally, I am worried about the result affecting/stigmatizing my lineage. There is need for confidentiality.

**Number 7:** The information should be kept safe and confidential. I did a research on 118 breast cancer patient and found out that 117 of them confided either in the husband on eldest son before they went into the study.

1. **Moderator: *I want to move on now, I still have some questions that are a bit related to the study, you can answer them then. Tell us about what you know about informed consent? Informed consent, that is when you voluntarily agree to participate in a research and then the types that we have, let me quickly explain some of the types we have. There is a restricted consent- in which a donor agrees to the use of samples or data collected for immediate research only and doesn’t agree to the use of samples in future then there is tiered consent- this is one in which a set of options is given to Numbers to select on how they want to participate in the research. The dynamic one is a web based one in which the individual are engaged, it emphasizes continuous re-contact with the biobank, they give them real time information on specific projects and they can engage as much as possible on the issues pertaining to the samples that are given and they determine how their personal information are used and there is the broad one. Broad form of consent is the one that allows you to collect, store and use samples later even for not yet determined projects. So we have 4 type-the broad, the restricted, tiered and dynamic types. I will like you to tell me, the ones your support and what you know about them and the reason for your choice?***

**Number 7:** I am prepared, broad consent is my choice; it will be helpful because my God knows who helped me change my case. I can make my decision myself.

**Number 2:** I will want to go for the broad one because in research, going back to take any other consent may be complex not be achievable. Research is broad and new things can come up.

**Number 5:** From the word informed, you need information about what you are volunteering into, if it’s based on what is written, I give my full consent. Beyond what it’s contained, I submit to restricted consent.

**Number 3:** I want broad consent.

**Number 4:** I support the broad consent. I remember when my daughter wanted to give birth , she had caesarean section, she is O negative, if not there is informed consent, I would have lost her. I think the broad consent is the best.

1. ***Moderator: I won’t ask this question again because we have already discussed it. (Your opinion on storage of blood samples and fractions?)***
2. ***Moderator: What do you know about sharing data? Do you know anything about sharing data? Sharing blood/blood fractions? Brain imaging? Whether is commercialized or not commercialized? Do you have any information on that? Does anybody know?***

**Number 7:** I don’t know

**Number 3:** I know there is something called transfer of biomaterial and tissues. There was a time someone was doing a research here and we had to help them do some agreement, clearance and transfer out of the country.

**Number 5:** Being someone who has worked in ethical review committee of the hospital, I am aware.

1. ***Moderator: we want you to share your thought about return of individual results and incidental findings. What do you think about it? How do you think one should receive the results of genetic research? It’s all about feedback, and if there is incidental findings? What is your opinion about it?***

**Number 9:** My view is that, can it be anonymous? In case of incidental findings, it may be useful to the society but devastating to the individual. There should be a way to make it anonymous.

**Number 7:** I think there should be a way to deal with negative finding. There should be a way the hospital should discuss with the patients.

**Number 3**: In as much as I believe when the result is out, it may be of benefit also to the donor, at the same time, the way the information will be disseminated should not be devastating and managed properly. There was a time I wanted to donate for open heart surgery case, blood sample was taken and I was called from the lab and I went there and right in the open office I was told that I had Hepatitis B. This could have been done more properly. When I got home, I told my husband and he said no problem , we went back to repeat it and it was negative. The way the information was given made me feel bad and I even shed tears. My problem is not the message but the way it was delivered. I prefer they tell me in secluded place.

1. ***Moderator: what do you understand by Bio-right? The rights to biological specimen for research. What rights do individual have? Are you aware of Bio-right?***

**Number 5:** As much as they consent to, one should transfer all rights. As mentioned earlier, we consider the benefit of the community in this environment, we are each other’s’ brother. Once consent is sought, informed consent is given.

**Number 3:** Right. If you give a broad consent, you should relinquish all rights while in restricted you have some rights over it so you should be contacted.

1. ***Moderator: What are your opinions about regulation of bio-banking? Should there be a regulatory body? Should there be an ethical body?***

**All:** There should be a regulatory body.

1. ***Moderator: explain possible interventions for implementation of bio-banking? What suggestions do you have that can raise awareness and improve people’s attitude towards brain donation for research and encourage people to adopt the practice***.

**Number 5:** A major setback is government will to ensure capacities for bio-banking, most research that involve bio-banking are founded by multinational/ international research. The view of this is to ensure change but first from the government. There is need for awareness. We also need to involve religious and cultural leaders (Emirs, Obas, Mogaji and Baales, and husband).

**Number 7:** I want to add that we go for community heads, they will go to their people and tell them what it entails. Nigerians are not all that bad, they will consent.

**Number 4:** We need to consult the traditional leaders, opinion leaders, youth leaders. If all these groups are informed, we won’t have many problems.

**Number 3:** As earlier said, religious leader plays vital role, they have a strong on their followers. Sometimes ago, I was in Abuja and I just entered through a marwa (commercial tricycle), you need to see the boys, ware saying in Hausa that they are ready to die for El Zaki Zaki (a religious leader), anything the man says to them, they will believe him and it is almost like that in all religions. Look at Jehovah Witness, there is nothing you can see to make them take blood transfusion. Religion plays a very important role, if you go through cultural leaders and religious leaders it should work.

1. ***Moderator: Are there some things we messed out or your concerns? About this issue of blood or brain donation for research in Nigeria?***

**Number 3:** My concern is on our facility, do we have the facility and infrastructure because it will not be fair to people who donate to see the materials are thrown into the bush when it is no longer in use, it should be taken out of circulation properly.

**Number 4:** There should be very broad awareness.

***Moderator: So, we thank you all for your precious time, your valuable recommendations, the discussions we have had today. It was an enlightening time. Thank you. We have come to the end at 20minutes to one o clock***.
