# Supplementary material for: Research participants’ perception of ethical issues in stroke genomics and neurobiobanking research in Africa
Source: PLoS One. 2025 May 6;20(5):e0292906. doi: 10.1371/journal.pone.0292906 (PMC12054916; doi:10.1371/journal.pone.0292906)
Supplement: S3 File — (ZIP) [file pone.0292906.s003.zip › Files for PLOS ONE - updated March 2025/Ilorin_ SIREN Stroke Cases_FGD.docx]

**FOCUS GROUP DISCUSSION (FGD) FOR STROKE PATIENTS**

**Moderator** Dr Afolayan M.A.

**Note takers** Dr. Abiodun J.A.

Transcriber: Dr. Tijani A.O.

***Moderator: Do I have your permission to start recording?***

**Chorus:** Yes

***Moderator: Thanks you sirs. So we are starting now. The date is 26th August 2019 and the Time is 10:25am. My name is Dr. Afolayan and I welcome you all to this focused group discussion. We can either discuss in Yoruba or English whichever is convenient for anybody, if there is any question that I ask and you are not comfortable with it, we have the Yoruba translation, please we want you to tell us what you know about genetic research.***

***Moderator: Number 2 sir***

**Number 2**: As far as I am concerned, when we say genetic research, it has something to do with genes. You either inherit from parents, something like blood transfusion. It is inherited from the genes from either of the parents.

***Moderator: Thank you sir, any other input please? Have you heard about it? Any experience?***

**Number 6**: Like he rightly said, something inherited from parents or family line issue. That is what I think.

***Moderator: Do you have any experience about any genetic research? Do you know anything about genetic research in stroke?***

**Number 6**: None at all.

***Moderator: Yes sir, Number 2***

**Number 2:** I have heard many experiences but not to me personally, in a family set up, if a problem starts with the father, children will share from it.

***Moderator: Are we aware of any benefit(s) of genetic research? Do you know anything about genetic research in stroke?***

**Number 2:** The only benefit that you can derive from it is if you know there is something in family set up, it will be easy to detect.

***Moderator: (Speaks Yoruba)….The first question is-- Have you heard about it? Any experience? Do you have any experience about genetic research? Do you know anything about genetic research in stroke? Are we aware of any benefit(s) of genetic research? Do you know anything about genetic research in stroke? We all need to participate.***

**Number 6:** (*Answers in Yoruba)…*Can we take the questions one after the other.

***Moderator: (Ask the questions again in Yoruba): Do you have any experience about genetic research? Have you heard about it? Any experience?***

**Number 6:** (*Answers in Yoruba)*… My experience is that my dad had it before he died. I feel myself and my siblings don’t take it serious. When I had it, my siblings and I now took it serious.

***Moderator: Number 5 sir.***

**Number 5:** (*Answers in Yoruba)…* I don’t have any family history. I just woke up, after morning prayers, I had a tingling sensation and I was rushed to the hospital where I was then admitted. Prior to having stroke, I was walking around, I just noticed I couldn’t carry my body and I was then rushed to the hospital and I was told its stroke.

***2. Moderator: If there are no further comments, let’s go to question two***

***(Ask in Yoruba, then repeat in English)….What do you know about biobanking? How does biobanking operate? How important is biobanking to medical breakthroughs?***

***What are you belief/thought/opinion relating to biobanking? Are you aware of any policy or law guiding biobanking?***

**Number 2:** *(Answers in Yoruba):* please ask again

***Moderator: (Ask again in Yoruba)… Please accept it the way it is written. What do you know about biobanking? How does biobanking operate? How important is biobanking to medical breakthroughs? What are you opinions about to biobanking?***

**Number 6**: (*Ask in Yoruba*)… Can we take it one after the other? Because if you ask too many questions, we may not remember.

**All**: *(Answers in Yoruba)…*what is biobanking?

***Moderator: (Explains in Yoruba)… Biobanking means keeping of live materials e.g blood, brain or part of the brain, or brain CT in a save place (bank, similar to conventional bank)***

**Number 6:** *(Answers in Yoruba)*…Apart from blood that is banked, I don’t know about biobanking. I have only watched it in the film that people donate part of their body in western world, instead of them dying with it, they rather donate it.

***Moderator: (Ask in Yoruba)… who else has an idea about biobanking? What do you understand by biobanking? Baba (Number 7), please answer, have you heard about it? Number 7 sir***

**Number 7:** *(Answers in Yoruba)* I have only read it in papers. My understanding is that it may be beneficial.

***Moderator: (Ask in Yoruba)…What is your own view?***

**Number 7:** *(Answers in Yoruba)*…My view is that it is God who has put it there i.e nature.

***Moderator: (Ask in Yoruba)…Are you aware of any law guiding biobanking? Number 2 sir***

**Number 2:** *(Answers in Yoruba)*…With the Nigerian constitution, nobody is allowed to take any part of the body without consent. Some people might be dubious and take a whole pint of blood,if someone finds out that something is taken without consent, it will cause trouble.

***Moderator: (Says in Yoruba)…Thank you sir. Do we have any addition, Number 1 sir, you wanted to say something.***

**Number 1**: *(Answers in Yoruba)*I want to ask a question, if a person is unconscious or at the point of death, if he needs blood, will he be able to give consent? They will rather contact his next of kin.

**Number 2:** *(Answers in Yoruba)…*If the person doesn’t agree, it is not possible.

***Moderator: (Ask in Yoruba)….We are talking about legislation, what is your view?***

***Any addition, Number 1 sir, Number 4 sir.***

**Number 1:** The blood will be beneficial

**Number 4**: *(Answers in Yoruba)*…Taking blood from someone for treatment is what I am aware of. I can give my blood voluntarily.

**3. *Moderator: (Asks in English then repeats in Yoruba)…what you understand by precision medicine? Are you aware? What is your understanding/perception of the concept? What are the benefits/demerits? Is it important in Africa? Can it be applied to stroke? Are you aware of any policy or law guiding precision medicine?***

**Number 4:** For the black or what?

***Moderator: Precision medicine is a model which proposes customization of healthcare with medical decision being tailored to individual person’s needs, condition and peculiarity (she then explains in Yoruba). What do you then understand by this?***

**Number 2**: *(Answers in Yoruba)*… If someone has headache, he should take drugs for it, however the drug that works for a person, may not work for another e.g someone that has peptic ulcer should take anti-ulcer drugs specifically prescribed for the person. We need to investigate carefully because our body is different.

***Moderator: Number 5, Number 3. (Ask in Yoruba)… What do you think will be the benefits for us in Africa? Number 5 sir***

**Number 5:** *(Answers in Yoruba)*…May God be with us, we should go to the hospital for treatment, not to quacks. In hospital, we will get the best of care. We need to seek medical help early. Arbitrary use of drug is not good.

***Moderator: (Ask in Yoruba)…You are saying it will be beneficial?***

**Number 5:** Yes

***Moderator: (Ask in Yoruba)…Does anyone have any addition***?

**All**: (silent)

***Moderator: (Ask in Yoruba)… Can we then say precision medicine will be beneficial in stroke management?***

**Number 3:** *(Answers in Yoruba.)*It is good. This will help doctors to manage patients well.

***Moderator: (Ask in Yoruba)…: What do you think about it? Where did you hear about it? Is there any laws guiding precision medicine? Number 7 sir, it seems you have anything to say?***

**Number 3:** *(Answers in Yoruba)…*I don’t think there is any law guiding it, otherwise we won’t be having people setting up private hospitals anyhow.

***Moderator: (Ask in Yoruba.)…I am not talking about setting up private hospital; I am talking about individual/personalized treatment.***

**Number 3:** Ok

***Moderator: Number 5 sir***

**Number 5:** *(Answers in Yoruba)...*I want to urge you doctors to research on the causes of stroke; maybe it is food we eat, use, do or our action. It affects people differently e.g when sleeping, working or walking, it occurs suddenly.

***Moderator: (Ask in Yoruba)…our understanding is that it may be beneficial, Do you think precision medicine can be harmful?***

**Number 5:** *(Answers in Yoruba)*…Some say it is caused by high blood pressure, excessive thinking, headache or stress can all lead to stroke. I don’t usually fall sick, I have never been admitted I have never taken IV fluids before I had stroke, it just occurred suddenly.

***5. Moderator: (Ask in English then repeat in Yoruba)…What do you understand by blood sample donation for genetic research? What are your thoughts on blood sample donation for research? Are you aware of any policy or law guiding blood sample for research? Are there cultural, social and religious belief on donating blood for genetic research?***

**Number 3:** It is a good suggestion and a good opinion because if it is been done, it will helps doctors/physicians generally to tackle such ailments or diseases. If they do research and they get results as to the causes, if it happens to another person, it will quickly be resolved or treated.

***Moderator: Any other contribution? Our source of information? Our cultural, social and religious belief on donating blood for genetic research? Are we aware of any law regarding blood sample donation for genetic research in particular? What do we think will result from such research?***

***Moderator: Number 2 sir***

**Number 2:** Religiously, there are some churches that don’t donate or collect blood (I don’t want to mention the church). If they are dying, they prefer to die.

***Moderator: Culturally sir (Number 2)***

**Number 2:** Culturally, It is now that scientist let us know of blood donation. Our fore fathers don’t donate blood.

***Moderator: Culture? Which culture sir?***

**Number 2:** For example in Yoruba land, there was no testing of blood then by the forefathers they don’t test blood, no whether you are A plus, once someone is sick, they only use herbs.

***Moderator: What you are saying is that our culture doesn’t support it?***

**Number 2:** Yes

***Moderator: Are you aware of any taboo?***

**Number 2**: No

***Moderator: I still want to go back on religiously? Religious belief, which religion?***

**Number 2:** Christianity.

***Moderator: Broadly or a sub-set?***

**Number 2:** A subset, Jehovah Witness, I will now mention, you can quote me. Jehovah Witness do not collect blood and they don’t give blood

***Moderator: Before we move on, from what you have discussed, what are the sources of information?***

**Number 2:** The source of my information is:-- my last born was sick, we were at FMC Iddo, he has leg problem, they wanted to amputate his leg, we needed 6 pints of blood and it’s a very crazy blood. He is O negative and we couldn’t get blood early. There is this Jehovah Witness who said he will give us concoction to replenish the blood.

***Moderator: Thank you sir. Number 3 sir***

**Number 3:** Biblically, in the days of Cain and Abel, God hated shedding blood.

Culturally, in Yoruba land, they call something rituals. They normally extort blood. It is something great. *(continues in Yoruba)* They can also use blood for money rituals.

***Moderator: What is your own personal opinion on donating blood for research? What are the things that may encourage or discourage you from donating your blood for research?***

**Number 3:** Donating blood for research is a good suggestion, it is a good opinion, it will help you the physicians or reseachers to contribute immensely to science.

***Moderator: You will be willing?***

**Number 3:** Yes, I will be willing to donate blood.

***Moderator: What may hinder people or encourage people from donating blood for research?***

**Number 3:** What may hinder people from donating is the way the researchers handle it. Baba said earlier on, for instance, a doctor wants to take a blood sample for malaria screening, the person may not know that it is only a little that is needed.

***Moderator: What can be done to encourage people to donate for research?***

**Number 6:** Awareness about blood donation is needed for people to be aware of it and its benefits. People do not know about the benefits.

***Moderator: So you are saying we should let people be aware of it and they should also let them know the benefits?***

**Chorus**: Yes

***Moderator: Any other thing, Number 2 sir***

**Number 2**: Now, I am still in the line of my brother (Number 6), At Ado Ekiti, for instance, there’s a blood bank there, they even say it on radio, encouraging people to donate blood voluntarily; they even say if you have too much of blood in you, it is another hazard, they are encouraging people to donate and when you are donating, they will not give you any *kobo*, you do it free of charge.

You only write your name down and they continue talking good about you; that you are a good Nigerian, you have done well.

***Moderator: For the blood bank, it is to donate for other people?***

**Number 2:** Yes, they people of the blood bank they don’t sell.

***Moderator: What we want to talk about in particular is donating blood for research. When we talk about donation for others, that one for donation requires a unit (that quantity is much); the one for research requires little, this is what we are talking about.***

***Moderator: You are willing?***

**Number 2:** Yes

***Moderator: What are the things that will motivate/encourage you to donate for research? Number 3 sir.***

**Number 3**: I will have suggest compensation for those who donated blood.

***Moderator: Where are talking about taking sample for research?***

**Number 3:** You can’t just call me to donate for research. I won’t even come.

***Moderator: Are you willing?***

**Number 6:** Yes, I am.

***Moderator: What are those things that can encourage or discourage you***?

**Number 6:** Like I said earlier, a lot of people don’t know the benefit of donating for research. People in the western world are willing to donate samples unlike here where people are not willing to donate blood samples. For example in the case of HIV/AIDS, people are not willing to share or talk about voluntary testing or submitting themselves for research. A lot of intending couples don’t know there status and are not willing to talk about it. People that have experience/knowledge about it should be ready to speak to people, you are supposed to enlighten people. If we have the knowledge of it, so many people will come up to save.

Two things; those of us in Nigeria, Culturally, we are not predisposed to being used for research? That may be a deterrent.

***Moderator: Do we have anything to say about your friends/family (people around you). Will it affect our willingness? Our family members? Our parents? There are different angles to seeing it, so this is a personalized thing and I want everybody to have their input.***

***Moderator: Number 6, 3, and 2 have spoken. Number 5 are you willing to donate blood for research?***

**Number 5:** Yes

***Moderator: What are those things that can encourage or discourage you? In terms of your religion, social, cultural, family and friends***.

**Number 5:** it’s ok. Religion can’t make me not to donate.like disease wey dey worry somebody.

***Moderator: if you are to talk on behalf of your family?***

**Number 5:** For research it is ok. I will do that.

***Moderator: Number 5, people that are close to you, if you are to talk on their behalf, will they be willing to donate or not? What are the things we need to do to encourage or discourage them?***

**Number 5:** they will

***Moderator: Number 6 sir***

***Moderator: the question is blood donation specifically for research? Are you willing? If you are to speak on behalf of your family? What are the things that may encourage or discourage you from donating your blood for research? Let’s think in that line***

***we have anything to say about your friends/family***

**Number 6**: are you talking about those that have HBP(high blood pressure) or stroke or just anybody?

***6. Moderator: Generally, anybody. Whether you are patient or not? Stroke research?***

**Number 6:** then, it is good for us. Creating awareness;

***Moderator: Creating awareness, what else?***

**Number 6**: Those that have had stroke should talk to their people. Like me it happened to me two months ago, I am a musician, when I had it, I just finished singing some few minutes to 8pm, after receiving the first call, second call and it happened to me, to me, I feel I am healthy but it happened. As a musician, I drink, I cannot hide this, I don’t drink to stupor, just to raise my morale, due to influence by friends, because I don’t drink before.

This thing I can say it because it has happened to me, don’t allow it to happen to you before you start to preach it. This thing should go round, father to children, like we can preach sex to avoid rape. Let’s talk in mosques, churches.

***Moderator: you are talking about preventing stroke, what I really want us to dwell on is donating blood for stroke research. It is not limited to whether we are patient or not.***

**Number 2:** If there is incentives, ok you will not have problem o, it will restore your blood. This will encourage people to donate. People will be willing to donate, some will not even wait till they are called.

**Number 6:** I don’t think these two ‘babas’ understand? Donation for research.

***Moderator: (Ask in Yoruba)…. What we are talking about is donating blood for stroke research? The amount of blood required is little compared to blood donation for people.***

***Moderator: What do you see as the barrier(s) that could hinder your donation of blood sample for stroke genetic research: family member, cultural and religious reasons, peer values, parental influence, level of awareness, legal issues involved, knowledge of where it can be done, familiarity with medical and research settings?***

**Number 3:** *(Answers in Yoruba)* as mentioned by number 6, awareness is key. Initially, you didn’t analyze it for us. What is the quantity of blood required for it? We didn’t understand you well before. Anybody who hears this will be happy to donate. Stroke is rampant in town. It is good for people to be aware about stroke research. There’s risk in it because I remembered when I donated blood for someone, I felt dizzy afterwards but my relatives got me “ malt’ and milk to to drink.

***Moderator: (ask in Yoruba) Any other thing?*** *(****ask in Yoruba) Numbers 1,7, 4 ; any addition? Number 1***

**Number 1:** *(answers in Yoruba)*…I can donate blood for stroke research. Researchers should inform people and they should be given gift/incentives to encourage people,

***Moderator: (ask in Yoruba)…what can discourage people?***

**Number 1:** *(answers in Yoruba)*…people may be discouraged if there is no incentives.

***Moderator: Number 7 sir***

**Number 7:** *(answers in Yoruba)*…people need to really understand it before they will donate otherwise they will turn it to another thing because our understanding is poor.

***Moderator: (asks in Yoruba) our culture too?***

**Number 7:** *(answers in Yoruba)*…religion

***Moderator: (ask in Yoruba)…religion, how?***

**Number 7:** *(answers in Yoruba)*…Religion also play a role. In the north, where there are predominantly muslims, they don’t even want to hear about blood donation. In my opinion, incentives will also motivate people. Government and organization also have roles to play.

***Moderator: (ask in Yoruba)… You mean government or organization has a role?***

**Number 7:** yes

***Moderator: (answers in Yoruba)…You have all talked about awareness and the likely benefits***

**Chorus:** *(answers in Yoruba)*…Yes

***Moderator: (ask in Yoruba)… Do you think a woman need to take permission from her spouse?***

**Number 7:** *(answers in Yoruba)*….Abroad, a woman has freedom, here a woman cannot just do that

***Moderator: (ask in Yoruba)…..What about taking permission from my parents?***

***Moderator: Number 6***

**Number 6:** *(answers in Yoruba)*…I don’t think you need to take permission from anybody once you are 18, you can just go ahead, after all there is no secret in it.

***Moderator: Number 7 sir***

**Number 7:** *(answers in Yoruba)*… I want to respond to that, it is not that simple. In Yoruba setting, it won’t be easy especially when your parents are still alive. I remember in 1962, I was with some people and somebody needed blood and when my parents got to know they were angry.

***Moderator: (ask in Yoruba) Like how old were you then?***

**Number 7:** *(answers in Yoruba)* I just finished primary school.

***Moderator: (ask in Yoruba) what will you say about a 30 year old person?***

**Number 7:** *(answers in Yoruba)*…. That person is mature enough.

***4. Moderator: (ask in Yoruba)…. Let’s leave blood donation. What do you understand by brain donation for research purpose?***

What factors promote brain donation? What are the cultural and religious reasons, peer values, parental influence, or other reasons that will influence your decision?

**Number 6:** *(answers in Yoruba)*… How do we donate brain? Since, we now understand that of blood donation for research.

***Moderator: (explains in Yoruba)….this entails donating brain for research after ones death.***

**Number 6:** *(answers in Yoruba)*… for me, I don’t have any problem with taking brain scans.

***Moderator: (ask in Yoruba)…let’s leave brain CT, talk about brain donation after ones death***.

**Number 6:** *(answers in Yoruba)*… Those in developed have better knowledge about brain donation especially when they die but here we don’t have such knowledge. However, if one is enlightened, I think people will cooperate. I am a Christian; the bible makes us understand that it is only the soul that goes to heaven. If not for money rituals, it will be easier.

***Moderator: (ask in Yoruba)…Thank you, Number 6, any other person, Number 5.***

**Number 5:** *(answers in Yoruba)*… may God be with us, the issue being discussed now, for muslims, we won’t allow it, once someone dies, if there is anything else they can use it will be better. However, some people may agree o. If there is any other thing, you can use, it will be better. I want to go God complete.

***Moderator: (ask in Yoruba)… Aside religion, what else can you say***?

**Number 5:** *(answers in Yoruba*)… Religion is the most important.

***Moderator: Thank you. Number 3***

**Number 3:** I am very sorry to say this, this question is strange. This should not be a disclosed question; it should be treated as confidential. *(Continues in Yoruba)*…on the day it happened to me, I drove myself to Ipata market, my wife told me to go and drive but I was suddenly paralyzed, I couldn’t drive back home. I was brought to the hospital and was asked to have CT scan, I was to pay N30,000, which I paid, all these investigations were not disclosed to my family members. You don’t need to ask us this question, you have unclaimed dead bodies in the mortuary, why not use them for research.

***Moderator: (ask in Yoruba)…it is not something you disclose or you don’t agree?***

**Number 3**: (answers in Yoruba)… Don’t get me wrong, all I am saying is, it shouldn’t be a disclosed question in a research. Nobody will agree to that.

***Moderator: (answers in Yoruba)… I want you to be specific, are you saying culture or religion?***

**Number 3:** *(answers in Yoruba)*… Both Culture and religion play a major role in brain donation for stroke research. They work hand in hand.

***Moderator: (answers in Yoruba)… so what do you think will make people change?***

**Number 3:** *(answers in Yoruba)*… No, not the brain!

***Moderator: Number 1 sir.***

**Number 1:** *(answers in Yoruba)* To me, I won’t agree. I will only agree, if it is to save my life. It is a taboo in Yoruba land for brain not to be buried with the body.

***Moderator: (explains the question in Yoruba)… what we are talking about is a dead person’s brain? i.e the person would have agreed prior to his death***.

**Numbers 1,2 and 3:** *(answers in Yoruba)*… the dead is gone, the family won’t agree, it is a taboo, they will say they have removed his brain.

***Moderator: (ask in Yoruba)… What do you think will make one to agree?***

**Number 2:** *(answers in Yoruba)*…repeat the question please

***Moderator: (ask in Yoruba)…what I am asking is, if I write will that if I die, they can use my brain for research and this research will benefit the upcoming generation, this is my question.***

**Number 2:** *(answers in Yoruba)*… culture will not allow it, even if I agree, my family members will not allow it because when the person comes back to life or reincarnates, he will not have that part of the body. If you notice, many people that die are the ones returning to life again. In the past, some babies that died and you remove a certain part of the body and they come back to life without such part of the body.

***Moderator: (ask in Yoruba) what if we now remove the brain?***

**Number 2**: *(answers in Yoruba)*If you remove the brain of someone and he returns, he won’t have brain and he will be an imbecile.

***Moderator: (ask in Yoruba)…any other reason?***

**Number 7:** *(answers in Yoruba)*… Ask again

***Moderator: (ask in Yoruba)… What do you think will make one to agree to donate brain for research?***

**Number 7:** *(answers in Yoruba)*… your question make sense but it depends on the society. We need to create awareness and educate each other. It is actually a good thing but it is difficult thing to implement.

***Moderator: (ask in Yoruba)… what can be done to encourage people?***

**Number 7:** *(answers in Yoruba)*… unlike number 5, In fact I am an Islamic scholar but I won’t use my religious sentiments on this but culturally I think it will be difficult. We also need to know more about the benefits.

***Moderator: (ask in Yoruba)…number 4, you have not said anything on this matter?***

***Moderator: number 4***

**Number 4:** *(answers in Yoruba)*… my own understanding is that in this part of the world, documenting such things is not common, it will take years for people to accept it. The documentation of the brain donor should be proper and they should be compensated e.g being immortalized.

***Moderator: (ask in Yoruba)… Ok, they should compensate those that donated samples for research***?

**Chorus:** yes

***Moderator: Any addition?***

**Chorus:** No

***7. Moderator: (ask in English then repeat in Yoruba)…Tell us what you know about informed consent? Are you aware of any law guiding research?***

***Moderator: number 3 sir***

**Number 3:** *(answers in Yoruba)*…In the constitution of Nigeria, it is not allowed, it is not permitted.

***Moderator: To do what sir?***

**Number 3:** To take samples without consent. It is important to take consent for genetic research. *(continues in Yoruba)*…A wealthy man who had 8 wives died in our area and it was the *will* they used in the law court to share his properties when there was family dispute.

***Moderator: (ask in Yoruba)…it is very important. any addition? What do you even know about consent?***

***Moderator: (ask in Yoruba)…number 4, you wanted to say something?***

**Number 4:** *(answers in Yoruba)* Legal practitioners are the best to let us know about informed consent.

***Moderator: (ask in Yoruba)… does anyone know about the types of informed consent?***

**All:** (silent)

***Moderator: (ask in Yoruba)…there are 4 types. There is broad, this means samples or data collected can be used for any research, not just limited to this research. Restricted consent means anything taken can only be used for that particular research; tiered means you have the right to choose from selected options available and dynamic is a web based form, this means you have agreed now and if there is need to change in future, you can withdraw anytime and it can be reviewed at anytime***

***Moderator: number 3 sir, which do you prefer and why?***

**Number 3:** I prefer broad, I want it to benefit others.

***Moderator: (ask in Yoruba)…do you want me to inform others (like your family or friends) before taking consent***?

**Number 3:** *(answers in Yoruba)*…very necessary, my wife and children need to know.

***Moderator: (ask in Yoruba)…number 2 sir, which type of consent do you prefer and why? Do you want me to inform others?***

**Number 2:** *(answers in Yoruba)*… please repeat the types available.

***Moderator: (ask in Yoruba)… there is broad, this means samples or data collected can be used for any research, not just limited to this research. Restricted consent means anything taken can only be used for that particular research; tiered means you have the right to choose from selected options available and dynamic is a web based form, this means you have agreed now and if there is need to change in future, you can withdraw anytime and it can be reviewed at anytime.***

**Number 2:** restricted consent *(continues in Yoruba)*….because I can’t decide for my child, I can only decide for myself.

***Moderator: (ask in Yoruba)…I am not talking about consent for your child, I am talking about taking consent from you and there is broad, this means samples or data collected can be used for any research, not just limited to this research. Restricted consent means anything taken can only be used for that particular research; tiered means you have the right to choose from selected options available and dynamic is a web based form, this means you have agreed now and if there is need to change in future, you can withdraw anytime and it can be reviewed at anytime***

**Number 2:** (answers in Yoruba) broad,

***Moderator: (answers in Yoruba) why?***

**Number 2:** (answers in Yoruba) for the betterment of others

***Moderator: number 1 sir.***

**Number 1:** broad

**Moderator: why?**

**Number 1:** *(answers in Yoruba)* I want you to inform my wife(s). so that they won’t be caught unawares, so that they won’t say they were not informed.

***Moderator: (answers in Yoruba) do we have anyone who prefers another form aside broad?***

**All:** (General silence)

***Moderator: number 7 sir, number 6***

**Number 6:** it is not clear to me

***Moderator: you have broad, restricted, tiered or dynamic. In broad, you have given me consent to use your information, (whether it is qualitative or quantitative data) or I have collected sample from you, I can use the information for which ever research I come up with; in restricted, you have given me consent for this particular research, I can’t use it for any other thing I come up with; in tiered, you can choose which research I can use it for and which I can’t use but in dynamic it is web based, you can always pick, I have to communicate with you. You know in broad I can continue using it, in restricted, if something comes up, I can only use it for this research, while in dynamic, you go along. The question is which of the 4 are you interested in and why?***

**Number 6**: broad, since it is beneficiary to others.

***Moderator: people to be involved in participation, Number 6, for your own particular information, would you prefer we involve other people like your friends, family, perhaps your lawyer? Which other people would you like to be in the process of getting consent from you?***

**Number 6:** I alone.

***Moderator: alright, any other contribution sirs, before we move on.***

**All:** (General silence)

***8. Moderator: (speaks English, then Yoruba)… the next thing is what is your opinion on storage of blood samples and blood fractions for genetic research?***

Number 7: *(answers in Yoruba)* everything is for the benefit of humanity, based on that it is OK.

Moderator:*(ask in Yoruba)*..Thank you sir, any addition?

**Number 2:** *(answers in Yoruba)*I agree. They should continue using the blood because I cannot get the blood back. After all, you are not asking for another blood and I cant get it back again, they should continue using it.

**Moderator: (says in Yoruba)… is it applicable here?**

Number 2: *(answers in Yoruba)*.it is possible, for me to have agreed in the first instance, means I want it, since it is not possible to get back my blood.

**Moderator: says in Yoruba)…number 3, you wanted to say something?**

Number 3: *(answers in Yoruba)* if we are talking about, it research doesn’t go empty, I remember Dr. Abalaka discovered cure for HIV and he became wealthy man. If you take my blood for research and it becomes fruitful, what will then be my benefit.

***Moderator: number 6***

Number 6: *(answers in Yoruba)*I want to go against daddy (number 3). What have we paid someone who researched on mathematics/arithmetic that we all have benefitted from? Do we know what he has put into the research? Do we need to benefit anything before participating in research?

***Moderator: there is no right or wrong answers.***

Number 3: I am a publisher, author *(continues in Yoruba)*…I have published some works on chemistry and I made some money through it. I used people’s brain to achieve this; thus I have gained from them and those that bought my book also gained from me.

***Moderator: (says in Yoruba)… we need to move on, we are digressing a lot, after the programme, we can discuss that, and there is a time limit for the discussion. We have to cone down than to the topic***

***9. Moderator: (speaks English then explains in Yoruba).what do you know about sharing of data, blood/blood fractions, brain images, as well as brain tissue samples? Sharing with researchers locally or abroad?***

**Number 2:** yes, you can share with others, we too can gained from their own research. For example they can do research we have not done, we can benefit from them. We too can do research and they will benefit from us.

***Moderator: (says in Yoruba)… We are not talking about result, it’s the data.***

**Number 2**: there is nothing bad in that.

***Moderator: (says in Yoruba) sharing data with another researcher for the study***

**Number 2**: it is like you are spoon feeding the person.*(continues in Yoruba)*you must reach a conclusion first, if you give your data and they are able to get break through First, they will get the benefit.

***Moderator: (says in Yoruba): we may not be working on the same thing***

**Number 2;** not the same topic, then there is nothing bad in it.

***Moderator: number 5 sir, what is your opinion?***

**Number 5:** I don’t understand

***Moderator: (says In Yoruba): what is your view about sharing of blood sample or data?***

**Number 5***: (answers in Yoruba)*…I won’t agree.

***Moderator:( ask in Yoruba) what about commercializing it?***

**Number 5**: *(answers in Yoruba)* I don’t agree. I will also not allow for commercial use.

***Moderator: number 3***

**Number 3:** *(answers in Yoruba)*..As for me, it is good. If you are successful, my motive is for others to benefit.

***Moderator: (says in Yoruba)…. You mean, you agree to commercialization?***

**Number 3:** *(answers in Yoruba)* I agree for commercial use, let it benefit others.

***Moderator: (in Yoruba) Numbers 6, 2 and 1; anything?***

**Numbers 2 and 1:** (nods) we agree.

***10. Moderator: (speaks English then repeat in Yoruba)…What are the ways that you think one can receive the results of genetic research? What are your thoughts on returning individual research results and incidental finding? Ways they would prefer to get feedback: Phones, email, letters, feedback by a healthcare worker ? A researcher? Or a clinician?***

**Number 3:** *(answers in Yoruba)* it seems the question is fluctuating, I am not clear?

***Moderator: (repeats in Yoruba): What are the ways that you think one can receive the results of genetic research? What are your thoughts on returning individual research results and incidental finding?. Ways they would prefer to get feedback: phones, e mail, letters, feedback by a healthcare worker ? a researcher? or a clinician?***

**Number 3:** *(answers in Yoruba)* I will like to get feedback, invite me back, when you took my blood, you invited me so you have to do same when result is ready. If there are any incidental findings, I will like to know.

***Moderator: (says in Yoruba) Who do you prefer to tell you the result? The person who took the sample or the researcher?***

**Number 3:** *(answers in Yoruba)* the person that invited me.

**Moderator: *(ask inYoruba)* what about incidental findings?**

**Number 3:** *(answers in Yoruba)* I want to know

***Moderator: (says in Yoruba): in what ways do you prefer to get the result, number 2 sir.***

**Number 2:** *(says in Yoruba)* I prefer to get feedback physically, so that the researcher can give detailed explanation and incidental findings. Even if I have questions, I will be able to ask.

**Number 3**: *(answers in Yoruba)*…for example, you take sample from me and I die before the result comes out, I want my next of kin to be informed about the result.

***Moderator: (says in Yoruba):Yyou are saying, if the person dies, next of kin should take charge?***

**Number 3:** *(answers in Yoruba)* yes. Since you doctors do autopsy.

***Moderator: number 1 sir***.

**Number 1:** *(answers in Yoruba)* you should invite me back and I may take my next of kin along.

***Moderator: (says in Yoruba) how do you want the feedback? What are the social and legal issue that may come up***?

**Number 2**: *(answers in Yoruba)* you will invite me back, I may also come with my next of kin

***Moderator: number 7 sir, number 4 sir***

**Number 4:** *(answers in Yoruba)* I want it privately

***Moderator: (ask in Yoruba) individually or group?***

**Number 4:** (in Yoruba) individually.

***Moderator: number 6 sir***

**Number 6:** *(answers in Yoruba)* As for me, it depends on a lot of things; anyone that is convenient. I can get the feedback physically, through phone call or email and if there is anything I don’t understand, I will reach out to the researcher.

***Moderator: (says in Yoruba) in a nutshell, everyone wants to get result?***

**Chorus;** (*(answer in Yoruba)*) yes, we want to get result

***Moderator: (in Yoruba) any other thing; legal or social?***

**Chorus:** none

***11. Moderator: (mixes Yoruba with English) what is your understanding about bioright? How much control should/can individuals have regarding how their biological specimens will be used in research? What rights do/should individuals who provide their specimens for research have over their specimens, how they are used in research, and any profits from research discoveries made possible from them? How should autonomy rights be best balanced with societal benefits that derive from the use of human specimens in research?***

**Number 1:** *(answers in Yoruba)* I don’t think we have any right since we have given consent

***Moderator: number 2 sir***

**Number 2**: *(answers in Yoruba)* since they have collected the samples, there is nothing we can do.

***Moderator: (ask in Yoruba)your belief is that you don’t have right but do you think you should?***

**Number 2:** *(answers in Yoruba)*we should but if you lease something you don’t have a right on it.

***Moderator: (ask in Yoruba) any other thing?(then says in Yoruba) We are getting to the end now.***

**Number 3:** we have right.

***Moderator: (ask in Yoruba) which kind of right?***

**Number 3:** *(answers in Yoruba)* I said I donated blood at FMC Owo and I felt dizzy after. It led to an argument and the person I donated blood for was begging me.

***12. Moderator: (ask in Yoruba. What is your opinion about governance and regulation of biobanking?***

**Number 2:** *(answers in Yoruba)*. You can continue using it.

***Moderator: (ask in Yoruba) Do you think there should be a body regulating research?***

**Number 1:** *(answers in Yoruba)*. I believe you won’t do anything against the law. There is no need for governance and regulation since I willingly donated.

***Moderator: (ask in Yoruba) …Is there need for a regulatory body?***

**Number 1:** *(answers in Yoruba)*. There is no need, since I have given you freely and I trust you.

***Moderator: number 3 sir.***

**Number 3:** (in Yoruba) there should be a regulatory body. In Africa, we normally abuse things. During the tenure of Obasanjo, he set up EFCC and ICPC to curb excesses.

***Moderator: number 2 sir***

**Number 2:** *(answers in Yoruba)*. There should be monitoring among you researchers

***Moderator: number 6 sir.***

**Number 6:** *(answers in Yoruba)*. It depends on how government sees it. I know it is prone to abuse, this depends on how the researchers use it to benefit humanity.

***13. Moderator: (says in Yoruba) What suggestions do you have that can help raise awareness and improve attitude towards blood sample or brain donation for research and encourage people to adopt the practice?***

**Number 2:** *(answers in Yoruba)* awareness on the importance of biobanking;

***Moderator: how do we create awareness.***

**Number 2:** *(answers in Yoruba)* you can create awareness on radio,tv and internet. We can also do community sensitization, including religious bodies- churches and mosques. Another thing is that there should also be compensation, people that donate should be given something.

***Moderator: (says in Yoruba). Aside awareness, what else?***

**Number 2:** *(answers in Yoruba)*. There should be given compensation,

***Moderator: (says in Yoruba) how?***

**Number 2:**  *(answers in Yoruba)* they should be given gift?

***Moderator: (ask in Yoruba) what other thing aside compensation***?

**Number 3**: *(answers in Yoruba)*. House to house campaign should also be, as for me, I don’t watch TV nor listen to radio. Traditional rulers should also be involved.

***Moderator: number 1 sir***

**Number 1:** *(answers in Yoruba)* Create awareness and use free medical services aespecially in the rural areas

***Moderator: number 7 sir, any addition?***

**Number 7:** no comment

***Moderator: number 4 sir***

**Number 4**: awareness is key, carry people along through seminars; one on one contact and in public gathering. For us to come from our different places to come volunteer for this research (for the service of humanity) is because you carried us along.

***Moderator: thank you sir, number 6 any addition.***

**Number 6:** Nods to say, NO

***14. Moderator: (english and then Yoruba)…in conclusion, do you have any other concerns or recommendation concerning donation of blood or brain for research.***

***Moderator: number 3 sir.***

**Number 3:** *(answers in Yoruba)* thank you, I want to suggest that this kind of FGD should be a continuous exercise. You should be inviting people seeking their consent. It will be a great help for the society.

***Moderator: any other contribution sir?***

**All:** None

***Moderator: any contribution from doctors?***

**Doctors:** None

***Moderator: (says in Yoruba) So, we thank you all for your precious time, your valuable recommendations, the discussions we have had today. It was an enlightening time. Thank you. We have come to the end at 12.42 pm.***
