# Supplementary material for: Research participants’ perception of ethical issues in stroke genomics and neurobiobanking research in Africa
Source: PLoS One. 2025 May 6;20(5):e0292906. doi: 10.1371/journal.pone.0292906 (PMC12054916; doi:10.1371/journal.pone.0292906)
Supplement: S3 File — (ZIP) [file pone.0292906.s003.zip › Files for PLOS ONE - updated March 2025/Ilorin_ SIREN Stroke Free Controls.docx]

**TRANSCRIPT OF FOCUS GROUP DISCUSSION FOR CONTROLS**

**Moderator: M.A**

**Note Taker: B. F**

1. ***Moderator: What do you know about genetic research? Have you heard about it? What do you know about it?***

Participant 2: It has to do with genes to find out if there are ailments that can be pass parent to children.

Participant 1: To my lay man understanding, it’s a research being conducted through generation or through family to find out if illnesses in family can be traced to run in families.

Participant 7: Gene has to do with the issue of family, if one person has a child it may circulate, from a father to the son.

***Moderator: Tell us about any experiences you or others you know have had with participating in genetic research.***

Number 7: I have never heard of anyone being involved.

***Moderator: What do you know about genetic research in stroke?***

Number 1: What I know is hypertension runs in families and can cause stroke.

Number 5: I know of a case that the mother has ulcer where at least three of her upspring has the peptic ulcer disease. I was also told that the father to that mother also had the disease.

Number 3: Concerning hypertension, what I know is that if someone has it, half of the children will have it.

***Moderator: What do you think are the roles/benefits of genetic research in medicine?***

Number 6: It will help create awareness as well as identify precaution to avoid it.

Number 4: It will bring about good health to the individual.

Number 5: The research will result in a reduction of the occurrence in the society because it will be possible to know what possible causative factors to avoid in order to prevent the disease.

1. ***Moderator: Explain what you know about bio-banking. How does biobanking operate? Awareness, understanding/perception of brain banking.***

Numbers [5; 2; 4 shakes heads to indicate they have not heard about it]

Number 6: I have heard about blood bank. I do not know much about bio banking.

***Moderator: Belief/thought/opinion relating to biobanking.***

Numbers 3: It can be used to help humanity in the future. It is a good thing to help investigate illnesses within a family.

***Moderator: Awareness of any policy or law guiding biobanking.***

No response

***Moderator: How important is biobanking to medical breakthroughs***

Number 3: Some family members may be late but have a child alive. Result of research on such stored bio sample may be of use to help the child. I do not know how the storing procedure is.

1. ***Moderator: Can you explain what you understand by precision medicine? Are you aware of precision medicine? Can it be applied to stroke disease? What is your source of information?***

Number 1: I have heard about it on the Kwara and NTA TV during health programs.

***Moderator: What do you think about precision medicine? What you think are the benefits or disadvantages? Is it important in Africa?***

Number 1: An advantage to the patient is the bond it creates between the patient and the care giver. It creates room for privacy.

Number 6: This precision medicine will not give chance for trial and error. It will bring about what works for the individual, not everyone.

Number 2: I believe it is good for research purpose. This is the first time I am hearing of this.

Number 4: I have not heard of it.

Number 5: I don’t know anything about it.

Number 1: It gives room for privacy.

1. ***Moderator: What do you understand by brain donation for research purpose? What is our personal willingness to donate? What are the cultural, social and religious beliefs on donating brain for research purpose?***

[Giggles by Numbers]

Number 2: I have seen it in movies and heard about it. They call them organs donors. I do not think I can donate my brain. I believe it is a personal thing, I do not think there is any religious issue on it. Family and peers cannot influence it.

Number 6: Religiously (Islam). The body is sacred and it cannot be given to another. I am not willing to donate. Islam does not support opening or desecrating a dead body.

Number 7: No, I can’t donate my brain for research purposes.

Number 3: People will not support it. I cannot do it. I am not sure of religious (Islam) position of it.

Number 4: (Shakes head in disgust) It is not good at all. I have never heard of it. It is unacceptable culturally. Culturally, socially and religiously, it is not tenable.

Number 1: I am Christian. I do not support it. When one gets to heaven and is asked of the brain, where will get the missing part, what will be it response? It is not right, (Laughter by the group), the brain is vital.

Number 6: Islam does not support. The body should be buried intact.

***Moderator: Can we think of any benefit of brain donation?***

Number 7: I believe the brain also dies when the person dies so of what use is it. Investigation should be done when one is alive. I have never heard of such and I would not donate my brain.

Number 3: As you are mentioning it now, it looks like a good idea too it will add to the body of knowledge but our culture and religion will not support it, if the facilities are available, it can be practiced.

***Moderator: What may encourage this practice?***

Number 1: I think if health education is done to create awareness on brain donation and the benefits to the future generation, people might be interested; when a few people indicate interest, others may follow suit. It should be from grass roots via communal gatherings, religious gatherings. It is people that practice religion, these people should be educated on the fact that all the vaccines we have access to now emanated from some people who donate themselves for the research in the past so we have to contribute our quota. Now I have just been enlightened and I think I can donate to benefit humanity. If I do so, some people may gossip about my family having buried me without my brain, but then I know the reason I took that decision. My decision can influence my children to do same.

Number 6: If people are aware of the benefits, they might be interested.

1. ***Moderator: What do you understand by blood sample donation for genetic research? What are your thoughts on blood donation for research? Uses of blood sample donated for research.***

Number 5: It is very good as it will be of benefit to future generation in turns of prevention. ***Moderator: Sources of information?***

Number 5: I have heard about it from sources in my village, Kogi State.

***Moderator:*** ***Cultural, social and religious belief on donating blood for genetic research***

Number 5: I don’t think it is influenced by social, culture or religious factor.

Number 7: It can help one to be more aware on other diseases in the community, for example a geographical region like West Africa. It may be reveal a disease within the blood, for instance, a communicable disease.

***Moderator: In this context, it is for*** ***genetic research, not communicable disease.***

Number 7: I do not know.

Number 1: I think religion or culture is against genetic research. Religion is on one side, health is on another side.

Number 6: culturally and in religion, we believe that some illness seen in families. Religion is not against it. There is a saying from the prophet that suggests “that blood walks/moves”. In those days families are investigated (by asking from people who know the family of the prospective spouse e.g epilepsy) for disease before the guardians consent to their wards getting married.

Number 4: No religion is against blood donation.

***Moderator: Awareness of any policy or law guiding blood sample donation of research and storage***

Number 1: The law I am aware of is that there should be confidentiality between the Number and the health institution. Whatever information that gotten from such research, is for the consumption of the two parties only.

Number 2: I think a contract should be signed between the person and facility.

1. ***Moderator: Share with us your opinion and thoughts about blood sample donation for stroke genetic research. Are you willingness to be involved in such research? What can you say about your family member or members of your community willingness to give blood sample for stroke genetic research? What do you see as the barrier that could hinder your donation of blood sample for stroke genetic research? What do you perceive as benefit(s) of giving blood sample for stroke genetic research that could promote your willingness to donate? What could be done to make you and more people give blood sample for research?***

Number 4: I am willing to donate blood for stroke research. My family members will also be willing. I will donate as it will be beneficial.

Number 3: I will be willing as well as my family members too. If people are aware of the purpose, they will accept to it. However people may not be willing because of fear of incidental findings, for example HIV.

Number 2: people may be scared of the outcome as people believe that what you do not know will not kill you. I believe the way forward in this case is to educate people on the preventive measures for the disease.

Number 7: I will be willing to donate, my family members will also be favourably disposed if they are made aware. A disadvantage is the fear that people have for the unknown. If their one’s father is diagnosed of having stroke, one would be scared of been aware of one predisposition of having same.

Number 6: I will be willing to donate blood for research. There’s nothing to discourage me. Benefits will be promoting factor.

Number 3: There are so many issues in the society and now especially money rituals, this might discourage people from donating.

Number 1: Some religion supports it.

1. ***Moderator: Tell us what you know about informed consent?***

Number 1: I know it happens if the doctors and nurses want to conduct practical on patients. They ask for the patient to co-operate with them in conducting the investigation. There should be agreement between the two parties (researcher and Number), most importantly there should be privacy (confidentiality), my information should not be for public consumption.

Number 4: I don’t really know about informed consent.

***[Moderator explains informed consent and the 4 types, Numbers nod in understanding]. Which is your preferred type and why? Who will you like to be involved before you participate? Will you consent to your data to be used in the incident of death and why? Do you support generic consent for community?***

Number 1: I prefer broad consent as I don’t mind. People have volunteered for research in the past that result in knowledge that is beneficial to my own health so I would like to contribute to humanity. I don’t want others involved in the process of obtaining consent, it is a personal thing. In case of death, I want research to be carried on with the data even after death. I’m in support of communal consent.

Number 2: It depends, I prefer broad consent in terms of blood donation but in terms of another thing I prefer dynamic. I believe whatever research you are conducting is to benefit myself and others. I cannot speak for others so others should not speak for me; I think it is an individual thing. Data can be used in the event of death if it involves blood. I do not support communal consent.

Number 3: I prefer broad consent; I want my husband and children to be involved. I’m in support of communal consent as it will benefit the group and I’m okay with data being used after death.

Number 4: I prefer broad. I want my family involved. In the event of death my data should still be used for research purposes. I am not in support of communal consent.

Number 5: I prefer restricted because I want my privacy and I want to be fully involved. When you are using it for multiple studies, I will be anxious about the results for many test so I will like to be spared the distress. I want my wife and children involved. In the case of death, I don’t want my data to be used. I’m not in support of communal consent.

Number 6: I prefer broad consent. No need to involve others. Data can be used in the event of death. I’m am not in support of communal consent.

Number 7: I prefer dynamic consent. My wife and the children should be involved. In the event of death, data can be used to help others. I’m not in support of communal consent.

1. ***Moderator: What is your opinion on storage of blood and blood fractions for genetic research?***

Number 4: I don’t think it’s possible to do that.

Number 1: It’s a very good thing as upcoming medical expert can have access to it and do new research in the future.

Number 2: I don’t think anyone who donates blood would mind. I think it’s a good thing and it would solve some issues in the future. I feel

Number 7: It is good and it would lead to progress or advancement in research.

1. ***Moderator: Tell us what you think about sharing of data, blood/blood fraction, brain images (CT/MRI) as well as brain tissue samples***.

Number 1: It’s just an exchange of medical idea/report/research, I think it is good. Research can be done in collaboration. A researcher in Ghana need not come over here before having access to such data or samples. Data may be shared via the internet. Research on races may be compared. It prevents the research being done over again. It is good to share data.

Number 2: It will enable a more efficient search work as more discoveries can be made.

Number 6: It is good to share it.

Number 4: It is good to share it.

***Moderator: what do you think about the commercial use of stored data, blood/blood fractions, brain images and brain tissue?***

Number 1: If the research is going to be commercialized, royalty must be paid to the Numbers/family.

Number 3: I’m indifferent as regards the commercialization.

1. ***Moderator: What are the ways you think one can receive the results of genetic research? Do you want feedback of research results and incidental findings? What are the challenges of returning individual results? Which ways will you prefer to get the feedback? Are there ethical, legal or social issues relating to returning such results?***

Number 1: I want feedback as well as the incidental findings. Channels for feedback include phone calls, written but I prefer a written hard copy of the result which can be delivered via mail or made available for pick by me. I don’t think there is any legal or ethical issue.

Number 3: I want feedback as well as incidental findings. I prefer a face-to-face verbal feedback. Feedback can also be written. I’m not sure anything ethical/social can affect it.

Number 2: I would love a feedback on the research as well as incidental findings. I prefer an email to be sent. An ethical issue is having who to contact in case there are enquiries.

Number 5: I will prefer one-on-one feedback, though documented evidence is also good. I don’t want feedback on incidental findings.

Number 7: I prefer one-on-one feedback.

Number 6: I prefer one-on-one feedback. I’m also interested in incidental findings.

Number 4: I prefer one-on-one feedback in person. I want to be informed on incidental findings.

1. ***Moderator: How much control should an individual have over their biological specimen, as regards use in research? What rights should the donors have over their specimen, how they are use in research, and over any profits made from the research discoveries arising therein?***

Number 1: I feel the Number should relinquish his rights to the researcher but have 25% right in finances.

Number 2: The Number should have full right and be able to specify what he/she wants it to be done with the specimen.

Number 5: The Number should have 50% right on it, he/she should be informed on anything to be done on it.

(Adigun et al., 2022)Number 3: The financial right should be 50-50.

***Moderator: How should autonomy rights be balanced against societal benefits as pertain to the use of human specimen in specimen?***

Number 1: For humanity sake, societal benefit should supersede personal right.

Number 5: Personal right should be 30% and societal benefit 70%.

Number 4: Societal benefit is more than personal right.

1. ***Moderator: What is your opinion about governance and regulation of bio-banking? Is there a needed for ethical committee approval on future use of stored data, blood or brain tissue resource for research? Is there a need to set up a regulatory board?***

Number 1: It is a good move. It will prevent arbitrary use of the tissues/material collected from the patients.

Number 3: The right of the individual will not be denied if there is a governing body.

Number 2: The governing body will enlighten Numbers on their rights and attend to their complaints. It’s a good idea.

Number 7: It’s good.

1. ***Moderator: Explain possible interventions for implementation of bio-banking. What suggestions do you have that can help to raise awareness and improve attitude towards blood sample or brain donation for research and encourage people to adopt the practice?***

Number 3: Sensitization program on Radio and TV.

Number 5: Organization of seminars in public areas. Use of social media, to create awareness on the issue.

Number 1: The press should be involved; theatre art can stage short plays on the advantages of bio-banking. People believe in seeing/watching, visual things rather than reading. It should be added to our education curriculum it will create enlightenment.

Number 4: Create sensitization and awareness on bio-banking by reaching out to many people, just as we are presently doing, to find out their opinion and ideas.

1. ***Moderator: Do you have other major concern or recommendation on use of blood or brain tissue for research in Nigeria?***

Number 1: The government should empower the health workers to be able to carry out bio-banking to advance research and break new grounds.

Number 5: Health works should be equipped to carry out these researches. Nigerians are doing a lot of research abroad because they have access to facilities to do so. If such facilities are available locally, it will reduce the brain drainage and encourage some to return home.

Number 2: People have an issue on what is in it for me. If they are given incentives, they would be more willing. These incentives are best if they are financial. Also promise of financial gain if the research proves profitable.

Number 1: Another incentive is the assurance of free treatment diseases are picked as a result of the research.

Number 3: Compensation in terms of money would really help, in this environment, even N20, 000 or N10, 000 for blood sample but depends on the person.

Number 1: For brain donation about a million naira incentive.

Number 6: Brain donation, even 10 million naira is okay.

(General laughter)

***Moderator : Thank you for your time***.
