# Supplementary material for: Research participants’ perception of ethical issues in stroke genomics and neurobiobanking research in Africa
Source: PLoS One. 2025 May 6;20(5):e0292906. doi: 10.1371/journal.pone.0292906 (PMC12054916; doi:10.1371/journal.pone.0292906)
Supplement: S3 File — (ZIP) [file pone.0292906.s003.zip › Files for PLOS ONE - updated March 2025/Kano _SIREN Stroke Free Controls_FGD.docx]

KANO SITE

TRANSCRIPTION AND TRANSLATION OF FOCUS GROUP DISCUSSION CONDUCTED DURING DATA COLLECTION ON AFRICAN NEUROBIOBANK FOR PRECISION STROKE MEDICINE - ETHICAL, LEGAL, AND SOCIAL IMPLICATIONS (ELSI) PROJECT

Type/Group: Focus Group Discussion.

Respondents: Stroke Free Control.

Moderator: AM

Note taker: ZS.

Transcriber/Translator: Abdulmajid Sani.

Date: 18/8/2019. Time Start: 8:45am. Time Ended: 10: 15am.

INTRODUCTION:

Good day. You are welcome. I want to thank you for coming today. My name is AM and I will be the facilitator for today’s group discussion. I am a staff of Aminu Kano Teaching Hospital and I work for Kano site on ELSI and SIBS project. We also have ZS present to take notes for us.

We are conducting a study among people who have had a stroke, their care givers and other people in the community to identify and look critically at ethical, legal and social issues relating to stroke biobanking in the African context. Therefore we will be asking and discussing issues such as your knowledge, attitude, perceptions, barriers and facilitators influencing ethical, legal and social issues related to the use of blood and stored blood fractions, brain images (CT/MRI) and brain donation in the context of stroke genomic research.

We invited you to take part in this discussion today because we believe that you have one important thing or the other to share with us on issues related to ethical, legal and social issues relating to stroke biobanking in the African context. We would like your suggestions on how to improve on the tools so that they can be clearer and more appropriate.

Whatever we learn from today’s discussion will help us develop intervention program to address the ELSI issues related to stroke genomic and biobanking research in Sub Saharan Africa

Before we begin, I would like to review a few ground rules for the discussion. I will ask you several questions and I’d like to give everyone a chance to give their opinions. We do not have to go in any particular order but we do want everyone to take part in the discussion. We ask that only one person speak at a time.

I am interested in your opinions and whatever you have to say is fine with us. There is no right or wrong answers. I am just asking for your opinions and suggestions. I am here to learn from you. Don’t worry about having different opinions from someone else. But please do respect each other’s answers or opinions.

You may choose not to answer any question you do not wish to.

Feel free to treat this as a discussion and to ask questions of each other and to respond to what others are saying, whether you agree or disagree.

I will treat your answers as confidential. I will not to ask for anything that could suggest your identity. I will only use first names during the discussion. I also ask that each of you respect the privacy of everyone in the room and not to say or repeat what is said here in any way that could identify anyone in this room.

I am recording the discussion today on an audio because we don’t want to miss any of your suggestions. However, once we start the audio recorder we will not use anyone’s full name and we ask that you do the same.

Finally, this discussion will last for about 1 hour and we request that you stay for the entire meeting.

Does anyone have any questions before we start?

Respondents: Yes, we have agreed, we don’t have any question, we can start.

Moderator. We want to start by asking you to tell us what you know about genetic research.

Resp. 8. Genetic research is an activity or investigation on human beings that involved using parts of the body or something from the body, e.g human lungs, which is responsible for filtering the Air we inhale.

Resp. 2. Genetic research is an investigation that concerns inheritance of the family.

Moderator: Can you explain what you understand by biobanking?

Resp. 1. Biobanking is keeping of something that concerns human beings; e.g blood and Kidney are kept for future use.

Resp. 7. Yes, as the last person said, is keeping of something to be used in future, e.g Eye is also taken and kept for future use in the hospital.

Resp. 4. Keeping od something to be used in future, especially things that concerns health.

Moderator: Can you explain what you understand by precision medicine?

Resp. 4. Is something that concerns medicine or drugs, e.g drugs or medicine curing diseases or illness like antiretroviral drugs for HIV.

Resp. 1. It is something that concern medicines e.g ACT for Malaria treatment.

Resp. 5. Yes, as he said, some drugs for headache like Sudrex to cure headache.

Resp. 8. Yes, as my colleagues said, like Antihypertensive drugs for hypertensions, they are good and active on the disease.

Moderator: What do you understand by brain donation for research purpose?

Resp. 1. I have heard of it, but don’t know what it entails, but is giving of the brain.

Resp. 8. Yes, have heard of brain test, because we once went to Sokoto to see a Consultant specialist on brain from Kano call Neurologist, but we were un able to see him, he went out of town to see other patients.

Resp. 4. I have heard of it but don’t know what it means, but to my own understanding is giving the brain for investigation in the hospital.

Moderator: What do you understand by blood sample donation for stroke genetic research?

Resp. 6. It means donation of blood for research, the benefits are; to diagnose a disease and make a further research on the medication to be used against the disease.

Resp. 8. It is giving of blood to identify a specific disease for the purpose of knowing the medicine.

Moderator: Share with us your opinion and thoughts about blood sample donation for stroke genetic research

Resp. 1. My opinion and thoughts are it is good to give blood for research, even me I can donate my blood for the research purpose especially stroke research.

Resp. 7. It is very good to give blood for research especially stroke because now is becoming common in our societies, it will add knowledge.

Resp. 2. As my colleagues mentioned, it is very good to donate blood for research it will help in knowing who has the disease.

Moderator: Tell us what you know about informed consent.

Resp. 4. Anybody who is to give blood as I have seen has to agree before taken his blood sample for research.

Resp. 5. If researchers come from outside the country, they have to contact the Government of the country for approval to do research.

Resp. 8. Any person or his relatives to be involved in the research has to agree and sign a form before undergoing any activity or surgery.

Moderator: What is your opinion on storage of blood sample and blood fractions?

Resp. 2. Storage of blood sample for genetic research is very good, it through that they will find any new issue concerning the sickness.

Resp. 1. It is good to store blood sample, it can be used later to make investigations on the disease.

Resp. 4. It is something very good; it can be used for future investigations. That is why even the USA set aside a day for donation of blood.

Moderator: Tell us what you know about sharing of data, blood/blood fractions, brain images (CT scan/MRI) as well as brain tissue samples

Resp. 6. Sharing of data and others like blood, CT scan and MRI and results is very good, as it provides good knowledge and effective communication between people to widen their experiences.

Resp. 8. It is good to share data and other results concerning researches and the researchers, it will increase their knowledge of treatments on diseases especially genetic research.

Moderator: Share with us your thoughts about return of individual research results and incidental findings

Resp. 4. It is also very good to give or return results to anybody that his sample was collected; it increases their confidence on the aspects of research.

Resp. 1. Yes, as said, it is a good practice to return results to the participants to the findings on the research, there is need to return the results through their contacts i.e phone numbers to call them to avoid missing of the results.

Resp. 5. There is need to return the results, but that has to be hand to hand, it is more better to do that.

Resp. 6. Yes, is good to return the results, but the medical doctors are the best to give the results.

Resp. 2. It is good to return the results, but to be given to the relatives.

Resp. 7. Returning results to participants is very good, it has to be through the medical doctor or the researchers, because they were trained on how to give it without any problem in case there is issues with results.

Moderator: Explain your understanding of Biorights.

Resp. 5. The participants has no right or control over the sample or anything given, because he/she was consented before taken the sample.

Resp. 2. A participant has the right over the samples taken or collected from him, even though he gave consent before taken.

Resp. 1. The participants has no right , that is why a consent form was given to him or her to agree and sign before taking the samples.

Resp. 6. As said, the participants has no right, that was is consent form was given.

Moderator: What is your opinion about governance and regulation of biobanking?

Resp. 4. There is need for ethical approval which is part of the regulations of biobanking.

Resp. 7. As the last speaker said, there is need for governance and regulations on that, if not people will be doing what they feels like doing in the name of research.

Resp. 5. There is much need for the government to come in and set rules on the issues of research especially that of human beings.

Moderator: Explain possible intervention for implementation of biobanking.

Resp. 3. There should be programmes that will inform and creates awareness to people on the issues of biobanking, because is something very new to us in this side of the country.

Resp. 4. The media e.g Television, Radio, etc, has to be used to create awareness among the public.

Resp. 8. There is need to involvement of traditional leaders and religious leaders on the sensitization of people especially at the rural areas on the issues of biobanking.

Resp. 7. Involvement and use of religious schoolars to create awareness among people on the issue of biobanking in the communities.

Resp. 1. The use of social medias, e.g Radio, Television, etc.

Moderator: Any other major concern or recommendation on use of blood or brain tissue for research in Nigeria

Resp. 3. There is need to inform people on the issues concerning brain tissue for research especially in the Northern part of the country because of the level of literacy. It is something very strange to them.

Moderator; Thank you for the time and responses, we want to seek for your consent again we have a brief written survey for just about 5 minutes.
