# Supplementary material for: Research participants’ perception of ethical issues in stroke genomics and neurobiobanking research in Africa
Source: PLoS One. 2025 May 6;20(5):e0292906. doi: 10.1371/journal.pone.0292906 (PMC12054916; doi:10.1371/journal.pone.0292906)
Supplement: S3 File — (ZIP) [file pone.0292906.s003.zip › Files for PLOS ONE - updated March 2025/Kano_ Caregivers_FGD.docx]

KANO SITE

TRANSCRIPTION AND TRANSLATION OF FOCUS GROUP DISCUSSION CONDUCTED DURING DATA COLLECTION ON AFRICAN NEUROBIOBANK FOR PRECISION STROKE MEDICINE - ETHICAL, LEGAL, AND SOCIAL IMPLICATIONS (ELSI) PROJECT

Type/Group: Focus Group Discussion.

Respondents: Care Givers of Stroke Patients.

Moderator: AM

Note taker: ZS

Date : 18/8/2019. Time Start: 10:20am. Time Ended: 11:45am

INTRODUCTION:

Good day. You are welcome. I want to thank you for coming today. My name is AM and I will be the facilitator for today’s group discussion. I am a staff of Aminu Kano Teaching Hospital and I work for Kano site on ELSI and SIBS project. We also have ZS present to take notes for us.

We are conducting a study among people who have had a stroke, their care givers and other people in the community to identify and look critically at ethical, legal and social issues relating to stroke biobanking in the African context. Therefore we will be asking and discussing issues such as your knowledge, attitude, perceptions, barriers and facilitators influencing ethical, legal and social issues related to the use of blood and stored blood fractions, brain images (CT/MRI) and brain donation in the context of stroke genomic research.

We invited you to take part in this discussion today because we believe that you have one important thing or the other to share with us on issues related to ethical, legal and social issues relating to stroke biobanking in the African context. We would like your suggestions on how to improve on the tools so that they can be clearer and more appropriate.

Whatever we learn from today’s discussion will help us develop intervention program to address the ELSI issues related to stroke genomic and biobanking research in Sub Saharan Africa

Before we begin, I would like to review a few ground rules for the discussion. I will ask you several questions and I’d like to give everyone a chance to give their opinions. We do not have to go in any particular order but we do want everyone to take part in the discussion. We ask that only one person speak at a time.

I am interested in your opinions and whatever you have to say is fine with us. There is no right or wrong answers. I am just asking for your opinions and suggestions. I am here to learn from you. Don’t worry about having different opinions from someone else. But please do respect each other’s answers or opinions.

You may choose not to answer any question you do not wish to.

Feel free to treat this as a discussion and to ask questions of each other and to respond to what others are saying, whether you agree or disagree.

I will treat your answers as confidential. I will not ask for anything that could suggest your identity. I will only use first names during the discussion. I also ask that each of you respect the privacy of everyone in the room and not to say or repeat what is said here in any way that could identify anyone in this room.

I am recording the discussion today on an audio because we don’t want to miss any of your suggestions. However, once we start the audio recorder we will not use anyone’s full name and we ask that you do the same.

Finally, this discussion will last for about 1 hour and we request that you stay for the entire meeting.

Does anyone have any questions before we start?

Respondents: Yes, we have agreed, we don’t have any question, we can start.

Moderator. We want to start by asking you to tell us what you know about genetic research.

Resp. 5. Genetic research is an investigation that characterizes inheritance from parents. E,g inherited diseases like mental illness. While research referred to as the process of investigation or finding out about a problem.

Resp.6. Diabetes and hypertension are a disease condition that flows and runs in a family, and this could be identified through research.

**Moderator: Can you explain what you understand by biobanking?**

Resp. 7. Biobanking is taking of sample from someone for future research. I could remember that, Knee bone was taken from one patient that at the hospital sometimes ago for research purpose.

Resp. 2. I have heard and aware that some part of human beings used to be taken e.g Kidney, blood, heart can be taken for the purpose of research.

Resp. 1. I am not aware that part of human being can be taken for any research, but I am aware that blood can be taken as sample find out if one is at risk of developing stroke in the future or not.

Resp. 4. I am aware that blood and tissue can be taken and stored for biobanking.

**Moderator: Can you explain what you understand by precision medicine?**

Resp. 6. Precision medicine is said to be a condition that one has an illness e.g facial stroke or urine incontinence, he was placed on medication and his condition improved within given time.

Resp. 9. As he said or mentioned, also, one of my relatives developed stroke and was taken to hospital, feels better after taken the prescribed by the doctor.

Resp. 3. Yes, I could remember, my brother sometime suffers Hiccup (SHAKUWA), he was taken to hospital, he was ok immediately.

Moderator: What do you understand by brain donation for research purpose?

Resp. 2. I have not heard of donation of brain or its part for research purpose.

Resp. 4. I am not aware of brain donation for research purpose.

Resp. 6. I am not aware also about brain donation for research purpose.

Resp. 8. I am not aware of brain donation for research purpose.

Moderator: What do you understand by blood sample donation for stroke genetic research?

Resp. 5. Blood donation for stroke genetic research is donation of blood for the purpose of finding out about the DNA of someone. E.g, if there is arguments about pregnancy; blood sample can be taken from both the man and the woman to determine the gene (DNA).

Resp. 8. Is giving of blood sample for the purpose of testing to know if one has the risk of having stroke or not.

Resp. 10. given of blood sample for testing to know if they will have stroke in future.

Moderator: Share with us your opinion and thoughts about blood sample donation for stroke genetic research

Resp. 6. My opinion is it is very important to donate blood for stroke genetic disease research.

Resp. 8. It is good to donate blood for stroke genetic research, but not all people agreed to donate.

Resp. 4. My opinion is that is good, but we are not good in doing the habit of donating for research purpose.

Moderator: Tell us what you know about informed consent.

Resp. 3. Sure, it is good to obtain consent, but not all people agreed do that, because of ignorance.

Resp. 5. Yes, consent is required, is to make sure that things are done according to the rules and guides.

Resp. 8. Consent should be obtained from the participants, if they are well informed, he can agree to give his sample or blood.

Resp. 9. There is need for informed consent but the health provider has to establish a good relationship with the participants.

Moderator: What is your opinion on storage of blood sample and blood fractions?

Resp. 2. Storage of blood sample or fraction is very good, it is through that future investigations or researches are conducted to know about the diseases and the new innovations on treatments and others.

Resp. 1. It is good to store blood or part of the blood for the purpose of use in later period.

Resp. 5. Storage of blood is very important, is for future use. Also research is the best way towards an effective treatment and prevention of diseases.

Moderator: Tell us what you know about sharing of data, blood/blood fractions, brain images (CT scan/MRI) as well as brain tissue samples

Resp. 4. Sharing of data and other findings concerning participants is very good, it is through that the health providers updates their knowledge and skills to treat patients.

Resp. 3. Yes, it is sharing of ideas on research or any findings that is conducted among the people, it gives room for researchers to get the current ideas on treating patients.

Resp. 5. It is good to share ideas with other people within or outside Nigeria. It improves good practice.

Moderator: Share with us your thoughts about return of individual research results and incidental findings

Resp. 7. Returning of individual results is very good, and is to be given through the researchers.

Resp. 1. The results should be returned or given to the participants verbally and followed with explanation by the health provider to the parents.

Resp. 5. The results are to be given through text messages.

Moderator: Explain your understanding of Biorights.

Resp. 9. Is having right over given sample e.g blood, or any other thing that researchers collect or use during researches. The participant has no right over what he donated.

Resp. 6. The participant has no right on the sample donated for research, because he has already given consent before given the sample for research.

Resp. 10. Yes, as others have said, the participant has no right over the sample donated for research, he/she was consented before taking the sample.

Resp. 5. The participant has no right over what he has donated, consent was obtained from him before he agreed to give the sample.

Moderator: What is your opinion about governance and regulation of biobanking?

**Resp. 3. My opinion is that there should be rules and regulations governing the biobanking, to avoid other people using the opportunity for rituals with the samples taken for the biobanking.**

**Resp. 2. Yes, I agreed with what my collequa said, there should be strong rules and regulations and all the agencies must be involved in the governing of biobanking.**

**Resp. 8. I strongly agreed, there should be rules and even Laws to govern the biobanking process and the use, rituals exist in many places. It is by this the act will be more acceptable especially in the Northern part of the country.**

Moderator: Explain possible intervention for implementation of biobanking.

Resp. 1. The possible intervention for the implementation of biobanking is through awareness creation using the media (Radio, Television etc).

Resp. 9. The use of health practioners or providers to inform people in the facilities.

Resp. 10. The use of religious leaders and traditional leaders in the communities.

Resp. 4. The use of town announcer and production of leaflets, posters etc.

Moderator: Any other major concern or recommendation on use of blood or brain tissue for research in Nigeria

Resp. 3. There is need to great different means of making people aware about use of blood or brain tissues for research, especially in the Northern part of the country, brain donation is something very strange and couple with ignorance.

Moderator; Thank you for the time and responses, we want to seek for your consent again we have a brief written survey for just about 5 minutes.
