# Supplementary material for: Research participants’ perception of ethical issues in stroke genomics and neurobiobanking research in Africa
Source: PLoS One. 2025 May 6;20(5):e0292906. doi: 10.1371/journal.pone.0292906 (PMC12054916; doi:10.1371/journal.pone.0292906)
Supplement: S3 File — (ZIP) [file pone.0292906.s003.zip › Files for PLOS ONE - updated March 2025/Kano_ Community Advisory Board_FGD.docx]

KANO SITE

TRANSCRIPTION AND TRANSLATION OF FOCUS GROUP DISCUSSION CONDUCTED DURING DATA COLLECTION ON AFRICAN NEUROBIOBANK FOR PRECISION STROKE MEDICINE - ETHICAL, LEGAL, AND SOCIAL IMPLICATIONS (ELSI) PROJECT

Type/Group: Focus Group Discussion.

Respondents: Community Advisory Board Members.

Moderator: AM

Note taker: ZS

Date: 19/8/2019. Time Start: 9:25am. Time Ended: 10:40am.

INTRODUCTION:

Good day. You are welcome. I want to thank you for coming today. My name is AM and I will be the facilitator for today’s group discussion. We also have ZS present to take notes for us.

We are conducting a study among people who have had a stroke, their care givers and other people in the community to identify and look critically at ethical, legal and social issues relating to stroke biobanking in the African context. Therefore we will be asking and discussing issues such as your knowledge, attitude, perceptions, barriers and facilitators influencing ethical, legal and social issues related to the use of blood and stored blood fractions, brain images (CT/MRI) and brain donation in the context of stroke genomic research.

We invited you to take part in this discussion today because we believe that you have one important thing or the other to share with us on issues related to ethical, legal and social issues relating to stroke biobanking in the African context. We would like your suggestions on how to improve on the tools so that they can be clearer and more appropriate.

Whatever we learn from today’s discussion will help us develop intervention program to address the ELSI issues related to stroke genomic and biobanking research in Sub Saharan Africa

Moderator. We want to start by asking you to tell us what you know about genetic research.

Resp. 1. Genetic research based on my understanding is taking sample like blood from human beings in order to test and diagnose or find if there is any disease or sickness on it. E.g Diabetes, sickle cell.

Resp. 7. Yes, as he said, is testing of anything taken from human beings to test for something like gene or disease, e.g sperm is taken and test to identify the Sex.

Resp. 4. Genetic research is investigations carried out in order to find out about something, e.g skeleton is used as specimen to carry out a research.

Resp. 2. Genetic research is taking of sample like spinal cord to know whether the patient has epilepsy or not, is done in the laboratory in the hospital.

Moderator: Can you explain what you understand by biobanking?

Resp. 1. Biobanking is keeping of something like blood from human for the purpose of diagnosing a disease for example in some countries heart is taken as a sample for biobanking and disease investigations.

Resp. 2. It is taking sample like blood, for the purpose of doing investigations, without investigations, success cannot be achieved, and therefore blood samples and others are taking for investigations.

Resp. 4. Some donates their kidney for some people to help save their lives; on the other hand it can be used for biobanking.

Resp. 7. We have been hearing about biobanking, e.g about taking a sample (tissues) for cancer and brain investigations.

Resp. 8. When there is growth on skin, the sample or part is taken to the tertiary hospital for test.

Moderator: Can you explain what you understand by precision medicine?

Resp. 5. I understand that if someone is having illness e.g malaria, the patient is placed on Arthemether injection, and he or she will feel better within a short period of time.

Resp. 2. It means identifying a disease and the type of drug or medicine that will directly cure the disease.

Resp. 3. Having or suffering from an illness and the drugs that acts immediately on the illness or disease. E.g hypertension is treated with some drugs i.e injections and respond quickly after starting the treatment.

Moderator: What do you understand by brain donation for research purpose?

Resp. 4. I am not aware about brain donation for research. Even most of the people I knew, they are not aware about that. Reason is ignorance, culture and some is religious beliefs.

Resp. 1. I am not aware about brain donation, most of the people I relates with are not aware about that, ignorance and religious are the key barriers.

Resp. 6. I don’t have any knowledge on brain donation, reasons are: inadequate equipment and lack of experts to carry out the activities in most of our communities.

Moderator: What do you understand by blood sample donation for stroke genetic research?

Resp. 5. It means donation of blood for research to help people know about their different types of diseases.

Resp. 4. People donates blood for genetic research, due to the increase in awareness by some members from the community.

Resp. 8. It is donation of blood samples voluntarily by for genetic research for students to get more knowledge.

Moderator: Share with us your opinion and thoughts about blood sample donation for stroke genetic research

Resp. 7. It is something worth doing to donate blood for genetic research.

Resp. 4. It is good to donate blood for genetic research., it is through that the young students will get more knowledge.,

Resp. 1. Donation of blood is very good, people donates blood free of charge for the purpose of research to diagnose such as disease as strokes.

Moderator: Tell us what you know about informed consent.

Resp. 2. It is a form that is giving or issued to the participants or anybody who wants to be enrolled in a research.

Resp. 8. It is a documents provided by the researchers to the participants to understand what the research is all about, and to get his willing to take part in the research or study.

Moderator: What is your opinion on storage of blood sample and blood fractions?

Resp. 7. It is good to store blood sample or part, it can be used to teach students to know about some diseases.

Resp. 1. It is good to store blood samples or any part taken as sample to increase knowledge about that.

Moderator: Tell us what you know about sharing of data, blood/blood fractions, brain images (CT scan/MRI) as well as brain tissue samples

Resp. 6. It is very good to share the findings as it will add knowledge and will increase type of care among the providers.

Resp. 3. Sharing of data or results of research to others, will help in increasing the knowledge of people.

Moderator: Share with us your thoughts about return of individual research results and incidental findings

Resp. 4. Every participant that give sample, will need to know his results, as a form of feedback, this researchers or health providers are the best to give the results.

Resp. 2. Results has to be given to the participants, but it has to be done by a trained health researcher or provider.

Resp. 6. The there is need to be abiding the rules and confidentiality has to be followed.

Moderator: Explain your understanding of Biorights.

Resp.3. Yes, the participant has the right over what he gave as specimen for research, idpite he gave consent.

Resp. 7. To me, participant has no write or control over what he has already signed a consent form and already he has no any knowledge on what type of test will be conducted with the sample.

Resp. 5. To me, participant has no write or control over what he has already signed a consent form and already he has no any knowledge on what type of test will be conducted with the sample.

Moderator: What is your opinion about governance and regulation of biobanking?

Resp. 7. There should be governance and regulations on biobanking to guide on how the activity will be conducted.

Resp. 1. Yes, there are rules and regulations for taking blood, and any other sample that can be used.

Moderator: Explain possible intervention for implementation of biobanking.

Resp. 6. There should be mobilization of the people especially those in the rural areas.

Resp. 3. There should also be use of media to mobilize people to become more aware on the process of biobanking.

Resp. 2. There is need to use the media houses in implementation of biobanking in our community.

Resp. 8. There is need to advocate at different level, the religious and youth groups.

Resp. 5. Health providers some should educate participants and the public on the importance of biobanking.

Resp.4. There is need involve every importance person e.g sensitization, involving traditional leaders, provision of incentives to the participants, o matter little it is.

Moderator: Any other major concern or recommendation on use of blood or brain tissue for research in Nigeria

Resp. 1. There is need to enlighten, mobilize and create awareness and involve the religious and community leaders on the introduction of biobanking to the community.

Moderator; Thank you for the time and responses, we want to seek for your consent again we have a brief written survey for just about 5 minutes.
