# Supplementary material for: Research participants’ perception of ethical issues in stroke genomics and neurobiobanking research in Africa
Source: PLoS One. 2025 May 6;20(5):e0292906. doi: 10.1371/journal.pone.0292906 (PMC12054916; doi:10.1371/journal.pone.0292906)
Supplement: S3 File — (ZIP) [file pone.0292906.s003.zip › Files for PLOS ONE - updated March 2025/Kano_SIREN Stroke Cases_FGD.docx]

KANO SITE

TRANSCRIPTION AND TRANSLATION OF FOCUS GROUP DISCUSSION CONDUCTED DURING DATA COLLECTION ON AFRICAN NEUROBIOBANK FOR PRECISION STROKE MEDICINE - ETHICAL, LEGAL, AND SOCIAL IMPLICATIONS (ELSI) PROJECT

Type/Group: Focus Group Discussion.

Respondents: Stroke Patients.

Moderator: AM

Note taker: ZS

Venue of interview: Village heads place, Nasarawa, Kano State.

Date: 17/8/2019. Time Start: 11:25am. Time Ended: 12: 33pm.

INTRODUCTION:

Good day. You are welcome. I want to thank you for coming today. My name is AM and I will be the facilitator for today’s group discussion. I am a staff of Aminu Kano Teaching Hospital and I work for Kano site on ELSI and SIBS project. We also have ZS present to take notes for us.

We are conducting a study among people who have had a stroke, their care givers and other people in the community to identify and look critically at ethical, legal and social issues relating to stroke biobanking in the African context. Therefore we will be asking and discussing issues such as your knowledge, attitude, perceptions, barriers and facilitators influencing ethical, legal and social issues related to the use of blood and stored blood fractions, brain images (CT/MRI) and brain donation in the context of stroke genomic research.

We invited you to take part in this discussion today because we believe that you have one important thing or the other to share with us on issues related to ethical, legal and social issues relating to stroke biobanking in the African context. We would like your suggestions on how to improve on the tools so that they can be clearer and more appropriate.

Whatever we learn from today’s discussion will help us develop intervention program to address the ELSI issues related to stroke genomic and biobanking research in Sub Saharan Africa

Respondents: Yes, we have agreed, we don’t have any question, we can start.

Moderator. We want to start by asking you to tell us what you know about genetic research.

Resp. 10. Genetic research is investigation on what you inherited from your parents/family. Also the diseases associated with genetic research are: stroke and can affect blood, heart and brain.

Resp. 9. Genetic research is investigations or finding that concerns non communicable diseases that affects family, e.g Leprosy, Blindness etc.

Resp. 7. Genetic research is an act of findings; it is through genetic research a lot of diseases could be diagnosed (identified).

Resp. 6. Genetic research is making findings on diseases like sickle cell and other blood related diseases.

Resp. 4. Genetic research is is carried out to know if stroke patients have family history of the disease.

Moderator: Can you explain what you understand by biobanking?

Resp. 10. Biobanking is keeping as the name is banking of blood in the hospital for use after sometimes.

Resp. 2. Biobanking is keeping of something like blood or anything that concerns human beings.

Resp. 1. Biobanking is keeping of blood, part of brain or heart in the hospital.

Resp. 9. As they said, kidney and heart can be stored for future research.

Resp. 5. Yes, I knew that, skin and bone can be removed and kept in certain places for further researches.

Moderator: Can you explain what you understand by precision medicine?

Resp. 7. Is having illness or disease, specific type of drug is used and the illness is treated. E.g Malaria treated with some Anti-malarial and cured.

Resp. 6. Yes, as he said, my brother also suffered from hernia and he was operated and had since get cured, some drugs were given to him.

Resp. 4. Even me, my child was having stomach ache and was taken to hospital and was given drugs and immediately cured.

Moderator: What do you understand by brain donation for research purpose?

Resp. 6. Yes, I have heard of brain donation for research but do not know how it is done.

Resp. 9. Yes, heard of it, it is possible to do that but did not see it done here. I am sure most people will not do it due ignorance.

Resp. 7. Yes, I heard of it, it is possible, because I had once undergone brain scanning in the hospital.

Resp. 1. It is possible, but due to ignorance and lack of awareness they don’t donate.

Moderator: What do you understand by blood sample donation for stroke genetic research?

Resp. 8. Giving blood to do research on stroke, I am ready to do that.

Resp. 7. Giving of blood donation to do test for stroke, and also DNA to determine the gene of an individual.

Moderator: Share with us your opinion and thoughts about blood sample donation for stroke genetic research

Resp. 6. It is something very good to donate blood for stroke genetic research, but people lack awareness to do that, there is need to inform the public about that.

Resp. 10. My opinion is that, it is very important to donate blood for stroke genetic research, in fact, when it is done or carried out, it facilitates early prevention of diseases.

Resp. 9. It is very good and important, prevention is better than cure. People now a days are becoming more aware, they visits health facility for HIV screening to know their status.

Resp. 2. It is important, the donation can serve as ways of knowing and prevention of diseases, that is why some diseases like Leprosy is about to be eradicated in the whole world.

Moderator: Tell us what you know about informed consent.

Resp. 6. There is need for consent to be obtained from the participants; the person must be give consent before participation or taking his samples for the research.

Resp. 7. There is very need for the consent, the researchers or doctors must explain to the participants, and they have to agree and also sign.

Resp. 9. There is need for consent, explanation on the aims and objectives of the research. And also Ethical approval has to be given.

Moderator: What is your opinion on storage of blood sample and blood fractions?

Resp. 2. It is good to store blood sample and sometimes even the fractions, if not because of such act, diseases like leprosy will not have been eradicated.

Resp. 9. It is good to store samples, if not; diseases will be increasing and alarming, and it will affect the developments of the country.

Resp. 1. It is good, researches help to inform and make people and government to know the types of drugs or medications for certain types of diseases.

Moderator: Tell us what you know about sharing of data, blood/blood fractions, brain images (CT scan/MRI) as well as brain tissue samples

Resp. 10. Sharing of data or results of tests is very important, because problems will be identified and solutions to the problems will be suggested.

Resp. 7. It is very important to share data and other information with others, it will make people to be off to dates.

Resp. 6. It is very important, it increase knowledge of the researchers and the providers.

Moderator: Share with us your thoughts about return of individual research results and incidental findings

Resp. 3. There is much need to return the results to the individual, a skilled health provider ro researchers has to be used to give result.

Resp. 6. It is very important, but there id need to counsel the participants not to give results without counselling.

Moderator: Explain your understanding of Biorights.

Resp. 7. Bioright means someone having right to dictate how his sample will be use, but my view is that he has no right after consenting and giving the sample..

Resp. 6. Yes, the participant has no right over his taken blood or anything from him as sample.

Resp. 9. My opinion is that the participant has right to his collected sample, because is done with agreements and he has sign.

Moderator: What is your opinion about governance and regulation of biobanking?

Resp. 4. Yes, there is need to have rules and guided principles to biobanking, it will help in making the work very well with sanity and sincerity.

Resp. 6. There is need for Ethical approval or clearance governing biobanking to avoid any issue that will spoil the good act.

Moderator: Explain possible intervention for implementation of biobanking.

Resp. 1. There is much need for government to create awareness among public, through Radio, Leaflets and workshops to educate people on the issue.

Resp. 9. There is need also to use skilled health workers when taking the sample for research.

Moderator: Any other major concern or recommendation on use of blood or brain tissue for research in Nigeria

Resp. 6. There is need to increase ways of making people to be aware about research especially in the rural and semi urban areas because people are still having doubt on giving samples for research especially now we are talking of giving brain or its part for research.

Resp. 1. There is need to have a special training for the religious and community leaders on the issues of research and the types of samples to be collected for research at different levels.

Moderator; Thank you for the time and responses, we want to seek for your consent again we have a brief written survey for just about 5 minutes.

**Table 1: African Neurobiobank for Precision Stroke Medicine ELSI Project Novel Intervention Tools and Platforms**

| **Novel Tool/Platform** | **Goal** | **Target** |
| --- | --- | --- |
| Biobanking and Personalized Medicine Community Advisory Board (CAB) | To bridge the academic-community gap an enhance participant and public education regarding ELSI related to biobanking and stroke genomics. | stroke community and general public |
| Based on the data and my analysis, the following needs to be included in CAB: | | |
| Neurobiobank Expert Consensus Guideline | government and professional association sanctioned expert consensus ELSI stroke biobanking and genomics policy guideline document to enhance best practices among professionals involved in biobanking and genomics research | Stroke community, stroke genomic and neurobiobank reseachers |
| Based on the data and my analysis, the following needs to be included in the Neurobiobank Expert Consensus Guideline: | | |
| Biobanking Training Programs (Workshops, Seminars) | To enhance biobanking awareness and understanding of related ELSI issues | Researchers, Ethics committee members, policy makers, public health practitioners, health administrators, CAB members |
| Based on the data and my analysis, the following needs to be included in the curriculum of the Training Programs | | |
| Biobanking and Precision Medicine Community Awareness Programs | To promote public understanding regarding biobanking and stroke genomics ELSI | Faith-based organizations, lay public, print and electronic media, mHealth technologies, social media platforms, film industry such as Nollywood |
| Based on the data and my analysis, the following needs to be included in Community Awareness Programs: | | |
| Stimulate the establishment of the African Neurobiobank Network | To provide a platform for regular engagement of biobank researchers, development of harmonized guidelines for neurobiobanking in SSA based on international best practices | Stroke community, stroke genomic and neurobiobank researchers |
| Based on the data and my analysis, the following info would be helpful for sharing with researchers: | | |
| Development of informative video on neurobiobanking, genomics and precision medicine | To enhance biobanking, stroke genomics and precision medicine literacy and related ELSI | Stroke community (patients and persons at risk, caregivers, advocates) and lay public |
| Based on the data and my analysis, the following needs to be included in the video: | | |
| Development of training video on neurobiobanking and precision medicine | To enhance biobanking, stroke genomics and precision medicine literacy and related ELSI | Researchers and academics (stroke, genomics, biobanking) and ethics committee members |
|  | | |
| Development of comics and case vignettes relating to neurobiobanking, genomics and precision medicine | To use comics and case vignettes to illustrate key ELSI-related scenarios and how to deal with them | Stroke community, lay public and especially young people |
| Based on the data and my analysis, the following comics and case vignettes need to be developed and should include: | | |
| Capacity training program in genetic counseling and biobanking science | To develop a crop of professionals with relevant competences in neurobiobanking and genetic counseling. | Biomedical scientists, public health professionals, postgraduate students with relevant qualifications |
| Based on the data and my analysis, the following needs to be included in the genetic counseling and biobanking science for educating professionals:including Biomedical scientists, public health professionals, postgraduate students with relevant qualifications: | | |
