# Supplementary material for: Research participants’ perception of ethical issues in stroke genomics and neurobiobanking research in Africa
Source: PLoS One. 2025 May 6;20(5):e0292906. doi: 10.1371/journal.pone.0292906 (PMC12054916; doi:10.1371/journal.pone.0292906)
Supplement: S3 File — (ZIP) [file pone.0292906.s003.zip › Files for PLOS ONE - updated March 2025/Kumasi-Community Advisory Board_FGD.docx]

DATE OF TRANSCRIPTION: 10^th^ October, 2019

Interviewer: Thank you. Please erhm there is something called genetic research. It is a research on blood about where you stay. When we talk about genes… blood, where you stay, the environment, studies about the body and the surroundings that you live in. When we talk of genetic research, this is what it means. So what do you know about this. Anyone can answer. You raise your hand, mention your name and tell us what you know about this.

Member: This is a really tricky question

All: [Laugh]

P: I don’t know, I think when we talk about community, we are talking about how the environment is about cleanliness and things that enable people to live well, like the water that the person will have to drink has to be clean, maybe in the environment, what do we do? Do we dispose our rubbish well? ...all these things are involved and also with regards to the body itself, how the person will keep him or herself clean. Excuse me to say, there are some people even the way they dress, we are not saying go and buy new cloths but whatever you have, you to always keep yourself clean so that whenever we talk of human beings, we can count you among them.

Interviewer: Please what I said was… please have you heard anything about genetic research? If you didn’t understand, we can explain again. All that we are saying is that it is about the human body and things in this community’s surroundings or the environment that can cause disease in the body. Have you heard of any studies about this?

P: oh yeah! Yeah!

Interviewer: you have heard about this?

P: yeah!

Interviewer: do you want to speak to me about it?

P: yes, what I heard about this is, as I was saying earlier, maybe generalized it. Ermm, all that I have about this research is that, erhm it enables people to know about the character of persons in the community. As I was saying about the rubbish, it is the source of most of the diseases so it doesn’t make the body feel strong all the time. And also, I said if the water in the community does not help, it can cause diseases to the body, it doesn’t make the body strong. Because the things in the community are not good, you will see people falling sick in the community all the time.

R: erhm, from what we are studying and discussing here, for me I think what we eat, drink and the air we breathe in bring about diseases. So when in our community, just like the man said, the overflowing rubbish is the same thing we breathe in and it the same thing that will cause us to fall sick. What we eat, if for you, you prefer to use the bigger Maggi cube for your soup, it means that it can latter cause you to fall sick. Whatever you are eating, you have to ask yourself what the outcome is going to be the next day. The water that we drink, most of us have even stopped drinking pipe borne water, we prefer sachet water. The sachet water too I can say that about 95% of them are not good but that is what we drink. When you test the pipe borne water, although there is dirt in it, it has a good pH with nothing bad in it. So if we do this research, I think it will all depend on our neatness. This is what will enable us to know whether we can get rid of the sickness or not.

Interviewer: please does anyone have anything else to say? Please have you heard about anything, as I asked earlier that comes with stroke?

R: I have something to say about it. Stroke comes with high blood pressure. And what brings about the pressure is anger or too much thinking. You see, the bible says we shouldn’t think to worry ourselves because you can’t think to change one strand of hair to white or black. And because most of us can get angry with people for a day or get angry with people we live in the same house with for a year, this brings the pressure. There is some ocean in our body…water. This make what we eat, drink and the air we breathe in like the galamsey work. If you make it dirty, it will result in disease in the near future. So, the more that you take in food that you think are rich but are actually death, these are all the things that bring about the pressure with its consequence resulting in stroke. So I think if we advertise this well and as we have a journalist here, if we take this to the radio station and the other time I was telling the leaders that we should do more programs and talk more about this on radio for more people to hear so that we can consider what we eat to see whether it won’t cause diseases later. The little thing I learnt from this enabled me see that when an Israelite is coming to your house, he or she comes with his or her water. Why? Because he or she doesn’t have confidence in the water that you drink so he or she brings his or her own water, because he or she is thinking about the next ten years ahead. Whatever you are eaten now is the same thing that is going to cause sickness in the ten years ahead. Sometimes we blame God for somethings but it may not be God. The bible says lack of knowledge my people perish. If you that learn something and eat well, breathe well or drink good water and you take sugar and too much meat the only end result is to cause diseases, I believe so.

Interviewer: Please does anyone have anything more to say about it? So if we do genetic research as I said, studies about the human body and surroundings; the environment. These are the things that come together to form the human body. It is in your blood, your hair, a lot of things come together to bring about this. If we research on this, do you think it will be of any benefits to us?

M: I think with the genetic research if we do it will help but my initial comment is that, if you take a look at genetic research, little is known by people in this environment or community. It is only known to those in the academia or people that it matters to them but when it comes to our part of the world, people don’t even know what genetic research is all about. With little education, or if we are all given the opportunity to know such this, it will really help us in terms of our lifestyle. When you look at our environment, the food we eat and everything, it will really help us. So with the research that we are doing, I feel like it will help in the sense that they will come out with their findings and through their findings they can help the community. They can be able to identify the various elements in the community then as a result of that they can find remedies or solutions to our problems. So I feel genetic research is very important, the outcome will be very important to the entire people or the entire community. Thank you.

P: ermm, they say a problem known is half solved. So when you are doing any research, the purpose of doing the research is to find out something, then you know that based on the things that you found out, what are the steps to take, what solutions should be given to the problems identified. Provided that after everything we won’t take it as a book and put it somewhere, but rather we implement and make sure that we are solving the problems identified through the research, then it will be of importance. But should we finish the research and we don’t work with it, then it is off. So personal, I think we benefit greatly from research and this genetic one is no exception provided that we implement the outcome and be able to find solutions so for me is you ask me, I will say genetic research is of really great importance.

M: I think we have already spoken about what causes stroke. Some years back, when we say someone had stroke, then it was someone who has money or a wealthy person but nowadays stroke is common. What made it common? I think it is the duty of doctors to find out what bring the stroke, what medications was the research able to provide for stroke patients. That hasn’t come out yet. When you go to the hospitals, most of the patients are stroke patients. If you take a look at this old lady, it started just like joke and left her there. If there was a medication for it when it started, the moment she came to the hospital, we would have done research already to know which medicine to give her, but we don’t have this. Most people have now been using herbal medicine for stroke. Why should it be so? We have the so-called professors; do professor manufacture the herbal medicine? So can’t they find out whether a medication is good enough to cure stroke? Haven’t they been able to do it? So, we have a problem there. Erhm, the research that we are doing will also help but just like my brother said, not that we will do the research and put it in our rooms, you should go and play the it to know the things on the it and work on them. That is what will help us.

I: When you take a look at stroke, initially when it came it was for the elderly that were getting it but now, even children are getting it. So, the research will be really necessary because it will tell us the source of the stroke. Also, mostly it is brought about by pressure and the pressure too, when we take a look inside the blood, there is something called predisposed factor; if your father has it, then the likelihood of you having it is very great. So if there a child whose father has this, we can take a look to see whether the child is also carrying the genes, then we quickly start advising him or her so that his or her lifestyle won’t worry him or her in the future. So the research is very important.

Interviewer: Please there is something called bio banking. Who has heard of it before? Bio banking?

P; ermm I have heard of biobanking but I don’t know what biobanking really is.

M: When we say bio banking, it is something like; it is process of storing some human tissues such as blood, like the brains, erhm maybe the kidney and other things. The act of storing all these things is known as the bio banking.

Interviewer; So, just like the man said when we say bio banking, it is taken part of the body tissues or some blood or some part of the body for storage so that it can be used later for some research. Do you believe that it is necessary that we do this, store these things?

R: ermm it would really be necessary but in this country Ghana, our problem is after collecting the thing we won’t use it for that purpose, you will only see it being sent to a different place. Just like Gee (referring to KATH), or the hospitals that collect blood, when we go to donate the blood, they don’t give us any money or we don’t collect any money but when you fall sick and go there, they tell you to bring money. Also, the education has to be intensified so that a lot of people can hear about this because maybe if we didn’t come here like most of us wouldn’t know that bio what…?

All: Banking!

R: … banking is. So you see? It is by the grace of God that we have come here to hear about bio banking. So in my opinion, I think the education has to be more because I can say that if we are about thousand people in Kumasi, about nine hundred and ninety haven’t heard of this before or don’t know anything about it. I think so.

Interviewer: Alright.

M: I think bio banking is very, very important, taken blood bank as a typical example. In this part of our world, accidents occur too much. In one vehicle colliding with another, we can have about more than thirty, fourty casualties and at that moment, they need blood so if they are rushed to the hospitals, the first thing they need is blood. When you go to the blood bank and we have run shot of blood or there is no blood in storage, lives will be lost so it is very very important. When you look at the other tissues like the brains, kidneys and so on and so forth, it is very important but my question is that do we have the technology and the equipment to handle them. The expertise, sometimes, with the heart I know at Korle-Bu we have the heart surgery department of... ahaah and so on and so forth. So that is ok but what about the brains? Is it possible to transplant brains in Ghana? If no, then why do we keep it? Why do we have to have bio banking for the brains? What purpose is it for? Or it should be there so that if there is any collaboration between Ghana and maybe let’s say South Africa, in the event we need it or they need it there, we can quickly transport it for transplant? Fine! If that is the issue, then it is also very very important that we have the biobanking for the other tissues. So in a nutshell or in a whole, it is very very that we keep bio banking in our system.

Interviewer: Thank you very much… When you take a look at how things are in this country Ghana, does our culture, our way of life and our character help these things to go on? Like taking part of the human body or blood, hair, heart and things like that for storage so that we can use them for something else later when the need comes? Do you think our culture, our way of life and our character allow this?

P: I believe that in some aspect, yes. For instance, we have blood banks that radio stations, NGOs and other institutions intermittently organize that people should come and donate blood to be taken to the hospitals so that they can be used to help someone when he or she is in need. So with the blood aspect I think it is working very well. But with the brains and others, just as honorable was saying, it’s alien to most of us. I have never even heard that we can keep brains and kidneys. What I have heard is that when your family member has a problem and needs something that one, they say we should bring a family member so that they can take something from his or her body to support the person who is sick. For the blood, we know people even donate without even knowing who is going to use it, if they test and it matches then that’s all so our culture is not against this. But for the other, I think if the education goes on well, I believe that how Ghanaians have empathy - we feel for ourselves - so if we say we are going to use it to help a brother or a sister who is having a problem, everybody is willing to do it and I don’t think any of our laws is against this so I think it is the education that has to be carried out well.

Interviewer: thank you.

M: ermm when you take a lot at our cultural set up or our environment, erhhh it is made up of erhhh religious groups, tribal groups and then other affiliated groups. So let’s say the religious, there is a certain church that don’t even accept the donation of blood. Yes, so when you are in that community with some of these religious things then you see that we have differences in our beliefs in terms of whether we will accept or not. So for me I think it varies. It depends on the set up of the community. Like I was saying, when we were discussing, some religions don’t accept blood transfusions so in that case when you look at it religiously, some will say no and others too will say yes. So I believe he rightly put it. Some of these things are not known to us so when we have education, when we have awareness creation; it will help people to know that oh. if you can donate blood for the wellbeing of your neighbor, then in that same way brains, the kidney, the liver and so on and so forth can also be stored especially in accidents where somebody just dies on the spot, some of the tissues function or can be used so in that case that can be taken and then with the agreement of the parents or family members and even if possible, the government can come in, sometimes the government can enforce certain laws for us to this or that and you cannot run away from that.

Interviewer: Please those on side you are not talking, from here to here.

[Some respondents laugh]

Interviewer: do you want to say something ermm pastor?

R: it is really necessary that…the last time we came, we learnt something that the brains and all the other parts included will be needed at ermm their banks, how do we call it…?

Respondents: bio bank!

R: …biobank so that they can be used for research. So for this, honorable, they are storing it so that when time comes that someone is having a stroke and we test to see that his genes and mine are a match then they can use mine for research to see how we can treat this. So in case mine and this woman’s genes are a match, they can do research to find out whether the medication that will be helpful to me will also help when this woman takes it. So it is really necessary and required that we do such things for research.

Interviewer: Please there is something called precision medicine. When I say this, with the understanding I have to give a little explanation. Precision, while explaining this, it will seem like I am explaining the answer to you. With this we needed to ask who know something about precision medicine. Please does anyone know something about it?

M: are you talking about prescription?

Interviewer: precision medicine.

M: is it erhhh… then you need to explain to us because I was thinking you were talking of prescription.

Interviewer: when we say precision medicine, we want to say that assuming that uncle Patrick here, not because someone was given paracetamol and was healed of his or her illness, we should give him paracetamol too when he is sick. We have to take a look at his body and his genes and know the drug that will be good for treating him. That is what we call precision. It is like you are attacking or directing the treatment straight or you are doing direct treatment that is related to the person by virtue of the genes or the things in his body. Please have we understood it this way? Ahaa, so who knows anything about this? All that I have explained who knows anything about it? How do you understand it?

P: if I understood it well, I don’t really know. I am hearing it for the first time but the understanding that I got from the explanation suggests to me that for instance, if I am sick or if Mr. A is sick, the reason they say go to the lab to be tested is to assess you to know that this is the medication to give you. Some medications have high dosage and maybe because of your blood you are not supposed to be given that. For instance, maybe you are given paracetamol which is 200mg or 500mg and you have to take one of the 200mg or you are to take one or two of 250mg. Maybe for some people, they say because of your condition, take two or take it in the morning, afternoon or evening and others too they can say because of your condition you have to take it only in the morning. Also, there may be differences in the ingredients used in making the medicine. Although you can have the same sickness but when your blood is tested it could be that the medicine to give you will be a different medicine that also treats the same disease so you should use the different medicine given you. This will let me know that this is the medicine that is good for me. This is how I understand it.

Interviewer: please does anyone else understand? I have explained.

S: please what I also know about this is that for some people when they fall sick, they say oh I have a headache. Then the next person will say ohhh you have headache? I felt okay yesterday when I took brufen so you too go and buy brufen for it to go. That’s how I understand this.

M: What I also know about this is almost the same time but the problem is we are all different because we all belong to different blood groups. So what is good for me may not be good for you even though we have the same sickness. Maybe we both have headache, but when we look at the medicine that I have to take due to my blood group, there is no need for you to take that medicine and the dosage and all. But this part of our world, we abuse it in the sense that ermm if someone has especially hypertension… if someone has hypertension and I also have it, the drugs that were given you when you went to the hospital, when I tell you I have hypertension then you say ohhh I have been given a lot of the drugs, they have given me for about two-three months’ dosage so I am given you one month’s medication. So when it happens this way then we are abusing the drugs and it’s not the best because sometimes you worsen your situation or your sickness.

Interviewer: does anyone else know anything about it, precision medicine? Or you have said all that you know? Do you know anything about it, maybe whether there is a certain law backing it? Is there a law backing it or has it been arranged that for this, this is what is needed to be done with relation to precision medicine? Precision medicine as I said earlier is treating you or your disease based on your own test results.

Respondent: Say it in English for me to see something.

Interviewer: you want to understand… the question?

Respondent: Yes!

Interviewer: (laughs). So are you aware of any law or policy guiding precision medicine? This is what I have explained. (laughs)

**Member**: I wanted to look at…

Interviewer: …but the first question was can you explain what you understand by precision medicine? The caption of precision medicine… that’s why I initially laid down the things for you so that you get it.

S: what I know about this is we are told to stop buying over-the-counter drugs but we can’t but they always announce that we should stop. Although those at the drug store are not trained, they sell to anybody who comes there to buy.

M: you see, with the minister of health, Ghana health service, there are so many policies. Even Ghana has policies on over-the-counter ermm… how do we call it? When you go to countries like the French speaking countries, you can never walk to the drug store and just go and buy drugs or medicine. You can’t. Without prescription, nobody will give it to you because when you do it it’s a crime but here we don’t enforce. (Coughs) excuse me. The laws are there, the policies are there but the enforcement and implementation are our major problems.

P: yes! ermm as a media person for close to two decades, I have never come across any policy that controls people in that area much. I believe that there may be some policies but we don’t know so if there is I think that people have to be educated to know that there is something against or there is a policy made with regards to health service.

Interviewer; please what do you about studies done on ermm like someone given his brains to scientist for research after death. The person is dead and we know the person is just going to rot and decay and we say maybe he or she wrote before that… or his or her family members say that if the brain is intact and can be removed for research, then they should use it.

M: I think the other time I said these things that because we are not used to it, we don’t know about it. So when you see someone doing that we say he or she is a ‘lodge’ person. ‘lodge’- like those who say remove my head or some body part for storage… they do that… in this our community, I haven’t seen some before. Erherhh that’s why we are saying that the education… it solves this problem easily, because those things, for someone to write in his or her will that when he or she dies they should remove his head, eiiihhh!! The family members… I think something will happen. Unless the ‘lodge’ people, for them they are forced to do this, that and that. That’s what I think.

I: ermm we hear, within academia, we hear that someone has donated his or her kidney, someone his or her brain…

Interviewer: this is related to brain?

I: yes! Brain, we hear and also we hear about the kidney too but I haven’t seen some before. Culturally, it doesn’t happen often because if you go and do it your family members will even leave your dead body for you. Ermm we hear of it but we haven’t seen some before within the community.

R: I think here in Ghana, maybe for some of us if not for today wouldn’t have known that you can donate your brain to be store for research… maybe we wouldn’t have known. Here in Ghana the only one I have heard is President ‘Ackaah’ who said when he dies we should burn him and he was burnt when he died. Apart from that, I didn’t have known that someone can even ask to be burnt after his or her death. I saw some when I once travelled to Nigeria but here in Ghana the only person I heard about was that our former president ‘Ackaah’… vice president. For him, he was in a certain group just as honorable was saying, he was in a group, like how do you burn a whole former vice president? But he himself wrote to be burnt when he dies. I think right now this is new to Ghanaians, we haven’t heard it before so if it happens that someone can use his brains for something like that unless there is more education, announce it on televisions, radios even the radio man hear said he hasn’t heard it before, how can someone like me hear about this? (laughs).

Interviewer: so please what are the things that prevent someone from doing this? Can you mention some? may be because of his or her religion, ethnicity or place of work or what?

P: okay! ermm I believe well that the things that prevent this include religion, family and also the beliefs of the person. Moreover, the key aspect is lack of knowledge… the person doesn’t know so why should you tell him or her to do such a thing. That’s what I can say.

Interviewer: thank you very much. Please does anyone else have anything to say?

M: these things that we are discussing, I think religion is really part. Ermm when I was in Nigeria, we had a certain professor, Professor ‘Teye’, he doesn’t wear shoes and he has willed that when he dies…because he’s an academia…he’s not in good terms with his family so it’s only him and his children…that when he dies, they should remove his kidney or brain for research. When you go to the University of Lagos, they are still researching on it there.

I: when you look at this, it is largely about our knowledge. We don’t have the knowledge at all. If really had the knowledge it would be very easy. It is the knowledge that is low.

Interviewer: so what factors or what are the things that will encourage as to do that?

R: I think for all that we are saying… for bible I will say that I have read small even when I wanted to read the Quran I read small and stopped when it got to a point where I wasn’t getting the understanding when I read. The thing is that I haven’t seen in the bible that we shouldn’t give your brains for studies in order to save the life of others; I haven’t seen that in the bible. The bible also doesn’t prevent someone from donating blood to help someone gain strength or use it for research. So for religion in Ghana it is only JW who doesn’t allow members to donate blood to a patient. All the remaining groups donate but the problem rather is that I don’t even know what it is so how can I say I am going to give my brains for research? So that is where the problem is.

M: the problem is ermm education and awareness. These two things if we are able to increase ir it will help the person to do it because like you said erhhh erhhh for the lack of knowledge my people perish. So if people don’t know it, people think that if I remove my brains, it is not the entire head that is going to be removed or chopped off, I believe it’s the brains. So the education… we have to let people know that it is not the head that is going to be removed but maybe it is going to operated and maybe some tissues removed and then preserved. Because for this part of the world, if you go and bury someone without a head, it’s a problem. If you go and remove somebody’s legs deliberately like the person dies with the legs and you are now going to remove for research, it’s a problem. So we have to intensify our education, there has to be awareness creation so that people know, they will appreciate it and I know within the shortest possible time we will be able to live up to some of these things.

P: ermm yes! ahh I think that education is key for the people to be able to get the understanding that the thing that we are doing, this is how we can do it and these are the benefits that will be derived from it. One other thing is discrimination…like a relative of mine has donated some before… his brain before…and it gets to a point that another relative of mine needs some. When he goes he won’t be given, someone will say they should give it to another person at the expense of the one whose family member has donated before. When it happens this way, the people will not do it again. Then two, the rich and the poor… when a rich person is about to die, they run to give him or her some because he or she can pay and for the poor person he or she can’t pay so they won’t give him or her so it will discourage the other family members in times of need if someone is to also donate, that person will not do it. If the education goes well, the work that they will be used for has to also go on well. It doesn’t matter who the person is, whether the person is a Muslim, a Christian or whoever, or whether the person is rich or poor, they have to benefit from it equally so that the discrimination cannot take place. Other than that it won’t cause people to have interest in it.

Interviewer: ok please thank you. Please what do you know about donating blood for genetic research? Let me remind you again that genetic research is a research done on things that come together to form the human body. It is in our blood, our character, our hair, our environment, anything that makes us who we are is the genetic, the genes that come together to form the body. A lot of things come together, so scientists will say that genes form the basic units of human life… ahaahh that’s how we say it. So what do we know about donating blood so that it can later be used for studies on genetic research?

M: erhhh if we want to do genetic research with blood…using blood, definitely people have to donate. If we don’t donate or have the blood, the sample or the availability of the blood, then the research on the genetic this thing cannot go on. So erhhh people donating and for us having a blood bank for the purpose of research is very very important because research is something that is ermm…ailment or sicknesses doesn’t prompt people before it comes, it comes suddenly. During the outbreak of Ebola, we needed blood to do research, we needed blood to do research on Ebola and so on and so forth. Therefore, the blood should be readily available; people should be prepared to donate so that we can do the research. It shouldn’t be the case that the sickness will come before we do the research. So I believe that the storage…the donation of blood and its storage is very very important towards genetic research.

P: I believe when it comes to blood, almost every house in Ghana believe that we donate blood and they also the importance of it because when accident happens, we say the person will need blood…someone can fall sick and be taken to then hospital and they will say he or she needs blood. So with the blood donation I don’t think it is a problem for Ghanaians because most of them understand it very well. At my station we do blood donations; we bring in nurses to come and take control of the donation so we know how people respond to this. Other stations and other…like I said earlier on other NGOs do some for people to… some people even voluntarily go to the hospital to donate blood, but aside all these, I also think there may be some small group of people that don’t see the need to, so there is the need to educate them also. So without education… we cannot remove it from anything because day in day out peoples mind can change… someone can even say that don’t do this again, it doesn’t help… why because you were not given any incentives when you went to donate. People should know that the required thing is not about getting something from your donation but about saving people’s lives that is what has to go on. So I think that with the blood donation it very very important that it goes on so that we can get many bloods in our various blood banks to save lives.

I: I think we blood donation for the purposes of genetic research, I think it will really help if we use that blood donation approach to save lives because if someone is donating blood and he or she doesn’t know what we are going to do with it, he or she will be a little apathetic to donate the blood but if we show them the importance and the purpose of that donation, the person will be more willing to give.

Interviewer: please does anyone have anything else to say? Father you haven’t spoken.

A: [laugh] I think we are also learning from what is being said.

Interviewer: we want your opinion too.

A: you go on.

Interviewer: please do we know of any tradition or anything surrounding us or any religion that doesn’t want us to donate blood to be used for genetic research or those that want us to donate blood for genetic research? Traditions or religions or things surrounding us, our family… laws that prevent us from donating and also those same things that also encourage us to donate for genetic research. Do we know anything like that?

R: ermm I think the thing we don’t know. I think in Ghana, there is only one religion that says it not good to donate blood. Even with that there is a difference between this and that. With this you are saying we are taken the blood and keep them in storage so that we can do research on it to know whether me and honorable are of similar blood or something like that. But with this, I think they haven’t even taken their stand so we don’t know whether they will accept or not. In Ghana, I don’t know whether apart from them, there will be another church that will say that donating your blood for research is wrong. I don’t believe so. We have our elder Muslim brothers here, for this man I think he has been in it for long. Ermm I think Muslims are not against that… if they are against that then those professors who manufacture big drugs and become big doctors, what research did they do to be able to become that. So if they are able to do it then it means they do it over there so we can also do it. But all about this is that I don’t know anything about it. Something that I don’t know, how can I say that I am going to give? So if you are able to announce for everyone to hear then I think if the person understands it won’t be hard to donate as the presenter was saying that people come to their station to donate blood. If people understand, they will go there and donate the blood for them to work with. That is what I think.

S: please apart from religion, for some people their Hb level can be down. For women like this, if you even have ten you cannot donate. The men too if yours reach twelve you may not be able to donate. Maybe you will create awareness that maybe for research, we won’t take much… maybe we won’t take one unit blood… then we will get the understanding. Other than that someone can say he or she doesn’t have much so he or she won’t donate.

P: ermm I believe well that just like pastor started saying initially that only one religion in Ghana is known to not agree to donating blood. Someone who doesn’t donate blood, it you tell that person to give you a sample for research, that person will not give it to you because at the end of day, he or she won’t need or benefit from it because it’s not something accepted by his or her religion. If you ask whether we know something that prevents donating blood, religion is part. At least we know a religion that doesn’t donate, maybe if there are other beliefs that don’t do it… I can’t tell but at least we know that some peoples’ own doesn’t allow. Another thing that prevents this is I know that… some people don’t have… how do I say it?... doesn’t believe in him or herself because someone may know that when he or she goes, maybe they are going to test or collect his or her blood for research but at the end be used for different test to diagnose different disease, HIV and others. So it all comes to education that no, the purpose of this sample is for research, either research on stroke or…specifically this is what we are going to use it for. If that happens that way, the person will be willing to do it but other than that it will also prevent the person from coming to do it at all.

Interviewer: please do we know… thank you… know any law or way or something that has been said to help in donating blood for genetic research or storing it for research? Do we know any law like that? Are we aware that there is a law like this that encourages such thing?

P: ermm personally, I am not aware but if there is then we have to encourage it for it to help because at the end of the day… we have already said that there is a purpose for every research. The purpose it to bring out something good… the outcome will help the body so I think if it’s there then we have to encourage it… even if it’s not there, we have to bring it to help.

Interviewer: please does anyone have anything else to say? Can anybody share with us his knowledge on donating blood to do research on stroke… genetic research that is related to only stroke?

Respondent: the question again?

Interviewer: can you share with us or do you have any knowledge of or what do you want to tell us about collecting blood to do research on stroke?

M: you see I have already talked about these issues that initially, doctors, professors… before the disease begins to manifest… we take the blood and do research to see that or someone who has stroke comes to the hospital, we take his or her blood and do research to see that this sickness is what is causing the stroke then we announce that come and let’s take blood and… like everybody will go. Because that sickness is very dangerous, it can destroy a person so the purpose… announce it and let’s see what we can do.

Interviewer: so you would like that…?

M: I would like it, yes! yes!

Interviewer: does anyone else have anything to say?

S: please, a lot of people know that when we talk of stroke then maybe it is a blood vessel in our brains that have become weak or burst. If maybe the stroke was as a result of a disease from the blood, a lot of people don’t know. A lot of people think it’s the blood vessel.

Interviewer: does anyone have another comment? Do we have…does anyone know about things that prevent someone from donating blood specifically for stroke research, stroke genetic research? Does anyone know anything that prevents something like this?

P: I will be very surprised for someone to say as for me we are doing a certain research about stroke so I have to contribute or a certain relative can contribute maybe by way of donation it could… it would be disturbing. If there is, I will be very surprised. I think…I don’t believe; I don’t believe… but if it is there too it won’t be that surprising because it is humans that we are dealing with. There are some people too no matter what you do they won’t do it.

Interviewer: So for you, what will prevent you from donating blood for stroke research?

Patrick: nothing… nothing... unless my strength…unless I am told that I cannot donate because of my strength, other than that nothing will prevent me.

Interviewer: so what do you know about obtaining… ermm receiving help that we would want to work with you or do research with you… since we want to do research with you, there is a contract to sign which will show that you have accepted to work with us… this study… what we want to do, who knows anything about it? In English we call it informed consent… consenting that we should do something or consenting that we should we should use your body or we telling you to do something… the whites call it consenting. Ok I tell you that we should go and donate your blood today and you accept and sign a contract… what do we know about this?

M: what I know is that everything that you do you need to be briefed, you need to be educated on it. If you get the understanding and accept to do it, before if it is about signing some documents, then you sign. Because I don’t believe that ermm we won’t inform you or educate you or let you know if we want you to do something, just that they will tell you to come and donate or come and let them do something to you. Definitely, the awareness will be created, your consent will be sorted and then at the end of the day if there is a mutual agreement then… how do we call it… you will also endorse or sign certain documents. So I think that thing exists.

Interviewer: please I’m still the informed consent that I am asking about, that…

S; please it is necessary that you sign the informed consent because every human that… every human that…

Interviewer: what you know about it?

S: yes! What I know about it? What I know is that it is important because of issues that may come in the future… to have evidence; you have to make sure that you sign a contract.

Interviewer: please what again do you know about informed consent?

I: it means you are aware of everything that you are doing… you know what you are doing.

Interviewer: please its let with just two questions for us to finish so don’t lose hope now

All: [laugh]

Interviewer: please what do we know about signing a contract for genetic research? The question is similar but there is a little difference… that we are signing a contract… the other one was what we know about informed consent in general but for this we are saying… using it for genetic research, like our body, our environment, things in our body… what does ermm…how do you understand it, you are signing a contract to be used for a research about such thing?

P: I believe that for every research that you do, you have things to look out for, the sample size and everything. At the end of the day when writing your report, you have to know that this person or that person was part of those that were used for the research and this is the proof that the person even agreed to be used for the research so the person signed the consent form that why we used it. So for the purpose of evidence that is why I think it is necessary to sign the consent form… someone should sign when the researchers come to do their work.

M: I think research… if you want to do research and you sit down and reach an agreement; you are not to go beyond the things that you have agreed on unless with the consent of that person because you have agreed that this is the purpose of the research. Any other thing incidental, if it is not part of the agreement, then you don’t have to go there because if you do then you have gone against the contract or the consent of that person. So the consent that has been signed will guide you and also guide the one who has volunteered to donate the blood so that he or she can also monitor to see that you have not gone outside that agreement. We have some agreements too that initially doesn’t go with any financial benefits, you are donating for free but at the end day if incidentally some profit is obtained, at least morally even if it’s not part of the contract the person should also benefit. You shouldn’t use his or her blood sample, do something incidental then when there is some financial benefit you enjoy it alone. So I think ermm the agreement will go to the extent of trying to monitor the things that they are doing. It shouldn’t go beyond what you have agreed upon. If you go beyond that then it is an abuse of the contract.

Interviewer: please does anyone have anything else to say? Please we have several types of the consent some include what I am coming to explain. Which of them would you like to do? Also if you say the one you would want, you will have to tell me why you would want that when you are donating or doing something. We have one called broad… broad consent – with that one when you donate blood or donate part of your body to be used for something, you give them the right to do whatever they want with it. It is broad, it is open, and you give us the right to do whatever we want with it. Would you want that or want the one that you can specify that the blood that you are donating should be used for maybe stroke research only and nothing else; if you do anything else with it, I will sue you. Or do you want the one called tiered where you are given a lot of options for you to decide the one you like… they give you A, B, C, D, then you choose that you want C so they should use it for C? We have another one called dynamic. That one too you will do a follow up to know that this or that is what your sample is being used for. All that I have mentioned, which one would you like to do and give me reasons why you would want that.

P: ermm I think I will go for the last one, the dynamic one because for that at any particular point in time… what is at stake if I am okay then I go by it. So I think that is what I would choose.

Interviewer: Father, please what would you also want?

M: I would like the restricted one. Ermm, previously I said broad but I will limit myself to the restricted because sometimes when you look at this part of the world, people tend to abuse even contract with impunity… nothing is done to them. You send it to the law court… this is the agreement, the person has abused it and yet you don’t have any remedies to it. So if you give him or her broad consent, he or she will do everything to the extent that whatever the outcome, you may not get the first hand information or feedback on it and it will be going somewhere else. So I will prefer the restricted, we sign an agreement restricting what you are going to do with it, I monitor it and in the event that you breach it, I will hold you accountable or responsible.

I: I will go for the dynamic one so that I will be able to track the progress of everything that we do.

Interviewer: Auntie nurse, which one would you choose?

S: the restricted one. (laughs)

Interviewer: why?

S: that is what I think is good for me. (laughs)

Interviewer: okay! Okay! Pastor which one would you choose?

R; you see the thing is new we haven’t understood it well.

Interviewer; we are not saying we are coming to do it to you.

All: [laugh]

R: oh I know that… I know that. The thing is new so it’s like something new in my life.

Interviewer: ok so we are bringing everything to an end. There is a man here; we didn’t hear him speak for once. Father, please what have you learnt about everything that we have said today. Please mention your name.

A: we can learn a lot of things from here when we go, we won’t hear anything, we won’t know how to teach someone to do this or that, and we won’t see that, ahaahh it has to …

Interviewer: is that why you won’t say some?

All: [laugh]

P: I think we have learnt a lot of things from this for instance I heard there is something called biobanking. I have heard of it but didn’t know what it was but ermm through honorable and also you the resource persons I have been able to understand certain things. Now with the stroke aspect I have seen that people are thinking of finding a medication to prevent stroke or they are looking for things to prevent stroke even if someone is not having, by the time the research is done it can be that the person can be prevented from getting stroke… I think it is good. I have also realized that nowadays we have a place where we can store the brain to help people; we can store hair samples for research to help people. So I can say that I have learnt a lot of things from today’s meeting.

Interviewer: please what can you say about it? Is it a good thing or not?

P: it is really a good thing… I believe it is a good thing but in order for it to be a success, just like I said in my earlier statement that… how we can implement… work with it, even if it’s a book, the book should be available for people to read or the education should go on well for it to help because we are just a few people here, if it doesn’t go out there and help the remaining people then whatever we came here to do didn’t yield any good result.

Interviewer: please what does anyone else have to share us?

Ibrahim: I have learnt about genetic research, I have learnt about biobanking and I have also learnt about ermm a lot of things from the education on stroke. Also, what I can add to this is that if we leave all that we have learnt here, all that we are doing will go to waste. So if we take it to our churches and our mosque and educate people and our whole community… that is what will really help us.

Interviewer: Pastor, what have you learnt today from we donating blood, brain or part of our body for storage for future research purposes related to stroke or anything else, what have you learnt from this?

R: I have learnt a lot of things but one thing is that I alone or those here, just like the man said, are few and the thing has to go out there for a lot of people to hear. A person donating his or her blood or brain for studies on stroke will help a lot people… a lot of people. So it is a good thing. That is what I think.

Interviewer: so elders we will thank… nurse you didn’t say yours? What have you learnt?

S: (laughs) please I have learnt a lot of things since I came here especially just as the journalist said I didn’t know what bio banking was, today I have known what it is, so I have learnt a lot of things from here.

M: For me, with this meeting or arrangement, I am very very very sad or sadden because I have seen that we have a very very long way to go. If you take a look at our community, we don’t have information on so many things so I know… a journey of thousands begins with a step. I know that what we have learnt here… I believe that it will help us and I would want us to expand it… whether we will form some clubs or committees so that we can train them so that they can also educate our people on the ground… it will really help so I think it’s a very good thing, we have learnt a lot of things like they said, biobanking, whatever… we have learnt a lot and I hope that it will help us. Thank you very much.
