# Supplementary material for: Research participants’ perception of ethical issues in stroke genomics and neurobiobanking research in Africa
Source: PLoS One. 2025 May 6;20(5):e0292906. doi: 10.1371/journal.pone.0292906 (PMC12054916; doi:10.1371/journal.pone.0292906)
Supplement: S3 File — (ZIP) [file pone.0292906.s003.zip › Files for PLOS ONE - updated March 2025/Kumasi-SIREN Stroke Cases_FGD.docx]

DATE OF TRANSCRIPTION: 6^th^ October, 2019

***Good afternoon once again. Thank you for your patience and we are sorry for keeping you waiting. And so erm, my first question is, there is something referred to as genetic research, does anyone know anything about it please. Please have you heard of anything called genetic research?***

3: Please, in saying that, can you explain it in Twi.

***Arrrrrrrnn, please has anyone heard the word before?***

R2: How please?

***Please have you heard of anything concerning genetic research?***

R3: I don’t understand please

***An example is screening the blood or taking a part of someone’s body for paternity test. We are able to prove if a child is legitimate or not by taking the blood of the person.***

***Ahaa!! With this have you heard of a similar thing being done concerning the stroke disease?***

R1: Yes it can be done.

***Daddy please say your number.***

R1: R1

***Please have you heard of anything like that?***

R1: It is possible to detect whether a disease is stroke or erm, what is the other disease called?...diabetes by screening the blood.

***Please does anyone else know anything about it?***

R5: Please what’s my number?

R5: I am NUMBER 5. Errrh, if it happens like that and you get a stroke attack, you will not know. So you have to screen the blood to be sure if you really have stroke or it is another disease say diabetes. It is the doctor who can tell.

***Does anyone have anything else to say?***

R6: What I know is that when the attack comes, you wouldn’t know that it is stroke. The more you visit the hospital frequently to get screened, this makes it possible to know whether stroke or some other disease. Without the blood screening you wouldn’t know.

***Please erm, yes R 2***

R2: Blood pressure is what bring all of this. When you tie you hand…

***Dad please hold the recorder and talk into it, I can’t hear you.***

R2: Blood pressure causes stroke and other diseases. High blood pressure will eventually lead to stroke. So eeerh stroke begins with high blood pressure.

***Please does anyone have a different idea?***

R4: When you realize you have stroke…

***Mom, please you are Number 4***

R4: Aaaarh, your hands and legs change and you feel pains in your back.

***Mom please did you want to say something?***

R3: I cook in a school. One day after work, I was fine and felt no pain till I got home. I decided to wash. While washing, I suddenly felt dizzy and collapsed. My mom thought I was playing only to realize later that I had actually collapsed. They sent me in a car to a hospital at our place but the doctor asked them to send me to Kokofrom because my pressure was very high. Upon arrival, I was given a drip- that was on Friday. The doctor attended to me on Monday and asked to be sent to the emergency ward. I became unconscious and didn’t know what happened but some part of my body was nonfunctional after I regained consciousness. Hmmmm.

***Please, from the example I gave from the paternity test using the blood or the hair, the stroke… before I continue, what is your take concerning the causes of stroke. Daddy mentioned high blood pressure earlier, what other causes do you know of?***

***I can then base on your answer to explain what I wanted to convey.***

R6: Some symptoms include dizziness and a feeling of something crawling on your body which I mistaken for ants sometimes and even try to get them off my body. This happened continuously. I usually wake up at 2:00 am to pray but when I tried to turn sideways, I suddenly felt like someone who has epilepsy and is falling down. So it stopped after some time. I tried to wipe my face and realize I was salivating. I wondered what was happening and tried to tell my children but they thought I was lying. They said I always claim to see something when I sleep. I told them that when they sleep they don’t see anything so they don’t know what is going on.

I took my bath and went to my small shop in front of my house. My sister visited that morning and whiles we were chatting, I tried to turn and felt my whole body turn suddenly: they had to hold me to keep me from falling and then they brought me to Kuffour emergency. This is what happens.

***But please what are the sort of things that we do that results in stroke? As a person without stroke, what are the practices that can result in stroke?***

R6: Our diet.

***Concerning our diet, what are some of the things?***

R6: A lot of oil intake as well as eating late. We should always eat food that can lower our blood pressure and avoid eating foods that can increase our pressure. We should also avoid eating late.

***Please is there anything else***?

R 5 tries to say something].

R7: As Daddy said, pressure can result in stroke. To me, high blood pressure can be prevented totally by checking our eating habit as mummy said. Foods that is high in cholesterol like oil and eating heavy food when it is late. If you want to eat heavy food like fufu, you have to eat early so that by 6pm, it has already finished digesting. We also have to eat a lot of fruits; it can also help bring the cholesterol down. Exercising can also decrease the cholesterol level in the blood. All these will help decrease our chances of getting high blood pressure which especially causes stroke but when we check our diet and eat early and exercise regularly, the pressure would be checked and in essence the stroke.

R1: ….

***R5 wanted to talk so when she is done. (Laughs)***

R5: For me, the symptoms of my stroke didn’t include body pains that should have resulted in stroke. My hand was the only part that really hurt: it felt as if it was broken at times and any attempt to turn it brought great pains. These are the symptoms I had until the doctor told me it was stroke when I visited the hospital. I woke up one morning and fell while going about my duties but I later stood up. I made the youngest among my children stay home with me while the oldest went to school. I fell again and became unconscious and in my unconscious state they took and brought me here. They didn’t even know what was wrong when attending to me. It was the doctor who made us realize it was stroke and admitted me at D5. As for me, I didn’t become mute. But for me one thing was each time I take my kids to the hospital, I check my BP and they always told me it was okay as a result I forgot about it. That’s what happened to me.

R1: Some of the causes also include eating red meat and high fat meat. Too much eating of meat can also cause stroke. When it happens your heart beat very fast and also makes you dizzy.

***Mom please do you want to say something?***

R4: [fumbles] being careful of our salt and fatty meat intake.

***Please would you want to say something?***

R3: Please can you tell us what food to eat? Mmm okay. I get stomach pain because I have ulcer. I don’t eat plantain because each time I eat plantain I don’t sleep; my stomach really hurt. So I don’t eat plantain.

R5: What if in preparing soup with beef I decide to grill it; would it be okay for people with stroke?

***Have they asked you to grill it now?***

R3: I have not been told what to do.

***Have they asked you to stop eating the beef?***

R3: I have not been told.

***You haven’t been told?***

R3: Ahaa, what bothers me is the fact that the doctors do not tell us what to eat and what not to eat. Rather the ‘doctors at home’ are the ones who tell us what and what not to eat. Since we’ve met you can you tell us what to do?

R6: It results mostly from the intake of alcohol. If you are an alcoholic, you are easily prone to stroke.

***Dad please did you want talk?***

R 1: Our lifestyle – the foods we eat. If we are not careful about the food we eat, and as the woman said, also alcohol intake, living an ‘I don’t care’ life, paying no attention to our health. You have to frequently go to the hospital and check your blood pressure. After visiting the hospital, you do everything the nurse asks you to do after they check your blood pressure. Eating especially, we should abstain from intake of salt, sugar, oil and pay close attention to our well-being.

***Thank you daddy***

R1: Getting angry often as well as thinking too much can increase pressure.

***You have talked about eating, exercising, alcohol intake, fatty meat and so many others. But are you aware that stroke can be hereditary. In some families, almost every member of that family at a particular age gets stroke. It can even happen that the person tries very hard to stop eating some foods but still at a particular age the person gets stroke. Have you heard of instances like that?***

R2: Aaaarrnn. They say it is in the blood and if especially either your mom or dad has it, it can be passed on to you. For instance, my dad was a pressure person. Pressure killed him. Even though he tried by buying several drugs and took them but at the last minute that was what killed him. They said he had it at a tender age and those days, drugs were hard to come by. He eventually developed a heart disease from the pressure and died from it. As a result I strongly agree to that fact.

R4: I would want for what’s happening to my hand and leg to go. I have ceased taking the fatty meat, the alcohol and the salt.

***Does anyone else have something to say?***

R6: For me as well, as they say it is in the blood. My dad did not have it but had rheumatism, and my mom become diabetic and died of it. My sister is also diabetic now, I believe therefore that it is hereditary that’s why we have it. I even inherited my dad’s rheumatism and I feel pain in my leg sometimes when walking. If I am careful when walking I can easily fall.

***Please I was trying to use these to explain what we were saying. Some people have the stroke in something we call genes which in Twi can be translated as in the blood or in the family, in others also, it may have resulted from somethings they did. It maybe because they didn’t pay attention to their eating habits or exercising and others. I asked about genetics research because now they want to be able to check our genes. If there is the presence of this in your genes, by taking a part of our body and checking, then we can know that if you are not careful you might get stroke. Sometimes the stroke is not in your genes but depending on your lifestyle you might get it. This brings me to my next question, there is something called Bio banking thus taking the part of the person say the blood, the hair and keeping them for the purpose of the research. Please, I really don’t but does anyone know anything concerning it. Please do you know something like that sort is possible where we take the body part of some people and keep them for the research? Has any witnessed or being involved in something of that sort where they take a body part for research?***

R1: As for that, it is only the blood they take because they can’t take you skin to research into your stroke except the blood.

R6: I also know that aside the blood they can also take your hair, your nail and even part of your buttocks for it.

***Mom please you can say what you want to say.***

R4: Should I say it?

***Someone’s hand is raised but if you have something to say, you can.***

R 4: Please it is possible to use the blood, the blood.

***Aside the blood and hair, is there any other part they can use? Please don’t you know of any part they can use? Apart from that we can take something like…. Some people whom when they are about dying, not only those with stroke, but some when they are about dying can donate their whole body for research. Some people donate their heart for the research, the blood we are taking is different from the blood we take when we visit the hospital or during blood donation where blood taken is given to other people, and we take blood for the purpose of research which is not necessarily for you but for general research. We can take the blood and as mom said the hair and even we can take your brain. When it comes to the brain or donating the whole body, even the heart it is dependent on how close the person is to death. At the stage the person makes a will telling his family to donate the brain and the heart to what we call the Biobank. As we can save money in the bank, there is also a bank where we can keep some parts of the body for research. I would want to know your take on this. For instance when we come to you for blood not for the purpose of donation or for your personal screening but for the purpose of the research, I would want to know your take on it. Is it something worthwhile or good?***

R5: Unless the doctor demands for my blood for the purpose of research when I go to the hospital, can I by my own will say I want to donate my blood for the purpose of the research?

***It can be either ways. The doctor can ask you to or the team can come and ask for the sample, or you can go on your own too.***

R5: Because I know a woman whom when she was about dying asked for her whole body to be given for the purpose of research. There was no proper funeral for her. She donated her whole body to the people at Tech. Is that what you are talking about?

***Yes what’s your take on it?***

R5: To me I think it can help someone.

***How?***

R5: People die from reasons we do not know, so in donating their bodies for such a purpose, it will help.

***Please does anyone know anything of it? If you’ve heard anything of it, do you know of any arrangement concerning giving your body or part of it out for the purpose of research? Do you have any idea concerning the arrangement that is for the research?***

***Also there is something in English language called Precision medicine. It means that….. Before that let me ask if anyone knows anything concerning it.***

R6: No please

***As we were saying, we are able to search the genes to know a person is likely to get stroke in the future or the person is free from stroke. Everyone has stroke but I am sure each person’s stroke was different. As mom said, a part of her body became weak and you also said your hand…***

R5: This hand was what got affected.

R5: NUMBER 4

***No,R5***

R5: The stroke I had affected this hand and this leg. The doctor after confirming it was stroke gave me drugs. I was limping a bit after I got well so I began taking herbal medicine as well as the one the doctor gave me. It was later I stopped the herbal medicine and concentrated on taking the drugs the doctor gave me till now. Since I no longer limp, there is no sign of the stroke. So if I don’t tell you I’ve had stroke before, you wouldn’t know. People even marvel when I tell them I had stroke.

***Do you realize everyone’s……….***

R5: I do not joke with my drugs at all.

***It is possible that the drugs that will work for one person will not be effective in another person. I am sure each of you had different manifestations of stroke disease. Medical research is seeking to be able to screen and identify what works for each person. They now want to be exact concerning what each person needs. This is referred to as Precision medicine. Strokes differ in people. As she said, you wouldn’t know if she doesn’t tell you, but others don’t have to tell you they’ve had stroke. I want to find out, do you think Precision medicine is possible? First of all, can it actually work?***

R 6: Concerning what you are saying, my first child died not long ago. Before he did, I was made to send him to Suntreso where I was asked to buy some drugs. After four days, I sent him to Kuffour emergency upon request by the doctor. I was asked to buy drugs again but it was the same as the ones I bought from Suntreso but he died. He was buried about two months ago. I fell ill last week: I was feeling dizzy and vomiting as well. They sent me to the hospital, Suame Kwanta hospital. When I went, they made me do lab and everything. But I realized that thr drugs they gave me were the same drugs thry gave my son. When my son died, I threw the drugs I was asked to buy away but I was asked to buy the same drugs when I went there with my illness. Amazingly, I got better but my son on the other hand died after taking the same drugs. So, it is possible to take samples from someone to help another person.

***Any more ideas? If no one has anything else to say then…***

R7: I believe we can give our brains out to others so that even if you’re not there it can be used to do what you said it should be used for.

***Any other ideas? What’s your take on Precision medicine?***

***Ok, I would want to continue then. Stroke originates from the brain which controls all the other part of the body. That’s what explains the weakness in the body part like the hand and leg when one has stroke. It all originates from the brain. The Neurobiobanking aims at obtaining brain from donors. For instance, someone can write in his will that when I die, my brain should be donated for research. This is an ongoing practice overseas but due to some reason it hasn’t been started in Africa. We are now looking to start.***

***What are the cultures and beliefs that can hinder it? Do you all understand the question? What are some spiritual beliefs and others that can hinder the start and progress of the Neurobiobanking? Do you have some personal beliefs that can hinder you from donating your brain for the purpose of the research or is there something you’ve heard?***

R 2: I think people need to be educated on this so that they will understand: blood donation was not a common practice but due to education, people are now willing to donate. The corpse of the person is, excuse me to say, useless therefore if there is enough education I think people would be willing to donate the brain for the research.

***What are some of the beliefs that prevented people from donating blood?***

I’ll say beliefs. Some churches such as Jehovah witnesses don’t accept the concept of Blood donation as it is against the norms of the church. Others also have the belief that blood donation can lead to a shortage of blood in their system, even heart transplant for example, the whites are doing it but here we don’t. Therefore, educating people on organ transplants can help people embrace it because our beliefs as Africans prevent lots of people from various donations.

***Is there anything else?***

R 4: [I can’t hear her]

***So apart from Jehovah Witnesses, are there no other hindrances? Can a family decide to disagree to donate even after an individual has willingly given out his/her consent to donate an organ after death?***

R7: The thing is, you the person who would want to donate your brain after death should first tell the family in order to prevent any form of quarrels and disagreements after your death. For instance, my uncle who was a Jehovah Witness gave out his word and gave out written documents before he died that no funeral rites should be done for him when he passed on. The family did it just as he wanted it done. So if you want to donate any part of your body, you have to make the family aware.

***R5, you mentioned that someone donated his body. What are some of the issues that came up after that in the family?***

R5: Yes, I saw it that way. Before his death, he did the necessary documentation which proved that he wanted to give out his body after death. In this case, the family raised no objection because they were informed and they had the documents to prove it. If there is no documentation or a word given out, then the family will disagree.

***This question is going to be answered individually; will you want to donate your brain after your death?***

R 1: I won’t do it.

***Why won’t you do it? Can you give me your reasons?***

R1: I won’t do it. I won’t.

***Why won’t you do it?***

R1: I don’t think it’s good for me.

***It’s not good for you? Why do you say so? We’d like to know why?***

R 1: Oh! It’s not good for me. I think that if I fall sick before I die, my blood will have sickness in it and I wouldn’t want to have it transferred into another person.

***We are not giving it to anyone. I am referring to brain donation, not blood donation. The brain donation will help us to do more research into stroke and get a better understanding of the disease. What will prevent you from doing it?***

R1: I still don’t want to do it. I don’t want to do it.

R 2: I won’t do it.

***Why?***

R2: Oh, I want to present my body to God intact. He has to see me in the same way He made me even after my death, I have to go and show everything to Him.

R3: I cannot do that thing.

***Mom please why?***

R3: If I’m dead it means the thing is spoilt.

***No, it’s not spoilt***

R3: It’s not spoilt? So can it save someone’s life?

***We’re not giving it to anyone***

R 3: You’d use it for research! And what work will it do again apart from the research?

***It is the research that will help us understand the disease better***

R4: Please…[fumbles] I want to use it to take care of my life and send it back to God.

***Mom please I didn’t hear you well. Are you also saying you won’t do it?***

R 4: Yes

R 5: We’ll get some from other people to do it so let’s use the ones from the other people to do it. [LAUGHS]

R6: As for me, I can’t because if I’m sick and dying, how can I tell someone to take something from me for something. I can’t say that use a sample from my body for something like that. When I die, then I know that it is of no use.

***This is in a form of a will so you don’t need to say it on your death bed. You can even say it now and have it documented.***

R 6: As for me, I can’t do that. I’m saying that because I know of someone – he died overseas – who said his body burnt. They brought the body and burnt it here in Ghana at Suame but everyone has different beliefs but my personal beliefs do not allow me to donate an organ like that.

***What are some of those beliefs?***

R 6: That’s what I’m saying. My beliefs don’t tell me to sacrifice part of my body like that. I will not take that of another person.

R7: I also will agree with NUMBER 2; I have to present myself intact to God after death.

***Since we have all decided not to be donors, how can the research continue?***

R 6: I also want to ask a question. Will you as a researcher be willing to donate your body after death?

***As daddy said early on, the moment you die, the brain becomes useless so I will gladly do it unless my family objects to it. I believe research is very important and we have to advance in that area so I will gladly do that.***

R 6: Someone is willing to do that, another person is not willing to do it. I believe that if the person does it willingly, it is better.

R 1: You didn’t answer the question she asked you. She said can you do it?

***Aah, and I said I can and I explained that when I die, my brain will just decay for nothing, so if it can be utilized in research, I personally do not have any issue with it.***

R7: Apart from using the brain for the research, what other means can be used to conduct that research?

***The brain is key because stroke affects the brain.***

***But again, apart from the brain, will you be willing to donate blood for research?***

R 5: Is it when you die?

***Blood can be taken even while you are alive***

R 5: Since it is only a portion of the blood will be taken and not my whole blood, I will be okay to donate. If they are taking some for research then I can do that.

R 4: I can donate blood for research.

***Is there anyone here who is unwilling to donate blood for research? I’d like to know why.***

R 6: If there is opportunity to donate my blood, I’ll do that. It could be that I’m sick and the doctor might say I can’t donate. But if it can be done, I will gladly do it.

***Apart from religious beliefs, don’t we have any superstitious beliefs in Ghana which do not allow people to donate blood? I have noticed that blood donation exercises go on a lot. Are there no beliefs that go against that apart from that of the Jehovah witnesses?***

R 2: Apart from Jehovah Witnesses, I haven’t heard anything like that.

***Apart from religious beliefs, do we have superstitious beliefs?***

R 2: [INAUDIBLE]…someone might have such thoughts but I haven’t heard any family or group or society saying that something will happen if you donate blood.

***Oh okay but have you heard anything regarding the use of donated blood for rituals?***

R5: We hear that a lot. As for that, they say it every day.

***Don’t you think that can affect a program like this?***

R 7: It is those who don’t have the understanding and education on blood donations that think it will harm them but there is no way donated blood can be used for rituals. It cannot be possible. I don’t believe that.

***Don’t you have any relative who will not donate blood due to some of these beliefs about blood donation? You may want to do it but can you think of anyone who wouldn’t want to donate?***

R 1: Well, I’m speaking for myself so I can tell if anyone have that idea. Personally, I will agree but I don’t know anyone who will not agree.

***In research works which involves humans, there has to be informed consent just like I sought your permission before I started asking you some questions. There are various ways through which the consent is sought with regards to how your donated sample is to be used and I want to explain them to you.***

***Firstly, there is the broad consent where for instance you donate your brain to be used for whatever research we wish to use it for.***

***Secondly, others also specify the particular research that he/she may want us to use the donated organ for.***

***Lastly, there is the form of consent where the donor is always updated on researches which may need his/her donated sample. Here, the donor can decide to allow or disallow the use of his/her donated organ for a particular research. The donor can choose to always receive updates on whatever that the donated organ will be used for.***

***These are the three ways; I want to know which one you will be okay with after you have donated. I want everybody to give me his or her view.***

R7: Once I have allowed them to take my blood, I don’t care what they use it for, I leave that to them.

R 6: I support what my brother said. Since I have allowed them to take the sample, I cannot restrict the use of the sample. It can be used for whatever purpose.

R 5: My donated blood can be used for whatever research there is to do. I have no objection.

R 4: I will also give my consent for you to take my blood.

R 3: They can use it for whatever research there is to do.

R 2: Once I have given it out, I don’t mind what happens after it

R1: I will be fine with anything.

***Thank you very much.***

***Will you be willing to donate your CT scan for future research works? Were you made to take CT scans at the hospital after you were diagnosed with stroke?***

R1: I was made to take two of the scans and they kept one at the hospital, so I don’t have a problem.

***Does anyone have any contrasting ideas?***

(Everybody: No)

***Okay, that’s fine. Researches go on all over the world and sometimes the research will require that samples be taken from other countries. For example, a research going on in Nigeria can require that we get samples from Ghana, will you be okay if your sample is given to other people to use in research?***

NUMBER 1: (laughs) Ok! What I want to say is can’t they donate some in their own country to use? You are coming from Nigeria to take our blood, what are the Nigerians there for?

***Ok, so you don’t want it that way?***

NUMBER 1: oh they should also donate and use it there.

NUMBER 6: I think that many factors come into play when we need to give out samples. For example, blood group compatibility is needed in order to give out blood. I went to the hospital and they checked my blood group. They told me that I am blood group O and this makes me a universal donor but in cases where I will need blood, I can only take from blood group O. So if they check and it’s compatible with the one who needs it, then I am okay.

***My question is that without you being informed will you be okay if another researcher comes from elsewhere to take your sample for his or her research?***

NUMBER 7: Like I said earlier, I will be okay with it. I have given it out and that is all.

NUMBER 5: [fumbles], I don’t have any issue with that.

NUMBER 3: Oooooooh I don’t have a problem with it. I gave it out so that it can be used to help others so I am okay with it.

NUMBER 2: Once I have willingly given it out, I don’t care what they do with it.

***Is that all? Do you have issues when donated blood is sold?***

NUMBER 1: I don’t have any problem. They can sell it in dollars if they want to. Once I’ve given it out, I don’t care whether they sell it or not.

***Won’t you wish that we get some of the money?***

NUMBER 1: We won’t even hear of the money aspect. Who will come and inform us? You won’t even know that they are selling it. They are using it to save someone’s life so if I hear it, it will not hurt me.

***Mummy, do you have anything to say?***

NUMBER 5: As for me. I will be happy if I can get a share of the money but if they don’t give me some, I won’t bother me.

(They all laugh)

NUMBER 4: They have my permission to sell it.

***I have two more questions to ask then we end for the day because we are all hungry. We need to eat. We will finish soon.***

***So the next question is that in case we notice that there is something in the sample which you donated, will you want to be informed? If yes, through what medium do you want to be informed; by calling you on phone or through face to face interaction?***

NUMBER 1: this is like breaking the news of the death of a child to his or her parent; no matter how long it takes, the news will eventually come out. I would like to know in any way possible. Treatment can be given.

***Through which medium can we get the information to you. Some people will panic when they get information over the phone. Others love to be talked to in person; so which one will you prefer?***

NUMBER 3: You can invite the person over to talk to him or her. For instance, you called us here to talk to us. We didn’t know what it was about but after you talked to us we accepted to do it.

NUMBER 4: Unless we tell you about it before.

NUMBER 5: It will be good that you tell me your findings. I prefer you invite me over to tell me in person and if possible give me treatment as well.

NUMBER 6: Call the person in a very nice manner. Don’t scare the individual with plain language. Such issues require patience and sweet talks so not to scare the person. Educate the person on the possible means of treatment.

NUMBER 7: Issues reported over phone can make people panic and cause the person to even die. Inviting people over to talk to them is the best. It is best to let people know of the possible treatments available.

***What can we do to introduce the bio banking into the Ghanaian system looking at our culture, tradition and beliefs?***

NUMBER 6: Education is very important to get people to accept the idea. We have to taught

NUMBER 7: I think that after people have agreed to be organ donors, there should be documentation to support the deal to prevent any quarrel with family.

***Does anyone have any other opinion? Okay, so do you think that setting up a committee to take up the project will help?***

NUMBER 5: It will be very important to set up a committee to take it up because it will be bad if we leave the project hanging with no one to attend to it. The government can take it up so even after research we can have qualified personnel to give us feedback on the findings without causing fear and panic in the donors.

***Does anyone have any contribution? Probably I left something out during our conversation or is there any question you want to ask?***

NUMBER 7: I am bothered about something and I think it should be worked on. Doctors don’t give us (stroke patients) any guidelines for healthy living. They don’t tell us what to eat and what not to eat. When we visit the hospital, they don’t tell us anything.

***Is it the same for everyone here? Were some of you given guidelines for healthy living?***

NUMBER 1: We have not been told yet. I heard fruits are good for healthy living but the doctors don’t tell us. We buy soft drinks and eat all sort of things but no one tells us to stop.

NUMBER 5: Only the local doctors draw our attention to it (laughs). Sometimes I will be eating something and then they will go like, ‘you shouldn’t be eating these things as a stroke patient’. I always forget to ask the doctors too, sometimes I plan to ask whenever I go to the clinic but I always forget. They only give us drugs

NUMBER 1: The herbal doctors say lots of things when they are on the radio. Personally, I can’t say that the drugs I’m taking a good. They just give us drugs and though I take them as prescribed, I really don’t see a change in my condition.

NUMBER 5: I for instance I have stopped taking my drugs because I met a new doctor when I went to the hospital the last time. The regular doctor has been changed. All the drugs the new doctor gave me makes me feel very uneasy, I palpitate each time I start taking it and my breathing rate also increases. I even brought the drugs today so you could take a look at it. So the doctors have to tell us the right things to do, it is one thing that bothers me a lot.

NUMBER 2: Salt, sugar and all of those things……... Coffee is something I really like; I used to take it every morning till I met a doctor who told me to stop and take cocoa instead. I had taken the coffee for a long time and I was well adapted to it but he advised that if I want to get any breakfast it should be cocoa. Now I have stopped taking the coffee and I have started taking cocoa. I don’t know why they don’t tell us; I think probably there are lots of people to attend to.

***Any more contributions……? Oh I see. Thank you so much for your time and patience. We will end here today.***
