# Supplementary material for: Research participants’ perception of ethical issues in stroke genomics and neurobiobanking research in Africa
Source: PLoS One. 2025 May 6;20(5):e0292906. doi: 10.1371/journal.pone.0292906 (PMC12054916; doi:10.1371/journal.pone.0292906)
Supplement: S3 File — (ZIP) [file pone.0292906.s003.zip › Files for PLOS ONE - updated March 2025/Kumasi_Caregivers_FGD.docx]

# **FOCUS GROUP DISCUSSION TRANSCRIPTION FOR SIREN STROKE CASES**

## Tell us what you know about genetic research?

6: We do research to identify the causes of maybe the disease which has occurred so that we would know how to prevent it. I could say that maybe its causes and its prevention. That is what I know about research.

“Tell me about your source of information”

6: News and documentaries made to gain that experience.

Have you heard about it?

What do you know about it?

6: For instance, maybe concerning the liver; maybe the liver let’s take it as we have certain things when it gets into the human system, it doesn’t help the liver and when it doesn’t help the liver, it would cause diseases in the human system.

Tell us about any experiences you or others you know have had with participating in genetic research

What do you know about genetic research in stroke?

8: The time my mother was sick and we brought her, they said that is the pressure that when it goes high then it brings the disease stroke. That what they said.

5: What I know about stroke disease is that if fats get piled up in your system and the veins get blocked then the blood cannot flow into the brain and the brain cannot pump the this thing, that’s what causes the stroke. If the heart cannot pump the blood into the brain, you see it’s the brain that makes the human system functions and if the blood cannot flow into the brain then part of the human system cannot function.

6: I also see it that if you have blood pressure and maybe you didn’t take your drugs or you don’t take your drugs and then your blood pressure rise up, that could also make the stroke disease affect you.

What do you think are the roles/benefits of genetic research in medicine?

6: Research helps a lot because it helps us to know the causes of the disease which occurred. It also helps to know the medicine which would help to cure the disease or its prevention; the things that you would do for the disease not to affect the body. That’s my opinion.

5: What research in medicine has helped us. It has made us to get good drugs that help us to when even a disease occurs, it could help us for the disease to cure early because at first there was no research and if any drug then you take but when health workers brought research in medicine; it has helped us to get good drugs which immediately when you take them then they help to cure the disease which has affected you.

## Can you explain what you understand by biobanking?

6: What I understand in blood bank is a place where we store blood and in case someone is sick and we need blood for him/her; that is where we go to for us to get the blood and give to the person. And that is the same place that when you want to do any blood donation that is where you go and do the donation and they would also store the blood so that when someone needs some and goes there, they would also get some for him/her.

Probe for level of awareness, understanding/perception of concept, sources of information,

6: What I have heard is kidney. It is kidney that I have heard that we have a place where it is stored which they do transplant on kidney. For biological samples, it is kidney and blood that I know are being stored.

The kidney; I know that we do transplant so they store kidney then they use it for the transplant. I heard it on a documentary. It was health documentary about kidney transplant.

8: recently my sister was sick so when we went to the hospital then they said we have to come and purchase blood for her because she was bleeding for about 3months. So they made us come to Komfo Anokye Teaching Hospital here and we came to buy 4quantity and they did the transfusion. So it was when they did the transfusion that they were able to do her operation. That is what I also know about it.

How does biobanking operate?

6: Is it as in where you would go or where when you need some of the samples you would get some? What I could also say is that the doctor who is treating you; he would show you on how you could get some and how you would go about it to get it. Or if it is a family member who could donate some for you; I know about that or a close relative could also donate some for you.

“Let focus our minds on blood; how does the blood banks operate?”

6: You coming to seek for blood; they would check your blood group. Your blood group would determine the type of blood they would give you. So for instance maybe if some is not at the bank or if maybe your sibling or your father or someone else and they test the person and see that you and the person’s blood are of the same group; you could donate to them and they would use it to treat your relative who is sick.

Belief/thought/opinion relating to biobanking

2: When someone needs blood, maybe when he/she is sick and he/she goes to the hospital and the doctor says they should him/her blood; they would send her to the place where they test the blood then they go and check his/her blood. If some is there, then they transfuse some to him/her. And if some is not there, they would tell him/her that he/she should call his/her family members then they check their blood and see; the one with a matching blood group, then they would take and transfuse it to the sick one. And if some is available and they give to him/her then later his/her family members come to replace it; that one too, they could also do it like that.

For me, the biological samples what I have heard is that it is only blood which is stored. I haven’t heard that there is any other which can be stored apart from the blood.

7: Please biobanking is good because the act of storing blood is good. Because if someone is sick and he/she needs blood urgently and we don’t have a blood bank, they person; it may happen that he/she could die because it wouldn’t be at the instant that they are going to call him house that he/she needs blood so they should come and donate some to him/her but if some blood has been stored there and it would help the person if he/she needs the blood urgently, they would be able to give it to the person. That what I know about it.

6: The biobanking especially blood bank; what really helps is that maybe then me and my sibling’s blood is the same group but maybe there’s an infection in mine, it is the blood bank people who would go and test the blood to see that there’s no infection in it before they give it to the sick person. So that also helps; it helps.

“What are the benefits of biobanking?”

8: The benefit it brings is that some is what I was saying relating to my sister. If they hadn’t gotten some of the blood, maybe she could have died but when I came to Komfo Anokye Teaching Hospital here and I showed the paper to them; and I showed her group to them immediately then they gave us some. So I see it as a benefit that every person maybe in a while when they say they are it, you have to be able to do some and help so that it would help to save other person’s life.

5: The benefit in it is that it makes us to even see the blood group that we have. Maybe as I am here either I am O or I am a B; I don’t know but the moment they would check me to go and donate the blood, it makes me to see that my blood group is B or that. So that helps us to know our blood group.

How important is biobanking to medical breakthroughs?

7: Because a biobank is a place that we store biological tissues or specifically the blood that we are talking about. We store it and keep it safe so that the blood wouldn’t be infested and for it to be helpful for our health. Because as I already said, if the person needs blood; it’s not just any blood you can transfuse to the person but I think that if the blood that we have stored in that particular place is a blood which has been stored well and there is a policy guideline for it, it can help to sustain the person’s life.

6: The blood bank helps a lot because in case of any emergency and we need blood, maybe even if it’s may sibling; maybe then he’s not even close to me to come and donate some of the blood for me but when we go to the blood bank, we would get some of my blood group to give me before later a relative would come and donate some for replacement. So for that, it helps a lot.

2: Maybe then someone is in labor to deliver, then she loses blood and they say they would need blood, if the need it urgently for transfusion; unless they go to the blood bank in search of the blood for transfusion.

1: It has yielded a lot of benefits. Some of it is that, maybe as I’m here; maybe I don’t know that my blood is infested but the moment; maybe my sibling needs blood and when they made me went there, some is not there so I have to donate some to him/her but when they tested, it could happen that then there was an infection in my blood. So with that, it would let me know that my blood is not clean or it is infected and they can’t give it to him /her. That’s all I can say.

4: it has helped to save people’s lives.

Awareness, understanding/perception of brain banking

8: Please with that, I haven’t heard anything like that yet.

6: I have heard something as such that, they can transplant the brain but for where it’s being stored, I don’t know that as we have the blood bank there is a brain bank.

“What have you heard about it?”

6: For instance, let’s say someone has a brain tumor or has a brain problem; the documentary I was watching, the brain is something like a curve so maybe then there is a particular part which is being affected and they could operate and take that from there then they replace it with someone’s own. But what I also want to ask is that, is it only a living person whose brain can be used or only sorry to say; a deceased person? Though I can’t tell.

2: I want to know that, does the doctors see that the person is going to die before they do that thing or after he/she has deceased or is it after when the person is deceased or is it as he/she is alive that he/she gives permission that you should do it?

It’s left with my one question; so does it mean that the person has to die before they remove it?

Awareness of any policy or law guiding biobanking

6: I know there is especially with blood; if someone comes to donate blood to you then you test it and see that there’s an infection in it, it’s secret and confidential. You can’t go and tell someone that when you went to test this person’s blood; theirs is an infection in it. I know that’s a policy there.

2: What I have also heard about blood donation is that, when a person donates six times; he/she is not supposed to continually donate again. Is it true?

## Can you explain what you understand by precision medicine?

6: Precision medicine is important and it helps because with my case in particular; my uncle that I’m taking care of, we have a medicine that he takes twice daily; he takes one in the morning and he takes one in the evening. There are some which he takes three times daily; one in the morning, one in the afternoon and one in the evening so you have to follow it accordingly. If you’re supposed to give him one in the morning and you give him two; you are killing him, you see. Then I also bring him for physiotherapy. When I bring him for physiotherapy, when I alight; how I bring him out of the car to sit in the wheelchair, there was a time one man at the physiotherapy showed me how I should sit him up in the wheelchair when I come and how I would push the wheelchair. So I would say that also forms part of the precision medicine because that also helps the patient you are taking care of.

8: Please with the medicine; what I see is that when you come and they instruct you that, give it to him/her in the morning, in the afternoon and in the evening as you are saying; there could be someone who would say maybe I’m going somewhere or something like that so I’m giving him/her both the morning and afternoon ones together. The time we were on admission here, we saw something like that; the person was giving both the morning and afternoon ones to the sick person because he/she would be able to visit in the afternoon. When you do that, the person you are taking care of could die or the disease would become worse again. So what’s important is for you to get time and the times that they would instruct you to give to the person in the morning, in the afternoon and in the evening; you would follow.

Probe level of awareness, understanding/perception of concept, benefits and demerits; is it important in Africa?

5: The benefit it has brought in our country; it has helped us not to as we are saying we combine the drugs; you don’t have the time so you have combined the drugs for the person to take. It has helped us to know that if we don’t follow the instructions and we don’t give them timely; it wouldn’t make the person get cured of the disease and it would still get worse so it has helped us to give the drugs to the person well and at appropriate times then it would make the disease affecting him/her get cured. So it has helped us not to get overdosed; in the person’s blood, the drugs wouldn’t be overdosed in it. It makes the drugs work as it’s supposed to be.

8: Please what I see is that; for instance, if maybe the person, the disease attacks him/her at home and you haven’t brought him to the hospital yet; you are human, you that you are taking care of him/her you would see the difference maybe when you come and they start to give him her some medications. When he/she is at home, you would realize that the person can’t do anything of her own but when you come and they give you drugs; and with the drugs; what I see is that when they prescribe; maybe when you give it to him/her for about 2-3days and they don’t see anything, they could let you go and buy different ones. So you could see that some changes would happen to the person.

6: With the precision medicine; maybe when you are taking care of the patient and you don’t follow the dosages of the drugs, you make his/her disease even worse. Sometimes you could cause death; you would kill him/her. That’s a disadvantage.

2: Maybe the times that you are supposed to take the drug and the times that you have to eat; it’s my husband who is suffering from stroke; for him, he’s able to walk. He could come to the hospital himself even if I don’t come he could come. But when we came here, they told us to check the times he would eat and the times he would take the drugs so when they asked me, I told them that he would take it in the morning 8 o’clock. So when he takes it in the morning 8 o’clock, he would take it again in the evening at 8 o’clock. So we have to make sure he eats early; so by 5pm then he has finished eating. So he would walk and chat around till 8pm exactly; for the time, it’s besides us and we check. So immediately at 8 o’clock, he has to take his drugs. So when we started doing that, it has helped him a lot because he takes the drugs timely. But at first when we hadn’t come here and the drugs he was taking were too many; we put some in water and we were doing some this and that. Sometimes with the drugs; he felt lazy taking them. When you give it to him; he would resist unless you beg and patiently entice him like a baby before he takes it. But for God’s grace when we came here, the drugs have reduced to just one. They said they have combined all to it one so he uses time to take the drug. So when they tell you take it timely; you have to try your possible best to obey.

5: One of the disadvantages about it is that; for me it’s my father who’s sick. When the stroke started, my work has been affected. It can’t even attend to work because of the drugs. Sometimes even when you are at work, you have to be fast and leave work; maybe at that time since you went to work then it would the time work would be coming in and you leave the work then you would see that; all because of the drugs. Because you wouldn’t get anyone who would give him the drugs so if you don’t go and give him the drugs; it wouldn’t be possible. So for me, one of the disadvantages it has given is that; it has affected my work negatively.

4: Me too; it’s my granny who is sick. A times when I give her food, she doesn’t want to eat. Even when you force her, she doesn’t want to eat so I’m unable to give her the drugs at the times I’m supposed to give her.

3: Me too; it’s my mother who is sick. The time that we were home; later we were in the house when they brought her. So when they brought her, she was unable to walk; she couldn’t do anything. But they brought her and when they started giving her the drugs and she got better here and after we went home; the time they instructed us is the same time we give the drugs to her. Right now she couldn’t walk though she’s not fully recovered but she could walk and bath and she could feed herself and do everything. And first then her hand was paining her, she couldn’t wear clothes with it but now she could wear clothes with it and bath as well unless somewhere which is far that she can’t go; but when you guide her, she could go because the time they gave her the drugs at the hospital and we went home is the same time we’ve been giving her till now.

7: One disadvantage also is that, they would give him/her the medicine and when they take him/her home; they add traditional medicine and give it to him/ her that they performing traditional way of treatment. Aside the orthodox medicine that he/she uses, they also add traditional medicine to it. And with that when they mix it like that, the medicine take effect appropriately in the body; like the orthodox ones that the hospital has prescribed for him/her, it wouldn’t be able to take effect in the body so it makes the disease gets worse. So the disadvantage in it is that, with the medicine if we don’t stick to the orthodox medicine which has been given to us and we do addition of another traditional medicine; it wouldn’t make the disease get cured like how it’s supposed to be.

“Do you think the precision medicine is important in Africa?”

5: For me, I see it to be 50:50; in some instance it would help and in another instance it wouldn’t help. As I was saying mine for instance; my work and the giving of the medicine as it’s timely; my father takes some at 11 o’clock; sometimes 11am then he takes some at 6am and sometimes too 11pm. So even with that time; I don’t get time to sleep. When you are asleep then you wake because you have to go and give him the drugs. So in Africa here, it’s difficult.

8: Please though what you are saying is important but I see it that with the person, the disease has occurred; it has already affected him/her, what would you do? And if you don’t attend to it and it gets worse, it lies upon you because with my mother when it happened she couldn’t do anything. If I don’t do it, who would come from outside and do it. I pick nurse to the house for them to come and take care of her; healthcare, they were in the house and we pay them but still you have to be attentive on her. If you don’t pay attention to her, no one will. For them when it gets to the difficult times, they could go. That’s what I see; when it comes; the stroke disease when it affects someone unless you that you are close to him/her. He/she would drink water; he/she can’t say, he/she would go to the lavatory; he/she can’t say; there nothing he/she can’t say even if he/she wants you to feed him/her and he/she is satisfied, he/she can’t say so if you don’t help that person; it would be difficult. And they say the traditional medicine; if you think you would stop the orthodox medicine as he was saying and you would give him/her the traditional medicine and you think the traditional medicine would help him/her and if that would help; it’s also good.

1: The little I would also say in addition is that; the person, it’s his/her life which is important now; whether time or whether the cost of the drugs or whatever. Though it’s also inclusive. If with all these and the person would retain his/her life, that’s good than to leave him/her; we wouldn’t give him/her drugs to take, we wouldn’t feed him/her; we would leave him/her there whilst we know that he/she can’t do anything. As my mother is there; no one is around her. Even when I was coming, I sought for an outsider and I left her in the person’s hands. As I’m sitting here, my concentration is back home because I know there’s no one taking care of her. So with the person to me, whether my work or whatever; I don’t care, it’s my mother’s life which is my concern right now. Because there’s no one with me, it’s only my mother so right now if she’s not there; what would I do. So that all I could say.

6: With the precision medicine, it is important because it’s my uncle who is sick but when we brought him to the emergency, he was weak; he couldn’t do anything. Even when they were discharging him, he was fully cured but I exercised patience; something touched my heart and I said that it’s my uncle I would save him. I didn’t see what happened; when they discharged him then that thing touched me. So with drugs and his feed, I took care of him of all that and I brought him for physiotherapy as well but when I followed the time and his feeding; he was using a walking stick recently but now he doesn’t use the walking stick again. He gets up to go and bath, wears his clothes and does everything himself. So right now it has even made my work time increase more because he himself checks the drugs and take. Even when I’m not around; as I’m not around, he would take it. In the evening when I’m not around, he would take it. So for that I see it helps if you are able to follow the time and God intercedes, I believe that he would get cured for you to also get time to do whatever you also want to do.

Can it be applied to stroke disease?

Source of information

Awareness of any policy or law guiding precision medicine

6: For me what I can say about it is that, for instance maybe they have prescribed the drugs for him; he’s supposed to take one tablet; one morning, one evening then you give him two in the morning, two in the afternoon instead; you are killing him so with that, my opinion is that you could even be surcharged because you are playing with the person’s health.

Belief/thought/opinion relating to precision medicine

6: My opinion is that; it helps a lot. I have a belief that when you follow it and you take care of the person properly; sorry to say, with the sick person it’s not only the medicine which could cure him/her; you also that you are taking care of him/her; how you treat him/her; how you talk to him/her, you see; the response you give to him/her; all that could make the person get cured. So with the precision medicine, for me when I followed and I believed in it; to my uncle, it has helped me. It helps a lot.

## What do you understand by brain donation for research purpose?

Probe for level of awareness, perception, sources of information, perception of complexity of the procedure, benefits of brain donation, misconceptions, personal willingness to donate

Cultural, social and religious belief on donating brain for research purpose

What factors inhibit brain donation? Probe for cultural and religious reasons, peer values, parental influence, level of awareness, legal issues involved, knowledge of where it can be done, familiarity with medical and research settings

What factors promote brain donation? Probe for cultural and religious reasons, peer values, parental influence, level of awareness, legal issues involved, knowledge of where it can be done, familiarity with medical and research settings

## What do you understand by blood sample donation for genetic research?

8: Please what I see is that, what it has helped for the improvement of research is that; we would all go and donate blood as we are here but maybe then there is a disease which maybe they haven’t seen yet; but through that they would know there is a disease like that. So that also helps them to know different disease which would; for instance, HIV, if it’s even about 10people; they would know that right now the disease is in the system which is spreading. That’s how I see it.

6: What I also see is that it helps. It has helped a lot because there is a disease which occurs in the blood and if it’s not blood donation; because when you donate, they are going to test the blood. So that even makes us to know a particular group and the kind of disease which is often found in their blood. So with that the blood donation helps a lot.

What are your thoughts on blood sample donation for research?

Source of information

8: Sometimes in maybe news, they could say that maybe today there is going to be a blood donation and when they did it; those who came to donate, maybe some of the people have this kind of disease. So that what made me know that there’s something like that.

6: I’m someone who reads a lot and I also watch documentaries so that’s where I pick that information from.

4: Just recently, I went to donate blood for my sister. They checked and they did a lot of tests and it make me know that. They first test to see that you don’t have any infections before they donate to someone else.

Uses of blood sample donated for research

6: For research, what they use is for is that, it helps us to see that maybe; as I was saying at first there’s even a group which it helps them to know that this type of blood group; this is type of infection is what often occurs in their blood. So that helps for them to know maybe that disease; for instance, if you are this group; this disease could affect you or its causes and its preventions are all something that it could help.

7: As he said, it helps to examine the blood to discover the infections and aside the blood groupings; that maybe A or AB or O and the B. Aside the blood grouping, it also makes you discover the infections which are in the blood so that the person that they are going to do the transfusion for; they are going to give the blood to; they wouldn’t give it to because the blood that they are going to give to him/her, there is an infection in it. So that’s one thing it also helps.

Cultural, social and religious belief on donating blood for genetic research

8: Please what we know is that; a church called Jehovah Witness; for them they don’t donate blood. They don’t allow that when a member is sick; their member would donate the blood for another person. So for them when it happens like that, they don’t allow. So even if the person would die, he/she would die because they don’t allow that.

6: It’s true. We have lots of beliefs. Apart from Jehovah Witness, there’s another church; that’s their belief. That they don’t want someone’s blood to be donated to them.

“Can you give an example?”

6: Apart from Jehovah I know that True Faith Church (Gyedi Kokoo); we have different types. They have one type of the True Faith Church members who, for them; they don’t allow that. They wouldn’t receive transfusion. And also even if he/she would die; they would overlook for him/her to die than for someone to donate his/her blood which would sustain him/her.

Awareness of any policy or law guiding blood sample donation for research and storage

6: For me what I know about it is that; they have for instance maybe I have come to donate blood and you have gone to examine it then if there is a disease in my blood; you can’t disclose it to a third person. I know that’s against the law. I know that if you see an infection in my blood whilst I came to do the donation, I know you can’t go and tell a third person that maybe when this person donation his blood; maybe this disease is infested in it. I know there’s a policy guiding blood donation.

## Share with us your opinion and thoughts about blood sample donation for stroke genetic research.

6: With that it helps because how the stroke disease is becoming very common these days and causing people to die and affect people, if blood is being donated for the use of stroke genetic research; it would help. It would make us prevent it and it would also help to know the type of medicines which they would improve and give to stroke patients which would cure him/her early.

Willingness to be involved in such research

8: Is it as in if they are going to take my blood sample to check whether stroke or they are using it to help the stroke people; those who have stroke? What I see is that; are you using it to help someone who has been affected by the disease before or what?

6: If it happens that we are donating blood for stroke genetic research; as I am taking care of a stroke patient, I know the problems the person is going through so I would be readily involved in it for me to donate mine for them to use it for research to help treat other people of their diseases because it’s a disease which is very worrisome.

8: Please as my brother said; for us who have been affected or have seen relatives who have been affected, it’s a disease which is worrisome so if something like that happens; we have to help and it would help so that later the people affected wouldn’t increase in number; it would help a lot.

2: As my sister is saying; the disease is a disease which is dangerous; it’s dangerous and it’s also very worrisome so as I’m sitting here if right now they say they are going to research to find out if I have some or if in future I could be affected; I would hurry up and go and do that research. I wouldn’t sit and say someone should go first before. I would be in a hurry to go and do that research because the disease is dangerous and it’s very painful.

1: As I wouldn’t want to see my fellow affected with that disease because it’s not easy. Even with me that I am not sick but just taking care of the person; it’s not easy for me so if I hear that an activity like that is happening, I would be happy to be able to go and help for that activity to go on.

5: For me the difficulty in it would make me go in hurry to go and do because as I was saying, it’s the difficulty in it; the time wasting in it so it would make me be in hurry to go and do that thing.

What do you see as the barrier(s) that could hinder your donation of blood sample for stroke genetic research: family member, cultural and religious reasons, peer values, parental influence, level of awareness, legal issues involved, knowledge of where it can be done, familiarity with medical and research settings?

5: As you were saying there could be someone who if the education hasn’t gone down well for him/her to see that the stroke; there could be someone who doesn’t even know there’s a disease called stroke and there could be someone who hasn’t even met someone being affected before but for us who have seen someone affected by stroke; we know the problem in it so you the health workers you have to send the education down; you should bring it to various communities for people to see. You have to create the awareness that the stroke disease is dangerous and this and that so when that comes in; it would make people; if they are donating blood sample for stroke genetic research; everyone would get involved. What I see is that; it’s the education which hasn’t gone down.

3: Me too what I know is that if it’s vital to donate my blood for someone and what my siblings or family members or my friends would be against it; I wouldn’t listen to what someone would say. I would donate because my next sibling, anytime she could go and donate her blood. My father has cautioned her several times but she doesn’t listen. She could come to the hospital herself and donate but when she returns and you complain, she wouldn’t mind you. So for her, every time she donates. So she doesn’t care about what someone would say about her donating. She said for her to donate to help someone; that one is good for her than for her to sit down. So for her every time she could donate.

“Do you think knowledge of where it can be done is also a barrier?”

6: That can also be. It could happen that maybe it’s Komfo Anokye Teaching Hospital that they are doing it; the person maybe where he/she would come from is very far. He/she would have preferred to come but when her/she consider the distance; that could also be a barrier to someone for him/her not to be able to come. But when it’s close to him/her, it would make him/her get involved.

What do you perceive as benefit(s) of giving blood sample for stroke genetic research that could promote your willingness to donate: cultural and religious reasons, peer values, parental influence, level of awareness, legal issues involved, knowledge of where it can be done, familiarity with medical and research settings?

What can you say about your family member or other members of the community willingness to give blood sample for stroke genetic research

8: That’s what we have already said; it’s not a bad thing that you are going to do so you would advise the person and tell him/her that, go and do it. It’s not a bad thing because you are using it to help something. Maybe you wouldn’t see the importance of it today but maybe your blood that you would donate; maybe it would get to sometime maybe they would examine it so that the stroke disease they could do something to prevent it that it wouldn’t affect us anymore so you would tell him/her to go and do.

2: I would first campaign about it if it happens like that. I would first inform people about it because the disease is dangerous and tell them about the experience I had; that if they see someone with stroke, they should study how his/her condition is. The person is very pity so we shouldn’t let it occur and increase before we work on it.

5: I for instance if I’m there and my sibling come and tell me that he/she is going to donate some; I would motivate him/her that he/she should go ahead and go and do it. It’s motivation that I would give him/her.

What could be done to make you and more people give blood sample for research: mass media, husband consent, family consent, donors group, peers?

8: What I see is that; today, concerning television, radio and other things; today it’s not something which is scarce or even at the hospital here; maybe you could print some posters for maybe those coming to the polyclinic and the down there then you print some posters and give it to people so that it would make people aware. You could do those things so that people would be aware that something like this is happening. People could get involved.

6: It all balls down to education. There could be someone who doesn’t know how stroke is and he/she doesn’t know the problems it causes; time wasting, financial issues, a whole lot of things; he/she doesn’t know. So if the education goes down for the person to see. If they advertise about it that maybe there’s going to be a blood donation exercise to do stroke research because he/she has heard what stroke could do; they would even come before you who are going to initiate the exercise come. So I think the education would help.

## Tell us what you know about informed consent?

6: It’s all about education. How you would educate about how the disease is happening and the problems it brings to the body and how it’s increasing. So when you take time and explain it to him/her; even if he/she hasn’t heard about it before or he/she had made up his/her mind that he/she wouldn’t do it but when you explain to him/her and he/she understands it then sees how the disease is; I know that he/she is human, it would change his/her mind for he/she to go and do it.

What do you know about the consent process for genetic research?

2: As I was saying; for all of us here, I believe that it’s not impromptu that we knew something is going on here and we are coming to do it; it’s with the help of someone and he explained to us on why we should come and we also saw how important it is for us to come that’s why we also worked on it and came. It’s not like we just did so that how we would if we want to help others; we could take the same procedure to communicate with them because as I was coming, I was calling the man and he was also calling me. That’s how you own also went on and we came to reach here. So that’s how we are supposed to do it so we can use it to work on the disease.

6: what I know is that; you would talk to the person. So for us here, this man talked to us and we understood before we board a car to come. So I see it that when you talk to the person and he/she sees how important it is to the disease they are doing the research about. If he/she hasn’t seen some before and you explain it for he/she to understand, he/she would give his/her consent or he/she would agree with you then he/she would also bring the little knowledge he/she has to come and help.

Types of inform consent preferred (broad, restricted, tiered and dynamic). ***Facilitator to please explain each type to the participants

8: For me I would go for the dynamic consent. That’s what I think I can do.

7: I would go for the restricted consent because with that it would be like as I have accepted the consent; it would be between the person seeking the consent and I so that I can have power over it and it wouldn’t be in the public domain or it wouldn’t be disclosed to people but it would be something concealed so I want the restricted one.

6: I also want the restricted one because the purpose for which we are using it for is what I want it to go and perform and it wouldn’t go anywhere else or after you are done with the purpose; you wouldn’t take it for another purpose. So for me I want the restricted one.

5: I want the broad one because with how we are talking about stroke genetic research; we have to make the consent and after the consent, we have to take it to places for people to understand what stroke is about. So for me it’s the broad one I prefer.

4: I want the restricted one so that I would know how to talk to the person in patience for him/her to understand me.

2: For me I want the broad one because it would be like you are advertising for everyone to hear about what is happening but if say for you, you are; guess it’s not anyone who would go to the internet to go and see what you are doing there. Am I lying? I for instance I wouldn’t go there so I wouldn’t see what is happening there but I want what they would say for everybody to hear.

1: I also want the broad. I would want to be able to share the information. It would help others. Maybe then someone have no knowledge about it but if it gets somewhere for people to hear; that’s better than for me wanting to keep it. To me it wouldn’t help me.

Reason(s) for their choice

Person to be involved before participation

Data use in the incident of death and why

7: I would believe that the consent form that we signed is a witness so even if I have passed on; the signings we did shows that unless you seek for my consent before you can do anything. So even if I have passed on, the signings we did is there so you don’t have the right to use it for whatever you want. For instance, it’s like someone being sick and they are going to perform a surgery on him/her at the theatre and they have signed consent forms; if the patient doesn’t understand and the doctor uses his own will to do anything without the patient giving the consent; he could be held responsible. So even if I have passed on that consent forms that we signed serve as witness that this is what we agreed on so he doesn’t have the right to use it for anything else.

Support for generic consent for community

8: In order for you to share the information with others I would support it.

“Why would you support it?”

8: Because it not something which is shy to do; there are certain things which is shy to do but this is not something which is really shy to do.

5: All these that we are discussing and the research are all about our health and for good health, everyone needs it. Everyone in the world need it so if they need information about health and I give it to you; there’s nothing which prevents that.

“Does anyone has anything else to say?”

6: To me, it depends on the purpose of the information; what it’s going to do. So if I consider what it’s going to be used for and to me, if it would help everybody; I would consent it.

## What is your opinion on storage of blood sample and blood fractions for genetic research?

6: when it comes to the fractions; they are not the same. There are differences. That’s my opinion. So when it happens that we are storing them; maybe we have kept red blood cells here and we have kept the other ones too here. So when we need the red blood cell, we know where when we go we would get it. So that’s my opinion. It also helps.

Opinion on storage of blood sample for future use in genetic research

## Tell us what you know about sharing of data, blood/blood fractions, brain images (CT scan/MRI) as well as brain tissue samples

The reason why we share information or we collect data is that when something about it occurs; we could go for the data or the information and what it contains that can help us about the thing then we use it. For instance, with what we are doing; if you gather all, it’s a data. So maybe if you want to do something about stroke, you would go into the data to go and the opinions of what the stroke caregivers said then you can share with others for them to also pick something out of it.

Opinion on sharing data, blood samples, brain images (CT scan/MRI) or brain tissue samples with another researcher locally and internationally

6: I agree with that because that would also help for those who are also at other parts of the world to see what we are also going through here. So that in case maybe they are also doing some research or if they have any assistance that they would also come and help us with; they would also come through that they would also come and assist us.

Commercial or non-commercial use of stored data, blood/ blood fractions, brain images and brain tissue.

6: My opinion about it is that, when I go and donate blood; I donate it for free so if someone also need it then he/she is coming to collect it, though we would keep the blood bank because we have to maintain the place or the equipment we use for keeping the blood; we maintain them. In case it’s even faulty, we repair it or we replace so if we would collect something little which is not much to keep the bank running. You see but it wouldn’t be like; bring money to purchase the blood. But if you are collecting something little to keep the bank running, that’s also good.

1: What I can say about is that; it has to be optional. It’s not supposed to be compulsory. Maybe then the person; when he/she was coming then there was no money on him/her but maybe if he/she doesn’t get the blood to send; the person is dying so if he/she get there and forcefully if you don’t get the money, I wouldn’t give it to you; that’s not appropriate.

3: What I also know is that; with the blood in case you are sick and you need it, when you are going for it; they don’t just give it out like that. They collect something little before they give you the blood so if you don’t have something little to pay, you wouldn’t get the blood. You could die. For instance, maybe you to need the blood and immediately when you go they write is for you that maybe go and collect the blood; I don’t know it as such. I know there is something little you have to pay before they give you the blood.

“Do you think it’s appropriate for them to collect that little token and why?”

3: I don’t see it as appropriate. Because when I’m going to donate mine for them to store it; I donate it for free. I don’t collect anything so why is it that if someone also needs it and he/she is coming for it then they say unless you pay something little before you get it. That doesn’t help.

5: Also, my opinion about it is that; as we are giving it out to everyone for free, if we give it to everyone for free; the time they were mobilizing people to come and donate, they involved money to mobilize the people and even with the equipment they sent; with all monies were used. So if they don’t take some of the money to replace to get a fund; in case even what the blood stored in gets faulty, what would be used to repair it; it’s all about money so if they collect something little; it all helps. It helps for them to sustain the thing to improve.

3: What you are saying, I don’t agree with you because you said the blood when we went to donate; me for instance, I donated it for free. I didn’t collect money from anyone. And when someone need some and the person was going for it, they said unless you pay some little; they wouldn’t mention the specific amount so when you say; when you were coming to donate, you donated it for free and if someone is going for it; he/she should pay money because maybe when the machines get faulty; they would use it to repair it. Why is it that when I was coming to donate, you didn’t pay me but when somebody else need it, you are collecting something little before you give him/her the blood. I know someone whom when he was sick because of money he didn’t get the blood; he passed on. That thing could be worrisome. They wouldn’t state the specific amount but it’s compulsory that when you don’t pay you wouldn’t get it. Maybe then the person doesn’t have some but he/she needs it. That doesn’t help.

5: My opinion also is that, even health service delivery is not free. Health service delivery is not free. It’s all about money. When you came to donate the blood, it could happen that they gave you some soft drinks. They used money to purchase all of those drinks for you to take. So with the money, they can’t say that they wouldn’t collect. And what I also know is that if you are a donor then you show your card that you are a donor, they give it to you for free. Even if it’s your relative, they would give it to him/her for free; only if you show your card that you are a donor; they would give it out for free.

## Share with us your thoughts about return of individual research results and incidental findings

What are the ways that you think one can receive the results of genetic research?

8: If you examine the person’s blood and you find an infection; maybe you call him/her to your office one on one then you tell him/her that maybe when we examined it, there’s this disease in your blood. Maybe this is the drug the when you take, it would help you then it would make the person be aware.

6: For me, my opinion is that; it is secret and confidential. It’s between only the two parties because if you have seen an infection in my blood, you have to tell me; you can’t go and tell any other person about it. So it’s between the two parties.

What are your thoughts on returning individual research results and incidental findings?

8: That’s what I was saying; maybe the person even doesn’t know that infection in his/her blood. Maybe he/she doesn’t know. Though it’s difficult but you have to tell the person.

“How?”

8: What I’m telling you is that, maybe as you are chatting with the person then you say maybe the disease when I went to check; maybe this and that. You chat with the person a little then you tell him/her that; “it’s not easy oo”; he/she would ask you why then you tell him/her that, this disease that we are discussing; what would you do if it affects you? Then you ask that person that; maybe if some affects you what would you do? So if you listen to the person’s response then you tell him that when I examined yours; there’s some in your blood. He/she said if it happens to some this is what he/she would do. So it has happened to him/her; you give him/her a clue that there’s some in your blood so what would you do?

4: You would talk to the person and advise him/her then in a polite way you tell him/her. That’s all that she has said.

2: My uncle got sick for us to bring him here; then they said he has been affected with a brain tumor so there’s no way he would survive. So some of the doctors told us that if they tell us to go and do any test; we shouldn’t go and do it because the person wouldn’t survive. And if they prescribe any medicine for us to go and buy, we shouldn’t go and buy it. But it’s not all of us who came there that they informed. They look for the person maybe with the courage you see; who can keep the information; who for instance when they bring the test he/she would collect then he/she would say there’s no money we won’t go and do it. Whether there is money or there’s no money; he/she has collected and said we wouldn’t go and do it. So my elder sister is the one who happened to be the one; some of the doctors, she even asked them that the way she see her brother lying there; how’s he? Then they told her that it wouldn’t be possible. It happened here at the emergency. So when they told her that it wouldn’t be possible, they told my sister that; she should have courage for them to treat him until that thing would happen. So it’s not like just everyone who came there that they informed. They looked for someone whose heart could take that information. So me for instance I wasn’t there; my sister called me to tell me that they said my uncle wouldn’t survive then I immediately kept quiet and I told her to also keep her mouth shut for us to pray until he passed on. So they look at the person’s character and the courage he/she bears. Sometimes with the person you can’t tell him/her that, this is the disease which has affected him/her unless maybe you call his/her relative then you tell him/her that this is the disease your sibling has. Maybe there are drugs but we don’t know how we are going to tell him/her. He/she wouldn’t get the courage so we should start with something enticing for his hear to take the information before.

Opinion on desire for feedback of research results and incidental findings

What are the challenges of returning individual results?

6: The challenges in it is that; it’s not easy to disclose the news to the person. It’s not that easy so you that you are going to disclose it have to find a way. There could be someone; there are challenges in the way; maybe when you tell him/her; he could faint or something like that you see; he/she could get shocked or something like that. So I think that is also a challenge on how to disclose the news to the person.

4: As the woman said; it’s not everyone who has the courage to so unless you call his/her relative then you inform him/her that maybe this person; this disease has affected him/her but we have medicines there so; then they console and advise him/her before the inform him/her.

Ways they would prefer to get feedback: phones, e mail, letters, feedback by a healthcare worker? a researcher? or a clinician?

5: It’s the researcher who has to go to him/her and sit down with him/her to talk because when you send it through his/her email; the moment he/she reads it; he/she is there alone and you see as human when you are there and something borders you, you could harm yourself but if someone is with you; for instance, if something borders you and discuss it with someone, you could get over it but it’s only; it borders you a lot. So you the researcher if you are able to get the courage and go to the person to share and if you are able to tell him; that’s better.

6: My opinion also is that, you could let a counsellor go and talk with the person. Through that the counsellor know how he/she would psyche the person’s mind before he/she would relay the news to him/her.

What are the ethical, legal and social issues relating to returning individual research results and incidental findings generated by genetic research?

6: The person has already been affected by the disease so no matter what he/she should hear of it or his/her relative should hear of it so that they would know how they would treat him/her of the disease. So the ethical issue relating to it is that how you would give the information to the person is very ethical; you see. Maybe if it’s a disease which is very serious; you would involve a counsellor then he/she finds a way and give the information to the person.

6: My opinion is that; it has to be back by law because if you have conducted a research on me; it doesn’t have to go to any other person. It has to come to me so you would find a way the thing would come to me confidentially. It doesn’t have to pass through anywhere before it gets to me. And you can’t also send anyone to bring it to me.

## Explain your understanding of Biorights

6: What I understand about it that; if you could remember in the beginning we talked about consent so without my consent you can’t take my biological samples for anything until I agree and given you my consent before you get the right to do so. Even if you take my blood sample I have to give you my consent before for you to tell the purpose you are going to use the blood sample for so I give you that right before you could use it.

How much control should/can individuals have regarding how their biological specimens will be used in research?

6: With the entire research, I can’t follow but so far as I have given you the consent or the agreement was you were going to use it to conduct a research purposely for stroke though I can’t follow but later if I find out that the thing didn’t go purposely for stroke but you used it for any other thing; I would hold you responsible.

What rights do/should individuals who provide their specimens for research have over their specimens, how they are used in research, and any profits from research discoveries made possible from them?

8: How I see it is that; with everything the person has to be involved and ask him/her that maybe when you finished, you want to use it for other purpose; can we do it? Then he/she tells you whether to use it or not to use it.

“Do you think any profits from research discoveries made for them?”

6: I believe it has to be made known to him/her because he/she also contributed so for me; with any outcome, you have to inform the person either positively or negatively; you have to let he/she know.

How should autonomy rights be best balanced with societal benefits that derive from the use of human specimens in research?

6: It has to be balanced in a way which would help others and I also who contributed too; it would benefit me. It shouldn’t be a one surfaced sided thing. It should be balanced.

## What is your opinion about governance and regulation of biobanking?

Need for ethical committee approval on future use of stored data, blood or brain tissue resource for research

6: I think so. When the committee comes in so that; because if there is a committee in anything, there are regulations governing it so the regulations that the committee would bring if you go beyond that; they would sanction you. Because if you leave the individual to use his/her own will to do; he/she would use his/her own will beyond measures. So when there is a committee it’s good.

2: When there’s a committee, it’s good because it wouldn’t be one person’s decision. Everyone would give his/her opinion then they would consider and work with them.

Need to set up a regulatory board

6: I think so. There has to be someone to also check the committee because if the committee also finishes its work; it has to go to a regulatory board for them to also make sure that everything is in order.

## Explain possible intervention for implementation of biobanking

6: It’s all about education. We have to educate the public for everyone to; it’s not everyone who even knows about the biobanking. Maybe he/she doesn’t know about it. He/she hasn’t heard of it before. If we hadn’t come here, I wouldn’t have heard about the biobanking. I know there is only blood bank but there are brain and other things. So it all balls down to education. We have to educate the people for them to understand. When the people understand, I think that would also help for the biobanking to be implemented.

“Apart from education what ese can be done?”

4: Advertisement.

8: Data distribution.

What suggestions do you have that can help raise awareness and improve attitude towards blood sample or brain donation for research and encourage people to adopt the practice?

8: Example is what you are saying. Maybe you tell the person that when you do; I would pay you; I would give you this amount of money. You see for human, when you let him/her knows that or the thing when we finish; the benefit that would come out of it is this; so that the person can involve himself/herself well.

8: As you are saying; maybe when you organize campaigns; maybe they do it for months or maybe every one week then they would be roaming. Maybe today; we would go here or today we would go Stadium so that people would follow. As we are roaming, then people would be aware.

## Any other major concern or recommendation on use of blood or brain tissue for research in Ghana?

2: I want to know what causes the stroke disease. What causes that disease; through what it came from? So that when we are advertising about it then we add those to it that we should take care of ourselves from these things because those things brought this disease.

3: What I know is that; what causes stroke is someone who eat fatty meats. There could be someone who eats those meats and someone also doesn’t eat those meats and doesn’t get that disease but he/she hasn’t gotten some of that disease. So what really causes the stroke?

4: I also, it really surprises me is that; it’s someone who is very active who gets affected with stroke and those who are lazy (inactive) don’t get stroke. Why is it that those who are active get stroke?

5: The problem I have with the health system in Ghana here is that; particularly, when you come to the hospital the way the workers talk to you is not something encouraging. So with that if they could improve it for us; it would help for our healthcare delivery to be successful.

6: What I have to say is that; it’s about lifestyle. How we eat and other things that we take into the body; we have to consider all those things. We have to consider all those thing and alcohol intake and unnecessary things. If you avoid yourself from them; that would also help us to prevent those diseases.

“If I would ask again; what would you recommend on use of blood or brain tissue for research in Ghana?”

6: What I could say about it is that; especially blood for instance, you see the donation is for free so if whether the person has donated some before; maybe as I haven’t donated before, my relative has donated some before. So when it happens that we need it, they should let us pay something little. It shouldn’t be compulsory that go and bring this amount of money for us to give you the blood before you go and treat yourself. So they should make the blood donation accessible for everyone to have access to the blood in order to use it to get good health so it would help us.
