# Supplementary material for: Research participants’ perception of ethical issues in stroke genomics and neurobiobanking research in Africa
Source: PLoS One. 2025 May 6;20(5):e0292906. doi: 10.1371/journal.pone.0292906 (PMC12054916; doi:10.1371/journal.pone.0292906)
Supplement: S3 File — (ZIP) [file pone.0292906.s003.zip › Files for PLOS ONE - updated March 2025/KUMASI_Stroke Free Controls_ FGD.docx]

# **FOCUS GROUP DISCUSSION TRANSCRIPTION FOR STROKE FREE CONTROLS**

## Tell us what you know about genetic research?

4: The DNA, what I know about it is that when someone and a woman gives birth and when it gets to a time and he claims that the children are not his children; and when we check the we see that thing.

2: There could be someone whose health isn’t okay. He/she is sick but then he/she himself/ herself doesn’t know that he/she is sick so if a small disease affects him/her; he could get stroke or he could die at the spot. So that is what I know about it. Something like malaria, jaundice and others; some could affect someone instantly and for that if the person doesn’t check and goes to the hospital early or if he/she doesn’t get to the hospital early; he/she could lose his/her life.

Have you heard about it?

7: Yes

What do you know about it?

7: I think for research it helps to attain new findings, so that anything that is hidden; we could find everything. Let me take for instance, like the DNA or like the genetic research; maybe even with you then there is a hidden disease in you but through the constant hospital that you would go and the screening that they would do for you; you would notice. And research also helps us to get new findings so that, the year after or maybe after some years; we would be able to find out certain things in the world.

Tell us about any experiences you or others you know have had with participating in genetic research

7: I think for research; it has helped in a way that if maybe even with some disease; someone’s own that we’ve seen; it is able to prevent some people own. I would use someone as an example; the person, for almost about a week then he wasn’t able to remove his bowels; his belly has also swollen but the doctors said that; after a week he was able to remove his bowels. The doctors said they had wanted perform an operation on him to identify the cause so the if someday when someone brings something of that sought, it could help them so that they wouldn’t operate on the person. I think that has also helped. So for research, I think it’s very important.

What do you know about genetic research in stroke?

6: Doctors made us understand that when the stroke is coming; when you are seated, maybe your leg could become very rigid. When it happens like that, you have to go and show yourself to a doctor. Again when you see that, exercise could also help a little. That is what I know about it a little.

3: I took my own child to Komfo Anokye Teaching Hospital, the child was lying down; I don’t know what happened to him; at once he wasn’t talking. It made me send him to Dr. Boaitey for him to treat him. After he had finished treating him, the doctor said that we should take him to Komfo Anokye Teaching Hospital. So when I took him and the finished with the test, they told me that the child shouldn’t eat okro. He said with what happened to him, if he eats okro; it would happen again. So I agree that the research that they do has helped me in person.

What do you think are the roles/benefits of genetic research in medicine?

3: Why I’m saying this again about research is that; in the country we all agree that aside the supremacy of God, our doctors are second. It’s my opinion I am saying.

“Why do you say so?”

3: Why I am saying that is that, some disease could occur; me for instance I don’t have knowledge apart from the doctor who could determine that this disease, unless I give you drugs before you can get cured or unless I give you codeine or APC before you get cured. So with doctors apart from God, they are second. So we have to let our focus be on doctors so that the help that they have they can do the research for us and for the research to be well done. And anyone who something is wrong with has to take it to the doctor for him to check so you know where you would take it.

7: Like I was going to say about the stroke comment that 6 made; what I also know is that, doctors have made us to understand that, late eating; when you eat at night, it has to be that after you have finished eating, it has to take you at least about 4-5hours before you sleep but if you didn’t eat early and you couldn’t; immediately after eating then you’re going to sleep. I think if you that constantly, I think it could also bring the stroke.

5: With the issue of this stroke, I remember that about 4years now; we went to Yawso and we were eating at a chop bar then one man; for him, he was older than us; let’s assume he would be about 58years going and a doctor, one man was sitting beside him; that man was a doctor but for us, we didn’t know. And he called that man then told him that the food that he’s eating, that’s not the food he’s supposed to take and he often eats foods like these; he would suffer from diabetes, stroke could set in and hypertension. The man told him that then he passed and he also told him that he should stop eating at nights. He said at by 4-5pm, he would have finished eating but in the evening he could get some soft drinks and take then sleep but he shouldn’t eat that time and that was the time the man too was eating. We return around 11pm and when fufu has been prepared for him the he would eat. It wasn’t even up to 7months then he got that hypertension and diabetes also added up. So when he went to the hospital, what the doctor told him was that if he had delayed a little, stroke would have affected him. So I could see that late night eating is also one factor that we have to avoid. It could also bring that disease.

“So what benefits have we derived from genetic research?”

5: some of the benefits is that, doctors always warn us that we should stop late night eating so when we see to it and put a stop to that, it would help us. Me for instance, when it’s 6pm; foods which are heavy, I wouldn’t eat. I could see that when I started doing that, it has helped me.

2: the opinion I also have is that, as my brother was saying; it’s very true. There could be someone who, it’s in the night that he/she eats. I like this at first by 3-4pm then I had finished eating. But because of the work that we wake up early to go and do, I could sleep for a long while, by the time I return; maybe around 6pm to 6:30pm in the evening then now that I would be awake. The children had finished preparing food; maybe the food that they would prepare; even with banku, I don’t take it. The food I eat is rice and fufu. Even with the fufu when it is 6pm, it’s not something that I would eat but the rice; when I eat the rice, immediately I finish; then I feel sleepy so I have to go and sleep. So when I studied for some time now, if I check the situation I was through; I could see that it wouldn’t help me. At first, I could run but now if I go here and there a little then it seems I’m somehow very tired. So right now, the late night eating and other thing, I have reduced it a lot.

## Can you explain what you understand by biobanking?

2: My opinion about that is that, sorry to say but as the person was coming on earth; as God created the person and he/she was coming, his/her everything was intact the he/she brought it so for the person to die and they would say that they are going to remove his brain because of someone’s benefit in future. So as you remove and store the brain, when he goes; what brain would he/she used to answer when he/she is asked a question there? You see, secondly, as you are saying, the person; the kidney that you are talking about; sorry to say but if a person dies, he’s dead. You are removing his/her kidney and store it and transplant it to the one who isn’t dead or you are storing it then in future, you use it for research but sorry to say as you are storing this kidney; and that kidney too when he/she was coming, he/she brought it and you are removing for storage that he/she should go with none so when he/she goes; when he/she is being asked, what response would he/she give? As a human being also when you die, it doesn’t mean that you are dead; everything on you which has fallen short; even your hair on your head, even God who gave it to you knows the quantity. For us we don’t know the quantity but for Him, He know the quantity. Even if part of your body fall short; when you go, you would be asked that this thing that we gave you where is it or this that you sent on earth where is it? What response are you going to give? I for instance, I have a brother; he died. We didn’t tell them that they should cut him up and remove something from him. When we went then they had cut him up. In fact we got very angry. They cut him up and removed his heart so we made one family member asked them that the heart that they had removed, what are they using it for and they said that they are storing it for research purpose then them that no; if we the family members haven’t given you the permission you can’t do that thing so they should put the heart back. They said they had stitched it and I said if they have stitched it; they should follow the same process that they used to remove it; we did all we could and the put the heart back and stitched because when he was coming the brought it already. It is not you that you are there that; as you are also standing there and removing it, some is in you; when you go, you would be asked about it that the thing which was in you where is it? So to me the brain that the person is going to die for you to remove it and store it for future research; me in particular, left with me alone; we should put it somewhere then later we talk about it.

Probe for level of awareness, understanding/perception of concept, sources of information,

2: The blood that they say talk about it that they donate; though we are all one people, it’s the same blood that is in us. As I am sitting here, you don’t know the infections which are in my blood and I don’t know the infections which are in your blood. If we say we are donating blood to help other people; maybe then a doctor has made a test to see that, there’s an infection in that person whose blood is going to be taken. I know a doctor who did that thing; he took blood from the person; the person had HIV. When he took that blood, he himself knew that the blood that he was going to give to the person; these viruses were in it but he took it and gave it to the person. The person also had that disease. You see, it happened at Komfo Anokye Teaching Hospital that you are talking about. My elder sister was admitted there, it took her 5days and she passed on; that same place my sister was is where that issue happened. You see so it made us decided that for our family before we would donate blood, unless it is our own family member. For us going to Komfo Anokye Teaching Hospital to get blood for a family member, we have put a stop to that. For us, the house that we live in, some it not there as such. You don’t know the disease which is in that person. Someone could be there who really knows but he wouldn’t say it and the would transfuse that blood to the person then that disease which is in that person; someone could be affected by tuberculosis (TB), someone could be suffering from heart disease, someone could be suffering from high fever, someone could be suffering from hypertension but that disease maybe would not match with the blood. When you give it to the person, the person could get some of that disease.

6: The biobanking that you are talking about, it seems to me that it is voluntarily and as you are saying if it was supposed to be in goodness of it but I would prefer that before something of that sought would be done; of course we have a belief; in Africa we have a certain belief and the religion that we are in; normally when a person dies; it is in abroad that we often hear that someone has devoted part of his body to be used to research but in our setting we are not used to so like I would prefer that some education would be done for everyone to understand. It seems to me that when we do it like that it would help. I like this for instance, the religion that I am in; if it even happens like that people would say that I am an occult but maybe then it’s not like that so it would need education. It should go on more often. When it happens often, it would help. That’s what I have to say.

7: I think if the education happens more often, what it would be is that; it would bring low death rate because maybe when a person who was brought has a kidney failure because some of the kidney is available; we could do a transplant for the person early so that it could save his/her life. So with the biobanking as we are saying, if the education happens more often and everyone understands how it operates, it would amount to low death rates and it would help us.

1: The thing when you study the Bible it tells us that when a person dies then all his parts are dead. So when a person dies and all his/her part is dead and you are going to remove part of his/her body for a person who is alive; would that be possible? Guess it wouldn’t be possible. So if a person dies and his parts are dead as he/she is alive; how he/she would protect himself/herself for those diseases which would affect him/her and cause death; that is what you have to educate us on so that those diseases wouldn’t affect us and cause any death on us. Have you understood what I’m saying? That is what you have to continue and educate us on. For instance maybe this disease; if you do this thing, it would cause that disease and if that disease occurs, it would cause death so that if the person is doing anything on the contrary; he/she would be careful that he/she would not take that step because if I do, it would cause this disease and if that disease occur, it would cause death. If you don’t educate us on that and you say that you want people’s biological tissues when the person dies. The Bible also teaches us that when you die, everything dies. So if the person is dead and you removing part of his body for someone who is alive; I for instance, my family member wouldn’t agree and in our family, we don’t take someone’s blood apart from among ourselves in the family that we donate to ourselves. So that this that you are saying, let the explanations be clear for us to see how we are going about it.

3: The question I have is that, you first talked about stroke that it seems there’s no known cure for stroke and I want to make it known to you that we the herbalists, we have knowledge about it a little; when you collaborate with the herbalists about it, wouldn’t that be possible?

How does biobanking operate?

How important is biobanking to medical breakthroughs?

5: My landlord; then his child was sick. His child got sick and we took him to the hospital. So when we sent him ten they said he has short of blood so that blood that they stored; it’s that same blood that they gave to the child. So if that blood wasn’t there like the child would have died. The blood that the doctors collected and stored; it’s the same blood that they gave to the child and when they gave it to him; he became normal. He felt better so I see that there’s benefit in it. And the blood too, I see it that, it’s not immediately that when the doctors collect it then they would give it to you. They test it and check your system; before they would take your blood they test to see that if there’s no disease in you and the blood too when they take it; it’s not at the spot that they give it to someone; they store it for some months and after some months they test it again to see if there’s no disease in it before the give it to that person. So for me, with benefits; I have seen the benefits it has brought.

7: I think the blood bank is very important. How important is that sometimes we see it as because we are close it’s of no benefit. But maybe the person got involved in an accident at the time none of his family members is around and that person needs blood at the spot, that blood at the blood bank is what is being given to him. I know someone who gave birth and after given birth, she fell short of blood but because the hospital was close to Komfo Anokye Teaching Hospital, we were able to rush there so that we could collect the blood for her and save her life and the child’s life. So for the blood bank, I think it’s good and the blood that we donate, it’s not that maybe after they collect it, they wouldn’t test it and then give it to the person. They test it before they could see that there’s no infections in it. Even with the person’s blood group, it’s not any blood that is given to the person; they check the person’s blood group to see the one which matches with his/her own before they give it to him so the blood bank, I think it’s very important.

Belief/thought/opinion relating to biobanking

6: For me, for someone to fall short of blood and for someone’s own to be given to the person; my religion doesn’t go against it because you are saving human life. Because as a human being when you are sick, you have to take in medicine and if something falls short within you, you have to get some to replace it so for me I don’t see anything wrong with it. But we hear that there are some religions who are against it. But my religion doesn’t stop you from that.

4. My opinion is that if there’s no problem in doing that then we should give it to the person. If they give the blood to the person and it wouldn’t cause any problem, then they have to give it to him/her and that better than for the person to die.

Awareness, understanding/perception of brain banking

6: It seems I read a certain graphic and it said a certain a lady has given herself up to be used for research. It’s been happening but in our setting here, I have heard some before.

2: As my brother is saying, it’s true. For me I haven’t seen some before that a person would donate his/her brain for this purpose or part of his body that maybe I am devoting myself because this is what is having to me so you should take something like this. In Ghana here, I haven’t heard some before but in abroad, they do it. But here in Africa, I have not heard about it before and if it’s now they are bringing it then it’s now we are also going to know. If it has already come then I have heard about it before.

Awareness of any policy or law guiding biobanking

2: For me, I don’t really know any law that is guiding that thing. For me in particular I haven’t donated some before and none of my family members has done some before so I don’t know any law about it.

1: With blood donation, I have donated some to my sister-in-law at Komfo Anokye Teaching Hospital before. She had a car accident and when we went then the doctor said that they need blood and her husband too had not returned that’s why they called me that if I could donate. So when I went then they themselves took sample of the sister’s blood and they said that they are doing a test so when they did the test then my sister-in-law’s own and mine matched and they asked me if they would get permission to take my sample and I told them that if only it could save her and they did. It’s been long; almost about 18years now. It’s been long.

“So they didn’t tell you about any policy; all they did was you sought for your consent”

1: They didn’t talk about any policy.

“Did they tell you why they sought for your consent?”

1: they asked that where her husband was and her husband also was in Techiman which is far. When would he reached there? And I asked them that would they get some for her and maybe by the time my sister would come then she wouldn’t be having money to pay for the blood so if they check mine and check hers and it would be okay for her, then they should do it.

“Does any of you know about a law guiding it?”

7: I think there are policy guidelines about blood donation but donation and transfusion it depends on the person or relatives whether they would agree or not but there are policy guidelines about it.

“So why do you think they ask the person before donation or transfusion?”

7: So that the person, we wouldn’t give it to him/her before some day they wouldn’t return and say that it’s because we gave it to him/her; that’s why this thing is happening. If not he/she can even sue that hospital in court.

## Can you explain what you understand by precision medicine?

7: I think that for your own benefit so that the disease which has affected you, it would help you to get cured that is why. Even with the medicine if they want they could tell you that two in the morning, two in the afternoon and then two also in the evening. You would take it as such so that you would get cured. You can’t take all in the morning but they would give you timelines that you would follow so that your health would get better.

6: It seems the medicine, each has its own ability. There could be some when you go; some could be 500mg, some could be 250mg; maybe if the doctor want to give you 500mg, he could tell you that take 2x250mg in the morning or if it is 500mg; when he gets one and you take it, that’s all’ it could work. It would depend on the person strength; they would check your strength before they give you medicine.

“Is it only your strength that they check?”

And the disease which has affected you. Its medicine is what we are looking; that if you take it this way, it would help you.

“Does anyone else has anything to say about precision medicine?”

4: You see, how they could show the person how to take the medicine; if you don’t do it like that and the person go and take it overdose, maybe it could bring different problem. So for how they show us, it is very good.

Probe level of awareness, understanding/perception of concept, benefits and demerits; is it important in Africa?

6: It seems the drugs, doctors have made us understood that when you take it overdose; it is drug abuse. You see, it seems when you take it as how you have been taught; that would help well than you taking overdose. That’s what I have to say.

7: The benefit on it again is that, it would help so that it would go and cause any other disease in you when you take it as the doctor has prescribed; it would help you yourself.

2: As my brother said, it’s true. As I’m speaking, you are sick and you’ve gone to the hospital; you used to buy drugs yourself and it didn’t work and you have gone to the hospital and the doctor has checked and seen that the drugs that you took wouldn’t help you. What he would prescribe for you or what he would give you is what would help you so this time take it like this or take it at this time; so if you follow what the doctor has taught you, I think that disease which is in your life or that problem in your health, it could help you for you to also have freedom.

5: Personally, I enjoyed the benefits. I was sick but i was afraid of injection; I don’t go to the hospital. I always bought drugs and one day, a lady visited me then she advised me that I should go to the hospital because if the drugs I bought myself would help like it has helped. And I went to the hospital and the doctor checked me then he told me the drugs that he has given me; I should take this in the morning; that in the afternoon and in the evening too; I would take it two times before I sleep. When I followed exactly what he taught me, within 3days then I have felt better. So we have gotten many benefits on that. I for instance, I could see that there are benefits in it.

1: The drugs that the doctor showed us how to take it; it helps the body. Maybe there could be a drug which is highly sedative and maybe the doctor has showed you to take one in the morning. Recently if not for God’s mercy like my mother could have died. I was chatting with her and if my brother hasn’t come like my mother died without me knowing. The drugs that the doctor had given her that she should take one in the morning, she said the drugs are small in size and she took two. When she took it, she was lying down and we were chatting; she had complained about her waist so she’s stretching on the bench. As she was lying down and she was quiet, I taught she has slept. So my brother was coming and he shouted banku seller and I said that she is asleep and he said that what kind of sleep is that then he removed the cover cloth. When he did, my mum eyes was opened and we took her to the hospital. When we went and he said that the drug she too, she has taken overdose then I said that, in the morning she said the drugs are small in size so she said she took two and he said that the drugs the we give you; we check your strength and the drug’s ability before we prescribe for you. So when we give you the drugs, you shouldn’t look at the size of the drug and say that this drug I small so I taking two. There could be a drug which is small but highly sedative so the drugs that they give to us to take it once and twice at a time. It helps. When you obey; it helps the body a lot.

2: I for instance sitting here, I got sick. I went for a funeral at my husband’s hometown so I got sick there; my stomach and they admitted me at the hospital. So when they treated me and I was getting better then the doctor told me that he would discharge me but I shouldn’t eat pepper. I shouldn’t eat heavy foods and the place too is by the sea shore; when they discharged me and I came, they had prepared fresh fufu filled with varieties of meat; when I saw it, very catchy and I disobeyed and ate the food; there was pepper in it and you know that for fresh meat, there’s pepper in it and when I finished eating, the stomach pain started again. So they took me there again and the doctor asked my husband that what happened? And he said that when she came, we had prepared fufu and she came to eat then he said that she has been killing her own self. They gave me an injection and induced me with some infusion then I vomited all the food. When I vomited it then I regained my strength. So from then I don’t take pepper at all. It’s just recently that I started taking pepper again. I started with one and it didn’t do anything to me and I continued with two and with three and now if I’m preparing food I use four so I take more of the tomatoes than pepper and the onions. So they said when I do that I shouldn’t let it overcook then I eat it. So when I prepare it, I follow what they showed me and I eat it. And as I did that and studied, my health has improved. So what the doctor would tell you to do, if you do it, it helps you yourself and improves your health.

Can it be applied to stroke disease?

2: It can be applied to it to the extent that, you are sick; you’ve been affected by stroke and you’ve been taking to the hospital. You’ve been treated and you have been given drugs; you’ve been told that this drug, take it in the morning at 7; when it’s 10am, take this one; when it’s 12pm take this; let’s take it as in a day, you would take 5times a day. Maybe some are small; maybe some are big and when you compare the big one and the small one that you are taking each once; there could be someone who is as disobedient as me sitting here and with the small ones take even about 3 so with you taking it like that then the disease is getting worse. So what the doctor has given you, you have to follow that. If he says once a day; 7am-8am or 7am-9am; 10am-11am; 3pm-4pm; when you are about to sleep then you take about two. So I believe that what the doctor would say, if you do that for yourself; you would feel better. I know of someone who has stroke; I was part of those who sent him to the hospital. The drugs I was there and they showed him how he should take it and I was the one who was giving the drugs to the person and for about 2-3months then the person got better. I think if the drugs are giving to the person and someone checks and see to it that the person takes it as prescribed, he/she would feel better but if there’s no one close the person to see to how he takes it and he takes it as he want to recover quickly; he thinks that when he/she takes them all at once, he/she would feel better; he doesn’t know that when he/she does that he/she could die. So I believe that if someone is close to the person and helps the person to take the drug, I believe that he/she would feel better. That is what I also know about it.

Source of information

2: I for instance, I am a traditional person. You see during the olden days, we used herbs to cure diseases. As I am sitting here, I have given birth to 11children. I haven’t been to hospital before. I have 11children and I haven’t been to hospital to give birth before. I give birth at home and I cut their umbilici myself; 40days and I take the child for weighing and vaccination but I haven’t encountered any problem in everything before. Also my children who have also started giving birth, the also give birth at home apart from two; even one of them it’s just recently then I wasn’t a home and they took her to the hospital to give birth. When she went to give birth she lost the child and the elder one too; she was staying at tech and I was also here. When she was in labor and they took her to a hospital at Pampaso, she gave birth to twins; a boy and a girl. She’s there right now. In our family, from my mother there’s no one who has given birth and been to the hospital before. We all give birth at home. I think that from the beginning what they adopted that they taught us to learn is what we have been stacked to. What they would teach you for you to also follow; as I am sitting here if someone is in labor; I can see to her to deliver the baby without going to the hospital as we do it at home or as any nurse does it, I could do it.

“So when a family member insist of going to the hospital, would you stop her?”

1: oh for that, I don’t have a problem. I could allow her to go. There could be someone who bleeds a lot when in labor. As I am sitting here, I have knowledge about it on what drugs to give to the person for the bleeding to stop. When the person wants to go the hospital, I have no problem. I’m supposed to even help the person to go. I wouldn’t even know the reason why she’s insisting on wanting to see a doctor so I have to allow her and take her.

5: My source of information and how I said it is that, me personally; I got sick, I used my discretion to go and buy drugs to take then the sickness was going and someone advised me to go to the hospital. The doctor told me how to take the drugs so it made me gained experience that when someone is sick and goes to drugs; I would advise him/her that he/she should go to the hospital. Go to the hospital and you would get better because me I had that experience myself and not on someone. It happened to myself so I have gained that experience and now if I’m sick, I wouldn’t go and buy drugs. I would go for the doctor to examine me and give me the drugs because I see that the drugs that he has given me, when I take them; I would get better. So I have gained that experience.

7: I think the question you asked on our source of information; I think today, electronic media has increased. I think most a times we get those information from the electronic media and learn them.

“Of which some are what?”

7: Such as on radio, on television, from doctors; for instance on Crystal TV like this, we have a woman called Madam Diana; she teaches a lot of health stuffs. So on how we get knowledge on health; I think the electronic media has also helped for us to get a lot of those knowledge.

2: What my brothers are saying is true. That’s what I already said. During the olden days, there were no hospitals. After then, one hospital is situated here; as I’m talking you stay at Bolgatanga and you cannot run to the place so that knowledge if a relative or your parents taught you, you can use it to help that person. As I am sitting here, I have delivered about 30people in labor and none of them has gotten any problem. So now that I’m here; it’s even my husband who has made me put a stop to that. So now if you come to me, I would tell you to go to the hospital because my husband has told me to stop though I can if I see that it’s very serious; I could pick a car and take you to the hospital. My husband said I should stop and that’s what has made me to stop. I have saved about 30people and their children’s lives. And as I am talking, they are all alive right now.

Belief/thought/opinion relating to precision medicine

2: For the drugs given to patients, I think there’s no problem with that. As we already talked about; you have taken the person the person to the hospital and the doctor has showed how the person should take his/her drugs so you that you are taking care of the person should; if you could help him/her and see to it that he/she takes those drugs, I think the person would feel better.

5: I don’t believe that there is a belief that forbids that thing. Even those who say they don’t like blood donation; their belief doesn’t permit them to take someone’s blood; even them, they follow doctor’s prescription. They do that thing so I don’t believe that there’s a belief which would say that you shouldn’t obey doctor’s prescription. I don’t believe there is a religion which forbids that.

“Can we get a household which only pay attention to traditional medicine?”

2: The reason why someone would say that is as a result of financial issues, there could be someone who is afraid of injection; even when he/she sees a doctor then he/she gets tensed. I live with a woman in my house right now as I am talking to you; she went to give birth at the hospital; she said what distastes her is a doctor to raise the syringe and hit at the tip of it so because of injection, when she sick; there’s no way she would go to the hospital for them to inject her or for any of her children to be injected. She has given birth for about six month now. As she gave birth and took the child for weighing about two times and stopped; just recently that she went; they vaccinated her thighs with about 3vaccines then she got tensed because the child doesn’t sleep for her to also get the chance to sleep. And I told her that, have you seen? As you have given birth, if you are afraid of injection; the child isn’t afraid of injection. As she is younger and she is being vaccinated; it could prevent any disease that could come her way but you don’t take her; small child, for about 3-4days now, the child has been crying often. When anybody takes her she doesn’t go; she has been a burden on you. You see, there is someone whose depend on the injection or financial issues which are making the person not being able to go to the hospital.

1: This issue that we are discussing regarding to people not going to hospitals when they are sick; in my family it is there. No one has prevented us not to go to the hospital. In my family, someone gets pregnant, she doesn’t unless the newer generation like our children; they went to the hospital.

“What do they do when they are ill?”

1: They don’t fall sick. For them, they don’t fall sick unless those of us in this generation; our children because of the foods that they eat which makes them fall sick of late. Even when they get sick, they go and buy drugs for themselves or when I got sick, I went to pluck herbs and I’ve prepared and taken them; I feel better. For me to go to the hospital; so in my family when you come; even with health insurance they don’t have; they don’t have hospital cards; you see so that thing it’s there. It is something that started from our fore fathers and they trained us with it. There’s someone in my family who has given birth to ten children and doesn’t even has hospital card. When she’s in labor, she delivers at home.

“Do they have any belief in doing that?”

1: They said that how they were brought up. When they are pregnant, they don’t feel any pain at any part of my body so why then should I go to the hospital for the doctor to examine me and I told them that it’s not good. But those of us here in Kumasi we go. My sister has given birth to five children them and with the five children; none of them was delivered in the hospital. Some of her children are even nurses. She doesn’t go to the hospital.

2: For me I’m a traditional person; I’m old, I just look beautiful. One of my children is at Sawaba as I’m talking to right now. Her husband that she is living with; the man is much matured. My child hasn’t gotten pregnant and been to the hospital before. It’s just recently that when I went there I told her to go and check; that was her fifth child, maybe it’s twins. Because she hasn’t been there before when she went and they asked how long she’s been pregnant then she said 5 instead of her to say 5months. When she returned back home, she didn’t go there again till she gave birth. Now she has given birth to 8children. As I was saying, me in particular I haven’t been to the hospital before and I haven’t fallen sick whilst I’m pregnant before.

“Doesn’t anything prevents you from going?”

2: No. I give birth safely and I cut their umbilicus myself and do everything then the next day; I go back to doing my things. Even that day you would see that I’m going to fetch water. It depends on what you would start with then it remains. You see this new generation that have come; our children whom when you advise them, they don’t heed to. As I’m talking the man has gone to impregnate her; she wouldn’t come home because she knows when she does you would punish her or she’s afraid so she would stay with the man or she would look for elsewhere to stay because of fried rice and other things. So for someone like that if God permits and someone brings her home to come and plead for her; she would be lying down quietly; even when you pluck herbs to treat her, it wouldn’t work unless you take her to go and see a doctor. She didn’t give you the permission to train her with it. I for instance that I’m sitting here, if I tell you that I fall sick; I don’t fall sick. I wake up at 3:30am at dawn; I don’t fall sick. Even with headache I don’t fall sick. It’s just recently that my knee and they said it is old age but it’s by God’s grace. Our forefathers they aged. In our home, we aged; my mum at the age of about 90years, then she goes to the farm and prepares her own foods before she died. She never got sick nor admitted.

4: The little I would say is that, with what you are saying; there’s a certain church, they don’t like hospital. I have a friend; the church is even at Kotei. They don’t go to the hospital but because of their belief when they pray for them then the disease goes.

“Do they tell you their reason for not going to the hospital?”

4: They said if God exist then they believe in God. They don’t believe in any medicine and I have studied them and noticed that it’s true. They don’t go to hospital.

“Is any of us here awareness of any policy or law guiding precision medicine?”

4: Personally, I don’t know.

## What do you understand by brain donation for research purpose?

Probe for level of awareness, perception, sources of information, perception of complexity of the procedure, benefits of brain donation, misconceptions, personal willingness to donate

Cultural, social and religious belief on donating brain for research purpose

What factors inhibit brain donation? Probe for cultural and religious reasons, peer values, parental influence, level of awareness, legal issues involved, knowledge of where it can be done, familiarity with medical and research settings

What factors promote brain donation? Probe for cultural and religious reasons, peer values, parental influence, level of awareness, legal issues involved, knowledge of where it can be done, familiarity with medical and research settings

## What do you understand by blood sample donation for genetic research?

4: For me I haven’t done some before so I have no knowledge about it.

3: For that we the person, we would know unless you the doctors would understand that; for us we wouldn’t know unless you.

What are your thoughts on blood sample donation for research?

6: For blood donation, it seems when you donate blood, then you have saved a life. As we already said, someone could fall sick and none of his/her family member would be available but they would need blood; it seems that’s what they used to help. Sometimes his/her family members could be around; maybe a relative’s blood group could not match with his/hers so they go and test for the one which could match then they give that to the person to save his/her life. So for blood donation for me, it’s good. It really helps.

Source of information

6: For that normally, we often hear that before you could donate; they test. For that people have been saying it for us to hear and we have kept it in our minds.

“What are some other uses of blood sample donated?”

2: For that we have already told you that we have no knowledge about that. For them to store it and use it for research; we have no knowledge about that.

Cultural, social and religious belief on donating blood for genetic research

2: You see, because of the rituals blood are being used for, if you send the person to the hospital and you say you’re going to test his/her blood, he/she thinks you are going to use it for rituals especially with pregnant woman; the umbilici, many people use it for rituals. So there are some people when they go to the hospital to give birth, then she would request to take the umbilici home; you see how the situation is. Many doctors are rich because of that thing.

“Why do you say so?”

2: I have experienced some before that’s why I’m saying that. I wouldn’t person the person’s name.

“When did that happened?”

2: It’s about 7-8years now. One friend went to give birth at that hospital; I went personally with a polythene bag for them to put the umbilici inside but they told me they had flashed it. Something that has been flashed would have been gone but he had used a white polythene bag to wrap it and placed it into another polythene so he had put it into their dustbin. So as he was putting it inside there was one woman sitting there who saw it but he told us that he had flashed it. So when he said that I didn’t say anything and I ignored. The doctor was rich but he wasn’t all that rich. After a year, you could see that the doctor had built houses. So from then that I never liked giving birth at the hospital though I have done that before; my friend was older than me although we are of the same age group. So since then and I said to myself that I would never give birth at the hospital. So a lot of that is as a result of that thing. When someone donates blood, he/she thinks the doctor is using it for research to discover the kind of disease which has affected me but he/she doesn’t know other purpose it would be used for. He has written your name on it but you don’t know the purpose it would be used for.

“Does anyone has anything else to say?”

3: I would want to plead with the woman that with what we are discussing, there are certain things she need not say. Though no one is here but it’s a recorder we are using so I would plead with you that there are certain things you have to exercise patience for us.

Please with regard to the blood donation, it’s a good thing for us, our country Ghana because maybe if I’m from here and I travelled to Accra and I get attacked by a disease, there’s none of my family member there and none of my friend are close to the place; even if a relative could come, maybe our blood group would not match unless maybe an outsider who donated his/her blood matched with mine. So when it’s used to save me, there’s no fault with that. It’s of a good purpose that the person donated for so such people the government has to even honor them that they are doing something good to help the country. So for me I see it as a good thing.

Awareness of any policy or law guiding blood sample donation for research and storage

## Tell us what you know about sharing of data, blood/blood fractions, brain images (CT scan/MRI) as well as brain tissue samples

7: I think with how we receive it; way back in 2004, I think when I went check my blood group, it was done secretly. They put it in an envelope and you send it then he opens it in front of you. It’s confidential; between you and the doctor that he share it with you about your blood group. It’s confidential between you and the doctor.

Opinion on sharing data, blood samples, brain images (CT scan/MRI) or brain tissue samples with another researcher locally and internationally

7: I think if he would share it with another person, he has to seek for my consent before he could share it with another person. If not, he is exposing my secret to someone else. Maybe with my blood group, I should have anything to do with this man or this woman and I are not supposed to even get intimate but when he goes to tell the person, the blood group that I’m in; she’s also the same type and we are not supposed to get intimate; I think he has betrayed me. So for that he has to seek for my consent before.

Commercial or non-commercial use of stored data, blood/ blood fractions, brain images and brain tissue.

7: I think for that, its commercialization and when we say we are not commercializing too; I think it wouldn’t help. I know one man who they collect him money that, [interrupted] I think with its commercialization, to some extent it’s good and to some extent it’s not good. I know one man, it was sold to him. Later then his child came and when his child came then their blood group was the same so the collected his own; at that time, they had already collected the money. But when the man got better, he demanded for refund that if his child has donated; they should give him back the money at Komfo Anokye Teaching Hospital; I think they didn’t agree to refund the money. I think for its commercialization, it’s good.

“Why do you say so?”

7: Because if it’s also done for free, I think in Ghana here; in our society, anything which is free; we abuse it but when a policy or money is being collected; we wouldn’t abuse that thing.

6: I think with the commercialization of the blood; it seems now they have changed the name; they said it’s processing or something I don’t know because one brother of mine; he often donates. At first when he goes, he has a certain paper; when he needs the blood for someone then he sends it there but now when he goes, the mention a certain name; processing fee or something like that then they collect. And for that of course if the person is sick or maybe if you come to help, it seems it is part of the bill but for him/her to bring money before it being given; for that It seems it’s a little frustrating.

“Do they tell you the reason for collecting the money?”

7: Most a times they tell you that they are selling it to you and if you can get someone to donate for replacement then they would agree but if you have no one then they sell it to you. That’s how they explain it.

## Share with us your thoughts about return of individual research results and incidental findings

7: I think most a times when you go to some of the hospitals they ask for your email address and other things. So if the person has an email address and I think only he/she has the password to that email address. Only he/she knows it in his/her mind. I think if it could be pass through the email address to the person, I think it would help because I think he/she has the password to the email address.

6: I think with regards to writings of doctors, it’s in signs and other things. As I’m talking, I’ve gone to test; what the doctor would write if it’s not him who would understand and explain it to me that this is what is says. If you show it to me there’s no way I would know. And also sorry to say, many of us are illiterates; you write in English not Twi. So I think that how you’ve been doing it, it should continue that way. If you keep it in an envelope then you hand it over to a doctor to explain it to you; I think that would help.

What are the ways that you think one can receive the results of genetic research?

What are your thoughts on returning individual research results and incidental findings?

7: With regards to that, I think most a times it often happens. The person who is sick, most a times aren’t able to talk or the situation the person is involved; the doctor can’t have that conversation with him/her. So most a times, on his/her form; they have a place for the next of kin on it. I think that person they give the information to is a very close relative who they can give that information to and for him/her too, I don’t believe he/she could bring such information out. If it’s not because that person cannot talk at that moment or the way he/she is in that situation wouldn’t be possible to have that conversation with him/her; the next of kin should be someone who is a very close relative to person then we could give that information to so that it wouldn’t be able to come public.

Opinion on desire for feedback of research results and incidental findings

7: I think it’s very important to ask or request for feedback because with you per say; it would help you. Because I remember I went to a certain hospital; when I went I asked that what was really the problem with me; they told me that if I knew what was wrong with me like I would stay there for them to treat me. I ran away from the hospital; I didn’t stay there. I ran away from there and went to a different hospital at Pampaso and went to seek for treatment there. I think that when you ask, it would help you yourself and it’s important for us to ask.

What are the challenges of returning individual results?

7: I think with that most at times the patient who is sick is eager and the doctor also consider the situation which he/she is in; maybe it wouldn’t help the condition which the he/she finds himself/herself at that moment that is why maybe sometimes they also delay up till some time before they could return the results to that person. If not maybe it could escalate a different disease.

6: The words of my brother reminds me of a statement someone told me. He said HIV doesn’t kill but the moment a doctor would tell you that you have HIV; it’s the thoughts of it which would kill you so there are some information when you hear of it, it’s irritating. [Loud laugh]

3: Somewhere around last month June; then my father was sick a little. He got sick for about a month before he passed on. Without know, the time he went to take a scan, when the doctors were chatting; he overheard it. They were saying all his intestines and other tissues were damaged and they didn’t tell us his relatives. They transferred him and a referral letter to Komfo Anokye Teaching Hospital but the conversation they had; he overheard it and when we return home and was about to take him to Komfo Anokye Teaching Hospital then he told us that we should wait a little and we told him that the doctor has transferred him to Komfo Anokye Teaching and you are saying we should wait a little. And he still insisted we wait a little so we didn’t understand then he later made us understood that this is what he has heard so when we take him to the Komfo Anokye Teaching Hospital, we are only going to waste money. So he waiting on the days God would call him that’s why he’s telling us that.

Ways they would prefer to get feedback: phones, e mail, letters, feedback by a healthcare worker? a researcher? or a clinician?

1: the doctors are supposed to maybe the relative who is close to you, then they tell him/her about the information but for the sick person; he/she is in pain then you go on to tell him/her that maybe when you did the research/test; this is what your findings are; it’s difficult. Then my daughter was pregnant and she went to the hospital, that was where she had gone for antenatal; if you had seen that she was going to be operated, maybe they could have told her that when she goes; she should bring her mother. When they checked her result; the nurse promptly told her that they would give her a referral letter to go to Komfo Anokye Teaching Hospital for them to operate her. The child left the hospital with tears; she cried a lot then we asked her why. So when we took her to Komfo Anokye Teaching Hospital, her pressure had increased, you see; if it had not been my father, I would have gone to the hospital to insult them that they are not supposed to do things like that. From the beginning the person came there so when you saw that the point she had reached, she wouldn’t be able to deliver herself and she’s supposed to be operated; maybe take this letter and take it to Komfo Anokye Teaching Hospital or maybe call us your mother but when you call her then you promptly tell her that she wouldn’t be able to deliver herself so she has to be operated then that brings some kind of fear in her. So the doctors when something of that sought happens, you should call us the relatives and tell us that maybe this is what has happened and if you do it this way, it would help or when you continue to spend money, it wouldn’t help so when there is an expensive drug; you shouldn’t buy it. You should buy inexpensive ones which would help a little. But there are some they wouldn’t say it and in front of the sick person, they would just say that for this disease you would die then it brings problem and thoughts to the person. That would even cause you to die. So the doctors we plead with them.

7: With that I think it’s either they would inform a relative or the patient in particular should be counselled before they could inform him/her.

What are the ethical, legal and social issues relating to returning individual research results and incidental findings generated by genetic research?

## Explain your understanding of Biorights

How much control should/can individuals have regarding how their biological specimens will be used in research?

What rights do/should individuals who provide their specimens for research have over their specimens, how they are used in research, and any profits from research discoveries made possible from them?

How should autonomy rights be best balanced with societal benefits that derive from the use of human specimens in research?

## What is your opinion about governance and regulation of biobanking?

Need for ethical committee approval on future use of stored data, blood or brain tissue resource for research

6: I agree with that because if there’s something and there is nothing governing it, it becomes needless. So if a committee can be formed to see to it, it would help a lot. If it exists and there’s no law governing it or nobody is monitoring, it seems it wouldn’t help.

Need to set up a regulatory board

7: I think that one too is also good. If there is a regulatory board or if the laws are set, I think that would help the act to be successful.

“What are the benefits of blood donation and for research purposes?”

4: For benefit, we have achieved a lot. For instance, with what we are discussing, it has made seen that even if we are to take any drug, we have to consult a doctor before we take it.

7: I think with regards to benefits, it’s a lot which have helped us and also help the next generation. It has really helped us a lot because if blood donations and all those things were not there; I think like many lives would be lost. With me personally, it has help a very close relative of mine.

## Explain possible intervention for implementation of biobanking

What suggestions do you have that can help raise awareness and improve attitude towards blood sample or brain donation for research and encourage people to adopt the practice?

7: I think all those activities are good. After everything what would really is mass education. We should do mass education for instance during elections; sometimes the language is being said in Twi, then they say it in Hausa, then they say it in Ga and they say it in many languages so when we do all those educations and when the thing is being implemented; everyone has knowledge and understanding about it so the mass education is very important.

6: It seems today; social media helps a lot. So I would prefer that it should be advertise through it. They should explain it so that people would understand because with what we are even discussing now, there are lot of them I haven’t heard of them so when you do the education, I think it would help. So you should advertise it through social media.

5: My opinion is that; they have to recruit more people like you so that you would be many so that the work wouldn’t be difficult for you. They should recruit more people, you see and you should go down for the information to sink into people’s mind, when we do it like that it would help.

4: What I want to say is that, they should employ more people and they should roam about so that the education would increase. When that happens, it would help.

3: My little opinion is that, we Ghanaians; we are very disobedient. Something which is very little then we ignore until it becomes a very big problem. If the people in the country would help and agree for the education to go on; we should let the journalists, TV personalities and Radio personalities advertise about it and they should add you too so that someone without TV or radio but when you roam, it would help for everything to be successful for us.

2: I also my opinion is that, with you that you have the knowledge about it and you have called us here and educating us; you can do it like that. I for instance, when they called me, I thought I was just going to be asked a question and I would return. You heard me saying I have somewhere to go; it’s the work of many people, when you do it alone then insults set in. as you have seated me down and educate me; you can do the same to someone else but you have to take more people; you are here and then some are there and some are at other places. So when the people meet and they are discussing and one raises it up then the other too would say the same. I think when you do it like that, it would help us.

1: I plead to ask that, right now the things that you’re teaching us; are we supposed to go and donate blood at the hospital or what? My opinion is that; it should be advertised on the television. And it should be on radio so that it would be in everywhere in Ghana for people to know what is happening so that it would help us.

## Any other major concern or recommendation on use of blood or brain tissue for research in Ghana?

6: Mine is a question. I heard that regular exercises; as a human when you do regular exercise, it prevents you from getting stroke because I know someone who leads people to do regular exercise but at the end he got affected by stroke. What happened?

## Share with us your opinion and thoughts about blood sample donation for stroke genetic research.

Willingness to be involved in such research

What do you see as the barrier(s) that could hinder your donation of blood sample for stroke genetic research: family member, cultural and religious reasons, peer values, parental influence, level of awareness, legal issues involved, knowledge of where it can be done, familiarity with medical and research settings?

What do you perceive as benefit(s) of giving blood sample for stroke genetic research that could promote your willingness to donate: cultural and religious reasons, peer values, parental influence, level of awareness, legal issues involved, knowledge of where it can be done, familiarity with medical and research settings?

What can you say about your family member or other members of the community willingness to give blood sample for stroke genetic research

What could be done to make you and more people give blood sample for research: mass media, husband consent, family consent, donors group, peers?

## Tell us what you know about informed consent?

What do you know about the consent process for genetic research?

Types of inform consent preferred (broad, restricted, tiered and dynamic). ***Facilitator to please explain each type to the participants

Reason(s) for their choice

Person to be involved before participation

Data use in the incident of death and why

Support for generic consent for community

## What is your opinion on storage of blood sample and blood fractions for genetic research?

Opinion on storage of blood sample for future use in genetic research
