# Supplementary material for: Research participants’ perception of ethical issues in stroke genomics and neurobiobanking research in Africa
Source: PLoS One. 2025 May 6;20(5):e0292906. doi: 10.1371/journal.pone.0292906 (PMC12054916; doi:10.1371/journal.pone.0292906)
Supplement: S3 File — (ZIP) [file pone.0292906.s003.zip › Files for PLOS ONE - updated March 2025/Zaria_ Caregivers_FGD.docx]

CARE GIVERS

I welcome you.

Give answers to those you have answers to those if you don’t, keep it there is no right or wrong answer like I said earlier, all we want is your idea about this research. We start with number 1.

1. **TELL US WHAT YOU KNOW ABOUT GENETIC RESEARCH.**

Anyone you don’t know we can give you definition. What do you know, have you heard of genetic research? If you don’t know, just say you don’t know that’s all we want.

Have you heard about genetic research, where did you hear about it, what do you know about it, do you know anything about it?

**ANSWER:** Yes, genetic research is a research that is done on gene generally take for example, we have research that is genetic for genotype – genotypic and for also BP, when we say for BP, it’s all about gene and inheritance if am not mistaken. So take for example- your father is having high blood pressure, probably, it’s going to spread to the gene to the children also to be careful you have to be monitoring their BP. Most especially with an obese, when one of the children is obese even though it’s most common with obese patients and when we say for genotyping there is AA, AS, SS, SC and so on, there are six steps. Genetically if a father is AA and a mother is AS so when the cross matching is done they are going to get AS three and AS one, if they are AS and AS, definitely they are going to have AA, AS again and SS and soon and so forth, that is the little I can say about genetic.

**So is there any benefit or you have of it somewhere, or any other idea.**

Not really somewhere is just going through………… There is a lot of benefits because with the research we will be able to know how to modify our life style in general I mean.

It’s good for the essence of marrying each other, by the time you don’t check where you are SS or maybe the mother is of age, by the time you did not check in the hospital, they won’t match each other then they will start delivering SS.

Thank you let’s go to the second question.

**CAN YOU EXPLAIN WHAT YOU UNDERSTAND BY BIO-BANKING?**

**NUMBER 6**

Actually let me explain, Bio means life, it deals with cell because this research just as I said before, the main research is to,--- we want to start storing some tissues so that they can be used for some patients, you understand, you find people, you there is different tissues that we used to store: sperm, a lot of tissues even eyes, some part of eyes even blood so all tissue of the body.

Have you heard about it? What do you feel about it, Socially, Religious, culturally do think it is the right thing, or you have anything against it?

NUMBER 4: Actually I’ve not heard anything about it before. But when we say blood in other sense deals with life bio banking take for instance blood banking is very very important in the word in short because biobanking is very necessary for our population to move on for example in the blood bank, if there are no people that are donating blood so it means when the PCV is short, there is going to be a problem, when a patient is anemic, there is going to be a problem, a problem in the sense that if there is no donation, it not going to be fair the patient is going to move to coma and anything is going to happen.

Any other idea?....

**Number 2:**

Bio-banking in all aspect whether organ or blood is very important because for we here in Africa due to high rate of road traffic accidents one might damage one of his organs and we don’t have this in our country or in our continent and if we have this now it will help to safe a lot of lives. Okay even kidney and so on.

Any other idea on bio banking whether it is important, whether you think it’s right, whether you think it’s wrong?....

**Number 6:**

It about the issue of the blood, we have some or part of the Christians who don’t believe you fix any other blood for them in their system.

Okay like you do you think it’s right to store organs?

No, it’s not right… it’s not right to store the organ.

Any other idea?.....

Let’s go to question number 3: **Can you explain what you understand by precision medicine?**

I will help you define precision medicine then you tell us your understanding whether you’ve seen it before or you think it’s good to apply it, and whatever you have to say.

Precision medicine is a model of medicine that is tailored towards the need and peculiarity of everybody, for me to treat you now, I will base it on your need because Justas you know that we have different genetic makeup so my treatment for you will be based on your particular need, So have you heard about it before and what can you say about it, do you think it’s the right thing to do?

**Number 4:**

It’s not right as number 6 said earlier, some part don’t want blood donation in which as far as I am concerned is not right. Because if the whole Zaria now believe in that what do you think will happen to the population in Zaria? It’s going to be a problem. O-Negative everybody knows how it goes that it is very rare so if they now said if the whole Zaria one person is having it and he has already donate before in less than three months, is it possible for the patient again to get blood, it’s no right. In this aspect again, based on what the patient need that is what you are going to give, there is going to be a problem because the problem is some people don’t have money, most people the issue of don’t have so if you now need money and you say you are going to write some drugs for me and I say I don’t want that meanwhile the doctor knows that the one I want the pharmacist know that there are complains, there may be fake drugs outside, you understand, when there is fake outside, there is going to be a problem, and you say that is what you want. They are still trying to convince you that it is not proper, that you are not supposed to take it and the person say he is going to take it, to me that is-----.

What we are saying about precision medicine, in this medicine, we look at the person’s peculiarity just as you explained in the first place you know we were talking about gene. In developed countries they study about individual person’s gene so if you want to treat him, they will treat him based on his gene peculiarity and condition do you think it’s good, do you think it’s good, have you heard about it before?

Number 2:

I think it is a welcome development like now some do react to some drugs when they are going to be treated based on their gene, I don’t think there will be any reaction and it will really fasten in healing.

Number 4

Is there room for question?

Well you can ask, no problem,

based on gene does it fail?

What we are trying to say is that individual you have your composition just as they give you example of chloroquine, this person may react, this person may not react. Just like sickle cell you said, based on genes, you can say okay, this person can get it, this person cannot get it so that precision medicine is based on your peculiarity, your need as an individual, so have you heard about that type of medicine and do you think it is good?

With the way you explained it is okay

**QUESTION 4**

What do you understand by brain donation for research purpose? Any answer, do you understand brain donation for research purpose

Number 2: Based on my understanding, when one is about to die, one may decide to sign that he is going to donate his brain or any organ for research purpose and this research as far as I am concerned will help in improving the quality of health care..

Any other idea? What do you feel culturally, socially religious wise how do you feel, do you think it is right do you think it is wrong? Any other idea, anything?

Number 2: culturally we have a lot of ethnic groups so some might agree some might not but still if that person…. Let me move to religious, religiously if one signs and says okay part of this is what I want to be done, definitely that has to be carried out. So if he is donating his brain that has to be carried out before he is even buried.

Number 6: it is not only in the Christian religion that you donate part of the body, ----it is wrong

**Question 5**: What do you understand by blood sample donation for research purpose, donating your blood for genetic research. What do you understand by it, do you think it is the right thing, do you think it is wrong? You know sometimes you donate people’s blood to conduct research on it, to look at their gene compositions. Do you think it is right?

Number 4: with the patient consent? Yes of course anytime they are taking your blood they need your consent. When you are talking of research you must sign, you must get consent. But what do you feel about it

It is very right, the reason is this: it will help the patient to know what he or she is up to pertaining the genetic level it is not only sickle cell that needs genetic check, some people might be having high sugar, some might be carrying it in which they won’t know the only thing is they will be having the sign and symptoms in which if they did not come to the hospital, they will no know much about it a lot of people they will not know, but when they sign, that is very very important,

Any other idea…?

NUMBER 3: Before I have never test of genotype because I go to the hospital that’s where they tell me my group, at home, I don’t know my group.

Number 6: It is good to donate or to check your blood/donate I have one brother that he have hepatitis, its when he went to donate blood in 400Levels ABU, they notice he has this genotype, assuming he doesn’t do that, he will carry it, till that sickness comes, it is good.

Any other idea again?

NUMBER 4: To add to that, hepatitis, the way it moves now, its not only through sex, also through body fluid once there is cut so the person can contact it so with that it is very very important.

**Question 6: Share with us your opinion about blood donation for stroke genetic research.**

Number 2: With the high rate of stroke and high blood pressure which leads to the stroke, infact its supposed to even be mandatory for one to come and know all this things and donate for the research so that it can be curtailed.

NUMBER 4: it is very important most especially those coming for clinic. If it is possible, they should tell them to come to the hospital if it is free because you know money now, does a lot of things. If it is free so that the children will come and check and see what is going to happen in the nearest future.

**Question 7: Tell us what you understand by informed consent?**

Let me give you our definition, this is the voluntary agreement in a research. It is the process in which the subject has a clear and comprehensive understanding of research and its risk; in other word, informed consent can be referred to voluntary and guided expression of free will to participate or his or her legal representative to allow collection or storage or transfer of participant’s biological materials for research data. Just like we told you, at the beginning we have to get your consent that you are ready to participate, those who are not ready, can go, so informed consent-have you heard about it before, there are different types of informed consent, but we want to know if you have heard about it and what is your opinion about it.

**NUMBER 6:** I heard about it before like the time I had my last born pregnancy, they called my husband to come and donate blood he refuse he said that he will not be able to donate.

**NUMBER 4:** I heard about it because most people normally come to the lab to come and donate, for a lot of research. Most normally drop their consent form with me which I do help them to clerck the patient and take their information that is the place I heard about it.

There are different types of informed consent where you know and you agree there is also generic informed consent- this is the consent of those that does not seek specific information, it focuses to seek participant’s consent and blessing on broad and overall focus/scope of the research for instance for a bio banking, project where the objectives to collect information and human tissue sample for assess and use for multiple researchers.

Broad consent is a form of informed consent approval or agreement given to participants to allow collection, storage and transfer of his or her biological material and are associated data collected for future use in research, what the generic does for example if they are collecting your blood they are telling you what they want to use the research for. It is the broad one, they can even use it for what they did not tell you about.

There are other restricted this is the form of consent where a doctor or research participant restrict the use of sample or data collected from it to immediate research only and agree for use of sample for future research.

Then tied consent, it’s a consent model in which participants are given set of opinion allowing them to agree or select how they want to participate in the research. So these are the different types of consent. So what is your opinion about informed consent, and what can you say about it?

**Question 8: What is your opinion on storage of blood samples and blood fractions for genetic research?**

Number 2: I feel its okay, the sample collected is going to be used so far the research is going to be beneficial in limiting illness.

Number 4: it is very important it will help the community it will help them to help others through the research

Number 5: it is very important to donate blood for research purpose.

Number 6: it is very nice because you are helping others.

**Question 9: Tell us what you know about sharing data, brain images as well brain tissue sample?**

For example as you are in the hospital your patients carry out MRI or CT scan at a rate subsidized by the research what do you think if the results are shared with other researchers. Do you think it is right to share samples with colleagues in the US or Ghana to study the blood and see what is wrong….?

Number 2: it is said that two heads are better than one if its been shared maybe the first researcher may not see what the second researcher may see, so far if it been shared the person is going to send the result of the data also, I think it is okay.

Number 4: it is okay because it is with the help of this data that we move on without sharing of data, on the result that is been gotten there is not going to be a way forward.

**Question 10: Share with us your thoughts about return of individual results for incidental finding?**

For example if we say we are conducting a research on diabetes do you want us to give you the result and if in the process we get another finding, should you be told? And how should it be delivered is it face to face, is it through phone, is it through email. How are you going to get the result?

**Number 4:** it is good to get the result face to face by giving you the result you will want to know what is in it, after knowing what is inside, the doctor will tell you the way forward and the issue will die down, I also expect to be told of any accidental finding.

**Number 2:** it is very important to get back result, if it is unwanted feedback then the person needs to be counseled before giving him and if it is something good then, and it should be face to face that person should be told and to educate him more on how to maintain that state and if it is the unwanted one, it is good to tell him face to face and to tell him how to go about solving it.

**Question 11:**  **Explain your understanding of bio rights, any understanding, has any one heard of it?**

**Number 4:** Bio rights is still based on your consent…

Let me explain, Do you still think you should still have right over tissues or organs you have donated? So that you can say do this or don’t do that or if you make money from it people should be given?

Number 6: No, to say that is wrong people should not have right over their donations since they have already given it.

Number 4: Bio rights is a two way thing it is right and in another way wrong because when you get your result face to face, then they will tell you the forward if you need a counselling or not. In the other way round it is not, if you see that you have already given, you must not look back again and know what they want to do with it since it is with your consent and you sign.

Number 2: I think one should have the right but not all the right one should have the right to whatever he donated those who are using it are trying to use it against what he or she has signed or lets say after donating I was told it was free and they try to sell it to recipients, I think I should have the right to revoke it.

**QUESTION 12: What is your opinion about governance or regulations on bio banking. Do you think government should regulate it, are you aware of any regulation?**

Number 2: I think there should be regulation because the research might be used to exploit.

Number 4: there should be regulation but it should be standard so they will not abuse it.

**QUESTION 13: Explain possible intervention for implementation of bio banking, tell us your idea on how we can make bio banking work.**

Number 2: The government should create awareness on the importance of saving lives apart from that the religious leaders, traditional leaders should create awareness on the need to save lives.

**Number 6:** bio banking is good it is very good and important. Government can help us, community can help, mosque, church, market can help. All these people can help.

**Number 4:** Even the hospital too can help, if people come early in the morming there should be counselling, what we are supposed to do and what we are not supposed to do. Health talk.

**QUESTION 14: Any other issue or concerns on the use of brain tissue for research? Any recommendation, concern or fear?**

Number 4: they should standardize it. We have protocol, they should follow the protocol, there should not be short cuts.

Number 2: There should be adequate equipment to carry out the research.

**Number 5: I don’t have any recommendation.**

Thank you very much for being part of this research, we have come to the end we are going to have a very small survey which you are just going to answer and at the end we are going to give you three-thousand-naira incentive which you are going to sign for.
