# Supplementary material for: Research participants’ perception of ethical issues in stroke genomics and neurobiobanking research in Africa
Source: PLoS One. 2025 May 6;20(5):e0292906. doi: 10.1371/journal.pone.0292906 (PMC12054916; doi:10.1371/journal.pone.0292906)
Supplement: S3 File — (ZIP) [file pone.0292906.s003.zip › Files for PLOS ONE - updated March 2025/Zaria_ Community Advisory Board_ FGD.docx]

**Interviewer:** Anyone you don’t know we can give you definition. What do you know, have you heard of genetic research? If you don’t know, just say you don’t know that’s all we want.

Have you heard about genetic research, where did you hear about it, what do you know about it, do you know anything about it?

Participant 2: Genetic research to my knowledge is the research for a study that is done towards genes, human genes and how it affects individuals particularly in the compositions of the person likely sicknesses and then the cause of such sicknesses which will now aid in handling solutions and cure to the sicknesses.

Participant 5: Genetic research is a broad aspect of research on genes. Genes generally are part of a human being or a cell which actually carries the information or the blue print or the organism of a particular cell. The research can be for knowing the genetic nature of the individual, the specific genetic abnormalities in an individual that may expose the person to disease. They also include the genetic modifications that can be done to alter the cause of disease, or to cure some diseases or to even alter the appearance of the individual or the organism, so it has a lot of aspects, there are some aspect that have ethical problems particularly when a research involves the modification of genes which the product of such modification may not be acceptable to any of the society and depending on the purpose on which that research is made. A quick example is like producing organs, changing or reproducing an individual, stem cell research and so on. Some of the things that are still very controversial at the moment and have a lot of ethical implications. So it depends on the aspect why is done, there are some that are done regularly and is acceptable ethically, there are those that are not ethically acceptable in most parts of the world up till now. Thank you.

Participant 6: Genetic research I think I came across it as far back as 19years ago, I think I wrote articles on the student journal on the Human genome project which was then a project that was carried out by the united states whereby the whole genes of the human body were mapped out individually and what is responsible for what, so right from that time the fears were what would that information be used for, as rightly said by individual number 5, You can use it to define the risk of individuals developing certain types of diseases, particularly cancers and other diseases that are genetically influenced for example Hypertension which can give birth to this stroke and Diabetes mellitus. So many people were happy to know that once you define these genes and investigate further on them, how they dispose to these diseases then the knowledge can be used to prevent individuals from developing the diseases and not long from then the issues of cloning issue came whereby tissues were genetically developed, people were worried that that information could be used to a negative aspect and I believe a movie has been done on this cloning whereby certain group of people were cloned and caged in one place away from the general populace, so individuals within committees who had some illness that required part of their body to be replaced such as the kidney, those people that were genetically cloned from them sacrificed then their organs are used to cure other people so I think that is the aspect that really concerns, that brings about the issues of ethical concerns seriously. Thank you.

2. Can you explain what you understand by bio banking?

Participant 6: From what the word means, Bio meaning something to do with life and banking is a form of storing something, so I think what the word means is storing some parts of human tissue especially when we are talking about human tissues now. This storage is mainly for the purpose of treatment for research because I think the only we do is obtaining blood, storing it in the blood bank to be transfused into the individual or another person who really requires blood or part components of the blood. Other things that have been stored include skin of individuals, their skin are harvested and are stored for individuals who has sustained skin loss such as extensive burns, can be used to replace their own skin in form of treatments and other tissues are also obtained for organ donation for example, individuals that develop brain death which are believed to be vegetative that are not going to survive any *** that cannot recover from such insult to their brain, their relations or they themselves could have signed consent that when they see them reach that kind of state part of their body can be used to treat other individuals, so such individuals won’t know that body parts maybe hacked, like kidney, liver can be also stored and given to other individuals. The aspect I don’t know much about is if some parts of an individual who is living can be donated apart from blood because for research purposes especially as unique to this genetic engineering research, when it comes to taking part of the tissue form the living individuals apart from blood which is widely accepted, generally I think it will have a lot of social as well as legal implications. Thank you

Participant 2: The term bio banking is a term that has not escaped controversies. A lot of person have keepings as to why and how and why we might go into storage of life that has affected so much the acceptability of that very concept. I know of some people that are ready to die when it comes to the point that that is the only solution to their problem may be based on faith, religion, culture and things like that and the medical field comes into various challenge when it gets into that, they are at a cross road sort of on how to save life and how to respect the personal liberties of a person. And another aspect of it is when you look back that one that has already decided on his own that giving his consent, so this issue of bio banking which, is a bit difficult because it’s something you cannot compel anybody into and you cannot dissuade anybody of it, it is not for anybody that an individual must willingly condone training to decide on when he gets to that point. Thanks you

Participant 5: In addition to what they have said, those tissues from a living individual or a dead individual can be bad, living tissues or dead tissues can also be bad, but the key thing here is the purpose for which such tissue will be obtained and banned must be clearly explained to whoever is submitting it. How they will be protected, what they will be used for such that the individual is very clear when signing the consent for the tissue to be obtained from them. Now the major fear of people, even if I sign the consent, or accept whether a researcher or whatever to obtain tissue from either a living or a dead person, once you give it out the control of what it should be used for leaves your hand and one cannot guarantee the safety of such no matter how the researcher reassures or knows what it will be used for. So that’s the major thing, what will it be used for, how will it be protected, who is responsible for its protection, what if such tissues get into the wrong hands. These are some aspects, so the person donating the tissue is very clear about the purpose *** to a large extent the researcher or the collector must strictly abide by the purpose for which that tissue was obtained and nothing outside what the person consented for. Thank you.

Participant 2: Let me also add that there will be need for **** on how to address these fears raised by the latter, because its affecting the entire system.

3. Tell us what you understand by precision medicine. (Probes more)

Participant 5: when this question is asked, are you going to define precision medicine to your respondents?

Moderator: yes, of course if you want we can just enlighten you.

Participant 5: it will be nice to enlighten the respondents in shortening the process of information on what precision medicine is, it’s easier for them to answer it. The explanation should quickly follow the question so that it aids understanding. So from what you said regarding precision medicine, it’s a treatment tailored towards an individual, every individual is unique in his makeup and particularly the uniqueness, it stems from our genetic makeup and so the way we interact with the environment depends on our genetic makeup and the effects on the environment on us also depends on the genetic makeup. We can be born of the same parents, the same similar genes but one develops hypertension and the other doesn’t, that is because of peculiarity of genetic makeup. We are raised in the same environment and yet one person has a particular disease and the other doesn’t. now even if both individuals have the same disease they may not respond appropriately to the same treatment or*** So the concept is if we can unravel the genetic makeup of each individual then it is easier to tailor medications to them, there will be effective rather than trying same treatments on both of them and one is irresponsive and you start asking the question Why. So these are the ways they are thinking to see if the treatment can be targeted at an individual based on their peculiar makeup. Thank you.

Participant 6: To add to what number 5 said, I think precision medicine goes far beyond that level, up to the extent of cloning of body parts for the knowledge of genetic makeup, to know what genes responsible for what part of the body. Like some individuals that have lost some parts of the body like an ear, the nose or even the hand, without the knowledge of their genetic makeup, then body parts can be developed which will be exactly like that they had before or based on the knowledge on another organ which they have used such that the resultant organ or tissue that is developed will match same that that individual requires and that will be planted on that individual. Again that brings up the issue of are we trying to be God when you look at the religious aspect because its God that creates, so if one loses a body part and we create a body part same as that one as if we are trying to play God and because of that a lot of people will not want to agree to such form of treatment, but of course, that will be the peak of advancement in medicine as far as I am concerned. Thank you.

4.

Participant 6: The brain is considered as let me say as one of the most delicate organ we have perhaps including the heart, but to say that you will take part of somebody’s brain is not something that the public will really understand being that each part of the brain is believed to control general body parts, just as we with stroke, depending on what part of the body is affected that why somebody cannot develop the stroke in the part of the body he cannot use. So I think for people to agree for part of their brain to be taken for research will be something that is very serious, that will have to be given serious consideration except I think medicine, doing such form of brain donation, sampling is being done especially where people have some kind of diseases for example cancer, for you to know the type of cancer sometimes you have to take a part of it, and for treatment that part has to be taken and analyzed, what kind of cancer it is and what kind of treatment it needs. So I think that kind of treatment is clear and acceptable buy to say you will take of somebody’s brain will be really difficult to comprehend or to allow.

Participant 5: yes. Thank you very much, I think one of the most popular we’ve heard is Albert Einstein, I think his brain is still preserved up to today for research purposes. He is one of the geniuses of his time and up till today of course we are gaining from some of his fantastic ventures and research to be precise and so a lot of people have been studying that brain to know what made him a genius. In the western it is easier for consents to be given for body parts to be used either as organ donated for the use of who are still alive or for the purpose of research. Quite a lot of them would have given consent before they die, in case they die any part of their body can be used either for the purpose of research or to restore other people’s function. We don’t have that culture in our environment or **** that is one of the reasons why autopsy rates are so low in Nigeria and particularly in northern Nigeria. Our religious and cultural practices make it so difficult for consent to be given for that even just for autopsy were tissue is not removed maybe just a little part but mostly the body remains intact even after the autopsy but consent is difficult to obtain to a large extent but we have to appreciate that. Islamic religion, we bury our dead soon after death as soon as possible, we don’t even any room for thought or for any individual to consider such and of course even they are*** to giving any parts of the body of the dead for any of the purposes, so one has to be very careful the way such questions are put to whoever will give consent for that, we may have to modify it not necessarily lying about it but to make it appealing, like number 4 said, for you to get a consent the individual must know the benefit, I want to do this everyone will benefit from it, those who have stroke those who have not had stroke, they all will benefit from it, you can educate people better about the need of that research, it might be easier to obtain consent than expecting people to just**** with more education. Thank you very much.

Participant 6: A lot of people are afraid especially with the stigma of brain illnesses particularly psychological illnesses. Typically, locally people see it as those that have become mad, I think people will be scared that if you touch their brain, they know that madness arises from the brain. The public may be worried that if you go to take sample from their brain, you may touch a part that may just change my mental status and I may become mad, so I think you really have to know how to really design the question to clear this aspect so that they understand and agree to consent to sampling their brain

Participant 3: ******* one area I see some challenges is that, donating a brain tissue will require the person undergoing an invasive procedure, I see respondents not easily giving consent, why they may not have much reluctance to still subjecting their selves or their brains to other studies like ****** studies, asking them to go under anesthesia, being operated on the brain culturally many people may be withdrawn from it, so they will really have to be persuaded and ***

Participant 2: the area I want to come in is on the area of education, a lot of persons are not all that aware of the benefits that will be derived by such donations, and that is where the medical practice and government have so much work to do, from educating persons on the need for such donations not only for the brain but for other tissues being donated for further research and developments. Thank you

Participant 4: just like the last speaker has said, there will be need for proper education and encouraging people to go to the hospital as soon as they are sick because it will be difficult for one to take a corpse that died in the home, to take it to the hospital for such brain donations. So they need to educate them, giving them the importance of taking people to the hospital as soon as possible would be very necessary, so that the issue of donation or recovery can easily be ****

5.

Participant 6: Blood of course is normally looked at as life, so when someone sustains an injury and loses a lot of blood, most people know that that person is likely to die. It has been the most sampled tissue for majority of the test that are done in the hospital, it says a lot about an individual and I think the use of blood for genetic research can easily be accepted by the populace, it will easily be obtained and only a little quantity may be required that may not cause any detrimental effect to the individual for the sample that will be taken for the research but I think what will be done with it is exactly what will we have said in response to the last question. When they give their sample for genetic research, they have no control over it and a lot has been done on blood to determine most of the common diseases that we see genetically for example Sickle cell disease, it has been defined genetically very long time ago and people are benefitting from that product it has been done and a lot can be done again having other diseases but the truth is once these concepts are found, does the individual have any role to pick how the results can be used. I think by and large it is easy to access and there is evidence on ground that diseases have been defined through the blood and we are benefitting from that, I think it should be easier to use for further genetic research. Thank you.

Participant 2: It is just very simple as just giving out your blood for further studies based on the blood you have donated or given out. As simple as it is, that is the simplest in all these donations because it is easy to extract the blood, just a little scratch you will see the blood come out and a lot of studies has been done on it though there are still these controversies too of opinions of whether it is right or wrong but all these are all tied to religion and culture. Thank you

Participant 5: the donation of blood is quite common, most people are familiar with the acceptance of obtaining blood either for investigative purposes or research is much easier to do, similarly in orthodox medicine is relatable trusted with tissue or organ compared to those that are not in the orthodox practice. So some of the traditional beliefs of people using parts for fetish reasons are less expressed but people believe that those in orthodox will not do such things but then the fear still exist especially among the very illiterate ones they may still relate the fetish beliefs they have concerning what blood or its products or tissue can be used for. They may be skeptical thinking that what is obtained out there may be obtained when ****and of course appropriate enlightenment of such people will go a long way erase their fears. So there has to be trust, must be a trust to *** the fears of the participants that whatever they donate will be used both for them and the society at large. Thank you.

Participant 3: well blood is generally accepted *** to most generality of people but certain religious groups are still reluctant towards blood donation even some *** of Christians, we know about the Jehovah witness and their reluctance towards blood donation. In fact, the fact that certain ***** are being developed to replace blood donation**** aside that most generality of people for blood donation, the treatment for research purpose be it genetic *****

6.

Participant 6: I think compared to donating blood, I mean, brain for stroke. This is much easier as I think the common man will agree to give his blood, if his father or any relation died of stroke and he believes he is at risk and they say they can study the risk from his blood, he can easily donate his blood for that research and I believe if an answer can be found for stroke by genetic engineering into blood is easily accessible, I’ll accept that 100% rather than agree to somebody’s brain being taken for research**** if he is alive of course, if the person has demised and there’s adequate consent, the brain can be taken but I think blood sample is very accessible, is easy to store and transport without detrimental effect of the person donating but most times investigations are done with… a drop of blood can do so much in the be investigation so I think this can be encouraged and the populace will really not resist much about sampling their blood for research for stroke. Thank you.

Participant 5: quite a number of people in the society are aware of the consequence of stroke, many families or people have either experience it or have seen families who have people with stroke and they know how detrimental it is to people who have it an each time they go the hospital they are told, we can’t do much, this thing just give it time, you may recover completely or partially or they may not be recovering, there’s very little we can do other than lowering the blood pressure but the outcome of the brain damage is not predictable but the recovering there’s nothing one can do about it. In hearing that one, anyone who has had experience with a stroke patient will be more willing for research to be done in such patients in other to look for a solution to stroke which is still existing at the moment and if perhaps an additional question, I don’t know if it’s there, if people are asked if they have ever cared for a patient with stroke or if they have anyone close to them who has stroke and will they be willing for any part of this patient to be used for the purpose of research in order to find treatment or cure or prevention for stroke, that might be helpful. Thank you.

b.

Participant 2: The response or reactions of people will vary**** but I think the issue of education and enlightenment that we have raised will help in solving this problem if we take the issue of enlightenment and education very serious, the attitude, the disposition of people towards that I feel will change. So we have a job at hand here to do, the issue of educating the persons particularly when somebody has this challenge, this stroke challenge, we’ll educate the person the need for him to cooperate and also assist the effort towards the study of trying to improve**** like the solution to the problem I think it will help like the need for enlightenment and education. Thank you.

7. Tell us what you know about informed consent

Participant 2: informed consent is like getting the approval, getting the blessings, getting the support of the person before anything either negative or positive is done or further done in any action he is involved or if he is not personally involved, someone close to him who is either a minor or a major is involved, because there are instances where a major will be in a particular state that he cannot give an opinion he cannot give his consent so somebody has to give his consent on behalf of the person though a major and in cases on a minor whose consent cannot be obtained, it will require the consent of the parents or the guardian or somebody else to give such a consent. Consent simply means having the approval, the blessings, the consent of the person before a particular activity is carried out. That consent is going to be informed if that person understands in full, completely, in totality what he is going into, it is at that point that one can say that that consent is informed. Thank you.

Participant 5: yes, for a consent to be truly informed, informed consent, it has to be void of coercion because to voluntarily obtain such a consent there must be full disclosure and the complete information must be provided regarding the purpose for this research, that means, this… and you must be sure that individual truly understands, to the extent that if you must speak to the person in the language they understand, that, and even insist on the feedback, get a feedback and be sure the person understands what you are saying. Full disclosure also entails****of your action must be declared in full, you must not hide any information and the person must have the capacity, that is to understand what the implications or procedure you wish to carry out. So if it lacks capacity that consent is not valid clearly that means it must be mentally aware, just like number 2 said. So if those characteristics are not there or those aspects of an informed consent then such a consent is not valid. The other aspect is How true or how valid is the consent obtained from an individual with stroke let’s say who is unconscious or lacks the capacity to understand, he may be conscious but aphasic that means unable to speak or even understand, now the consent you obtain here may be from a relative, how valid is that consent for an adult just because he lacks the capacity. The individual giving the consent maybe need to part or, maybe by a legal person in that aspect for it to be valid, especially for the purpose of research so for the purpose research, that is on stroke one will have to be very careful about the way consent is obtained or proposed and how legal the consent is in respect to the research. Thank you.

Participant 6: I think number 2 and 5 have extensively defined what informed consent is and the information given has added to that. I think any informed consent for it to be fully valid must be attested, must be documented and the individual should or somebody representing him has to sign that document for it to be valid, that’s what I will add to it.

Participant 5: the various types of consent you have mentioned, for any researcher their wish would be to have the first and second, which is the generic and the broad consent because it gives you a lot of free will and particularly for the concept of bio banking when you talk about a bank it means you have a tissue bank or biologic bank where different researches can access that tissue for the purpose of research and the scope of research is why and that tissue can be used for literally any of the research that is possible that means if you must give your consent for bio banking it has to be unrestricted if truly it should be a bank because it’s like going there to make withdrawals so bio banks are usually a tissue or biology material bank where people seek for permission and use specific stored tissue consent do this tissue are stored forever, indefinitely, there are facilities for that in modern day research so definitely it should be the wish of every researcher to have that so that he can have a lot liberation to use that but on the other hand, people want their tissue or their biological product to be respected and not just be used outside the consent given, so the other three tend to protect the wishes of the individual and they are easier to obtained than the broader ones. People are more willing to accept a restricted consent well defined********** such consents than to give for broad one and this particularly so for our environment so to give a broad consent in our environment will be much more rigorous, more difficult, will require more interaction with such people, more education, more time spent to obtain such compared to the restricted one. A lot of people have discussed about it. Thank you.

Participant 6: the thing with informed consent is that at times you cannot get it at one sitting, you have to do multiple sitting with educating the individuals, giving them options, at times that particular individual giving the consent may have to take time, assimilate and even make consultations especially with family members before they ultimately give, so for the purpose of this research, I think we looking at intricacies of it, sampling tissues, I think we need to give the respondents time and we have to give considerations to that sometimes you need to do it 2, 3, 4, or even 5 times and you may have to see the person with a spouse or a parent especially if it the woman that will give the consent, the husband need to be there, if it is child even if he is not a minor the parents need to be there to take part in that decision so multiple sections and multiple individuals will be necessary and that will prolong your period of research and I think you have to give that be serious consideration. Thank you

Participant 5: thanks to the last contributor, we looked at consent in terms of practice of surgery here and we did highlight the peculiarities**** peculiarities from our interactions with the patients about the peculiar cultural nature of our society which a lot of people let’s say a child, even an adult, they relinquish their consent or their ability to give to consent to an older person in the family, that I may not agree with what you are proposing but I may have to ask my father, if my father agrees no problem we can go ahead or a husband, even if the woman is willing to consent, if the husband refuses**** because of the peculiarity of the society and so these things would have to be put into consideration when obtaining consent to know that there are a lot of peculiarities in our environment which you have to negotiate to obtain a proper consent. Thank you.

Participant 1: if you look at our situation, our people lack of awareness, so if you take culture seriously based on… like eh… if you at Zaria and our rural areas so people didn’t understand all kinds of things. So it’s good to organize or settle a team which is going round to enlighten people of these things, I think it’s more*****

8.

Participant 6: blood is made up of many components as you rightly mentioned and talking about precision medicine, when a person needs blood at times it’s only a particular part of it he requires even if the whole blood will amount to wastage, yeah. For example, if there are 5 components and he needs only one, they will give him 5, so why give him 5 why don’t you take the other ones and give others. It has been in existence for quite a long time, it is safe. For example, if a person has some bleeding abnormalities that has to do with some components or he has some deficiency of the clotting factor, you don’t have to give him the red cells, the white blood cells, what he needs is the perhaps the plasma which is in form of fresh frozen plasma so that can be taken specifically to that individual for the benefits he requires. It is safe, it has been in existence and so many people have benefitted. Another advantage of such type of storage of blood into the various component is that certain components may not be acceptable to an individual’s body or system but when you take only one component, perhaps it may not cause much individual**** but on the averse side is that for you to get such components in the right quantities**** platelets themselves or **** implants, you may have to pull a lot of **** maybe 5 or ten to get to one unit of that small component you want, so will also have its own disadvantage but it has been done and it is quite effective and its welcome err(interruption from the moderator). Let me add to that, I think my own view on the storage aspect is that they should be used either for research or for treatment which is both beneficial to man but in our local environment, my particular problem about it is the condition in which it is stored, it requires light continuously, uninterrupted power supply, we require specialized refrigeration at a particular temperature, in our environment not knowing our electricity, power fluctuations, when these blood are stored, will that continuous power be ensured so that when they are being used in their optimal form not that some of their component have degenerated or other things. And the other thing is proper collection as well as storage because for you to collect blood it must be screened, proper screening of the individual to make sure this blood is safe to be used for other individuals. If it is done blindly without proper screening then infections or other reactions may occur in the recipients as other people receive the blood, so I think these are things to be considered before the blood is stored and the condition in which it is stored.

Participant 4: there is need to also ensure that only professionals that are used to are used to blood samples or professionals that **** the issue of blood are the people that are allowed to handle collection, storage and what have you.

Participant 5: the question is what is your opinion on the storage of blood and its fractions. The lay man does not know whether blood has fractions or not, bottom line is I’m donating blood and I think some of the questions you asked earlier addressed the issue of blood, just blood without the issue of components. If an individual has given his blood, subsequently whether you fractionate it or use it as a whole to a large extent I don’t think it really influences whether a patient will give or not, he has given the blood already. So it’s a technical issue to start talking about components whether storing them separately, so I’m looking at this question, is it actually necessary if you have talked about blood donation and storage previously. Except if you are introducing something other than the blood or any other particular use of that blood other than the purpose of treatment or research or anything, because breaking it down to such little things and yet you are going to ask people who ordinarily may not know much about blood components may not add much value in the long run rather than just confusing the man of exactly what you’re talking about. So, a lay person really, blood is blood, “okay, I’m collecting your blood, this is what I’m going to use it for”. Thank you

Participant 6: before going to number 10, collecting blood for use in the future, the individual needs to know about something. You know, traditionally our people believe if I’m well, don’t go and analyze my, investigate me and discover things that will trouble me psychologically and physically like leave the sleeping dog to lie, I think it’s something that needs to be looked at before you tell people to donate blood like that and then you have to investigate them especially the screening process particularly the issue of hepatitis or HIV, some people will say Ah, I will just and discover that I have these things so*** respond to you psychologically so I think it’s something that we need to look at.

9.

Participant 6. I think the issue is to define to the populace what the purpose of collecting the blood and of course that will start from the issue of obtaining consent, most likely it will be the broad kind of consent to be informed*** so that the individual knows before giving out consent that his blood will not be restricted for use, it will be shared with other people, other researchers or the result will be shared(moderator: whether for commercial or non-commercial purpose) when it comes to commercial purpose then there will be issues, serious issues. The individual did not want to give part of his body or his blood for somebody to go and be making money with it, even if he has no control over it, even if he is told that it will not be for commercial purposes when the result id obtained from another an *** books may be developed and these books may translate into money and some people may want to benefit from that financial profit that has been obtained. So whatever way, I think the confidentiality of the information has to be respected. The source must not be disclosed so that the individual is protected because if something is discovered that may be detrimental let’s say there is a high placed individual in the community and the result is published and that discovery may undermine his position. That confidentiality must be protected. And it I think it must not be used for a commercial purpose particularly if the sample is to be given for research to another person not the primary researcher, it should be given for free unless the donor is going to benefit from it as well. When it comes to images and data collected as long as it’s of benefit to humanity, the outcome of the research will help in treatment, provided confidentiality is protected, it is a welcome idea, we want to get by some of these chronic illnesses, if somebody works in America, UK has the answer and requires information, maybe he has studied people in Europe or America but if we do not have information about Africa, in our locality, if giving them that sample or images will enable them define our own characteristics and help us develop new treatment peculiar to us with that precision medicine you are saying I think it’s a welcome idea but confidentiality must be protected.

Participant 2: I think that data sharing for the purpose of research is very important but that data has to be shared with questions where it will have relevance, it’s not just to share data for the purpose of sharing data, it has to be shared with places where there is peculiarity, where it will be useful. Where the genetic nature is same, similar, where it will be beneficial to the people you are sharing it with, let it not be let’s just share data, let’s just share information and that is why, where the issue of confidentiality comes in and then the issue of non-commercial purposes, I don’t think it will be right for me to take information of another person and then just sell it for the purpose of making profit, where the person that donated sacrificed and donated the information is left out of it. Thank you.

10.

Participant 4: returning of result will be good and will encourage future donors to key into this thing, because by the time you give out an organ or an information the person must have at the back of his mind that welcome back will be on the way, so it will encourage people it will educate them on the benefit of giving out data, giving out organ and what have you to really encourage them for them to actually there is an effective result of what they have done and some of them will take pride participating in such information.

Participant 2: I think that is where informed opinion or consent will still come in, it’s not everybody that willing to have such information returned to them, that may put some people off by information they get back, so it is important that the person’s consent is obtained let it be something that is done willingly, let be information you leave at their disposal so that if they are willing to come back for it fine an good then you disclose to them the outcome of you findings, it’s made available to them, that’s my mind. So in a better way leave it at their discretion, you tell them information is available and if they are willing to come for the information, there is no problem coming for them rather than taking it back to the because, this might raise another issue because they didn’t ask you to give it back to them and by giving them such information you have thrown them into such problem and other problem. (Means of relaying the information. Phone, mail, etc.) Visiting the hospital

Participant 5: feedback or returning of information to research participants is an important aspect and very delicate particularly if the findings are not positive findings, for findings that are positive, have no detrimental health effect or a stigma to any individual or a community. For detrimental ones, one has question, if it has negative one which maybe potentially harmful if we don’t intervene, that means if treatment or intervention is not done for such a finding you are putting the participants at risk, you have discovered the person has a problem and you fail to disclose it, you are infringing on some aspects of research malpractice. So in such cases confidentiality can still be maintained, the individuals should be contacted, called and appropriate information relayed to the individual and then appropriate steps taken because failure to disclose such information and knowing fully well it is harmful if you not disclose, like it’s a lot easier for you to print a disease you discovered incidentally than allowing it to take its cause of the individual. Ethically that is not right, you must disclose it and especially when there is a treatment available for it, if there is no treatment, it’s a disease condition that ********** matter, another thing is you may find a negative finding that may stigmatize a community but is not harmful, let’s say you do a genetic study and you find out that most of the men in the community are not the fathers of their children from the analysis, you wouldn’t take such information to the community, such information is best kept to the researcher. So one has to be very careful about the way you treat such information and I think a group like this will be very important to look into such incidental findings and how to relate it. Thank you

Participant 6: I think what I will add is, in relating this information if it must be done must be individualized, just like the example number 5 just gave, must be individualized and it better done physical, not by phone because you don’t know the person’s reaction might be, so it is better the person comes so that you can prepare them psychologically when the result is adverse. You may release some information and the person will just collapse and will require some treatment but if he is in the hospital environment then he can easily be treated and I think, if something adverse is wrong and treatment is available then the research should make effort either to direct the person involved to where he will get treatment or even try to treat the result of the research if ailment is discovered in their study and prevented, for example, in stroke if the ****, there is preventive method that is available even if not available locally, if it means going out that person should be accessed to accept that as his benefit to that. Thank you

11.

Participant 6: this is a new terminology; I’m hearing it for the first time. But of course looking at it like we did the first time with bio banking, we are talking about the right of an individual to life, rights to your own life, right to the components of your body, right, for it to be used in whatever form, right? Particularly for research, you have rights and that’s why a consent needs to be obtained so that these rights are protected when you have to give part of your life, part of your tissue, sample are going to be taken. The individual needs to understand those rights and then agree to those rights for they issue out any tissue or sample from his body for research or whatever purpose. Thank you

Participant 5: For one to have rights, rights must be protected. That means to have such rights, there must be legal frame work for the protection of such rights, I’m sure most countries have such laws that protect individuals from such, even if they don’t have they will be *****. An individual has the right over his body generally how or whatever he possesses, biologically or how the brain components are used. Rights of protecting them, how they are being used and so on, if such rights are violated, the individual can seek redress from appropriate legal bodies. Thank you

12.

Participant 5: in Nigeria I’m not sure of regulatory bodies that are charged with such responsibilities of regulating how we use bio-banking, and there are a lot of tissues and organs turn around most, a lot of tertiary institutions long term or short term basis and our regulations are generally weak, I don’t know to what extent we will have a functional law that guides this, I don’t know if we have any existing legal channels that is any law that specifically addresses that and it should be within the domain of the Federal Ministry of Health and collaboration with Ministry of Justice or something to actually have such laws and a body or a department within the ministry to regulate that, there may be but I’m not aware of any and even if they are I don’t think they are perhaps as effective as they are obtained in other developed world, they are strict towards those, that is the way those biological tissues are handled so that aspect of***** are over.

Participant 2: Personally, I don’t think I’ve ever touched anything on this particular area, have not touched any matter that****** but I won’t be surprised if there are laws, Nigeria is a country where we are very good at laws, it’s not the laws are not there, they are there but the problem we have in this country is implementing this laws. Implementation of the law has always been our problem so I know that having gone this far in our journey in medicine, I know that the laws are there and as beautiful as they are they can’t function on their own unless if there are bodies there to make sure that these laws are perfect so that is where the government also have to come in and then face the various agencies, government bodies we have to bring these laws to life because if we leave them they will just be **** they will end up being paper files they are just there, there is no use. That is why the persons concerned will wake the government from their slumber so as to bring these laws to life.

Participant 5: I have been opportune to work in a very patent research institute outside Nigeria, in the United states specifically, they have very strict regulatory authorities that monitor every aspect of research and they visit on a timely basis either impromptu visit of scheduled visit to ensure that people are complying, in fact before you are permitted to work in any research institute or engage in any form of research that include human subject or animals, you must be trained, certified, there are recommended training you must undergo and then you must know the laws as well before even engaging and then regulatory bodies consistently follow up to ensure that those laws are strictly adhered to and any laboratory that does not meet such requirements maybe are sanctioned and such labs are sealed****. Thank you

Participant 6: Concerning this, I think it should be the responsibility of any government of any nation and perhaps there should be international body that should regulate banking of tissues, so that guidelines are outlined so that the person, the donor is protected and also the way the tissues and organs as well as data generated from the are used are protected, just last month, someone sent me a post, earlier in this year a research lab in the United states was investigated, how they conduct their research, the lead researcher was given some official *** appointment so he could not really participate or monitor what was going in his lab because he was responsible for what was going on in that lab and so they published a lot of papers, some of these papers were recognized to have had some irregularities, they could not follow the legal or ethical issues and some of them were withdrawn. He was actually lucky to have not been punished because of lack of evidence to implicate him but of course attention was drawn to the fact that it’s not just enough to have your name in the paper, you must take responsibility and know everything that has been done in that research is ethically okay and you have contributed a lot of things to it. So I think there should be bodies, they have bodies that**** should be regulated, documented sharers, why I said this is you know in this our environment, parts and human trafficking, body parts trafficking is any international market from which people make lots of money, particularly in Nigeria people go to India for treatment, you go for one treatment before you know your kidney is harvested, you take yourself for one operation, they take your kidney and transport it and sell so when these bodies are there and they are working, they will be able to guide against the misuse of these tissues and organs that are going to be banked if not others will make it a very lucrative business that they will benefit from.

13.

Participant 5: Now before bio banking is documented in Nigeria, there should be a clear legal framework on which it will operate in Nigeria to avoid misuse, if they are existing it should be reviewed and to reflect present reality cause some of these things are new so the law may be a bit obsolete in existence*** and to soot a specific need of our country. If that is taken care of then infrastructure, bio banking is an expensive thing and it will not make sense to store very valuable biological products that only gets destroyed because facilities are not available, documents are not there, liquid **** regular supply of it is required for bio banking and a lot of other accessories so that, infrastructure is very important, talking about electricity, funding of such parts is very important, who bears the brunt of it because these are for nonprofit set up, so someone has to bear the cost of it, that means the government someway will have to partake in most of the cost and perhaps some research funding for both local and international funding may also contribute to finance such bio banks. In utilization of such bio banks *** clearly stated and then some of the regulation, so clear regulatory bodies must be available before such banks are established. Thank you

14.

Participant 6: It must be highly regulated, bodies must be there to regulate such a thing and it must be active and efficient not the usual *** and nobody is applying those laws.

Participant 5: These banks as said earlier are very difficult to maintain and it will not make sense for every institution to try to have one. We can put resources together, have a central bio bank where every institution can access, where every institution can store their products or their specimen, so the management of such will be easier and less expensive to maintain, so the simple one is for one or two around the country is the *** without having so many around the country that are inefficient or poorly managed. Thank you

Participant 4: For that to be established, it has to have****. A separate body or ministry has to be established to be solely responsible for that kind of bank. Its not something that comes under the present ministry of health without control. Let it have a separate ministry or body to handle it so that the responsibility, the outcome and management of such will be under that body.

Participant 3: the idea of bio banking should be done such that everybody is carried along, no matter you do there should be a disciplinary*** where people from the field of science, field of law, the community are all engaged. Awareness is created so that people accept it easily.

Participant 2: As important and special as this aspect of medicine is, I think it will be good if the government pays more attention to this aspect by training of persons particularly on this area both within and outside the country, just like other speakers have raised the issues of funding, the funding should not be on just infrastructure alone, it should be extended to building of human capacity, that’s experts on this particular illness so that we will have person whose area of specialization will be in this particular area. Thank you
