# Supplementary material for: Research participants’ perception of ethical issues in stroke genomics and neurobiobanking research in Africa
Source: PLoS One. 2025 May 6;20(5):e0292906. doi: 10.1371/journal.pone.0292906 (PMC12054916; doi:10.1371/journal.pone.0292906)
Supplement: S3 File — (ZIP) [file pone.0292906.s003.zip › Files for PLOS ONE - updated March 2025/Zaria_SIREN Stroke Free Controls_ FGD.docx]

**LAY PERSONS**

**QUESTION 1: WHAT DO YOU KNOW ABOUT GENETIC RESEARCH?**

ANSWER: To my own understanding genetic research is the studying of gene. Generational gene.

The research itself is what I don’t understand what it entails.

**QUESTION 2: WHAT IS BIO-BANKING?**

It is the collection of body part.

It is important because human beings have genetics similarities and can be used to collect stored cell or organ for people. The storing of such organ helps people who are deficient in organ or in that place.

Storing organs will help replace lost or deficient organ.

**QUESTION 3: WHAT IS PRECISION MEDICINE?**

Number one I have not heard any information about it, but I think people should be treated based on their genetic needs. It is the treatment based on the individual scale of preference according to the needs of the individual.

**QUESTION 4: What do understand by brain donation for research purpose?**

Number 8 this means the donation of brain for research purpose. Some people donate their brain for research purpose, this means when they die their family have no right to it. If you go advance country people donate their brain for research purpose.

Brain donation is good because it is help in treatment of diseases.

Religious wise is it good to donate brain.

People don’t like it. Some Christians and Muslims don’t like it. They don’t like body part to be removed.

Some when they go to mortuary they will check the body of their dead ones to see if any part has been removed. In some culture is it prohibited. Some people can sue a hospital to court if they discovered that some part of the body of the dead their dead ones is removed. But if the patient have made agreement with the hospital and signed a document for it, I think is it good.

So religious I think it is right because it is part of learning.

**QUESTION 5: What do you understand by blood sample donation for genetics research?**

Number 8: it is good because it will help to prolong the life span of a man. It increases the chance of man survival for example if someone needs blood and there is no one to give the person will die. But as result of banking if there is blood that has been donated which is the same as that of the sick person the person will survive.

Number 1: what I understand by this is someone giving freely his blood let say from a lineage to study the blood to know if the people actually live long. It good also it has advantage and disadvantage, for some lineage their male don’t live long so it can be used to study to determine what makes it so. In some cases they will tell you don’t marry from some place because they don’t live long. This can make the person feel one kind. In Igbo land some of the old men will tell don’t marry from this family because their male don’t live above 30 years.

So blood sample donation can address this issue if the research is done

Number 5: for example someone know it becomes a problem. The day you know where you belong it becomes a problem, it can help fix some problem because you will know your faith.

Religiously it is right

Number 2: I think there is nothing wrong his status, if someone know it will help the person to know which way out. There should be a way out prayer, medically, traditionally.

Number 7: medically I agree with that in the sense it will help us for future references so as avoid some kind of mistakes our fore father have made. In the other way round the disadvantage is that it is not good, finding out it might be a problem for the individual or family.

**QUESTION 6: Share with us your opinion and thought about blood sample donation for stroke genetic research.**

Number 7: like I said earlier it is good for what we about to venture into for us to know what we can be abstaining ourself from. It is neccesary to know for us know what we can stay away from. It will also create awareness.

Number 1: it is very very good because the rate at which people are having this stroke is of great concern. It is good too to use this blood of people who actually have the stroke to study and know what is the cause of it and it will enable them to tell other people how to prevent having it. It is good to me but there should be an agreement between the stroke patient and the person conducting the interview so that the person will not feel somehow. It is a very good thing in our environment especially now a days that young and old people- 30, 40 years are already having stroke.

Number 8: Generally anything that has to do with donation it is good to donate. The only problem I have is it everybody that will donate or only the patient that are having stroke. If it is only for the one that are having stroke then it is good to donate in order for them to know what causes it, how they can prevent their family from having the same thing and preventing it for future generation. I don’t think donating it religiously has any effect, if you have to donate, somebody needs it that is one thing I understand about donation. If you donate, your family or anybody close to you can receive.

**Question 7: Tell us what you understand by informed consent?**

Number 1: for my own understanding, in a layman language, informed consent, it means the person was contacted or informed that they are going to ask him question.

Number 7: to my own understanding, informed consent is an agreement between a researcher and a participant

Number 2: I feel informed consent is like someone agreeing to accept an interview or something

Any other opinion on informed consent?

Number 5: I see it as a cap or synopsis given to someone who is going to answer a question either to accept or to disagree with the interview.

**Question 8: What is your opinion on storage of blood samples and blood fractions for genetic research?**

Number 5: it is a good development in my own view because it is a type of research that will save life.

Number 1: it is good in the sense that it will save cost of the person travelling to go and donate the cost but another disadvantage is that I don’t know how long and how safe the storage is. You people in the medical line can tell us.

It is possible to store blood for many years, there is a way it can be done.

Number 7: I think it is good for genetic research.

I have a question, why storing blood before the research?

If you collect blood sample, it is possible to keep and do research on it for years.

For me it is not that good because it is not going to help the patient, or the person that donated the blood.

Number 1: I wouldn’t give my blood, because it’s for next generation.

Number 8: it is good to donate because they might find something that will cure your own child, so it is good to donate for children for the research to be done.

Number 6: it is good to donate so that you can help others.

**Question 9: Tell us what you know about sharing data, brain images as well brain tissue sample?**

Number 8: I think it is good. You need to share for them to do their own research, compare and they will bring their own tissue you will take yours to them, they will try to understand our own part of the world how our sickness is, how theirs are, we share information. By giving them is very good, by collecting is very good so that people will understand how peculiar or how different our sicknesses are for them to make their own research how to cure some kind of sicknesses that they might find and may not find here within our own research system.

Number 5: I see it to be a good thing even though there is advantage and disadvantage. The advantage is it will enhance knowledge, the disadvantage is that by you giving them, after making the research, and they discover some sickness, they might end up stopping you from coming to their place because they know you are having a sickness which they don’t want you to bring it to their own place. I remember a year Nigerians were stopped from Hajj simply because of one sickness which is called meningitis which I think is because they have idea of what is happening in Nigeria. And that does not mean anybody who goes there will transfer it to everybody in Saudia. To me it has its advantage and disadvantages but to me I will be in support of doing it.

Number 2: it is good so that the doctors can come to final conclusion about the sickness and the cure.

It is good it will enable the researchers to know the community or countries that are having the type of sickness more than other people.

**Question 10: Share with us your thoughts about return of individual results for incidental finding and which?**

Number 7: it is good to return the result to the individual so as to make the individual know his or her faith concerning the research work and for him to also give advise to others if need be.

Number 8: I think it is good, if it is accidental, I think it is not good. If he is the one that presented himself for the research.

I have a cousin who have been living with HIV he contracted from birth through his mother, he has been living fine unknowingly for 22 years until when he went for malaria test and was told he also has HIV, for 2 years now he has not been fine.

Number 1: to me you can seek the consent of the person where you done research, ask the person what if you get another finding. If the person say yes, or if the person say No.

Number 7: it is very okay to tell me my present status whether by mailing, text or so. If I am told I will know how to tackle it and be save in the future.

Number 5: whoever ask me for sample, infact I am not going to give if he will not give me result, and I will prefer it face to face, one on one not by text, mail or paper.

Number 4: I agree with number 5 to give me the result and it should be face to face

Number 3: I agree with my colleagues what they said

Number 6: I don’t want to know the result

Number 2: I prefer if the sample the patient gave, and you have another finding I think you should not tell the person face to face but someone close to him. They should tell me the result face to face.

**Question 11:**  **Explain your understanding of bio rights, any understanding, has any one heard of it?**

Number 8: I prefer to have right or autonomy over the blood or organ that I am giving. If it is not been used for the person it is donated for it should be given back to me. And if it is for research purposes, it should be for research purposes. I don’t want to have right over it.

Number 7: I think when I have a right, if it is not for the purpose of the research I gave it for, it should be given back to me. I don’t want it to be for another purpose.

Number 8: I think whatever research you want to do you can, do but if there is any financial implication over the research, you are going to produce a drug from it, I think I am entitled to any money from the drugs generated from the drugs as a donor.

Number 7: I concur with what the previous speaker said, with his idea about financial proceed.

I don’t have much right. If I donate and they take it for research, except I know where the research is done. I don’t think it is necessary

**QUESTION 12: What is your opinion about governance or regulations on bio banking. Do you think government should regulate it, are you aware of any regulation?**

**I don’t know**

Number 8: I don’t think there is any government regulation for now in Nigeria that regulate the donation of organs. I think there should be regulations for hospitals. I don’t think there is any punishment for any one whom steals a kidney.

**QUESTION 13: Explain possible intervention for implementation of bio banking, tell us your idea on how we can make bio banking work.**

Number 2: I think they should create a platform where people who want can login to know what they are supposed to do

Number 7: Awareness can be created for patient relatives visiting the hospital.

Number 5: I suggest there should be a form of rally to touch the local area

Number 7: I am also in the opinion of passing information in religious places, market places create awareness so that people will be aware of such things

Number 8: I think we should go through school, primary, secondary and tertiary institution, start the awareness in the grass root put it in the minds of our children to know that such thing exisits.

Number 4: Also give motivation to people

**QUESTION 14: Any other issue or concerns on the use of brain tissue for research? Any recommendation, concern or fear?**

Number 8: my only fear is if it is not going to be used for the research. I know they pay people money to buy their kidney, without telling them the implication. Researchers may start buying the samples will not go deep to tell you the consequences or implication of the research, they may use the research against people, they will just tell you the financial benefit part of it,

I suggest that government could come. We have abandoned dead bodies of robbers book haram members, if government will come donating such bodies to a research center whereby these bodies could be donated for research.

Number 5: I suggest that government should come in because we might end up abusing it as the researcher may even use samples for ritual not necessarily research again.

Well, I thank you all for coming, I thank you for participating, to this study. To end it, very small survey which you are just going to answer and at the end we are going to give you three thousand naira incentive for participating in this.
